# Asparagine synthetase regulates the proliferation and differentiation of chicken skeletal muscle satellite cells

**DOI:** 10.5713/ab.24.0271

**Published:** 2024-08-26

**Authors:** Hangfeng Jin, Han Wang, Jianqing Wu, Moran Hu, Xiaolong Zhou, Songbai Yang, Ayong Zhao, Ke He

**Affiliations:** 1 Key Laboratory of Applied Technology on Green-Eco-Healthy Animal Husbandry of Zhejiang Province, Zhejiang Provincial Engineering Laboratory for Animal Health Inspection & Internet Technology, Zhejiang International Science and Technology Cooperation Base for Veterinary Medicine and Health Management, China-Australia Joint Laboratory for Animal Health Big Data Analytics, College of Animal Science and Technology & College of Veterinary Medicine of Zhejiang A&F University, Hangzhou, Zhejiang, 311300, China

**Keywords:** AMPK, Asparagine Synthetase (*ASNS*), Chicken, Muscle Satellite Cell Differentiation, RNA-seq

## Abstract

**Objective:**

Asparagine synthetase (ASNS) is an aminotransferase responsible for the biosynthesis of aspartate by using aspartic acid and glutamine. *ASNS* is highly expressed in fast-growing broilers, but few studies have reported the regulatory role of *ASNS* in muscle development.

**Methods:**

To explore the function of *ASNS* in chicken muscle development, the expression of *ASNS* in different chicken breeds and tissues were first performed by real-time quantitative reverse transcription polymerase chain reaction (RT-PCR). Then, using real-time quantitative RT-PCR, western blot, EdU assay, cell cycle assay and immunofluorescence, the effects of ASNS on the proliferation and differentiation of chicken skeletal muscle satellite cell (SMSC) were investigated. Finally, potential mechanisms by which ASNS influences chicken muscle fiber differentiation were identified through RNA-Seq.

**Results:**

The mRNA expression pattern of *ASNS* in muscles mirrors trends in muscle fiber cross-sectional area, average daily weight gain, and muscle weight across different breeds. *ASNS* knockdown inhibited SMSC proliferation, while overexpression showed the opposite. Moreover, *ASNS* attenuated SMSC differentiation by activating the adenosine 5′-monophosphate (AMP)-activated protein kinase (AMPK) pathway. Additionally, 5-aminoimidazole-4-carboxamide1-β-D-ribofuranoside (AICAR) treatment suppressed the cell differentiation induced by siRNA-*ASNS*. RNA-Seq identified 1,968 differentially expressed genes (DEGs) during chicken SMSC differentiation when overexpression ASNS. Gene ontology (GO) enrichment analysis revealed that these DEGs primarily participated in 8 biological processes, 8 cellular components, and 4 molecular functions. Kyoto encyclopedia of genes and genomes (KEGG) pathway analysis identified several significantly enriched signaling pathways, such as the JAK-STAT signaling pathway, tumor necrosis factor signaling pathway, toll-like receptor signaling pathway, and PI3K-Akt signaling pathway.

**Conclusion:**

ASNS promotes proliferation while inhibits the differentiation of chicken SMSCs. This study provides a theoretical basis for studying the role of ASNS in muscle development.

## INTRODUCTION

The development of skeletal muscle plays a crucial role in determining the meat production performance of broiler chickens. The proliferation and differentiation of skeletal muscle cells form the foundation of broiler chicken muscle development [1]. Identification of the genes and regulatory factors related to muscle cell growth and development will provide important theoretical references for molecular breeding and genetic improvement of broiler chicken varieties.

Asparagine synthetase (ASNS) primarily functions within the cytoplasm of cells and utilizes glutamine as a nitrogen source. Through an adenosine triphosphate (ATP)-dependent reaction, it converts aspartic acid and glutamine into asparagine and glutamic acid [2]. It has been found that *ASNS* might play a key role in the process of porcine skeletal muscle development which was promoted by β-adrenergic agonists [3]. Furthermore, the expression of the *ASNS* in the muscles of fast-growing broiler chickens is significantly higher than that in slow-growing ones [4]. Transcriptome sequencing analysis reveals that *ASNS* is a potential target gene of *miR-181a-5p*, possibly playing a role in the growth of primary chicken myoblasts [5]. However, the role of the *ASNS* gene in chicken muscle growth and development is unclear.

In several tumor cells, the *p53* gene downregulates *ASNS*, impacting the balance of intracellular aspartate (Asp) and asparagine (Asn), thereby providing feedback regulation to mediate *p53* activation through adenosine monophosphate (AMP)-activated protein kinase (AMPK) phosphorylation [6]. The phosphorylation of AMPK inhibits the differentiation of C2C12 myoblast cells [7]. So, we speculated that *ASNS* might regulate chicken muscle growth through activating the AMPK signaling pathway.

The RNA sequencing technique (RNA-Seq) has been a beneficial tool for exploring important genes and signaling pathways related to chicken skeletal muscle development [8]. Meanwhile, the chicken skeletal muscle satellite cells (SMSCs) are powerful *in vitro* cellular models for studying gene function during cell proliferation and differentiation [9]. Therefore, we explored the relationship between *ASNS* and the AMPK signaling pathway in chicken SMSCs, screened genes and signaling pathways influenced by *ASNS* during chicken SMSC differentiation through RNA-Seq. This aims to provide new insights into the mechanisms underlying chicken skeletal muscle development.

## MATERIALS AND METHODS

### Ethics statement

All animal procedures were approved by the Ethics Committee for Animal Experiments of Zhejiang A&F University (Ethical license number: ZAFUAC2023034) and were performed in accordance with the Guidelines for Animal Experimentation of Zhejiang A&F University (Hangzhou, China).

### Experimental animals and tissues

Ten chickens from each of three Chinese indigenous broiler breeds from Zhejiang Province – Lingkun Chicken, Xianju Chicken, and Xiaoshan Chicken were selected. They were reared under the same feeding conditions until 180 days at a testing station in Hangzhou, after which they were uniformly slaughtered. There are 5 roosters and 5 hens for each breed, and the initial weights are similar. The individuals within the breed came from the same lineage. The average daily weight gain of chickens of each breed and gender was calculated by dividing the weight gain during the rearing period by the number of rearing days. All broilers were individually euthanized by cervical dislocation and exsanguination. Leg muscles were collected from both sides of all experimental chickens. The gastrocnemius muscle was fixed in 4% paraformaldehyde for hematoxylin and eosin (H&E) staining, while the other were rapidly frozen in liquid nitrogen and stored at −80°C for RNA extraction. Three roosters with similar body weights of the same breed were randomly selected. Samples were collected as 3 replicates from 8 tissues of one broiler breed for RNA extraction. In the tissue real-time quantitative reverse transcription polymerase chain reaction (qRT-PCR) experiment, three individuals from each breed were used for detection. In the cell qRT-PCR and western blot experiments, the cells used for the tests were isolated from the same chicken. Fertilized eggs were purchased from the same farm and incubated in our laboratory for 13 days before being used to isolate cells.

### Cell culture

The method of collecting chicken SMSCs has been comprehensively described in our previous research [9]. The growth medium (GM) consisted of 84% Dulbecco’s modified Eagle medium (DMEM)/F12 (HyClone, Logan, UT, USA) supplemented with 15% fetal bovine serum (Thermo Fisher, Shanghai, China) and 1% penicillin-streptomycin (Solarbio, Beijing, China). The differentiation medium (DM) was composed of 97% DMEM/F12, 1% penicillin-streptomycin (Solarbio, China), and 2% horse serum (HyClone, USA). SMSCs were first cultured in GM until reaching a cell confluence of 70% to 80%, and then induced to differentiate using DM. All cells were incubated in 5% CO_2_ at 37°C.

### Hematoxylin and eosin staining

After fixing in 4% paraformaldehyde, the gastrocnemius muscle samples were dehydrated in alcohol, followed by tissue decolorization using xylene. Subsequently, the samples were embedded in paraffin. Paraffin blocks were subsequently sliced into sections, each measuring approximately 4 to 5 μm in thickness, and affixed onto glass slides. The glass slides were incubated in hematoxylin solution for 10 min and then stained with eosin solution for 3-min. The slides were immersed in 70% ethanol, 90% ethanol, 100% ethanol, and xylene for 20 s, 20 s, 1 minute, and 3 min, respectively. After air drying, the mounting medium was applied, and coverslips were sealed. The fluorescence inverted microscope was used to capture images. The muscle fiber characteristics was analyzed by Image J software (National Institute of Health, Bethesda, MD, USA). For each group, 3 slices were prepared and the diameters of 5 muscle fibers in each slice were measured. The number of muscle fibers in 3 different fields of view for each slice were counted and the total area of muscle fibers in these fields of view were measured. The cross-sectional area of the muscle fibers was calculated using the formula: average area = total area of muscle fibers/number of muscle fibers. The muscle fiber density was calculated by measuring the area of each field of view and counting the number of muscle fibers within each field, then converting this to the number of fibers per square millimeter.

### RNA oligonucleotides and plasmids construction

The RNA oligonucleotides, including small interfering RNA (siRNA) and siRNA negative control (NC), were designed and synthesized by GenePharma (Shanghai, China). The sequences were shown in Table 1. According to the NCBI Reference Sequence: NM_001030977.2, pcDNA 3.1 *ASNS* expression vector was designed and constructed by TsingKe (Hangzhou, China) using the pcDNA-3.1 vector (Invitrogen, Carlsbad, CA, USA).

### Cell transfection

Cells were seeded into a six-well plate at approximately 1×10^5^ cells/cm^2^. When the cell confluence reached 70% to 80%, siRNA or plasmid transfection was performed according to the instructions of the Lipofectamine 3000 kit (Invitrogen, USA). After 4 to 6 h, serum-free medium was replaced with GM or DM. Proliferating cells were harvested after 24 h, while differentiating cells were collected after 72 h. The AMPK activator AICAR powder was bought from Beyotime (Shanghai, China), dissolved in dimethyl sulfoxide (DMSO), and diluted to 1 mM in the culture medium. For co-transfection, cells were transfected with siRNA-*ASNS* or NC, and the original medium was replaced with 1 mM AICAR or DMSO-containing medium after 4 to 6 h.

### 5-Ethynyl-2′-deoxyuridine assay

Cells were seeded into a 12-well plate with pre-coated cell slides. After transfection for 4 to 6 h, the chicken SMSCs were cultured in fresh GM containing 10 mM 5-ethynyl-2′-deoxyuridine (EdU) for 24 h. Following the EdU Apollo 567 kit manual, cells were fixed, permeabilized, and stained. Using a fluorescence inverted microscope, three randomly selected fields of each treatment were observed. EdU-positive nuclei and total nuclei were quantified.

### Flow cytometry

After 24 h transfection, cells were washed with PBS (phosphate-buffered saline?) and then fixed in pre-chilled 70% ethanol at 4°C overnight. Subsequently, cells were washed and 100 μL of RNase A solution was added following the instructions of the DNA content detection kit. Then, 400 μL of 50 mg/mL propidium iodide (PI) solution (Solarbio, China) was added to the cells, and then were incubated at 4°C in the dark for 30 min. BD flow cytometer was utilized to determine the cell cycle distribution. Data analysis was conducted using ModFit software.

### Immunofluorescence staining

Cells differentiated for 0 to 6 days were used for morphological observation or immunofluorescence analysis. Cells were washed three times with precooled PBS for 5 min each time, followed by fixation in 4% paraformaldehyde for 15 min. Then, cells were permeabilized with 0.2% Triton X-100 for 10 min, blocked with serum for 30 min, and incubated overnight at 4°C with the anti-MYH primary antibody (Santa Cruz Biotechnology, Santa Cruz, CA, USA). Subsequently, fluorescent secondary antibodies (Thermo Fisher, China) were added and incubated at room temperature in the dark for 2 h. After washing, 4′,6-diamidino-2-phenylindole (DAPI) (Invitrogen, USA) was added and incubated at room temperature for 15 min to stain the cell nuclei. The samples were observed using a fluorescence microscope (Olympus, Tokyo, Japan). Image J software (National Institute of Health, USA) was used for calculating myotube area. Myotube area (%) refers to the percentage of the fluorescent area of myotubes relative to the total area of the field of view. The fluorescent area of myotubes were determined by carefully tracing around myotube structures using Image J software (National Institute of Health, USA).

### Western blot analysis

Total protein was extracted using radio immunoprecipitation lysis buffer (Beyotime, China). The protein concentration was determined using the BCA assay kit (Nanjing Jiancheng, Nanjing, China). Proteins were separated by 12% sodium dodecyl sulfate-polyacrylamide gel electrophoresis gel and transferred onto polyvinylidene fluoride polyvinylidene fluoride membranes (Millipore, Billerica, MA, USA). After blocking with 5% bovine serum albumin (Beyotime, China) at room temperature for 2 h, membranes were incubated with primary antibodies overnight at 4°C. After washing, membranes were incubated with secondary antibodies at room temperature for 1 hour. The blots were visualized using ECL reagent (Thermo Scientific, Waltham, MA, USA) and exposed using a chemiluminescence detection system (Tanon, Shanghai, China). Image J software (National Institute of Health, USA) was used to analyze the data. The primary antibodies included: TBB5 Antibody (AM1031a; Abcepta, Suzhou, China), ASNS (E6C2C) (#92479; Cell Signaling Technology, Danvers, MA, USA), MYH (H-300) (sc-20641; Santa Cruz Biotechnology, USA), AMPKα (D5A2) (#5831; Cell Signaling Technology, USA), phospho-AMPKα (Thr 172) (#2535; Cell Signaling Technology, USA). The secondary antibodies used were HRP-labeled Goat Anti-Rabbit IgG (A21020; Abcepta, China), HRP-labeled Goat Anti-Mouse IgG (A21010; Abcepta, China).

### Quantitative real-time reverse transcription polymerase chain reaction

Total RNA was extracted using TRIzol reagent (Invitrogen, USA) following the reagent instructions. Subsequently, the concentration and purity of the RNA were assessed using the Agilent Bioanalyzer 2100 spectrophotometer (Agilent Technologies, Santa Clara, CA, USA). The RNA integrity and concentration were confirmed by the specified criteria (OD 260/280>1.8, OD 260/230>2.0), the 5X All-In-One RT MasterMix transcription kit (abm, Zhenjiang, China) was used for cDNA synthesis. Quantitative real-time RT-PCR was conducted using Eva Green 2x qPCR Master Mix (abm) through the CFX96 instrument (Bio-Rad, Hercules, CA, USA). Differential expression analysis was performed using the 2^–ΔΔCT^ method [10]. The primer sequences are listed in Table 2.

### Library construction and sequencing

The transcriptome library was constructed using the TruSeqTM RNA Sample Preparation Kit (San Diego, CA, USA) with 1 μg total RNA which was obtained 3 days after the SMSC differentiation. The poly A selection method was used to isolate messenger RNA (mRNA) using oligo (dT) beads, followed by fragmenting the mRNA through fragment buffer. Subsequently, the SuperScript Double-Stranded cDNA Synthesis Kit (Invitrogen, USA) and Random Hexamer Primers were used to synthesize double-stranded cDNA. Next, the synthesized cDNA underwent end-repair, phosphorylation, and the addition of ‘A’ bases. A 300 bp target fragment library of cDNA was selected on a 2% low-range Ultra agarose gel. PCR amplification was conducted by 15 cycles using Phusion DNA polymerase (NEB, Ipswich, MA, USA). After quantification, the paired-end RNA-seq libraries were sequenced on an Illumina HiSeq Xten/NovaSeq 6000 sequencer (2×150 bp read length). The PC represented control group, which was labeled as PC1, PC2, and PC3, while the experimental group was represented by PA, which was labeled as PA1, PA2, and PA3.

### Sequencing quality assessment and differentially expressed gene screening

The SeqPrep and Sickle software were used to eliminate low-quality sequencing reads from the raw data. The fastp tool was utilized for calculating Q20, Q30, GC content, and sequence repeat levels. All subsequent analyses were grounded in high-quality clean reads. The chicken’s genome sequence was acquired from the NCBI database (http://asia.ensembl.org/Gallus_gallus/Info/Index).

Subsequently, the obtained clean reads were aligned with the chicken reference genome using Hisat2. To determine the differentially expressed genes (DEGs) between the two groups, the expression levels of each gene were quantified in terms of transcripts per million reads. Gene abundance was performed using RSEM (http://deweylab.biostat.wisc.edu/rsem/). Differential expression analysis was carried out using the DESeq2, DEGseq, and EdgeR methods. Genes displaying |log2FC| >1 and a Q-value ≤0.05 (for DESeq2 or EdgeR) or a Q-value ≤0.001 (for DEGseq) were considered as DEGs.

### Gene ontology and kyoto encyclopedia of genes and genomes pathway enrichment analysis

The Goatools software, based on the gene ontology (GO) database (http://www.geneontology.org/), was used for GO enrichment analysis. The analysis involved classification annotation into three categories: biological process (BP), molecular function (MF), and cellular component (CC). The Kyoto encyclopedia of genes and genomes (KEGG) database (http://www.genome.jp/kegg/) and the KOBAS software (version 2.1, Peking University) were used to statistically assess significant enrichment of DEGs within KEGG pathways.

### Statistical analysis

The experimental data are mean±standard error of the mean. Each experiment was conducted with three biological replicates. One-way analysis of variance was performed on all data using IBM SPSS Statistics 20.0 (2010, SPSS Inc., Chicago, IL, USA). p-value <0.05 indicates significant difference, and p-value <0.01 indicates high significance.

## RESULTS

### Comparison of growth traits, muscle fiber characteristics and *ASNS* gene expression in different broiler breeds

**Figure 1:**
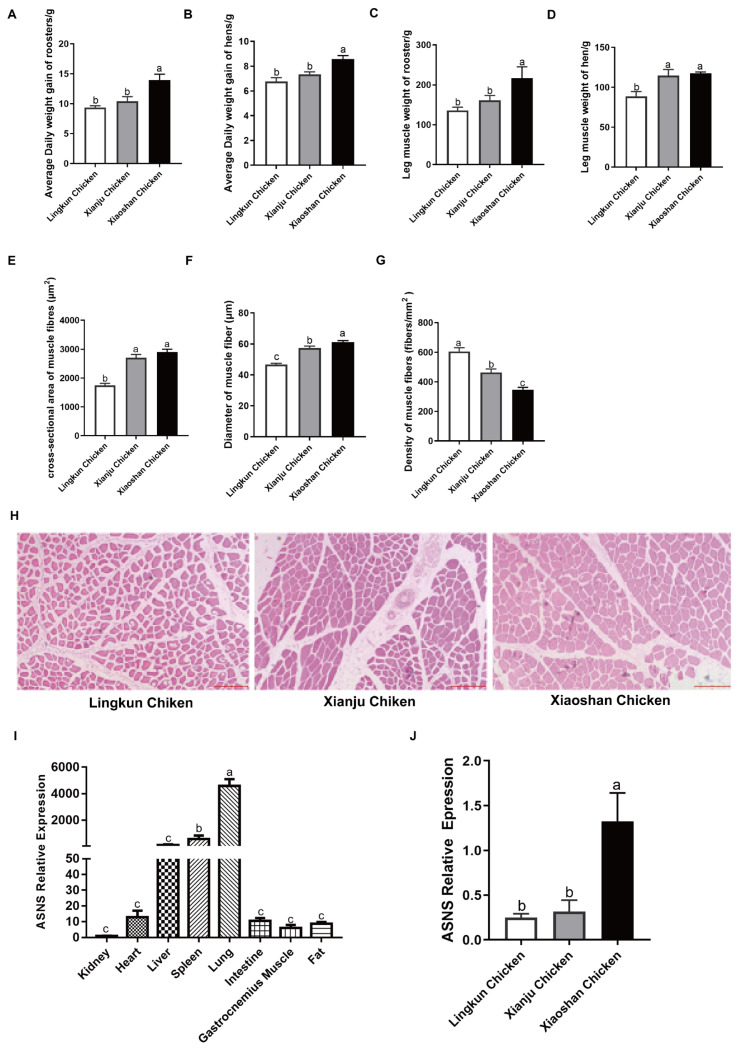
Growth traits, muscle fiber characteristics and expression profile of *ASNS* in broiler. (A) Average daily weight gain of roosters. (B) Average daily weight gain of hens. (C) Leg muscle weight of roosters. (D) Leg muscle weight of hens. (E) Cross-sectional area of leg muscle fibers. (F) Diameter of leg muscle fibers. (G) Density of leg muscle fibers. (H) H&E staining sections of the gastrocnemius muscle. (I) Expression profiles of *ASNS* genes in different tissues. (J) Expression profiles of *ASNS* genes in the gastrocnemius muscle of different chicken breeds. ^a–c^ Different letters indicate significant differences (p<0.05). H&E, hematoxylin and eosin; *ASNS*, asparagine synthetase.

To explore the role of *ASNS* in broiler muscles, the expression pattern was determined among three Chinese indigenous broiler breeds with different growth rates. The results indicated that the average daily weight gain of roosters and hens, as well as the leg muscle weight of Xiaoshan rooster chickens, were significantly higher than those of Xianju chickens and Lingkun chickens (Figure 1A–1C). Conversely, the leg muscle weight of Lingkun hen chickens was significantly lower than that of Xiaoshan chickens and Xianju chickens (Figure 1D). The Lingkun chickens had the smallest cross-sectional area of muscle fibers (Figure 1E, 1H). Xiaoshan chickens had the largest muscle fiber diameter, followed by Xianju chickens (Figure 1F, 1H). However, muscle fiber density exhibited the opposite trend (Figure 1G, 1H). Otherwise, *ASNS* was not only highly expressed in the liver, spleen, and lungs, but also showed relatively high expression in the heart, intestines, adipose tissue, and gastrocnemius muscles (Figure 1I). Furthermore, the mRNA level of *ASNS* in the gastrocnemius muscle of Xiaoshan chickens was higher than that in Lingkun chickens and Xianju chickens (Figure 1J), consistent with the trend observed in the leg muscle weight of rooster chickens. These findings suggest that *ASNS* may play a potential role in the growth and development of chicken skeletal muscles.

### Morphological observation and identification of chicken skeletal muscle satellite cells

**Figure 2:**
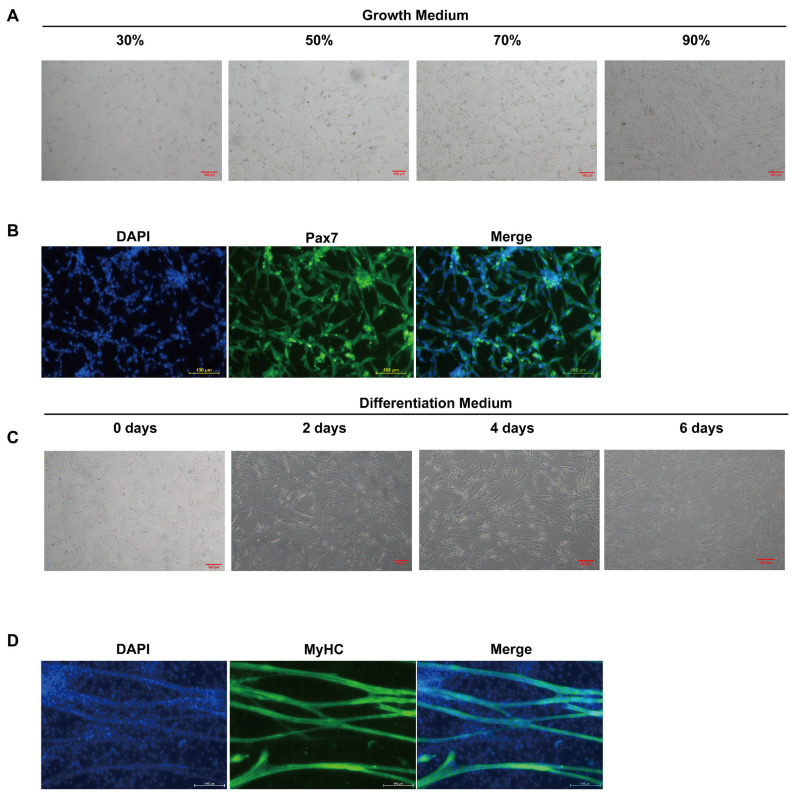
Morphological observation of proliferation and differentiation of chicken SMSCs. (A) Morphological observation of cell confluence from 30% to 90%. (B) Pax7 was detected by immunofluorescence. DAPI was used to stain nuclei; a merged fluorescence and DAPI image is also shown. (C) Morphological observation of cells 0 to 6 days after induction of differentiation; (D) MyHC was detected by immunofluorescence. DAPI was used to stain nuclei; a merged fluorescence and DAPI image is also shown. SMSCs, skeletal muscle satellite cell; DAPI, 4′,6-Diamidino-2-phenylindole; MyHC, myosin heavy chain.

The cells became spindle-shaped when the cell confluence reached 30% (Figure 2A). Subsequently, the cells started to proliferate and reach the confluence level of 50%, 70%, and 90% (Figure 2A). The results demonstrate that *Pax7*, a marker for quiescent and proliferating muscle satellite cells [11], is positively expressed within the cell nuclei (Figure 2B). Thus, the isolated cells were SMSCs. The SMSC fused to form myotubes during cell differentiation (Figure 2C). MyHC fluorescence staining confirmed the formation of myotube (Figure 2D). Therefore, the chicken SMSC model has been successfully established.

### ASNS has a positive effect on chicken myogenic proliferation

**Figure 3:**
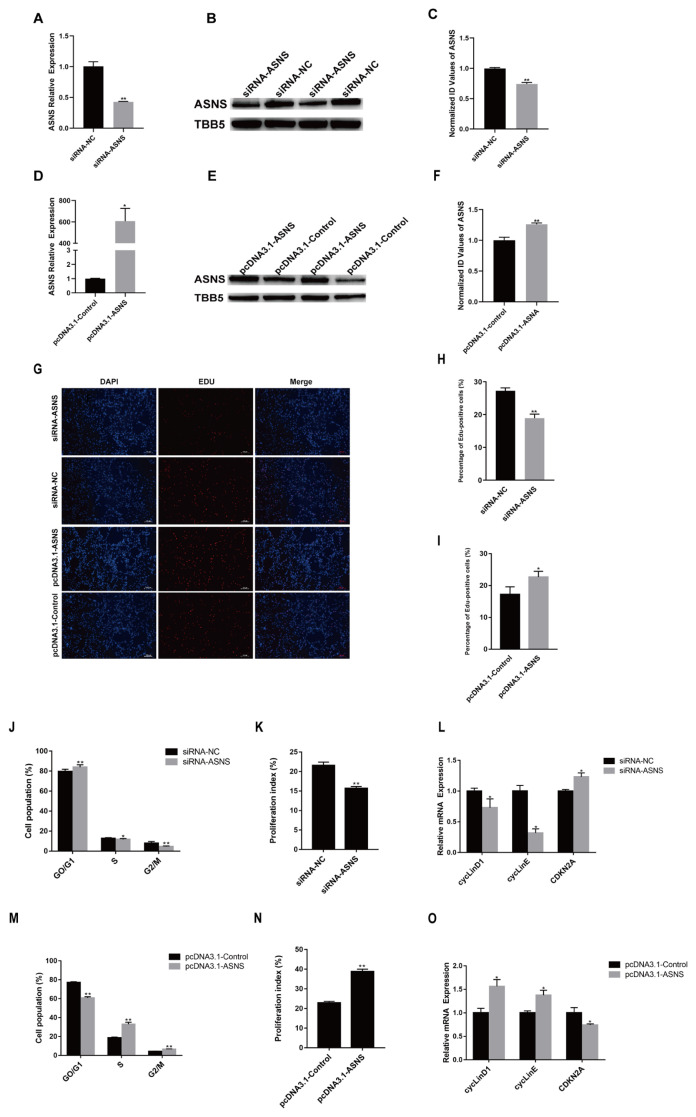
*ASNS* promotes chicken myoblast proliferation. (A) *ASNS* mRNA level at 24 h post-transfection in GM. (B) ASNS protein level at 24 h post-transfection in GM. (C) The densitometry value of the treatment group compared to the control group. (D) *ASNS* mRNA level at 24 h post-transfection in GM. (E) ASNS protein level at 24 h post-transfection in GM. (F) The densitometry value of the treatment group compared to the control group. (G) After transfection for 24 h, cells were fixed for EdU staining (red). Scale bar, 100 mm. (H) Proportion of EdU-positive cells after transfection siRNA-*ASNS* or NC for 24 h. (I) Proportion of EdU-positive cells after transfection pcDNA3.1-ASNS or pcDNA3.1-Control or for 24 h. (J) Cell cycle distribution by PI flow cytometry after transfection siRNA-*ASNS* or NC for 24 h. (K) Proliferation index was calculated as [(S+G2/M)/(G0/1+S+G2/M)]×100%. (L) Expression of cell cycle-related genes at 24 h post-transfection. (M) Cell cycle distribution by PI flow cytometry after transfection pcDNA3.1-*ASNS* or pcDNA3.1-Control or for 24 h. (N) Proliferation index was calculated as [(S+G2/M)/G0/1+S+G2/M)]×100%. (O) Expression of cell cycle-related genes at 24 h post-transfection (* p<0.05; ** p<0.01). *ASNS*, asparagine synthetase; GM, growth medium; PI, propidium iodide; NC, negative control.

To assess the role of ASNS in chicken SMSC proliferation, ASNS knockdown or overexpression cells were generated using synthetic siRNA or pcDNA 3.1, respectively. The TBB5 also known as β-tubulin, was widely used as a loading control in western blots to normalize the abundance of the protein in muscles [12]. The expression of *ASNS* was decreased after transfection (Figure 3A–3C). Conversely, transfection with pcDNA3.1-*ASNS* significantly upregulated *ASNS* level (Figure 3D–3F). EdU detection results indicated that the proportion of positive cells decreased in the siRNA-*ASNS* group compared to the control group, while the pcDNA3.1-*ASNS* group showed the opposite trend (Figure 3G–3I). Moreover, cell cycle analysis revealed that *ASNS* interference arrested cells in the G0/G1 phase, resulting in a significant decrease in the cell proliferation index (Figure 3J, 3K). In addition, *ASNS* overexpression promoted the number of cells in the S and G2/M phases, accompanied by a significant increase in the cell proliferation index (Figure 3M, 3N). At the mRNA level, the siRNA-*ASNS* transfection group showed a significant downregulation of two cell cycle activators, G1/S-Specific cyclin-D1 (*cyclin D1*) and E-type cyclins (*cyclin E*). Conversely, the expression of *CDKN2A*, a cyclin-dependent kinase inhibitor, was significantly upregulated (Figure 3L). The pcDNA3.1-*ASNS* transfection group showed opposite results (Figure 3O). These findings indicate that *ASNS* promotes chicken SMSC proliferation.

### *ASNS* inhibits chicken skeletal muscle satellite cell differentiation

**Figure 4:**
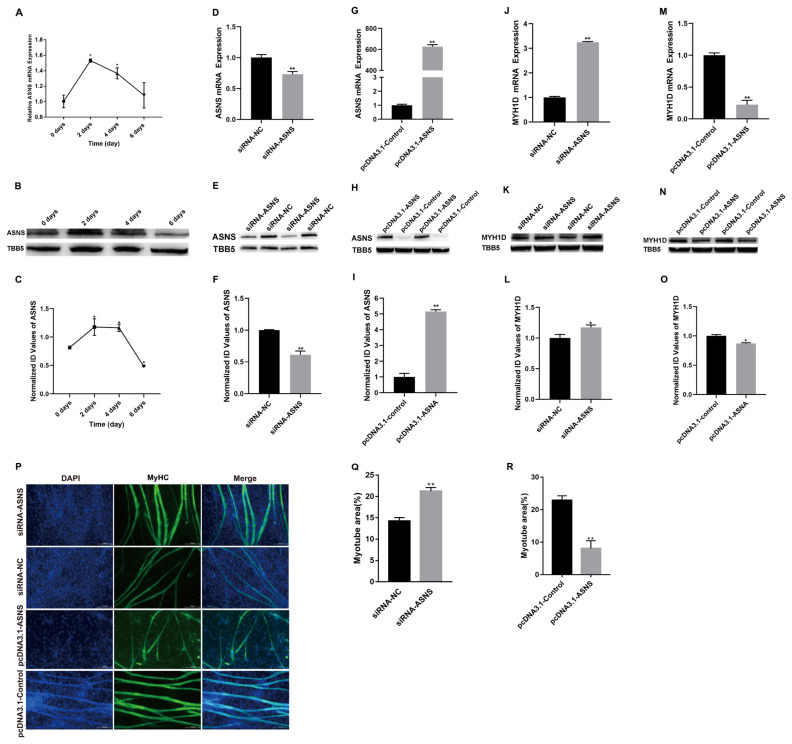
*ASNS* represses chicken skeletal muscle satellite cell differentiation. (A) *ASNS* mRNA level at 0, 2, 4, and 6 days of differentiation in SMSCs. (B) ASNS protein level at 0, 2, 4, and 6 days of differentiation in SMSCs. (C) The densitometry value of ASNS protein level at 0, 2, 4, and 6 days of differentiation in SMSCs. (D) ASNS mRNA level at day 3 of DM after transfection siRNA-*ASNS* or NC. (E) ASNS protein level after transfection siRNA*-ASNS* or NC. (F) The densitometry value of ASNS protein level after transfection siRNA*-ASNS* or NC. (G) *ASNS* mRNA level at day 3 of DM after transfection pcDNA3.1-*ASNS* or pcDNA3.1-Control. (H) ASNS protein level at day 3 of DM after transfection pcDNA3.1-*ASNS* or pcDNA3.1-Control. (I) The densitometry value of ASNS protein level after transfection pcDNA3.1-*ASNS* or pcDNA3.1-Control. (J) *MYH1D* mRNA level at day 3 of DM after transfection siRNA-*ASNS* or NC. (K) The densitometry value of MYH1D protein level after transfection siRNA-*ASNS* or NC. (L) The densitometry value of MYH1D protein level after transfection siRNA*-ASNS* or NC. (M) *MYH1D* mRNA level at day 3 of DM after transfection pcDNA3.1-*ASNS* or pcDNA3.1-Control. (N) MYH1D protein level at day 3 of DM after transfection pcDNA3.1-*ASNS* or pcDNA3.1-Control. (O) The densitometry value of MYH1D protein level after transfection pcDNA3.1-*ASNS* or pcDNA3.1-Control. (P) MyHC (green) was detected with immunofluorescence after transfection at day 3 of DM. Scale bar = 100 μm. (Q) Myotube area at day 3 of DM after transfection siRNA-*ASNS* or NC. (R) Myotube area at day 3 of DM after transfection pcDNA3.1-*ASNS* or pcDNA3.1-Control (* p<0.05; ** p<0.01). *ASNS*, asparagine synthetase; SMSCs, skeletal muscle satellite cells; DM, differentiation medium; NC, negative control; *MYH1D*, myosin heavy chain 1D; MyHC, myosin heavy chain.

The expression of *ASNS* at different stages of cell differentiation was examined. The results demonstrated that *ASNS* mRNA expression sharply increased from day 0 to day 2 but gradually decreased from day 2 to day 6 (Figure 4A), with a similar trend observed in protein expression (Figure 4B, 4C). To further validate the role of *ASNS* during chicken SMSC differentiation, the *ASNS* was overexpressed or interfered (Figure 4D–4I). *MYH1D* is a marker gene of muscle fibers [13]. *ASNS* interference led to an increase in *MYH1D* expression, while *ASNS* overexpression exerted the opposite effects (Figure 4J–4O). Additionally, knockdown of *ASNS* promoted the formation of myotubes, while *ASNS* overexpression showed the opposite results (Figure 4P–4R). In summary, *ASNS* could inhibit cell differentiation in chicken SMSCs.

### *ASNS* inhibits chicken skeletal muscle satellite cell differentiation by activating the AMPK signaling pathway

**Figure 5:**
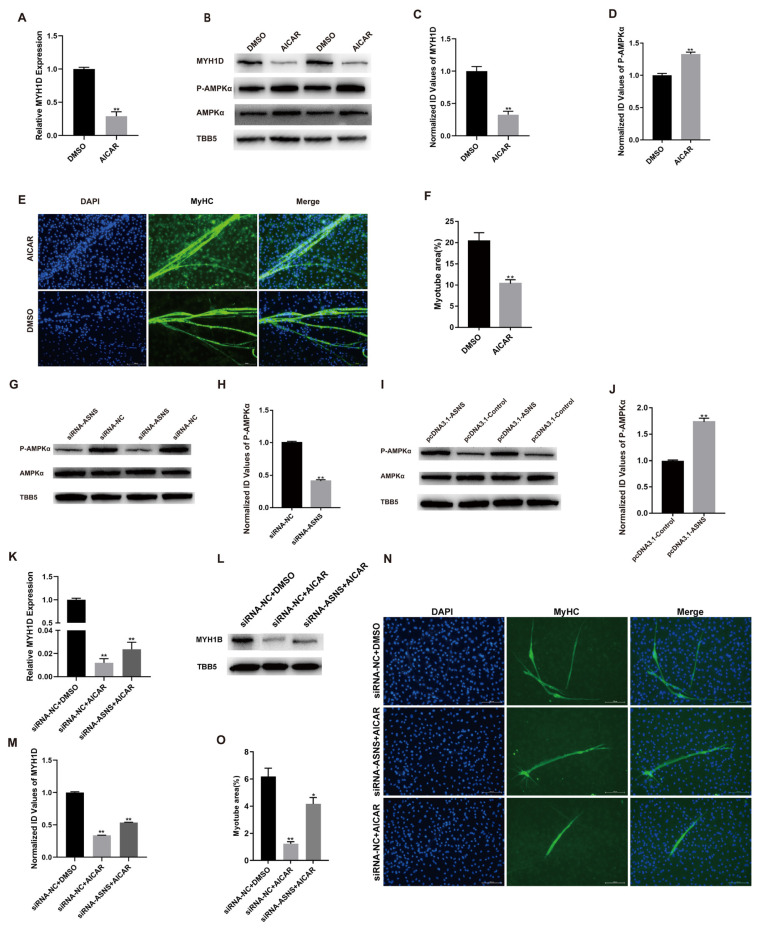
Effect of *ASNS* on AMPK pathway during chicken muscle cell differentiation. (A) *MYH1D* mRNA level at day 3 of DM after treating with AICAR. (B) The protein level of MYH1D, P-AMPKα, and AMPKα at day 3 of DM after treating with AICAR. (C) The densitometry value of MYH1D protein level. (D) pAMPKα and the densitometry value of pAMPKα/AMPKα protein level. (E) MyHC (green) was detected with immunofluorescence after transfection at day 3 of DM. Scale bar = 100 μm. (F) Myotube area at day 3 of DM after treating with AICAR. (G) The protein level of P-AMPKα and AMPKα at day 3 of DM after transfection siRNA-*ASNS* or NC. (H) The densitometry value of P-AMPKα and AMPKα protein level at day 3 of DM after transfection siRNA-*ASNS* or NC. (I) The protein level of P-AMPKα and AMPKα at day 3 of DM after transfection pcDNA3.1-*ASNS* or pcDNA3.1-Control. (J) The densitometry value of P-AMPKα and AMPKα protein level at day 3 of DM after transfection pcDNA3.1-*ASNS* or pcDNA3.1-Control. (K) The mRNA level of *MYH1D*. (L) The protein level of MYH1D. (M) The densitometry value of MYH1D protein level. (N) MyHC (green) was detected with immunofluorescence after transfection at day 3 of DM. Scale bar = 100 μm. (O) Myotube area at day 3 of DM after transfection (* p<0.05; ** p<0.01). *ASNS*, asparagine synthetase; AMPK, adenosine 5′-monophosphate-activated protein kinase; *MYH1D*, myosin heavy chain 1D; DM differentiation medium; AICAR, 5-Aminoimidazole-4-carboxamide ribonucleotide; P-AMPKα, phospho-AMP-activated protein kinase alpha; MyHC, myosin heavy chain; NC, negative control.

AICAR is a widely used AMPK activator [14]. AICAR treatment reduced *MYH1D* expression (Figure 5A–5C). Western blot results demonstrated that AICAR facilitated the phosphorylation of AMPK pathway within the cells (Figures 5B, 5D). Additionally, immunofluorescence analysis revealed that AICAR inhibited the formation of myotubes (Figures 5E, 5F). Therefore, AICAR suppresses the differentiation of chicken SMSCs.

The levels of phosphorylated AMPK protein in *ASNS*-knockdown cells significantly decreased but the total AMPK protein level unchanged (Figure 5G, 5H). Conversely, the levels of phosphorylated AMPK protein significantly increased in cells overexpressing *ASNS*, while the total AMPK protein level remained unchanged (Figure 5I, 5J). Therefore, we speculated that ASNS might regulate the muscle satellite cell differentiation through the AMPK signaling pathway. Next, the cells were treated with siRNA-*ASNS* in a DM containing AICAR. The results indicated that AICAR treatment suppressed the *MYH1D* expression induced by siRNA-*ASNS* (Figures 5K–5M). Moreover, immunofluorescence analysis demonstrated that AICAR treatment also inhibited muscle fiber formation induced by siRNA-*ASNS* (Figures 5N, 5O). Therefore, *ASNS* regulates chicken muscle satellite cell differentiation through the AMPK signaling pathway.

### Identification of differentially expressed genes related to *ASNS* regulation

**Figure 6:**
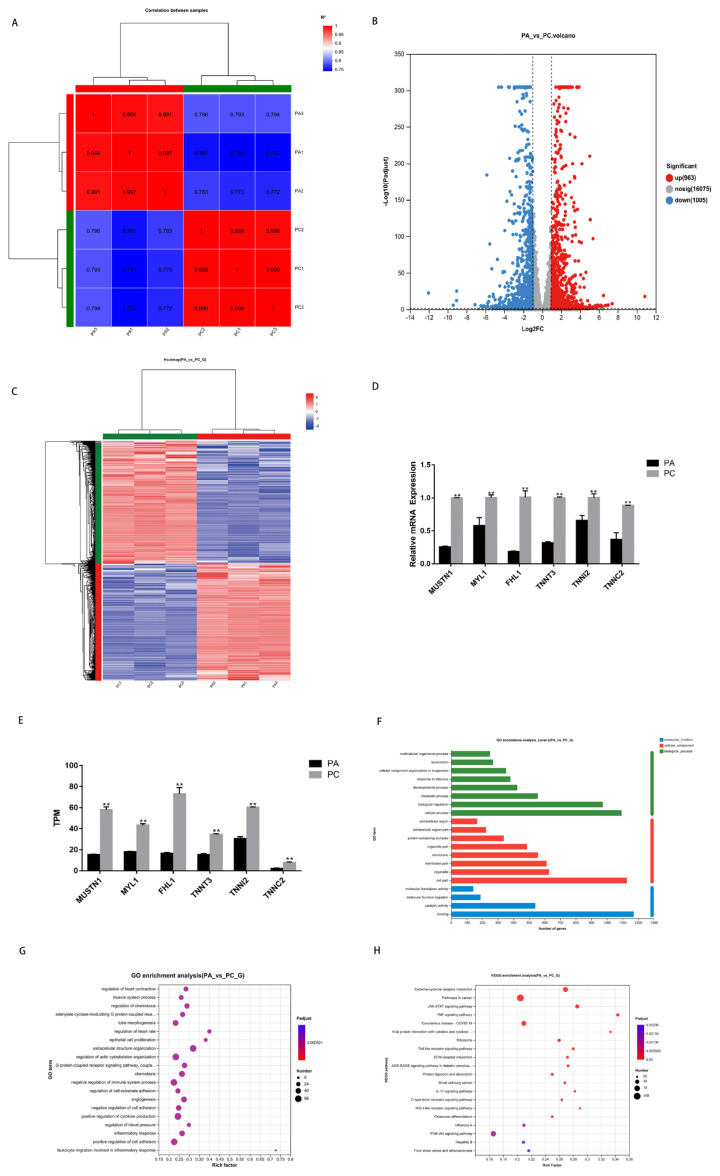
Identification of differentially expressed genes related to ASNS regulation. (A) Heat map of inter-sample correlation. (B) Volcano plot for differential gene expression. (C) Heat map of differential gene expression (D) Validation of DEGs by qRT-PCR. (E) DEGs in RNA-seq results. (F) DEGs were annotated with 20 GO terms. (G) the top 20% significantly enriched Go terms. (H) Top 20 significantly enriched KEGG pathways (* p<0.05; ** p<0.01). *ASNS*, asparagine synthetase; qRT-PCR, real-time quantitative reverse transcription polymerase chain reaction; DEGs, differentially expressed genes; GO, gene ontology; KEGG, Kyoto encyclopedia of genes and genomes.

To identify the important genes and pathways that influenced chicken muscle satellite cell differentiation by ASNS, RNA-Seq was performed. During the sequencing process, raw data underwent quality control and assessment to ensure the accuracy of subsequent analyses. The results presented in Table 3 revealed that Q20 scores were consistently above 98%, Q30 scores were consistently above 94%, and the average sequencing base error rate was below 0.1%. The GC content varied between 49% and 51%. These outcomes demonstrated a low error probability in the data, meeting the sequencing requirements. More than 93% of reads could be mapped to the reference genome, with over 91% of clean reads having a unique alignment position on the reference genome (Table 4). This indicated that the obtained clean reads were suitable for post-sequencing analysis. Correlation analysis was conducted to assess the quality of within-group replicability. The results indicated variations among the groups, while exhibiting good biological reproducibility within each group (Figure 6A).

To visualize gene expression distribution, a volcano plot was created with −log10 (P adjust) on the y-axis and log2 (FC) on the x-axis (Figure 6B). A total of 1,968 DEGs were found. In the comparison between the PA and PC groups, 963 genes were upregulated, and 1,005 genes were downregulated (Figure 6B; Supplementary Dataset S1). For accuracy, hierarchical clustering was conducted on selecting DEGs. The x-axis signifies sample clustering, with each column representing a sample. Samples were clustered based on gene expression pattern similarity. Closer distances implied higher sample similarity (Figure 6C). Six DEGs were randomly chosen and validated using real-time qPCR, showing strong agreement in expression trends with sequencing data, affirming the reliability of microarray analysis (Figures 6D, 6E). These findings also confirmed DEGs efficiently differentiate SMSCs samples into PA and PC groups.

To gain a deeper understanding into the molecular characterization of these DEGs, a GO annotation analysis was performed. The results revealed that 1968 DEGs were annotated with 20 GO terms, including 8 BP, 8 CCs, and 4 MFs. The primary enriched GO terms were observed in BP, including multicellular organismal processes, localization, cellular component organization or biogenesis, response to stimulus, developmental processes, metabolic processes, biological regulation, and cellular processes (Figure 6F). Additionally, enrichment was observed in CC terms, including extracellular region, extracellular region part, protein-containing complex, organelle part, membrane, membrane part, organelle, and cell part. The main functional categories in MF were molecular transducer activity, MF regulator, catalytic activity, and binding (Figure 6F). Otherwise, the top 20% significantly enriched Go terms (P adjust ≤0.05) were shown in Figure 6G, including the muscle system process.

To know gene distribution-related signaling pathways, KEGG pathway analysis was performed. A total of 1,477 DEGs were mapped onto 332 KEGG pathways (Supplementary Dataset S2), with 44 pathways showing significant enrichment (p<0.05). Several significantly enriched signaling pathways were identified, including the JAK-STAT signaling pathway, tumor necrosis factor (TNF) signaling pathway, toll-like receptor signaling pathway and PI3K-Akt signaling pathway (Figure 6H).

## DISCUSSION

*ASNS* is implicated in chicken muscle development and the formation of wooden breast syndrome in broiler chickens [15]. However, there has been little investigation of its function in skeletal muscle development, especially in broiler chickens. In this study, the Xiaoshan chicken exhibited faster average daily and muscle weight gain. Meanwhile, ASNS showed a high expression in Xiaoshan chicken thigh muscles. In cases where age and rearing conditions are the same, genetic inheritance is the primary factor influencing muscle growth and development [16]. *ASNS* may play a crucial role in regulating muscle development in fast-growing chicken breeds. The expression trend of *ASNS* was also opposite to the muscle fiber-specific gene *MYH1D* in the chicken muscles. When comparing woody breast myopathy in chicken breast meat with normal muscle tissue, *MYH1D* exhibited an opposite expression pattern to *ASNS* [15], thus further indicating the potential role of *ASNS* in muscle development.

The knockdown of *ASNS* suppressed the proliferation of chicken SMSCs, arresting the cells in the G0/G1 phase. The cell cycle is a fundamental process governing cell proliferation, primarily regulated by cyclins, cyclin-dependent kinase inhibitor and cyclin-dependent kinases (*CDKs*). Meanwhile, inhibition of *cyclin D1* and *cyclin E* represses cell proliferation by arresting cells in the G0/G1 phase [17]. After *ASNS* knockout, the cyclin‐dependent kinase (*CDK4*), cyclin‐dependent kinase (*CDK6*), and *cyclin D1* significantly downregulated in melanoma cells, which decreased cell proliferation [18]. In breast cancer, downregulation of the ASNS protein also induced cell cycle arrest and inhibited cell growth [19]. In the present study, *ASNS* inhibition or overexpression respectively downregulated or upregulated *cyclin D1* and *cyclin E* in chicken SMSCs. Therefore, *ASNS* might affect chicken SMSC proliferation by regulating the expression of cell cycle-related genes.

The expression of *ASNS* increased initially and then decreased during chicken muscle satellite cell differentiation. The trend was also observed in C2C12 muscle cells [20]. Between day 0 and day 2 of differentiation, most cells may still be in a proliferative state, and the increased expression of ASNS might facilitate the transition from proliferation to differentiation. Between day 2 and day 6 of differentiation, the cells have fully entered the differentiation state, and the downregulation of ASNS expression may be beneficial for cell differentiation. There are few studies on the regulation of the AMPK signaling pathway by *ASNS* in muscle cells. We discovered that *ASNS* promoted the level of AMPK phosphorylation, inhibiting muscle fiber differentiation. Prior studies show that phosphorylation of AMPK reduces the protein expression of MyoD, decreasing the formation of muscle tubes [21]. Glutamate treatment induces AMPK phosphorylation in rat myotubes, subsequently enhancing glucose uptake [22]. Hence, the activation of the AMPK signaling pathway in chicken muscle satellite cells may be associated with the glutamate production induced by *ASNS*. Further investigation is needed to elucidate the specific mechanisms of *ASNS* regulation of the AMPK signaling pathway and their impact on muscle fiber differentiation.

Through RNA-Seq technology, the key genes and signaling pathways affected by *ASNS* during chicken SMSC differentiation were screened. GO annotation analysis revealed that 424 genes were significantly enriched in the GO term: developmental process. Among these DEGs, musculoskeletal embryonic nuclear protein 1 (*MUSTN1*) was downregulated in the overexpressed group. In chicken SMSCs, knocking down *MUSTN1* downregulates the muscle cell differentiation related genes [23]. *ASNS* may influence chicken SMSC differentiation by inhibiting *MUSTN1*. The *MYH1D* and *MYL1*, which are muscle fiber markers [24], were significantly downregulated in the overexpressed group. This indicates the overexpression of *ASNS* indeed has an inhibitory effect on the differentiation of muscle fibers in chicken muscles. The *TNNT3*, *TNNI2*, and *TNNC2*, were identified as downregulated DEGs in the overexpressed group. Those genes may potentially maintain lower intracellular calcium ion levels and prevent the formation of slow muscle fibers [25]. Therefore, we speculate that *ASNS* may regulate these genes by participating in the regulation of calcium ion concentration, which in turn affects the composition of muscle fiber types. However, the specific mechanisms still require further investigation.

The pyruvate dehydrogenase kinase 4 (*PDK4*) was downregulated in the PA group. *PDK4* inhibition regulates AMPK activation in skeletal muscle [26]. NUAK family kinase 2 (*NUAK2*, also known as *SNARK*) were upregulated in the PA group. *NUAK2* can activate the AMPK signaling pathway in muscle cells [27]. Vascular endothelial growth factor A (*VEGFA*) was also identified as upregulated DEGs. AMPK has been reported to stimulate *VEGFA* gene expression [28]. Thus, we inferred that these DEGs may mediate the regulation of the AMPK signaling pathway by *ASNS* or downstream during myoblast differentiation.

KEGG enrichment analysis showed that some of the DEGs were significantly enriched in the pathways including the JAK-STAT signaling pathway, TNF signaling pathway, toll-like receptor signaling pathway and PI3K-Akt signaling pathway. The above-mentioned signaling pathways are all found to be related to muscle development [29]. *FHL1* was downregulated in the overexpression group and significantly enriched in the JAK-STAT signaling pathway. *FHL1* promotes the differentiation of chicken myoblasts [30]. Matrix metallopeptidase 9 (*MMP9*), significantly enriched in the TNF signaling pathway, was upregulated in the overexpression group. *MMP9* can degrade the muscle matrix and inhibit muscle cell differentiation when skeletal muscle is injured [31]. Forkhead box O 6 (*FOXO6*) was significantly enriched in the PI3K-Akt signaling pathway and downregulated in the overexpression group. Knocking down of *FOXO6* induces C2C12 myotube atrophy and significantly downregulates the expression of myogenic determination factors [32]. Therefore, *ASNS* may also influence the differentiation of chicken SMSCs through these pathways.

Taken together, our present study demonstrated that ASNS significantly promotes myogenic proliferation and inhibits the formation of muscle myotubes through AMPK phosphorylation in chicken SMSCs. In addition, RNA-seq analysis screened the crucial genes and signaling pathways regulated by *ASNS* during the differentiation process of chicken SMSCs. Altogether, our study provides a clear direction for further exploring the role of *ASNS* in muscle development.

## Figures and Tables

**Table 1:** Sequence of RNA oligonucleotides

Name	Sequence (5′-3′)
siRNA-*ASNS*	GCAGGAGCAGUUUGGAUUUTT
	AAAUCCAAACUGCUCCUGCTT
Negative control	UUCUCCGAACGUGUCACGUTT
	ACGUGACACGUUCGGAGAATT

*ASNS*, asparagine synthetase.

**Table 2:** Primers used in the real-time polymerase chain reaction

Gene	Sequence (5′-3′)	Product size (bp)
*GAPDH*	F: CGATCTGAACTACATGGTTTAC	153
	R: TCTGCCCATTTGATGTTGC	
*ASNS*	F: AGTTGCAGGAGCAGTTTGGA	235
	R: GCCTCAGAACAGACACCCAA	
*MYH1D*	F: CCACCAATCCATACGACT	216
	R: CTGGCTCTGCTTGCTCT	
*cyclin D1*	F: CACTTGGATGCTGGAGGT	110
	R: GGCTTTTCTTGAGGGGTT	
*cyclin E*	F: AAAAGCAATACGAAAACC	302
	R: AAAAGCAATACGAAAACC	
*CDKN2A*	F: GGCCTCTGTCCTTCTCGCT	100
	R: CTCAGAACCCGGCGCAGAAT	
*FHL1*	F:AAATGCACAAAGTGTGCCCG	87
	R:TCGTTTGGGACACTCAGCAC	
*MUSTN1*	F:AGCAGTGTGAACAAATGGGCTCTG	104
	R:GCTTTTGGGCGGCTCATCTTTTG	
*TNNT3*	F:TCCTCCATGGGTGCCTCATA	163
	R:CCTTAGCCTTGTCCCTCAGC	
*TNNI2*	F:CAAGCACAAGGTCAACATGGA	63
	R:TCCGTGTCCTCCTTCTTGACTT	
*TNNC2*	F:TGCGCCAGATGAAAGAGGAC	96
	R:TGTCGATGAACCCATCAGCG	
*MYL1*	F:ATGCCAAGATTACCCTGAGCC	70
	R:TCAGCGTTTGTGGGGTTCTG	
*IGF2*	F:AGACCAGTGGGACGAAATAACA	121
	R:CACGCTCTGACTTGACGGAC	

**Table 3:** Quality assessment of sequencing data

Sample^1)^	Q20 (%)	Q30 (%)	GC content (%)	Error rate (%)
PA1	98.03	94.17	49.91	0.025
PA2	98.15	94.44	49.58	0.0248
PA3	98.18	94.59	50.49	0.0247
PC1	98.06	94.24	50.05	0.0249
PC2	98.24	94.65	49.41	0.0245
PC3	98.24	94.72	50.02	0.0245

GC, guanine and cytosine.

1) PA1-3, experimental group; PC1-3, control group.

**Table 4:** Statistical results of the comparison with the reference genome

Sample^1)^	Total reads	Total mapped (%)	Multiple mapped (%)	Uniquely mapped (%)
PA1	61,415,614	57,158,828 (93.07)	1,139,330 (1.86)	56,019,498 (91.2^1)^
PA2	45,294,996	42,228,109 (93.23)	788,359 (1.74)	41,439,750 (91.49)
PA3	52,668,958	49,161,483 (93.34)	991,788 (1.88)	48,169,695 (91.46)
PC1	50,815,090	47,519,806 (93.52)	1,050,670 (2.07)	46,469,136 (91.45)
PC2	57,705,276	54,008,076 (93.59)	1,179,599 (2.04)	52,828,477 (91.55)
PC3	53,777,496	50,242,902 (93.43)	1,066,838 (1.98)	49,176,064 (91.44)

1) PA1-3, experimental group; PC1-3, control group.

## Data Availability

All data obtained or analyzed in this study are available from the corresponding author upon request. The sequencing raw data is uploaded to the SRA database (Access number: PRJNA 1022608, PRJNA1022609, PRJNA1022772, PRJNA1022773, PRJNA1022794, and PRJNA1022796).

## SUPPLEMENTARY MATERIAL

**Supplementary Dataset S1**. DEGs between the PA and PC group

**Supplementary Dataset S2**. DEGs which mapped onto KEGG pathways
