# Supplementary material for: Asparagine synthetase regulates the proliferation and differentiation of chicken skeletal muscle satellite cells
Source: Anim Biosci. 2024 Aug 26;37(11):1848–62. doi: 10.5713/ab.24.0271 (PMC11541025; doi:10.5713/ab.24.0271)
Supplement: Supplementary file 1 [file ab-24-0271-Supplementary-Dataset-1.pdf]

Supplementary Dataset S1. DEGs between the PA and PC group

Gene_id	Gene_name	Gene_descr	PA_vs_PC	Sum
1968			1968	1968
ENSGALG00C	ZDHHC4	zinc finge	yes|up	1
ENSGALG00000043909		microsemir	yes|up	1
ENSGALG00000049385			yes|down	1
ENSGALG00C	ALDH18A1	aldehyde c	yes|down	1
ENSGALG00C	DTNA	dystrobrev	yes|down	1
ENSGALG00C	GATA2	GATA bindi	yes|up	1
ENSGALG00C	ALOX5AP	arachidona	yes|up	1
ENSGALG00000049343		uncharacte	yes|up	1
ENSGALG00000051630			yes|up	1
ENSGALG00C	CCND3	cyclin D3	yes|up	1
ENSGALG00C	SLC9A9	solute car	yes|down	1
ENSGALG00C	gga-mir-66	gga-mir-66	yes|down	1
ENSGALG00000052960			yes|up	1
ENSGALG00000045478		exocyst cc	yes|up	1
ENSGALG00C	MYH15	myosin, he	yes|down	1
ENSGALG00000047616			yes|up	1
ENSGALG00C	CCL19	C-C motif	yes|up	1
ENSGALG00C	RNF144A	ring finge	yes|down	1
ENSGALG00C	P2RX7	purinergic	yes|up	1
ENSGALG00000031149		CD55 molec	yes|up	1
ENSGALG00C	IRF7	interferon	yes|up	1
ENSGALG00C	CHAC1	ChaC gluta	yes|down	1
ENSGALG00C	P2RY4	pyrimidine	yes|up	1
ENSGALG00C	ECM2	extracellu	yes|down	1
ENSGALG00000029381			yes|up	1
ENSGALG00C	CCL20	C-C motif	yes|up	1
ENSGALG00000048286			yes|up	1
ENSGALG00C	TMEM254	transmembr	yes|down	1
ENSGALG00C	NTRK3	neurotroph	yes|down	1
ENSGALG00C	AHR2	aryl hydrc	yes|up	1
ENSGALG00000030259		uncharacte	yes|down	1
ENSGALG00C	BPHL	biphenyl h	yes|down	1
ENSGALG00C	DUSP15	dual speci	yes|up	1
ENSGALG00000001604		galactosic	yes|down	1
ENSGALG00C	MATN2	matrilin 2	yes|down	1
ENSGALG00C	SBK1	SH3 domair	yes|down	1
ENSGALG00000049530			yes|down	1
ENSGALG00C	IFI6	interferon	yes|up	1
ENSGALG00000049127			yes|down	1
ENSGALG00000045684		plasmalemm	yes|up	1
ENSGALG00C	ARRDC1	arrestin c	yes|up	1
ENSGALG00C	ADAMTS1	ADAM metal	yes|down	1
ENSGALG00C	MAP1B	microtubul	yes|down	1
ENSGALG00C	LCAT	lecithin-c	yes|down	1
ENSGALG00000046819			yes|down	1
ENSGALG00C	ADORA2B	adenosine	yes|up	1
ENSGALG00000052514		early endc	yes|up	1
ENSGALG00C	BDKRB1	bradykinir	yes|up	1
ENSGALG00C	gga-mir-65	gga-mir-65	yes|down	1

ENSGALG00000051641		yes|down		1
ENSGALG00C GPSM1	G-protein	yes|down		1
ENSGALG00C SCG2	secretogra	yes|up		1
ENSGALG00C CDC6	cell divis	yes|up		1
ENSGALG00C CCDC85C	coiled-coi	yes|up		1
ENSGALG00000048674		yes|down		1
ENSGALG00000000104	cryptochrc	yes|down		1
ENSGALG00C FREM2	FRAS1 rela	yes|down		1
ENSGALG00C TSPAN10	tetraspani	yes|down		1
ENSGALG00C EDN2	endothelir	yes|up		1
ENSGALG00000051085		yes|up		1
ENSGALG00C CAPN9	calpain 9	yes|up		1
ENSGALG00C MFSD8	major faci	yes|up		1
ENSGALG00C ALDH6A1	aldehyde c	yes|down		1
ENSGALG00000051882	mucin-4-li	yes|up		1
ENSGALG00000051887		yes|up		1
ENSGALG00000051929		yes|down		1
ENSGALG00C HIST1H2B8	histone cl	yes|down		1
ENSGALG00C SFXN2	sideroflex	yes|down		1
ENSGALG00C OASL	2'-5'-olig	yes|up		1
ENSGALG00000010218		yes|up		1
ENSGALG00C LAPTM4B	lysosomal	yes|up		1
ENSGALG00000019325	uncharacte	yes|up		1
ENSGALG00000048671	E3 ubiquit	yes|up		1
ENSGALG00C PAK1	p21 (RAC1)	yes|up		1
ENSGALG00C NKX3-2	NK3 homeob	yes|down		1
ENSGALG00C NT5M	5',3'-nucl	yes|down		1
ENSGALG00C SLC25A48	solute car	yes|down		1
ENSGALG00000000720	guanylate	yes|up		1
ENSGALG00C SLC41A1	solute car	yes|up		1
ENSGALG00000005884	mitogen-ac	yes|down		1
ENSGALG00000047000		yes|up		1
ENSGALG00C NRCAM	neuronal c	yes|down		1
ENSGALG00C ANGPTL4	angiopoiet	yes|up		1
ENSGALG00C MET	MET proto-	yes|up		1
ENSGALG00C DLL1	delta like	yes|up		1
ENSGALG00C SLC25A47	solute car	yes|up		1
ENSGALG00C CEBPD	CCAAT/enha	yes|up		1
ENSGALG00C ADCYAP1R1	ADCYAP rec	yes|down		1
ENSGALG00C ELF3	E74 like E	yes|up		1
ENSGALG00C TRIM36	tripartite	yes|down		1
ENSGALG00C TFCP2L1	transcript	yes|down		1
ENSGALG00C TNFAIP3	TNF alpha	yes|up		1
ENSGALG00C CC2D1A	coiled-coi	yes|up		1
ENSGALG00C CYTH4	cytohesin	yes|up		1
ENSGALG00000037070		yes|down		1
ENSGALG00C RAB26	RAB26, mem	yes|down		1
ENSGALG00C RBP7	retinol bi	yes|down		1
ENSGALG00C IL20RA	interleuki	yes|up		1
ENSGALG00C KDR	kinase ins	yes|up		1
ENSGALG00C INSC	inscuteabl	yes|up		1

ENSGALG00C P2RY1	purinergic	yes|up	1
ENSGALG00C MAP7	microtubul	yes|up	1
ENSGALG00000031929	potassium	yes|up	1
ENSGALG00C C3orf33	chromosome	yes|down	1
ENSGALG00C GABRG3	gamma-amir	yes|up	1
ENSGALG00C ADAMTS4	ADAM metal	yes|up	1
ENSGALG00C PCDH18	protocadhe	yes|down	1
ENSGALG00C GNAO1	G protein	yes|down	1
ENSGALG00000032384		yes|up	1
ENSGALG00000000433	tubulin al	yes|up	1
ENSGALG00000048523		yes|up	1
ENSGALG00000005011	SHC adaptc	yes|up	1
ENSGALG00C XYLT1	xylosyltra	yes|down	1
ENSGALG00000006756	lipopolysa	yes|up	1
ENSGALG00C NEGR1	neuronal g	yes|down	1
ENSGALG00C APOD	apolipoprc	yes|down	1
ENSGALG00C SPP1	secreted p	yes|up	1
ENSGALG00C FAT4	FAT atypic	yes|down	1
ENSGALG00C MMP9	matrix met	yes|up	1
ENSGALG00C PPP1R12B	protein ph	yes|down	1
ENSGALG00000050458		yes|up	1
ENSGALG00C FAM83G	family wit	yes|up	1
ENSGALG00C GALNT17	Williams-E	yes|down	1
ENSGALG00C ADRA2A	adrenocept	yes|down	1
ENSGALG00C EEF1B2	eukaryotic	yes|down	1
ENSGALG00000001525	calcium bi	yes|up	1
ENSGALG00000044811	beta-micrc	yes|down	1
ENSGALG00000049652	estradiol	yes|down	1
ENSGALG00000047911		yes|up	1
ENSGALG00C COL1A2	collagen t	yes|down	1
ENSGALG00C EDNRB	endothelir	yes|up	1
ENSGALG00C ICOSLG	inducible	yes|up	1
ENSGALG00C NFKB2	nuclear fa	yes|up	1
ENSGALG00C ACOD1	aconitate	yes|up	1
ENSGALG00C PTGS1	prostaglar	yes|down	1
ENSGALG00C PLXNA4	plexin A4	yes|down	1
ENSGALG00C AOX2	aldehyde c	yes|down	1
ENSGALG00C NLRC5	NLR family	yes|up	1
ENSGALG00C CAMK1G	calcium/ca	yes|up	1
ENSGALG00C TNNT3	troponin T	yes|down	1
ENSGALG00000054601		yes|up	1
ENSGALG00C PAX3	paired box	yes|down	1
ENSGALG00000054607		yes|down	1
ENSGALG00C MOV10	Mov10 RISC	yes|up	1
ENSGALG00C SLC52A3	solute car	yes|up	1
ENSGALG00C IFIH1	interferor	yes|up	1
ENSGALG00C NXPH4	neurexophi	yes|up	1
ENSGALG00C FHL1	four and a	yes|down	1
ENSGALG00C SLCO3A1	solute car	yes|up	1
ENSGALG00C FRY	FRY microt	yes|up	1
ENSGALG00C IL8	interleuki	yes|up	1

ENSGALG00C C12orf75	chromosome	yes|down	1
ENSGALG00C PLXDC1	plexin dom	yes|down	1
ENSGALG00C KPNA7	karyopheri	yes|up	1
ENSGALG00C ROPN1L	rhophilin	yes|down	1
ENSGALG00C DAP	death assc	yes|down	1
ENSGALG00C PALM	paralemmir	yes|up	1
ENSGALG00000027571	histone cl	yes|down	1
ENSGALG00C PTGFR	prostaglar	yes|up	1
ENSGALG00C RPL13	ribosomal	yes|down	1
ENSGALG00C CRISPLD2	cysteine r	yes|down	1
ENSGALG00000036763	cadherin E	yes|down	1
ENSGALG00C ZC3H12A	zinc finge	yes|up	1
ENSGALG00000038920		yes|down	1
ENSGALG00C ANGPTL5	angiopoiet	yes|down	1
ENSGALG00C CNTN5	contactin	yes|up	1
ENSGALG00C CDH2	cadherin 2	yes|up	1
ENSGALG00C ARHGEF10	Rho guanir	yes|down	1
ENSGALG00000007710	ras-like p	yes|down	1
ENSGALG00C RPL4	ribosomal	yes|down	1
ENSGALG00000049820		yes|up	1
ENSGALG00C BCHE	butyrylchc	yes|down	1
ENSGALG00C ST8SIA4	ST8 alpha-	yes|up	1
ENSGALG00C SNCAIP	synuclein	yes|down	1
ENSGALG00C UPP1	uridine ph	yes|up	1
ENSGALG00C PLCD4	phospholip	yes|down	1
ENSGALG00C GXYLT2	glucoside	yes|down	1
ENSGALG00C RPS11	ribosomal	yes|down	1
ENSGALG00C BCAT1	branched c	yes|down	1
ENSGALG00C PIK3R5	phosphoinc	yes|up	1
ENSGALG00C LONRF3	LON peptic	yes|up	1
ENSGALG00C GAS6	growth arr	yes|down	1
ENSGALG00C SLC6A7	solute car	yes|up	1
ENSGALG00000043610	alpha-tect	yes|down	1
ENSGALG00C KIAA1755	KIAA1755 c	yes|up	1
ENSGALG00C GRIN2C	glutamate	yes|up	1
ENSGALG00000028327		yes|up	1
ENSGALG00C ARL8BL	ADP-ribosy	yes|down	1
ENSGALG00C TRPM6	transient	yes|up	1
ENSGALG00C KIF21A	kinesin fa	yes|down	1
ENSGALG00C GJC3	gap juncti	yes|up	1
ENSGALG00C TRAF2	TNF recept	yes|up	1
ENSGALG00C RMI2	RecQ media	yes|down	1
ENSGALG00C SOCS1	suppressor	yes|up	1
ENSGALG00000035453	retinitis	yes|up	1
ENSGALG00C PMAIP1	phorbol-12	yes|up	1
ENSGALG00C ATP6V0D2	ATPase H+	yes|up	1
ENSGALG00000042587	uncharacte	yes|up	1
ENSGALG00C STAT1	signal tra	yes|up	1
ENSGALG00C SLC9A3R1	SLC9A3 reg	yes|up	1
ENSGALG00C STEAP4	STEAP4 met	yes|up	1
ENSGALG00C PMEL	premelanos	yes|down	1

ENSGALG00C SMYD1	SET and MY	yes|down	1
ENSGALG00C IKBKE	inhibitor	yes|up	1
ENSGALG00C FBP1	fructose-b	yes|up	1
ENSGALG00C KCNN1	potassium	yes|down	1
ENSGALG00C SFTPA2	surfactant	yes|down	1
ENSGALG00C ABHD12	abhydrolas	yes|down	1
ENSGALG00C HES4	hes family	yes|up	1
ENSGALG00C LOX	lysyl oxic	yes|down	1
ENSGALG00C CPNE8	copine 8 [	yes|up	1
ENSGALG00C HHEX	hematopoie	yes|up	1
ENSGALG00C SLC39A8	solute car	yes|up	1
ENSGALG00C IGSF1	immunoglob	yes|up	1
ENSGALG00C WNT16	Wnt family	yes|down	1
ENSGALG00C PAX5	paired box	yes|up	1
ENSGALG00C BORCS6	BLOC-1 rel	yes|up	1
ENSGALG00C KRT18	keratin 18	yes|up	1
ENSGALG00C HEY1	hes relate	yes|up	1
ENSGALG00C GPRC5B	G protein-	yes|down	1
ENSGALG00C RPL23	ribosomal	yes|down	1
ENSGALG00C COLGALT2	collagen b	yes|down	1
ENSGALG00C EDIL3	EGF like r	yes|down	1
ENSGALG00C PRKX	protein ki	yes|up	1
ENSGALG00C ZC3HAV1	zinc finge	yes|up	1
ENSGALG00C ANKRD33B	ankyrin re	yes|up	1
ENSGALG00C C1QTNF2	C1q and tu	yes|down	1
ENSGALG00C TUBB3	tubulin, b	yes|down	1
ENSGALG00000048614		yes|down	1
ENSGALG00000047126	histone cl	yes|down	1
ENSGALG00C FGF18	fibroblast	yes|down	1
ENSGALG00C PPL	periplakin	yes|down	1
ENSGALG00C SLC44A1	solute car	yes|down	1
ENSGALG00C NR4A2	nuclear re	yes|up	1
ENSGALG00000052177		yes|up	1
ENSGALG00000047385		yes|down	1
ENSGALG00C NPL	N-acetylne	yes|down	1
ENSGALG00C BMP4	bone morph	yes|down	1
ENSGALG00000051681		yes|down	1
ENSGALG00C ITPR2	inositol 1	yes|down	1
ENSGALG00000040870		yes|down	1
ENSGALG00000044241		yes|up	1
ENSGALG00C NRARP	NOTCH regu	yes|up	1
ENSGALG00C TSPAN32	tetraspani	yes|up	1
ENSGALG00C CIDEC	cell death	yes|down	1
ENSGALG00C MTR	5-methylte	yes|down	1
ENSGALG00C LRRC20	leucine ri	yes|down	1
ENSGALG00C EVA1C	eva-1 homc	yes|up	1
ENSGALG00C KLHL24	kelch like	yes|down	1
ENSGALG00C PSAT1	phosphoser	yes|down	1
ENSGALG00C MAFB	MAF bZIP t	yes|down	1
ENSGALG00C HOXA1	homeobox A	yes|up	1
ENSGALG00C C1QTNF7	C1q and TN	yes|down	1

ENSGALG00C	NTNG2	netrin G2	yes|up	1
ENSGALG00C	MYF5	myogenic f	yes|up	1
ENSGALG00C	EPHA7	EPH recept	yes|up	1
ENSGALG00C	TBC1D12	TBC1 domai	yes|down	1
ENSGALG00C	LPCAT2	lysophosph	yes|up	1
ENSGALG00000051496			yes|down	1
ENSGALG00C	MAT1A	methionine	yes|down	1
ENSGALG00000047695			yes|down	1
ENSGALG00C	RASGRF2	Ras protei	yes|down	1
ENSGALG00C	SFRP1	secreted f	yes|down	1
ENSGALG00000005938		chromosome	yes|down	1
ENSGALG00C	ID1	inhibitor	yes|up	1
ENSGALG00C	SGCD	sarcoglyca	yes|down	1
ENSGALG00C	AGTR2	angiotensi	yes|down	1
ENSGALG00000042022		GSG1 like	yes|down	1
ENSGALG00C	FAM184A	family wit	yes|down	1
ENSGALG00000038671		uncharacte	yes|up	1
ENSGALG00C	TUBA3E	tubulin, a	yes|down	1
ENSGALG00000047577			yes|down	1
ENSGALG00000049235			yes|up	1
ENSGALG00C	DYNC1I1	dynein cyt	yes|down	1
ENSGALG00000047573			yes|up	1
ENSGALG00C	B3GNT4	UDP-GlcNAc	yes|up	1
ENSGALG00C	UNC5A	unc-5 netr	yes|down	1
ENSGALG00C	CAPN5	calpain 5	yes|down	1
ENSGALG00C	HIST1H110	histone cl	yes|down	1
ENSGALG00C	OAT	ornithine	yes|down	1
ENSGALG00C	SERPINF1	serpin fam	yes|down	1
ENSGALG00C	MFSD2A	major faci	yes|up	1
ENSGALG00C	AIFM3	apoptosis	yes|down	1
ENSGALG00C	SLC9A7	solute car	yes|down	1
ENSGALG00C	FMO3	flavin con	yes|down	1
ENSGALG00C	TMEM200B	transmembr	yes|down	1
ENSGALG00000054853			yes|up	1
ENSGALG00000034397		ectonucleo	yes|down	1
ENSGALG00C	HENMT1	HEN1 methy	yes|down	1
ENSGALG00C	ASPN	biglycan [	yes|up	1
ENSGALG00000051251		histone cl	yes|down	1
ENSGALG00C	SMAD7B	TGF-beta s	yes|up	1
ENSGALG00C	CPNE4	copine 4 [	yes|down	1
ENSGALG00C	RCSD1	RCSD domai	yes|down	1
ENSGALG00000030543			yes|up	1
ENSGALG00000051786			yes|up	1
ENSGALG00C	SEMA4C	semaphorin	yes|up	1
ENSGALG00C	SEMA4B	semaphorin	yes|up	1
ENSGALG00C	CLDN1	claudin 1	yes|up	1
ENSGALG00C	HTR2B	5-hydroxyt	yes|up	1
ENSGALG00C	SPHK1	sphingosin	yes|up	1
ENSGALG00000012836			yes|up	1
ENSGALG00C	SLC2A12	solute car	yes|down	1
ENSGALG00000049481			yes|up	1

ENSGALG00C EEF1D	eukaryotic	yes|down	1
ENSGALG00C LRIF1	ligand dep	yes|up	1
ENSGALG00C C10H15ORF5	chromosome	yes|down	1
ENSGALG00C IL1B	interleuki	yes|up	1
ENSGALG00000052853		yes|down	1
ENSGALG00C MEF2C	myocyte en	yes|down	1
ENSGALG00C GPX3	glutathion	yes|down	1
ENSGALG00C CYP3A4	cytochrome	yes|down	1
ENSGALG00000051325	histone cl	yes|down	1
ENSGALG00000004127	c-X-C chem	yes|up	1
ENSGALG00C CASP18	initiator	yes|up	1
ENSGALG00C ATG13	autophagy	yes|down	1
ENSGALG00C RELL2	RELT like	yes|down	1
ENSGALG00C MAPK10	mitogen-ac	yes|down	1
ENSGALG00C SLCO4C1	solute car	yes|up	1
ENSGALG00C CYP27C1	cytochrome	yes|up	1
ENSGALG00C GCG	glucagon [	yes|up	1
ENSGALG00C TNIP3	TNFAIP3 in	yes|up	1
ENSGALG00000008518	glutamine	yes|up	1
ENSGALG00000019233	phospholip	yes|down	1
ENSGALG00000026422	poly(ADP-r	yes|up	1
ENSGALG00000032428	chromosome	yes|up	1
ENSGALG00000049148		yes|down	1
ENSGALG00000008040	putative a	yes|down	1
ENSGALG00C INAFM2	InaF motif	yes|up	1
ENSGALG00C ST6GALNAC3	ST6 N-acet	yes|down	1
ENSGALG00C SLC39A13	solute car	yes|up	1
ENSGALG00C CLBA1	chromosome	yes|up	1
ENSGALG00C SPI1	Spi-1 prot	yes|up	1
ENSGALG00C NSMF	NMDA recep	yes|down	1
ENSGALG00C GABRA5	gamma-amin	yes|up	1
ENSGALG00C ARHGAP18	Rho GTPase	yes|down	1
ENSGALG00C TSPAN12	tetraspani	yes|down	1
ENSGALG00C ANOS1	anosmin 1	yes|down	1
ENSGALG00C KIF26A	kinesin fa	yes|down	1
ENSGALG00000050699		yes|up	1
ENSGALG00000049090		yes|down	1
ENSGALG00C ADGRA2	adhesion G	yes|down	1
ENSGALG00000051744		yes|up	1
ENSGALG00000020488	chromosome	yes|down	1
ENSGALG00C IL22RA1	interleuki	yes|up	1
ENSGALG00C PLAC8	Placenta-s	yes|up	1
ENSGALG00000051027		yes|up	1
ENSGALG00000000604	Na+/K+ tra	yes|up	1
ENSGALG00C GPR37L1	G protein-	yes|up	1
ENSGALG00C SPRY4	sprouty RT	yes|up	1
ENSGALG00000011747		yes|down	1
ENSGALG00C TNFRSF18	TNF recept	yes|up	1
ENSGALG00C SERPINH1	serpin fam	yes|down	1
ENSGALG00C SMCO3	single-pas	yes|down	1
ENSGALG00000048948		yes|up	1

ENSGALG00C	NAAA	N-acyletha	yes|up	1
ENSGALG00000054033			yes|down	1
ENSGALG00C	TGFB3	transformi	yes|down	1
ENSGALG00C	DOCK4	dedicator	yes|down	1
ENSGALG00C	SCG5	secretogra	yes|down	1
ENSGALG00C	PLPP5	phospholip	yes|down	1
ENSGALG00000037200		MACRO doma	yes|down	1
ENSGALG00C	MSRB1	methionine	yes|down	1
ENSGALG00C	TES	testin LIM	yes|up	1
ENSGALG00C	RANGAP1	Ran GTPase	yes|up	1
ENSGALG00C	COL20A1	collagen t	yes|down	1
ENSGALG00C	FRRS1	ferric che	yes|down	1
ENSGALG00000054048		transmembr	yes|up	1
ENSGALG00000047014		tumor necr	yes|up	1
ENSGALG00C	ARHGEF3	Rho guanin	yes|up	1
ENSGALG00C	TNFAIP2	TNF alpha	yes|up	1
ENSGALG00C	IL6	interleuki	yes|up	1
ENSGALG00C	EXOC3L4	exocyst cc	yes|up	1
ENSGALG00C	RPS16	ribosomal	yes|down	1
ENSGALG00C	MARCKSL1	MARCKS lik	yes|up	1
ENSGALG00C	SLC25A25	solute car	yes|up	1
ENSGALG00C	IP6K3	inositol h	yes|down	1
ENSGALG00000026553		cardiomyop	yes|down	1
ENSGALG00C	ATP8B3	ATPase phc	yes|up	1
ENSGALG00000052372			yes|up	1
ENSGALG00C	HNF4beta	hepatic nu	yes|down	1
ENSGALG00000047825			yes|down	1
ENSGALG00C	SYNRG	synergin g	yes|down	1
ENSGALG00C	TRAFD1	TRAF-type	yes|up	1
ENSGALG00C	GGT7	gamma-glut	yes|down	1
ENSGALG00000028262		prickle pl	yes|up	1
ENSGALG00C	JAKMIP3	Janus kina	yes|down	1
ENSGALG00C	FGF4	fibroblast	yes|up	1
ENSGALG00C	SH3GL3	SH3 domain	yes|down	1
ENSGALG00C	AR	androgen r	yes|down	1
ENSGALG00000054344			yes|down	1
ENSGALG00C	LBH	limb bud a	yes|up	1
ENSGALG00000054594			yes|up	1
ENSGALG00C	HBBR	hemoglobin	yes|down	1
ENSGALG00000054260			yes|up	1
ENSGALG00C	EGLN3	egl-9 fami	yes|up	1
ENSGALG00C	CASTOR1	GATS prote	yes|up	1
ENSGALG00C	OCM2	oncomoduli	yes|down	1
ENSGALG00C	ETV6	ETS varian	yes|up	1
ENSGALG00C	WDR86	WD repeat	yes|down	1
ENSGALG00C	ZDHHC20	zinc finge	yes|up	1
ENSGALG00C	GKN2	gastrokine	yes|up	1
ENSGALG00000027565			yes|up	1
ENSGALG00C	POFUT2	protein O-	yes|down	1
ENSGALG00C	ABCB1	ATP-bindir	yes|up	1
ENSGALG00C	NME2	NME/NM23 r	yes|down	1

ENSGALG00C	UBASH3A	ubiquitin	yes|up	1
ENSGALG00C	TFEC	transcript	yes|up	1
ENSGALG00C	RPSAP58	ribosomal	yes|down	1
ENSGALG00C	B3GNT7	UDP-GlcNAc	yes|up	1
ENSGALG00C	SFXN1	sideroflex	yes|down	1
ENSGALG00000007703		ligand of	yes|up	1
ENSGALG00C	ISL2	ISL LIM hc	yes|up	1
ENSGALG00C	TBX2	T-box 2 [S	yes|up	1
ENSGALG00C	WFIKKN2	WAP, folli	yes|down	1
ENSGALG00C	CLSTN2	calsynteni	yes|down	1
ENSGALG00C	KCNS1	potassium	yes|up	1
ENSGALG00C	AK5	adenylate	yes|up	1
ENSGALG00000019761		chondroadh	yes|down	1
ENSGALG00C	C1orf159	chromosome	yes|up	1
ENSGALG00C	HIVEP3	human immu	yes|up	1
ENSGALG00C	WDR31	WD repeat	yes|up	1
ENSGALG00C	ACSF2	acyl-CoA s	yes|down	1
ENSGALG00C	PERP2	PERP2, TP5	yes|up	1
ENSGALG00000031659			yes|down	1
ENSGALG00000016837		myosin XVI	yes|down	1
ENSGALG00000031714			yes|up	1
ENSGALG00C	PATL2	PAT1 homol	yes|up	1
ENSGALG00C	CDA	cytidine c	yes|down	1
ENSGALG00000031430		selectin E	yes|up	1
ENSGALG00C	CRB1	crumbs cel	yes|down	1
ENSGALG00C	ATRNL1	attractin	yes|down	1
ENSGALG00C	TCIM	chromosome	yes|up	1
ENSGALG00C	CORO2A	coronin 2A	yes|up	1
ENSGALG00000043582		lymphocyte	yes|up	1
ENSGALG00C	FTH1	ferritin h	yes|down	1
ENSGALG00C	DPP4	dipeptidyl	yes|down	1
ENSGALG00C	BNC1	basonuclin	yes|down	1
ENSGALG00C	AOX1	aldehyde c	yes|down	1
ENSGALG00C	GLP2R	glucagon-l	yes|down	1
ENSGALG00C	SGCZ	sarcoglyca	yes|down	1
ENSGALG00C	IL7	interleuki	yes|up	1
ENSGALG00000035447		interleuki	yes|up	1
ENSGALG00000051375			yes|up	1
ENSGALG00C	FBXO15	F-box prot	yes|up	1
ENSGALG00C	TMEM204	transmembr	yes|down	1
ENSGALG00C	C4orf48	chromosome	yes|down	1
ENSGALG00C	DIPK1C	family wit	yes|down	1
ENSGALG00000003595			yes|down	1
ENSGALG00C	SAT1	spermidine	yes|up	1
ENSGALG00C	PDGFRL	platelet c	yes|down	1
ENSGALG00C	SCEL	sciellin [	yes|down	1
ENSGALG00C	TIMM21	transloca s	yes|down	1
ENSGALG00C	OLFM1	olfactomec	yes|down	1
ENSGALG00C	CYP26C1	cytochrome	yes|up	1
ENSGALG00C	MSLN	mesothelin	yes|up	1
ENSGALG00000033591		kazal-type	yes|down	1

ENSGALG00000002046	synaptotag	yes|down	1
ENSGALG00000028016	eye-globir	yes|up	1
ENSGALG00000046192	c-C motif	yes|up	1
ENSGALG00C HES1	hes family	yes|up	1
ENSGALG00C NAB2	NGFI-A bir	yes|up	1
ENSGALG00C DENND3	DENN domai	yes|up	1
ENSGALG00000035027		yes|up	1
ENSGALG00C SLC22A4	solute car	yes|down	1
ENSGALG00C TMEM37	transmembr	yes|up	1
ENSGALG00000027778	ras-relate	yes|down	1
ENSGALG00C RAB15	RAB15, mem	yes|up	1
ENSGALG00C RSAD2	radical S-	yes|up	1
ENSGALG00C EMILIN3	elastin mi	yes|down	1
ENSGALG00000034000	uncharacte	yes|up	1
ENSGALG00C EPHA2	EPH recept	yes|up	1
ENSGALG00C RBM47	RNA bindir	yes|up	1
ENSGALG00000016127		yes|down	1
ENSGALG00C TTLL9	tubulin ty	yes|up	1
ENSGALG00C SNTB1	syntrophir	yes|down	1
ENSGALG00C ANKRD13B	ankyrin re	yes|down	1
ENSGALG00C MSANTD1	Myb/SANT I	yes|up	1
ENSGALG00C RGS4	regulator	yes|up	1
ENSGALG00000048605		yes|down	1
ENSGALG00000046425		yes|up	1
ENSGALG00C FRZB	frizzled r	yes|down	1
ENSGALG00C EXFABP	extracellu	yes|up	1
ENSGALG00C TNFAIP6	TNF alpha	yes|up	1
ENSGALG00C PLEKHG5	pleckstrir	yes|up	1
ENSGALG00C XKR8	XK relatec	yes|up	1
ENSGALG00000001963	serine/thr	yes|down	1
ENSGALG00000029333		yes|down	1
ENSGALG00C CALD1	caldesmon	yes|down	1
ENSGALG00000001620	cutA dival	yes|up	1
ENSGALG00C RPL26L1	ribosomal	yes|down	1
ENSGALG00000006864	collagen t	yes|down	1
ENSGALG00000048781		yes|up	1
ENSGALG00C GCAT	glycine C-	yes|down	1
ENSGALG00000054613		yes|down	1
ENSGALG00C VWCE	von Willet	yes|down	1
ENSGALG00000037870		yes|down	1
ENSGALG00C GCNT2	glucosamir	yes|down	1
ENSGALG00C CISH	cytokine i	yes|down	1
ENSGALG00C KLHL6	kelch like	yes|up	1
ENSGALG00C SLC22A16	solute car	yes|down	1
ENSGALG00000047181		yes|up	1
ENSGALG00000048048	mucin 4, c	yes|up	1
ENSGALG00C NME5	NME/NM23 f	yes|down	1
ENSGALG00000003645	phosphatic	yes|down	1
ENSGALG00C PECAM1	platelet a	yes|down	1
ENSGALG00C CCDC80	coiled-coi	yes|down	1
ENSGALG00C PDGFA	platelet c	yes|up	1

ENSGALG00000046693		yes|up	1
ENSGALG00C GATA6	GATA bindi	yes|up	1
ENSGALG00C SLC2A5	solute car	yes|down	1
ENSGALG00C FSTL1	follistati	yes|down	1
ENSGALG00C HBEGF	heparin bi	yes|up	1
ENSGALG00C IQGAP2	IQ motif c	yes|down	1
ENSGALG00000030571		yes|up	1
ENSGALG00C EPAS1	endothelia	yes|up	1
ENSGALG00C CCR7	C-C motif	yes|up	1
ENSGALG00C GLT8D2	glycosyltr	yes|down	1
ENSGALG00C CACNA1E	calcium vc	yes|up	1
ENSGALG00C RPL5	ribosomal	yes|down	1
ENSGALG00C EPB41L1	erythrocyt	yes|down	1
ENSGALG00C ADAMTS17	ADAM metal	yes|down	1
ENSGALG00000047569	plectin ps	yes|up	1
ENSGALG00C GAB2	GRB2 assoc	yes|down	1
ENSGALG00000033780		yes|up	1
ENSGALG00C PARP9	poly(ADP-r	yes|up	1
ENSGALG00C SDC4	syndecan 4	yes|up	1
ENSGALG00000047567		yes|up	1
ENSGALG00C DDX4	DEAD-box h	yes|down	1
ENSGALG00C PLPP1	phospholip	yes|down	1
ENSGALG00C CEP57L1	centrosoma	yes|down	1
ENSGALG00C RPL37	ribosomal	yes|down	1
ENSGALG00000047369		yes|up	1
ENSGALG00C CD44	CD44 molec	yes|up	1
ENSGALG00C PCBP4	poly(rC)-b	yes|up	1
ENSGALG00000027983	tyrosine k	yes|up	1
ENSGALG00C LMOD1	leiomodin	yes|down	1
ENSGALG00000052589		yes|up	1
ENSGALG00C GGT5	gamma-glut	yes|up	1
ENSGALG00000049810		yes|up	1
ENSGALG00C CTNNA2	catenin al	yes|down	1
ENSGALG00C RASAL2	synaptic R	yes|up	1
ENSGALG00C ITFG1	integrin a	yes|up	1
ENSGALG00C CHST13	carbohydra	yes|down	1
ENSGALG00C PAPPA2	pappalysin	yes|up	1
ENSGALG00000051244		yes|down	1
ENSGALG00C SHTN1	shootin 1	yes|up	1
ENSGALG00000052586		yes|up	1
ENSGALG00C KIF25	kinesin fa	yes|down	1
ENSGALG00C RPL31	ribosomal	yes|down	1
ENSGALG00C OSTM1	osteopetrc	yes|up	1
ENSGALG00C HKDC1	hexokinase	yes|down	1
ENSGALG00000005814	KIAA1161 [	yes|down	1
ENSGALG00C NAPEPLD	N-acyl phc	yes|down	1
ENSGALG00C VDR	vitamin D	yes|up	1
ENSGALG00C NEURL1	neuralizec	yes|up	1
ENSGALG00000001805	matrix rem	yes|down	1
ENSGALG00C FAM110C	family wit	yes|down	1
ENSGALG00C PHACTR3	phosphatas	yes|up	1

ENSGALG00000013994		yes|up	1
ENSGALG00000047412	poly [ADP-	yes|up	1
ENSGALG00C RPS12	ribosomal	yes|down	1
ENSGALG00C VNN1	vanin 1 [S	yes|up	1
ENSGALG00C LAMP3	lysosomal	yes|up	1
ENSGALG00000051832		yes|up	1
ENSGALG00000028341	mucosal va	yes|up	1
ENSGALG00C ACVR1C	activin A	yes|down	1
ENSGALG00C CD248	CD248 mole	yes|up	1
ENSGALG00C NEUROD6	neuronal c	yes|up	1
ENSGALG00C SEC16B	SEC16 homc	yes|up	1
ENSGALG00C CASP8	caspase 8	yes|up	1
ENSGALG00C AIF1L	allograft	yes|down	1
ENSGALG00C PTPRU	protein ty	yes|down	1
ENSGALG00C IFFO2	intermedia	yes|up	1
ENSGALG00000050208		yes|up	1
ENSGALG00C CSMD1	CUB and Su	yes|down	1
ENSGALG00000005958		yes|down	1
ENSGALG00C F10	coagulatic	yes|up	1
ENSGALG00000033653	hyperpolar	yes|down	1
ENSGALG00000050752		yes|up	1
ENSGALG00000050751		yes|up	1
ENSGALG00C IQCC	IQ motif c	yes|up	1
ENSGALG00000046908		yes|up	1
ENSGALG00C gga-mir-15	gga-mir-15	yes|up	1
ENSGALG00C STAT2	signal tra	yes|up	1
ENSGALG00C TIFA	TRAF inter	yes|up	1
ENSGALG00C VTN	vitronecti	yes|down	1
ENSGALG00C RPL8	ribosomal	yes|down	1
ENSGALG00C SESTD1	SEC14 and	yes|down	1
ENSGALG00C SLC48A1	solute car	yes|down	1
ENSGALG00000036831	cytochrome	yes|down	1
ENSGALG00C ADRB2	adrenocept	yes|down	1
ENSGALG00C DEPDC7	DEP domain	yes|up	1
ENSGALG00C FGL1	fibrinogen	yes|down	1
ENSGALG00000011687	AHNAK nucl	yes|down	1
ENSGALG00C PSTPIP2	proline-se	yes|up	1
ENSGALG00C LRRC34	leucine ri	yes|down	1
ENSGALG00000036838	peroxidasi	yes|down	1
ENSGALG00000051752		yes|down	1
ENSGALG00000050118		yes|up	1
ENSGALG00C SIVA1	SIVA1 apop	yes|up	1
ENSGALG00C PDZRN4	PDZ domain	yes|down	1
ENSGALG00C TSPAN15	tetraspani	yes|up	1
ENSGALG00C NT5DC3	5'-nucleot	yes|down	1
ENSGALG00C DUSP6	dual speci	yes|up	1
ENSGALG00C KITLG	KIT liganc	yes|down	1
ENSGALG00000001894		yes|up	1
ENSGALG00C HPSE	heparanase	yes|up	1
ENSGALG00C DDX60	DExD/H-box	yes|up	1
ENSGALG00C THBS2	thrombospc	yes|down	1

ENSGALG00000033009			yes|down	1
ENSGALG00C	TSNARE1	t-SNARE dc	yes|down	1
ENSGALG00C	REEP1	receptor a	yes|down	1
ENSGALG00000004814		rhophilin	yes|up	1
ENSGALG00C	ZNF106	zinc finge	yes|down	1
ENSGALG00000008141			yes|down	1
ENSGALG00C	PLAC9	placenta s	yes|down	1
ENSGALG00000044463			yes|up	1
ENSGALG00C	IARS1	isoleucyl-	yes|down	1
ENSGALG00C	TOR4A	torsin fam	yes|up	1
ENSGALG00C	AVPR2	arginine v	yes|down	1
ENSGALG00C	UAP1L1	UDP-N-acet	yes|down	1
ENSGALG00C	IL10RB	interleuki	yes|up	1
ENSGALG00C	AP1S3	adaptor re	yes|up	1
ENSGALG00C	SFRP2	secreted f	yes|down	1
ENSGALG00C	PCDH10	protocadhe	yes|up	1
ENSGALG00C	F8	coagulatic	yes|up	1
ENSGALG00C	C26H6orf89	chromosome	yes|down	1
ENSGALG00C	CHADL	chondroadh	yes|up	1
ENSGALG00C	LUM	lumican [S	yes|down	1
ENSGALG00C	EPHB1	EPH recept	yes|down	1
ENSGALG00C	DKK3	dickkopf W	yes|down	1
ENSGALG00000055112			yes|up	1
ENSGALG00C	FBLN2	fibulin 2	yes|down	1
ENSGALG00C	CERS6	ceramide s	yes|down	1
ENSGALG00000050475			yes|up	1
ENSGALG00C	COLEC10	collectin	yes|down	1
ENSGALG00C	MCRIP2	family wit	yes|down	1
ENSGALG00C	RASSF1	Ras associ	yes|up	1
ENSGALG00C	ELN	elastin [S	yes|down	1
ENSGALG00C	AFAP1L1	actin fila	yes|down	1
ENSGALG00C	SYTL4	synaptotag	yes|up	1
ENSGALG00000047705			yes|down	1
ENSGALG00C	NKAIN4	Na+/K+ tra	yes|down	1
ENSGALG00C	IRF1	interferon	yes|up	1
ENSGALG00000016934			yes|up	1
ENSGALG00C	ASB2	ankyrin re	yes|down	1
ENSGALG00000005675		PTTG1 inte	yes|down	1
ENSGALG00000050872			yes|down	1
ENSGALG00000031794		programmec	yes|up	1
ENSGALG00C	IER5L	immediate	yes|up	1
ENSGALG00000054572			yes|up	1
ENSGALG00C	ADAMTS8	ADAM metal	yes|down	1
ENSGALG00C	DHRS9	retinol de	yes|down	1
ENSGALG00C	CLEC3B	C-type lec	yes|down	1
ENSGALG00C	KLHDC8B	kelch doma	yes|up	1
ENSGALG00C	SUSD3	sushi doma	yes|up	1
ENSGALG00C	MCFD2	multiple c	yes|down	1
ENSGALG00C	KANK1	KN motif a	yes|down	1
ENSGALG00C	STOML1	stomatin l	yes|up	1
ENSGALG00C	JAM3	junctional	yes|down	1

ENSGALG00C	SLC26A3	solute car	yes|up	1
ENSGALG00C	TLR5	toll like	yes|down	1
ENSGALG00C	ITIH5	inter-alph	yes|down	1
ENSGALG00C	STC2	stanniocal	yes|down	1
ENSGALG00C	AKR1D1	aldo-keto	yes|down	1
ENSGALG00C	MMP16	matrix met	yes|down	1
ENSGALG00C	ACVR2B	activin A	yes|down	1
ENSGALG00C	RPS13	ribosomal	yes|down	1
ENSGALG00C	IRF4	interferon	yes|up	1
ENSGALG00000006152		tyrosine-p	yes|up	1
ENSGALG00C	SLC9A3R2	SLC9A3 reg	yes|down	1
ENSGALG00C	OGFR	opioid grc	yes|up	1
ENSGALG00C	GPX7	glutathion	yes|down	1
ENSGALG00000050526			yes|up	1
ENSGALG00C	RHOU	ras homolc	yes|down	1
ENSGALG00C	C3orf52	chromosome	yes|up	1
ENSGALG00000053296		chromosome	yes|up	1
ENSGALG00C	RAPSN	receptor a	yes|down	1
ENSGALG00C	IL17RA	interleuki	yes|up	1
ENSGALG00C	NCAPG2	non-SMC cc	yes|up	1
ENSGALG00C	SEMA3A	semaphorin	yes|down	1
ENSGALG00000053352			yes|up	1
ENSGALG00000033925		uncharacte	yes|down	1
ENSGALG00000004231		Interferon	yes|up	1
ENSGALG00C	SOX8	SRY-box 8	yes|down	1
ENSGALG00C	PLSCR1	phospholip	yes|up	1
ENSGALG00C	GPR68	G protein-	yes|up	1
ENSGALG00C	MPZL2	myelin prc	yes|up	1
ENSGALG00C	POU2AF1	POU class	yes|up	1
ENSGALG00C	TRIM55	tripartite	yes|down	1
ENSGALG00000013057		ubiquitin	yes|up	1
ENSGALG00C	KCTD12	potassium	yes|up	1
ENSGALG00C	SGCG	sarcoglyca	yes|down	1
ENSGALG00C	PROK2	prokinetic	yes|up	1
ENSGALG00C	NFIX	nuclear fa	yes|down	1
ENSGALG00000039652		Rho guanin	yes|up	1
ENSGALG00000047968			yes|down	1
ENSGALG00C	C5H11ORF96	chromosome	yes|down	1
ENSGALG00C	CENPS	centromere	yes|down	1
ENSGALG00000053759			yes|down	1
ENSGALG00C	FBXL8	F-box and	yes|down	1
ENSGALG00C	POMC	proopiomel	yes|down	1
ENSGALG00C	CAPN13	calpain 13	yes|up	1
ENSGALG00000041296		SRY-box 7	yes|up	1
ENSGALG00C	SREBF1	sterol reg	yes|up	1
ENSGALG00000053122			yes|down	1
ENSGALG00C	SCUBE2	signal pep	yes|down	1
ENSGALG00C	CPAMD8	C3 and PZP	yes|down	1
ENSGALG00C	ARHGEF9	Cdc42 guan	yes|down	1
ENSGALG00C	FAT3	FAT atypic	yes|down	1
ENSGALG00C	GFRA2	GDNF family	yes|down	1

ENSGALG00000053724		yes|down	1
ENSGALG00000039334	chromosome	yes|down	1
ENSGALG00C UGGT2	UDP-glucos	yes|down	1
ENSGALG00C VIP	vasoactive	yes|up	1
ENSGALG00C FBXO5	F-box prot	yes|up	1
ENSGALG00C PRCP	prolylcarb	yes|down	1
ENSGALG00C CYTB	cytochrome	yes|down	1
ENSGALG00C ARHGAP1	Rho GTPase	yes|down	1
ENSGALG00C EDN1	endothelin	yes|up	1
ENSGALG00000038978	dpy-19 lik	yes|down	1
ENSGALG00C RPLP0	ribosomal	yes|down	1
ENSGALG00C SETD7	SET domain	yes|down	1
ENSGALG00000013239		yes|up	1
ENSGALG00000043772	loss of he	yes|down	1
ENSGALG00C GADD45G	growth arr	yes|down	1
ENSGALG00000007123	uncharacte	yes|up	1
ENSGALG00000046940		yes|up	1
ENSGALG00000031398		yes|down	1
ENSGALG00C ASPHD2	aspartate	yes|down	1
ENSGALG00000054999		yes|down	1
ENSGALG00C MX1	myxovirus	yes|up	1
ENSGALG00C SORBS2	sorbin and	yes|down	1
ENSGALG00000035017	protein ty	yes|up	1
ENSGALG00C SYT6	synaptotag	yes|down	1
ENSGALG00000054995		yes|down	1
ENSGALG00C FBLN7	fibulin 7	yes|down	1
ENSGALG00C ID4	inhibitor	yes|up	1
ENSGALG00C RHOB	ras homolo	yes|down	1
ENSGALG00C RPS23	ribosomal	yes|down	1
ENSGALG00000012121	reticuloca	yes|down	1
ENSGALG00000042073	putative m	yes|down	1
ENSGALG00C FAM163B	family wit	yes|up	1
ENSGALG00C gga-mir-74	gga-mir-74	yes|down	1
ENSGALG00C PELI2	pellino E3	yes|down	1
ENSGALG00000012080		yes|up	1
ENSGALG00C NID1	nidogen 1	yes|down	1
ENSGALG00000050534		yes|down	1
ENSGALG00C TGFA	transformi	yes|up	1
ENSGALG00C MYCN	v-myc avia	yes|down	1
ENSGALG00C POU3F4	POU class	yes|up	1
ENSGALG00C ROS1	ROS proto-	yes|down	1
ENSGALG00C RPS15A	ribosomal	yes|down	1
ENSGALG00000048029		yes|up	1
ENSGALG00000012061	glycerol-3	yes|down	1
ENSGALG00000034305	chromosome	yes|down	1
ENSGALG00C HOOK3	hook micro	yes|down	1
ENSGALG00000053785		yes|up	1
ENSGALG00C RPS10	ribosomal	yes|down	1
ENSGALG00C GTF3C6	general tr	yes|down	1
ENSGALG00C NDST4	N-deacetyl	yes|up	1
ENSGALG00C GRM3	glutamate	yes|up	1

ENSGALG00CLVRN	laeverin [	yes|down	1
ENSGALG00000048333	protocadhe	yes|down	1
ENSGALG00CPHLDA1	pleckstrin	yes|up	1
ENSGALG00CCDHR1	cadherin r	yes|up	1
ENSGALG00CPTGER2	prostaglar	yes|up	1
ENSGALG00CKIF5C	kinesin fa	yes|up	1
ENSGALG00000048701		yes|down	1
ENSGALG00COSTN	osteocrin	yes|down	1
ENSGALG00000052451	uncharacte	yes|up	1
ENSGALG00CSCUBE3	signal pep	yes|up	1
ENSGALG00CGAS2	growth arr	yes|up	1
ENSGALG00CIL21R	interleuki	yes|up	1
ENSGALG00CARHGAP40	Rho GTPase	yes|down	1
ENSGALG00CPGF	placental	yes|up	1
ENSGALG00000028135		yes|down	1
ENSGALG00CRAB11FIP4	RAB11 fami	yes|down	1
ENSGALG00000038593		yes|up	1
ENSGALG00CCDCA7L	cell divis	yes|down	1
ENSGALG00000040680		yes|down	1
ENSGALG00CDSG2	desmoglein	yes|up	1
ENSGALG00CBLB2	Major hist	yes|up	1
ENSGALG00000049101		yes|up	1
ENSGALG00CHIC1	HIC ZBTB t	yes|down	1
ENSGALG00CCNP	2',3'-cycl	yes|up	1
ENSGALG00000046702		yes|down	1
ENSGALG00CPRDM1	PR/SET dom	yes|up	1
ENSGALG00CSORCS2	sortilin r	yes|down	1
ENSGALG00CLY96	lymphocyte	yes|up	1
ENSGALG00000046705		yes|up	1
ENSGALG00CPTGES	prostaglar	yes|up	1
ENSGALG00CWNT5A	Wnt family	yes|down	1
ENSGALG00CIL15	interleuki	yes|up	1
ENSGALG00CRERG	RAS like e	yes|down	1
ENSGALG00CHMGA2	high mobil	yes|up	1
ENSGALG00CCAPN6	calpain 6	yes|down	1
ENSGALG00CSMCHD1	structural	yes|up	1
ENSGALG00000047719		yes|down	1
ENSGALG00CNR2F1	nuclear re	yes|down	1
ENSGALG00CPRKCB	protein ki	yes|up	1
ENSGALG00CCYFIP2	cytoplasmi	yes|up	1
ENSGALG00000028222	uncharacte	yes|up	1
ENSGALG00000052987		yes|down	1
ENSGALG00CSTXBP4	syntaxin b	yes|down	1
ENSGALG00CC15orf62	chromosome	yes|up	1
ENSGALG00000052983		yes|down	1
ENSGALG00CCPZ	carboxypep	yes|down	1
ENSGALG00CTDRD12	tudor doma	yes|down	1
ENSGALG00CFOXP4	forkhead b	yes|up	1
ENSGALG00000044293		yes|down	1
ENSGALG00CGMIP	GEM intera	yes|up	1
ENSGALG00000011166	protein ki	yes|up	1

ENSGALG00000011164	chimerin 2	yes|up	1
ENSGALG000C	FOXF1	forkhead b	yes|up	1
ENSGALG00000050660		yes|up	1
ENSGALG000C DACT2	dishevelle	yes|up	1
ENSGALG000C RPS3A	ribosomal	yes|down	1
ENSGALG00000011285	nudix hydr	yes|down	1
ENSGALG00000042293		yes|up	1
ENSGALG00000011283	zinc finge	yes|down	1
ENSGALG00000043829	exostosin	yes|up	1
ENSGALG000C SGO1	shugoshin	yes|up	1
ENSGALG00000045436		yes|down	1
ENSGALG000C LRRC38	leucine ri	yes|down	1
ENSGALG000C TBX22	T-box 22 [	yes|down	1
ENSGALG000C TUBAL3	tubulin al	yes|down	1
ENSGALG000C TENM1	teneurin t	yes|down	1
ENSGALG00000047499		yes|down	1
ENSGALG000C SNTA1	syntrophin	yes|down	1
ENSGALG000C GDF7	growth dif	yes|down	1
ENSGALG00000052936		yes|down	1
ENSGALG000C CNDP2	CNDP dipep	yes|down	1
ENSGALG00000052523		yes|up	1
ENSGALG000C HOMER1	homer scaf	yes|down	1
ENSGALG00000023824	lipase mem	yes|down	1
ENSGALG000C SPTBN1	spectrin b	yes|down	1
ENSGALG000C DHX58	DExH-box h	yes|up	1
ENSGALG000C RPL27A	ribosomal	yes|down	1
ENSGALG000C NEBL	nebulette	yes|down	1
ENSGALG000C TMCC2	transmembr	yes|down	1
ENSGALG000C TTC19	tetratrico	yes|up	1
ENSGALG000C SSTR2	somatostat	yes|up	1
ENSGALG000C NRG2	neuregulin	yes|up	1
ENSGALG000C ITGB1BP3	integrin b	yes|down	1
ENSGALG00000054104	interferon	yes|up	1
ENSGALG00000053028		yes|down	1
ENSGALG000C CD36	CD36 molec	yes|down	1
ENSGALG000C MND1	meiotic nu	yes|down	1
ENSGALG000C SLC5A7	solute car	yes|up	1
ENSGALG000C GNAI1	G protein	yes|down	1
ENSGALG000C AADAC	arylacetam	yes|down	1
ENSGALG000C CADM3	cell adhes	yes|down	1
ENSGALG000C PRDX1	peroxiredo	yes|up	1
ENSGALG00000044739		yes|up	1
ENSGALG000C PTDSS2	phosphatid	yes|down	1
ENSGALG000C KLHDC3	kelch doma	yes|down	1
ENSGALG00000046914		yes|down	1
ENSGALG00000040313		yes|up	1
ENSGALG000C BEAN1	brain expr	yes|down	1
ENSGALG000C ZNF804B	zinc finge	yes|up	1
ENSGALG000C BAALC	brain and	yes|up	1
ENSGALG000C IQGAP3	ras GTPase	yes|down	1
ENSGALG00000051823		yes|down	1

ENSGALG00C	C1orf115	chromosome	yes|up	1
ENSGALG00000050269			yes|down	1
ENSGALG00C	BCKDHB	branched c	yes|down	1
ENSGALG00C	EFNB2	ephrin B2	yes|up	1
ENSGALG00C	FAP	fibroblast	yes|down	1
ENSGALG00C	FBXO2	F-box prot	yes|up	1
ENSGALG00C	AVPR1B	arginine v	yes|up	1
ENSGALG00C	MYBPHL	myosin bir	yes|down	1
ENSGALG00C	STARD5	StAR relat	yes|up	1
ENSGALG00C	HHIPL2	HHIP like	yes|down	1
ENSGALG00C	MEP1A	meprin A s	yes|down	1
ENSGALG00000048802			yes|up	1
ENSGALG00C	PTAFR	platelet a	yes|up	1
ENSGALG00000050064			yes|down	1
ENSGALG00C	CYSLTR2	cysteinyl	yes|down	1
ENSGALG00C	PRELID3A	PRELI doma	yes|up	1
ENSGALG00C	KCNB2	potassium	yes|down	1
ENSGALG00C	COMMD8	COMM domai	yes|up	1
ENSGALG00000008153		interleuki	yes|up	1
ENSGALG00C	MIDN	midnolin [	yes|up	1
ENSGALG00000009859		TBC1 domai	yes|up	1
ENSGALG00C	FHIP1A	family wit	yes|up	1
ENSGALG00000051964			yes|up	1
ENSGALG00C	PAPLN	papilin, p	yes|down	1
ENSGALG00000049680			yes|up	1
ENSGALG00000051969			yes|up	1
ENSGALG00C	SLC6A9	solute car	yes|down	1
ENSGALG00C	RPL22L1	ribosomal	yes|down	1
ENSGALG00C	CHST15	carbohydra	yes|down	1
ENSGALG00C	PDK4	pyruvate c	yes|down	1
ENSGALG00C	LOXL2	lysyl oxic	yes|down	1
ENSGALG00C	RAB7B	RAB7B, mem	yes|up	1
ENSGALG00C	BPIFB2	BPI fold c	yes|down	1
ENSGALG00C	PIK3AP1	phosphoinc	yes|up	1
ENSGALG00C	RAB29	RAB29, mem	yes|down	1
ENSGALG00C	TIMP2	TIMP metal	yes|down	1
ENSGALG00C	PPM1K	protein ph	yes|down	1
ENSGALG00000054064			yes|up	1
ENSGALG00C	ATP6V0E2	ATPase H+	yes|up	1
ENSGALG00C	STOX2	storkhead	yes|down	1
ENSGALG00000054067			yes|up	1
ENSGALG00C	PIK3IP1	phosphoinc	yes|down	1
ENSGALG00000037131		voltage-de	yes|up	1
ENSGALG00000054746			yes|down	1
ENSGALG00C	EIF2AK2	eukaryotic	yes|up	1
ENSGALG00C	KCNQ1	potassium	yes|down	1
ENSGALG00000049761			yes|down	1
ENSGALG00C	CHST11	carbohydra	yes|down	1
ENSGALG00C	IGFBP2	insulin li	yes|down	1
ENSGALG00C	IGFBP5	insulin li	yes|down	1
ENSGALG00C	MCOLN3	mucolipin	yes|up	1

ENSGALG00C	CYP2W1	cytochrome	yes|down	1
ENSGALG00C	G6PC2	glucose-6-	yes|down	1
ENSGALG00C	NFIB	nuclear fa	yes|down	1
ENSGALG00000038923		fatty acyl	yes|down	1
ENSGALG00C	ABCB11	ATP bindir	yes|down	1
ENSGALG00C	CDC42EP4	CDC42 effe	yes|up	1
ENSGALG00C	EPHX4	epoxide hy	yes|up	1
ENSGALG00C	PODXL	podocalyxi	yes|up	1
ENSGALG00000023083		KIAA1958 [	yes|down	1
ENSGALG00C	TNFSF10	tumor necr	yes|up	1
ENSGALG00000006407		Gallus gal	yes|up	1
ENSGALG00000054648		guanylate-	yes|up	1
ENSGALG00C	DOCK11	dedicator	yes|down	1
ENSGALG00C	TMEM243	transmembr	yes|up	1
ENSGALG00000000560		peptidase	yes|down	1
ENSGALG00C	SRD5A2	steroid 5	yes|up	1
ENSGALG00C	CMTR1	cap methyl	yes|up	1
ENSGALG00C	MFGE8	milk fat g	yes|down	1
ENSGALG00C	SMTNL2	smoothelir	yes|down	1
ENSGALG00C	TMEM61	transmembr	yes|down	1
ENSGALG00C	WWTR1	WW domain	yes|down	1
ENSGALG00000054366			yes|down	1
ENSGALG00000006708		collagen t	yes|down	1
ENSGALG00C	RBP	riboflavir	yes|down	1
ENSGALG00C	NALF2	family wit	yes|down	1
ENSGALG00C	SUSD4	complement	yes|down	1
ENSGALG00C	GSN	gelsolin [	yes|down	1
ENSGALG00C	TTLL2	tubulin ty	yes|down	1
ENSGALG00C	NFKBIE	NFKB inhit	yes|up	1
ENSGALG00C	TRPC4	transient	yes|down	1
ENSGALG00000009387		synaptojar	yes|up	1
ENSGALG00C	KLF15	Kruppel li	yes|down	1
ENSGALG00C	GPC3	glypican 3	yes|down	1
ENSGALG00C	ARSI	arylsulfat	yes|up	1
ENSGALG00000008936		ectonuclec	yes|up	1
ENSGALG00C	F13A1	coagulatic	yes|down	1
ENSGALG00C	CASP7	caspase 7	yes|up	1
ENSGALG00C	PKD2L2	polycystir	yes|up	1
ENSGALG00C	PKIA	protein ki	yes|down	1
ENSGALG00C	COCH	cochlin [S	yes|down	1
ENSGALG00000053285		mothers ag	yes|up	1
ENSGALG00C	DOK2	docking pr	yes|down	1
ENSGALG00C	CPB1	carboxypep	yes|down	1
ENSGALG00C	TNNC2	troponin C	yes|down	1
ENSGALG00000021405			yes|down	1
ENSGALG00C	TMEM86A	transmembr	yes|down	1
ENSGALG00000002671		transmembr	yes|up	1
ENSGALG00C	NT5C3B	5'-nucleot	yes|up	1
ENSGALG00C	HLX	H2.0 like	yes|up	1
ENSGALG00000007833		tetratricc	yes|down	1
ENSGALG00C	MICAL3	microtubul	yes|down	1

ENSGALG00000005257	vascular c	yes	up	1
ENSGALG00C	KAZALD1	Kazal type	yes	down	1
ENSGALG00C	GJB6	gap juncti	yes	up	1
ENSGALG00C	TWIST1	twist fami	yes	up	1
ENSGALG00C	CBFB	core-bindi	yes	down	1
ENSGALG00000037400		yes	up	1
ENSGALG00000042443		yes	up	1
ENSGALG00C	NOV	nephroblas	yes	up	1
ENSGALG00000047959		yes	down	1
ENSGALG00C	SLC46A2	solute car	yes	up	1
ENSGALG00C	VWA8	von Willet	yes	down	1
ENSGALG00C	MSX2	msh homeot	yes	up	1
ENSGALG00C	GALNT11	polypeptic	yes	down	1
ENSGALG00C	FAM107A	family wit	yes	down	1
ENSGALG00C	TPH1	tryptophar	yes	up	1
ENSGALG00C	gga-mir-21	gga-mir-21	yes	up	1
ENSGALG00C	JUND	JunD protc	yes	up	1
ENSGALG00C	CALHM6	family wit	yes	up	1
ENSGALG00C	PLTP	phospholip	yes	down	1
ENSGALG00C	RASD1	ras relate	yes	down	1
ENSGALG00C	NPM2	nucleophos	yes	down	1
ENSGALG00000016251	tetraspani	yes	down	1
ENSGALG00C	RFKL	riboflavir	yes	up	1
ENSGALG00C	GSTA3	glutathior	yes	up	1
ENSGALG00C	GSTA4	glutathior	yes	down	1
ENSGALG00000016326	organic sc	yes	up	1
ENSGALG00000016322	glutathior	yes	down	1
ENSGALG00C	APOBEC2	apolipoprc	yes	down	1
ENSGALG00C	UBASH3B	ubiquitin	yes	up	1
ENSGALG00C	FGR	FGR proto-	yes	up	1
ENSGALG00000037533		yes	down	1
ENSGALG00000001483	Golgi inte	yes	down	1
ENSGALG00C	ST8SIA2	ST8 alpha-	yes	down	1
ENSGALG00C	ARHGAP8	Rho GTPase	yes	up	1
ENSGALG00C	BACE2	beta-secre	yes	down	1
ENSGALG00C	WASF3	WAS protei	yes	down	1
ENSGALG00000046210		yes	up	1
ENSGALG00000039008	uncharacte	yes	down	1
ENSGALG00000039164	paracaspas	yes	up	1
ENSGALG00C	APOA1	apolipoprc	yes	down	1
ENSGALG00C	ADCY6	adenylate	yes	down	1
ENSGALG00C	PSPH	phosphoser	yes	down	1
ENSGALG00C	TOR1AIP2	torsin 1A	yes	up	1
ENSGALG00000007110	transmembr	yes	down	1
ENSGALG00C	IMP3	IMP3, U3 s	yes	up	1
ENSGALG00C	FOS	Fos proto-	yes	up	1
ENSGALG00C	PDLIM3	PDZ and LI	yes	down	1
ENSGALG00C	ADGRB3	adhesion C	yes	down	1
ENSGALG00C	PCSK6	proproteir	yes	down	1
ENSGALG00C	DIO2	deiodinase	yes	up	1
ENSGALG00C	SLC22A31	solute car	yes	up	1

ENSGALG00C	CSF1R	colony sti	yes|down	1
ENSGALG00000042388			yes|down	1
ENSGALG00000045817			yes|up	1
ENSGALG00C	TBXAS1	thromboxar	yes|up	1
ENSGALG00C	CRLF2	cytokine r	yes|up	1
ENSGALG00C	FBN3	fibrillin	yes|down	1
ENSGALG00C	RGS1	regulator	yes|up	1
ENSGALG00C	TRANK1	tetratricc	yes|up	1
ENSGALG00000039468		schwannomi	yes|up	1
ENSGALG00C	COL5A1	collagen t	yes|down	1
ENSGALG00C	GRIA4	glutamate	yes|up	1
ENSGALG00C	CDH17	cadherin 1	yes|up	1
ENSGALG00C	CSGALNACT2	chondroiti	yes|down	1
ENSGALG00C	DPP10	dipeptidyl	yes|down	1
ENSGALG00C	NUAK2	NUAK famil	yes|up	1
ENSGALG00C	TNFRSF11B	TNF recept	yes|up	1
ENSGALG00C	SLA	Src like a	yes|down	1
ENSGALG00000036613			yes|up	1
ENSGALG00C	Pou5f3	POU domair	yes|up	1
ENSGALG00C	HOXA4	homeobox A	yes|up	1
ENSGALG00C	RHOG	ras homolc	yes|up	1
ENSGALG00C	RNF213	ring finge	yes|up	1
ENSGALG00000022875		myelin oli	yes|up	1
ENSGALG00000047397			yes|up	1
ENSGALG00000039754			yes|down	1
ENSGALG00C	WNT11B	wingless-t	yes|up	1
ENSGALG00C	SH3GL2	SH3 domair	yes|down	1
ENSGALG00C	TAP1	transporte	yes|up	1
ENSGALG00000004627			yes|up	1
ENSGALG00000053084			yes|up	1
ENSGALG00C	KCNK5	potassium	yes|up	1
ENSGALG00C	LAMC1	laminin su	yes|down	1
ENSGALG00C	DHFR	dihydrofol	yes|down	1
ENSGALG00C	SLC30A8	solute car	yes|down	1
ENSGALG00C	PAQR8	progestin	yes|down	1
ENSGALG00000012072		poly(ADP-r	yes|up	1
ENSGALG00C	DTX3L	deltex E3	yes|up	1
ENSGALG00C	ALPK1	alpha kina	yes|up	1
ENSGALG00C	PABPC1	poly(A) bi	yes|down	1
ENSGALG00C	RPL35	ribosomal	yes|down	1
ENSGALG00C	HNMT	histamine	yes|down	1
ENSGALG00000048017			yes|down	1
ENSGALG00C	UHRF1	ubiquitin	yes|up	1
ENSGALG00C	PACSIN1	protein ki	yes|down	1
ENSGALG00000048309		uncharacte	yes|down	1
ENSGALG00000048658			yes|down	1
ENSGALG00C	ZNF462	zinc finge	yes|down	1
ENSGALG00C	EEF1A2	eukaryotic	yes|down	1
ENSGALG00C	CCL4	C-C motif	yes|up	1
ENSGALG00C	IL2RB	interleuki	yes|down	1
ENSGALG00C	ANKDD1B	ankyrin re	yes|up	1

ENSGALG00000012382		yes|down	1
ENSGALG00C BATF3	basic leuc	yes|up	1
ENSGALG00C IGFALS	insulin li	yes|up	1
ENSGALG00000047769		yes|up	1
ENSGALG00C BAIAP2L1	BAI1 assoc	yes|up	1
ENSGALG00000043381	C-RF amide	yes|up	1
ENSGALG00000052745		yes|up	1
ENSGALG00C ARL4C	ADP ribosy	yes|up	1
ENSGALG00C CRYM	crystallir	yes|down	1
ENSGALG00C ARHGAP6	Rho GTPase	yes|down	1
ENSGALG00C WFIKKN1	WAP, folli	yes|down	1
ENSGALG00C PI15	peptidase	yes|up	1
ENSGALG00C TMOD3	tropomodul	yes|down	1
ENSGALG00C PTPRT	protein ty	yes|down	1
ENSGALG00C EFR3B	EFR3 homol	yes|up	1
ENSGALG00C P2RX6	purinergic	yes|down	1
ENSGALG00000055067		yes|down	1
ENSGALG00000048563		yes|up	1
ENSGALG00C ARL4A	ADP ribosy	yes|down	1
ENSGALG00000034204	keratin, t	yes|up	1
ENSGALG00C RWDD3	RWD domair	yes|down	1
ENSGALG00000043947	G-protein	yes|up	1
ENSGALG00C GCH1	GTP cyclob	yes|up	1
ENSGALG00C COL8A1	collagen t	yes|down	1
ENSGALG00C AMY2A	amylase, a	yes|up	1
ENSGALG00C RB1CC1	RB1 induci	yes|up	1
ENSGALG00000048571		yes|down	1
ENSGALG00C CD200L	CD200 mole	yes|up	1
ENSGALG00C MGLL	monoglycer	yes|down	1
ENSGALG00C CD200	CD200 mole	yes|up	1
ENSGALG00000047118		yes|down	1
ENSGALG00000052087		yes|up	1
ENSGALG00000048402		yes|down	1
ENSGALG00000048401		yes|down	1
ENSGALG00000003283	cartilage	yes|down	1
ENSGALG00000049975		yes|up	1
ENSGALG00000031066		yes|down	1
ENSGALG00000048159	taperin [S	yes|up	1
ENSGALG00C FABP3	fatty acic	yes|down	1
ENSGALG00000051458		yes|up	1
ENSGALG00C PIK3R1	phosphoinc	yes|down	1
ENSGALG00000054964	putative E	yes|down	1
ENSGALG00C CFD	complement	yes|up	1
ENSGALG00C PCYT2	phosphate	yes|down	1
ENSGALG00C SYT12	synaptotag	yes|down	1
ENSGALG00000015324	family wit	yes|down	1
ENSGALG00C UACA	uveal autc	yes|down	1
ENSGALG00C WIPF3	WAS/WASL i	yes|down	1
ENSGALG00000011172	myosin-X-l	yes|up	1
ENSGALG00C SSPN	sarcospan	yes|down	1
ENSGALG00C POU3F3	POU class	yes|up	1

ENSGALG00C	DERL3	derlin 3 [	yes|down	1
ENSGALG00C	ADAMTS15	ADAM metal	yes|down	1
ENSGALG00C	BHLHE40	basic heli	yes|up	1
ENSGALG00C	IL11	interleuki	yes|up	1
ENSGALG00C	GRIA1	glutamate	yes|down	1
ENSGALG00C	IGF2	insulin li	yes|down	1
ENSGALG00C	MINDY3	family wit	yes|up	1
ENSGALG00000049424			yes|down	1
ENSGALG00C	PHYHIPL	phytanoyl-	yes|down	1
ENSGALG00C	PHETA1	family wit	yes|up	1
ENSGALG00C	CBX7	chromobox	yes|down	1
ENSGALG00C	COL5A2	collagen t	yes|down	1
ENSGALG00000004393		SITS-bindi	yes|down	1
ENSGALG00C	NMI	N-myc and	yes|up	1
ENSGALG00000051216			yes|up	1
ENSGALG00000052887		C-factor-l	yes|down	1
ENSGALG00C	INSYN2B	inhibitory	yes|down	1
ENSGALG00C	SAMD12	sterile al	yes|down	1
ENSGALG00C	CARD11	caspase re	yes|up	1
ENSGALG00000038728		probable (	yes|up	1
ENSGALG00C	NANOS1	nanos C2HC	yes|up	1
ENSGALG00C	ND5	NADH-ubiqu	yes|down	1
ENSGALG00C	SLIT3	slit guida	yes|down	1
ENSGALG00C	PHETA2	PH domain	yes|down	1
ENSGALG00C	IL18R1	interleuki	yes|up	1
ENSGALG00C	MMP10	matrix met	yes|up	1
ENSGALG00C	ZNF488	zinc finge	yes|up	1
ENSGALG00C	PTGS2	prostaglar	yes|up	1
ENSGALG00000037869		atrial nat	yes|down	1
ENSGALG00000053107			yes|down	1
ENSGALG00C	CDK15	cyclin dep	yes|down	1
ENSGALG00C	SIDT1	SID1 trans	yes|down	1
ENSGALG00C	TEC	tec protei	yes|up	1
ENSGALG00000048970			yes|up	1
ENSGALG00C	CREB3L1	cAMP respc	yes|down	1
ENSGALG00C	TIPARP	TCDD induc	yes|up	1
ENSGALG00C	MAP1A	microtubul	yes|down	1
ENSGALG00C	GNAT3	G protein	yes|down	1
ENSGALG00C	TRAF3	TNF recept	yes|up	1
ENSGALG00C	SLC8A1	solute car	yes|up	1
ENSGALG00C	SNAI1	snail fami	yes|up	1
ENSGALG00C	FAM20A	FAM20A, gc	yes|down	1
ENSGALG00C	RPL9	ribosomal	yes|down	1
ENSGALG00000051777			yes|up	1
ENSGALG00000051776			yes|down	1
ENSGALG00C	HYAL3	hyaluronog	yes|up	1
ENSGALG00C	NPPA	natriureti	yes|up	1
ENSGALG00000050176			yes|down	1
ENSGALG00C	FSTL3	follistati	yes|up	1
ENSGALG00C	TCEA3	transcript	yes|down	1
ENSGALG00C	MUSTN1	musculoske	yes|down	1

ENSGALG00C B3GNT9	UDP-GlcNAc	yes|down	1
ENSGALG00C RPL12	ribosomal	yes|down	1
ENSGALG00000041233		yes|down	1
ENSGALG00000035960	myosin-7-l	yes|down	1
ENSGALG00C C3orf14	chromosome	yes|down	1
ENSGALG00C FOXC1	forkhead b	yes|up	1
ENSGALG00000049699		yes|down	1
ENSGALG00000049751	histone cl	yes|down	1
ENSGALG00C TLR15	Gallus gal	yes|up	1
ENSGALG00C TMEM173	transmembr	yes|up	1
ENSGALG00C GDPD5	glyceropho	yes|down	1
ENSGALG00C ZBTB47	zinc finge	yes|down	1
ENSGALG00C TNFRSF21	TNF recept	yes|up	1
ENSGALG00C CTSS	cathepsin	yes|up	1
ENSGALG00000011799	heme bindi	yes|down	1
ENSGALG00C HEBP1	heme bindi	yes|down	1
ENSGALG00000009205	ankyrin re	yes|down	1
ENSGALG00C PPP4R4	protein ph	yes|down	1
ENSGALG00C DIP2C	disco inte	yes|down	1
ENSGALG00C KCNJ5	Potassium	yes|up	1
ENSGALG00000050556	translatic	yes|up	1
ENSGALG00000055077		yes|up	1
ENSGALG00C ITGB3	integrin s	yes|up	1
ENSGALG00C NOXO1	NADPH oxic	yes|up	1
ENSGALG00C UBTD1	ubiquitin	yes|up	1
ENSGALG00C MSC	musculin [	yes|down	1
ENSGALG00C SMYD3	SET and MY	yes|down	1
ENSGALG00C TRH	thyrotropi	yes|down	1
ENSGALG00000048907		yes|up	1
ENSGALG00C MYB	MYB proto-	yes|up	1
ENSGALG00C NAT8L	N-acetyltr	yes|down	1
ENSGALG00000049457		yes|down	1
ENSGALG00C ARHGAP45	Rho GTPase	yes|up	1
ENSGALG00C GLIPR1L	GLI pathog	yes|up	1
ENSGALG00C CD34	CD34 molec	yes|down	1
ENSGALG00000049868		yes|down	1
ENSGALG00C RAMP3	receptor a	yes|up	1
ENSGALG00C HIST1H46	histone cl	yes|down	1
ENSGALG00C GPT2	glutamic--	yes|down	1
ENSGALG00C UTS2R	urotensin-	yes|down	1
ENSGALG00000009603	prolactin-	yes|down	1
ENSGALG00C NELL2	neural EGF	yes|down	1
ENSGALG00000050951		yes|up	1
ENSGALG00000054659	transmembr	yes|down	1
ENSGALG00000050665		yes|down	1
ENSGALG00C ETV7	ETS variar	yes|up	1
ENSGALG00000050957		yes|up	1
ENSGALG00C IFNW1	interferor	yes|up	1
ENSGALG00C TMEM154	transmembr	yes|up	1
ENSGALG00C ALPK2	alpha kina	yes|down	1
ENSGALG00C SLC37A1	solute car	yes|up	1

ENSGALG00C RAB36	RAB36, mem	yes|down	1
ENSGALG00C NTN4	netrin 4 [	yes|up	1
ENSGALG00C HS3ST5	heparan su	yes|down	1
ENSGALG00C CSF3	colony sti	yes|up	1
ENSGALG00C GNAZ	G protein	yes|down	1
ENSGALG00C PTCH2	patched 2	yes|up	1
ENSGALG00000044841	tapasin-re	yes|up	1
ENSGALG00C FAM43A	family wit	yes|down	1
ENSGALG00C APOLD1	apolipopro	yes|up	1
ENSGALG00C RPS3	ribosomal	yes|down	1
ENSGALG00C ARHGAP10	Rho GTPase	yes|up	1
ENSGALG00C FAH	fumarylace	yes|down	1
ENSGALG00C OLFML1	olfactomec	yes|up	1
ENSGALG00C ZDHHC18	zinc finge	yes|up	1
ENSGALG00000053921	characte	yes|down	1
ENSGALG00C TNNC1	troponin C	yes|down	1
ENSGALG00C STX11	syntaxin 1	yes|up	1
ENSGALG00C FGF14	fibroblast	yes|down	1
ENSGALG00C TPST2	tyrosylpro	yes|down	1
ENSGALG00000034119	collagen t	yes|down	1
ENSGALG00C HELZ2	helicase w	yes|up	1
ENSGALG00C SPSB4	splA/ryanc	yes|down	1
ENSGALG00C DDO	D-aspartat	yes|down	1
ENSGALG00C COL9A3	collagen t	yes|up	1
ENSGALG00000050829		yes|up	1
ENSGALG00C CX3CL1	C-X3-C mot	yes|up	1
ENSGALG00C PARD6B	par-6 fami	yes|up	1
ENSGALG00C SPRY3	sprouty RT	yes|up	1
ENSGALG00C KYNU	kynurenina	yes|down	1
ENSGALG00000050668		yes|down	1
ENSGALG00C DCX	doublecort	yes|down	1
ENSGALG00C PHEX	phosphate	yes|down	1
ENSGALG00C VEPH1	ventricula	yes|up	1
ENSGALG00C PLA2G15	phospholip	yes|down	1
ENSGALG00C RASL11A	ras- like	yes|down	1
ENSGALG00C HIST1H2B7	histone cl	yes|down	1
ENSGALG00000017029	fibrinogen	yes|down	1
ENSGALG00C GOLGB1	golgin B1	yes|down	1
ENSGALG00C BST1	bone marro	yes|down	1
ENSGALG00000007530		yes|down	1
ENSGALG00C SERPINB10E	serpin pep	yes|up	1
ENSGALG00C SERPINB10	serpin fam	yes|up	1
ENSGALG00C TNMD	tenomoduli	yes|down	1
ENSGALG00C PTPRB	protein ty	yes|up	1
ENSGALG00C KLHL25	kelch like	yes|down	1
ENSGALG00C SERPINB1	serpin fam	yes|up	1
ENSGALG00C BCL2L13	BCL2 like	yes|down	1
ENSGALG00C NPY2R	neuropepti	yes|down	1
ENSGALG00C REL	REL proto-	yes|up	1
ENSGALG00000013033	carboxymet	yes|up	1
ENSGALG00C MTHFD1L	methylenet	yes|down	1

ENSGALG00C OLFM4	olfactomecyes	up	1
ENSGALG00C EGR3	early growyes	up	1
ENSGALG00C LECT1	leukocyte yes	down	1
ENSGALG00C IL2RA	interleukiyes	up	1
ENSGALG00C BID	BH3 interayes	up	1
ENSGALG00C PRKCD	protein kiyes	up	1
ENSGALG00C SYNDIG1	synapse diyes	up	1
ENSGALG00C RSPO4	R-spondin yes	up	1
ENSGALG00000039585	lymphocyteyes	up	1
ENSGALG00000002012	yes	up	1
ENSGALG00C CXCL14	C-X-C motiyes	up	1
ENSGALG00C WNK2	WNK lysineyes	down	1
ENSGALG00000007740	wiskott-Alyes	down	1
ENSGALG00000046160	chemokine yes	up	1
ENSGALG00000049005	yes	up	1
ENSGALG00C EGR1	early growyes	up	1
ENSGALG00C ESM1	endotheliayes	up	1
ENSGALG00C TAT	tyrosine ayes	down	1
ENSGALG00C K123	K123 proteyes	up	1
ENSGALG00C TK1	thymidine yes	up	1
ENSGALG00C MYCBPAP	radical S-yes	down	1
ENSGALG00000052001	yes	down	1
ENSGALG00C SYT2	synaptotagyes	down	1
ENSGALG00000053700	yes	down	1
ENSGALG00C RAB30	RAB30, memyes	down	1
ENSGALG00C PRSS23	protease, yes	down	1
ENSGALG00C ME3	malic enzyyes	down	1
ENSGALG00C CDKN2B	cyclin depyes	up	1
ENSGALG00C DPT	dermatoporyes	down	1
ENSGALG00C ADGRL4	adhesion Gyes	up	1
ENSGALG00000049412	yes	up	1
ENSGALG00C SLC7A2	solute caryes	down	1
ENSGALG00C PPP1R14C	protein phyes	down	1
ENSGALG00C SIPA1L2	signal incyes	down	1
ENSGALG00C RNF24	ring fingeyes	up	1
ENSGALG00C CDT1	chromatin yes	up	1
ENSGALG00C HACD4	3-hydroxyayes	up	1
ENSGALG00C CYP24A1	cytochromeyes	up	1
ENSGALG00C CSMD3	CUB and Suyes	up	1
ENSGALG00C LRRC74B	chromosomeyes	down	1
ENSGALG00000048824	yes	up	1
ENSGALG00000047735	yes	up	1
ENSGALG00C ECH1	enoyl-CoA yes	down	1
ENSGALG00000053483	protocadheyes	down	1
ENSGALG00C ADAMTS2	ADAM metalyes	down	1
ENSGALG00000015662	lipase memyes	up	1
ENSGALG00C ZAR1	zygote arryes	up	1
ENSGALG00000050245	yes	up	1
ENSGALG00C TNIP2	TNFAIP3 inyes	up	1
ENSGALG00C REPS2	RALBP1 assyes	down	1
ENSGALG00000037611	myosin IIIyes	down	1

ENSGALG00C	GSDMA	gasdermin	yes|up	1
ENSGALG00C	DEPDC6	DEP domair	yes|down	1
ENSGALG00C	FAM180A	family wit	yes|down	1
ENSGALG00C	MMP28	matrix met	yes|up	1
ENSGALG00C	DOCK2	dedicator	yes|down	1
ENSGALG00000042570		microtubul	yes|up	1
ENSGALG00C	OVALX	ovalbumin-	yes|down	1
ENSGALG00000043257		lysozyme g	yes|up	1
ENSGALG00C	NGF	nerve grow	yes|up	1
ENSGALG00C	RET	ret proto-	yes|down	1
ENSGALG00C	ABHD8	abhydrolas	yes|down	1
ENSGALG00C	PLEKHN1	pleckstrir	yes|up	1
ENSGALG00C	CYP1B1	cytochrome	yes|down	1
ENSGALG00C	RASSF9	Ras associ	yes|up	1
ENSGALG00C	RRM2	ribonuclec	yes|up	1
ENSGALG00C	ADAMTS5	ADAM metal	yes|down	1
ENSGALG00C	EIF5B	eukaryotic	yes|down	1
ENSGALG00C	LYG2	lysozyme g	yes|up	1
ENSGALG00000035100		characte	yes|down	1
ENSGALG00C	ANK2	ankyrin 2	yes|down	1
ENSGALG00000032803		proto-oncc	yes|up	1
ENSGALG00C	CASQ2	calsequest	yes|down	1
ENSGALG00C	IRF9	interferor	yes|up	1
ENSGALG00C	CMPK2	cytidine/u	yes|up	1
ENSGALG00C	CASP10	caspase 1C	yes|up	1
ENSGALG00000027056		electron t	yes|up	1
ENSGALG00C	PANX1	pannexin 1	yes|up	1
ENSGALG00C	MSX1	msh homeot	yes|up	1
ENSGALG00C	CYTL1	cytokine 1	yes|up	1
ENSGALG00000042001		hect domai	yes|up	1
ENSGALG00C	ZFP36L1	ZFP36 ring	yes|up	1
ENSGALG00C	PTGR1	prostaglar	yes|down	1
ENSGALG00000047831			yes|up	1
ENSGALG00C	NR4A3	nuclear re	yes|up	1
ENSGALG00C	UNC13D	unc-13 hom	yes|up	1
ENSGALG00C	RIPK2	receptor i	yes|up	1
ENSGALG00000015062		interferor	yes|up	1
ENSGALG00000048310			yes|down	1
ENSGALG00000046156		granulocyt	yes|up	1
ENSGALG00C	NKX2-5	NK2 homeot	yes|up	1
ENSGALG00C	SCX	scleraxis	yes|up	1
ENSGALG00000048488			yes|down	1
ENSGALG00C	GCNT4	glucosamir	yes|up	1
ENSGALG00C	CASZ1	castor zir	yes|up	1
ENSGALG00C	CREG2	cellular r	yes|down	1
ENSGALG00C	RPS6	ribosomal	yes|down	1
ENSGALG00C	SNRK	SNF relate	yes|up	1
ENSGALG00C	GATA3	GATA bindi	yes|up	1
ENSGALG00C	GAP43	growth ass	yes|down	1
ENSGALG00C	GPX4	glutathior	yes|down	1
ENSGALG00C	SPON2	spondin 2	yes|down	1

ENSGALG00C	C11H19orf1	chromosome	yes	up	1
ENSGALG00C	PAG1	phosphopro	yes	down	1
ENSGALG00C	MYL1	myosin, li	yes	down	1
ENSGALG00C	NOTUM	NOTUM, pal	yes	down	1
ENSGALG00000052881		protocadhe	yes	down	1
ENSGALG00C	TRAIP	TRAF inter	yes	up	1
ENSGALG00C	C6orf58	chromosome	yes	up	1
ENSGALG00C	SEMA5A	semaphorin	yes	down	1
ENSGALG00C	TAPBPL	TAP bindin	yes	up	1
ENSGALG00C	GRIK1	glutamate	yes	up	1
ENSGALG00000034697			yes	up	1
ENSGALG00000012847		cystine/gl	yes	down	1
ENSGALG00C	RPS7	ribosomal	yes	down	1
ENSGALG00C	QSOX1	quiescin Q	yes	up	1
ENSGALG00C	CCL4	chemokine	yes	up	1
ENSGALG00C	POLG2	DNA polyme	yes	up	1
ENSGALG00C	HELT	helt bHLH	yes	up	1
ENSGALG00C	KCNE4	potassium	yes	down	1
ENSGALG00C	SMIM5	small inte	yes	down	1
ENSGALG00C	CNR1	cannabinoi	yes	down	1
ENSGALG00C	MPP2	membrane p	yes	down	1
ENSGALG00000053647			yes	down	1
ENSGALG00C	ITGA9	integrin s	yes	down	1
ENSGALG00C	C15orf48	chromosome	yes	up	1
ENSGALG00C	COL3A1	collagen t	yes	down	1
ENSGALG00000054815			yes	down	1
ENSGALG00000051113			yes	up	1
ENSGALG00C	G0S2	G0/G1 swit	yes	up	1
ENSGALG00000013912		vacuolar p	yes	down	1
ENSGALG00000045510		mesothelin	yes	up	1
ENSGALG00000015234			yes	up	1
ENSGALG00000005964		family wit	yes	up	1
ENSGALG00C	CPLX1	complexin	yes	up	1
ENSGALG00C	MMP11	matrix met	yes	down	1
ENSGALG00C	TRIL	TLR4 inter	yes	down	1
ENSGALG00C	BDH2	3-hydroxyb	yes	down	1
ENSGALG00C	ENPP6	ectonucleo	yes	down	1
ENSGALG00C	GABRA4	gamma-amin	yes	up	1
ENSGALG00C	ADAMTS6	ADAM metal	yes	down	1
ENSGALG00000047526		proto-onco	yes	up	1
ENSGALG00C	ARHGAP28	Rho GTPase	yes	up	1
ENSGALG00C	DUSP5	dual speci	yes	up	1
ENSGALG00000051459			yes	down	1
ENSGALG00000052907			yes	down	1
ENSGALG00C	PLEKHA7	pleckstrin	yes	up	1
ENSGALG00C	RFFL	ring finge	yes	up	1
ENSGALG00C	PLK2	polo like	yes	up	1
ENSGALG00C	HTR7	5-hydroxyt	yes	down	1
ENSGALG00C	ALDH7A1	aldehyde d	yes	down	1
ENSGALG00C	FBN2	fibrillin	yes	down	1
ENSGALG00C	EPHA1	EPH recept	yes	up	1

ENSGALG00CHMCN1	hemicentir	yes|down		1
ENSGALG00000004365		yes|down		1
ENSGALG00000051203	MAS-relate	yes|up		1
ENSGALG00CABCG2	ATP bindir	yes|down		1
ENSGALG00CSLC7A4	solute car	yes|down		1
ENSGALG00CTOP1MT	DNA topois	yes|down		1
ENSGALG00CMCL1	BCL2 famil	yes|up		1
ENSGALG00CAQP9	aquaporin	yes|down		1
ENSGALG00000004009	dehydroger	yes|down		1
ENSGALG00CAVP	arginine v	yes|down		1
ENSGALG00000046879		yes|up		1
ENSGALG00CCAV2	caveolin 2	yes|up		1
ENSGALG00CCITED2	Cbp/p300 i	yes|down		1
ENSGALG00000049107		yes|up		1
ENSGALG00CCFAP58	cilia and	yes|down		1
ENSGALG00CZC2HC1C	zinc finge	yes|up		1
ENSGALG00000003149	inositol 1	yes|up		1
ENSGALG00000037029	phosphodie	yes|up		1
ENSGALG00CIL17REL	interleuki	yes|up		1
ENSGALG00CDUSP1	dual speci	yes|up		1
ENSGALG00CSPARC	secreted p	yes|down		1
ENSGALG00000051385		yes|up		1
ENSGALG00000048137		yes|up		1
ENSGALG00CPFKFB3	6-phosphof	yes|up		1
ENSGALG00CIL17D	interleuki	yes|down		1
ENSGALG00CCMTM6	CKLF like	yes|up		1
ENSGALG00CPIEZO2	piezo type	yes|down		1
ENSGALG00CPARP12	poly(ADP-r	yes|up		1
ENSGALG00CADORA1	adenosine	yes|down		1
ENSGALG00CISLR2	immunoglob	yes|up		1
ENSGALG00000049191		yes|up		1
ENSGALG00CABCB10	ATP bindir	yes|down		1
ENSGALG00CRGS11	regulator	yes|down		1
ENSGALG00CPDE4B	phosphodie	yes|up		1
ENSGALG00CSLC13A5	solute car	yes|up		1
ENSGALG00CDMB1	major hist	yes|down		1
ENSGALG00000029012	eva-1 homc	yes|up		1
ENSGALG00000044151	MORN repea	yes|up		1
ENSGALG00000048869	patched dc	yes|up		1
ENSGALG00CBSN	bassoon pr	yes|down		1
ENSGALG00CFNDC1	fibronecti	yes|down		1
ENSGALG00COGN	osteoglyci	yes|down		1
ENSGALG00000033055		yes|up		1
ENSGALG00CTEK	TEK recept	yes|up		1
ENSGALG00CIL8L1	interleuki	yes|up		1
ENSGALG00000049292		yes|up		1
ENSGALG00CCHRDL1	chordin li	yes|down		1
ENSGALG00CNT5C2	5'-nucleot	yes|down		1
ENSGALG00CPHF19	PHD finger	yes|up		1
ENSGALG00CDALRD3	DALR antic	yes|up		1
ENSGALG00CNOS1	nitric oxi	yes|down		1

ENSGALG00C RBPMS2	RNA bindir	yes|down	1
ENSGALG00C ST8SIA6	ST8 alpha-	yes|down	1
ENSGALG00C HIST1H101	Gallus gal	yes|down	1
ENSGALG00C ADRA1A	adrenocept	yes|up	1
ENSGALG00C RPS24	ribosomal	yes|down	1
ENSGALG00000011805	UDP glucur	yes|up	1
ENSGALG00C DSG4	desmoglein	yes|down	1
ENSGALG00C CPED1	cadherin-l	yes|down	1
ENSGALG00C RGN	regucalcir	yes|up	1
ENSGALG00C PYCR1	pyrroline-	yes|down	1
ENSGALG00000020679	pleckstrir	yes|up	1
ENSGALG00000009479	sterile al	yes|up	1
ENSGALG00000054164		yes|down	1
ENSGALG00000050544		yes|up	1
ENSGALG00C TMCC3	transmembr	yes|down	1
ENSGALG00C CYP2D6	cytochrome	yes|down	1
ENSGALG00C PPM1J	protein ph	yes|up	1
ENSGALG00000011324	tubulin al	yes|up	1
ENSGALG00000049589	polymeric	yes|up	1
ENSGALG00C RELT	RELT tumor	yes|up	1
ENSGALG00C SYNDIG1L	synapse di	yes|down	1
ENSGALG00C VRTN	vertebrae	yes|up	1
ENSGALG00C ATF3	activating	yes|up	1
ENSGALG00C UNC5D	unc-5 netr	yes|down	1
ENSGALG00C TTC39A	tetratricc	yes|down	1
ENSGALG00C TMEM56	transmembr	yes|down	1
ENSGALG00C FAM105A	family wit	yes|up	1
ENSGALG00000040269		yes|up	1
ENSGALG00C DOC2B	double C2	yes|down	1
ENSGALG00C CDH8	cadherin 8	yes|down	1
ENSGALG00C SYNGR3	synaptogyr	yes|down	1
ENSGALG00000045015		yes|down	1
ENSGALG00C SLC7A11	solute car	yes|down	1
ENSGALG00C IGFBP7	insulin li	yes|down	1
ENSGALG00C MNX1	motor neur	yes|up	1
ENSGALG00C PKIB	protein ki	yes|up	1
ENSGALG00000050947	interferor	yes|up	1
ENSGALG00C MYH10	myosin, he	yes|down	1
ENSGALG00C RBM14	RNA bindir	yes|up	1
ENSGALG00C RNF19B	ring finge	yes|up	1
ENSGALG00000054784		yes|down	1
ENSGALG00000001028	ring finge	yes|up	1
ENSGALG00C FANCA	Fanconi ar	yes|up	1
ENSGALG00C NCF1C	neutrophil	yes|up	1
ENSGALG00000046959		yes|down	1
ENSGALG00C COBL	cordon-ble	yes|up	1
ENSGALG00C MYOCD	myocardin	yes|down	1
ENSGALG00000040769		yes|up	1
ENSGALG00C LMBR1	limb devel	yes|down	1
ENSGALG00C PLK3	polo like	yes|up	1
ENSGALG00C RAPGEF3	Rap guanir	yes|down	1

ENSGALG00C	CSGALNACT1	chondroiti	yes|up	1
ENSGALG00C	RPS8	ribosomal	yes|down	1
ENSGALG00C	GPNMB	glycoprote	yes|down	1
ENSGALG00C	SRXN1	sulfiredox	yes|up	1
ENSGALG00C	PTPN22	protein ty	yes|up	1
ENSGALG00C	PRKAA2	protein ki	yes|down	1
ENSGALG00C	SH2D4A	SH2 domair	yes|up	1
ENSGALG00C	KCTD16	potassium	yes|down	1
ENSGALG00C	RASGRP3	RAS guanyl	yes|down	1
ENSGALG00C	TTC9	tetratricc	yes|up	1
ENSGALG00C	HTRA1	HtrA serir	yes|down	1
ENSGALG00C	NTF3	neurotroph	yes|up	1
ENSGALG00C	C3orf70	chromosome	yes|down	1
ENSGALG00000053742		GRAM domai	yes|up	1
ENSGALG00C	SLC6A4	solute car	yes|up	1
ENSGALG00C	NRXN1	neurexin 1	yes|down	1
ENSGALG00C	TBXT	T brachyur	yes|up	1
ENSGALG00C	IER5	immediate	yes|up	1
ENSGALG00C	SLC1A4	solute car	yes|down	1
ENSGALG00000050831		fatty acyl	yes|down	1
ENSGALG00C	SH3RF1	SH3 domair	yes|down	1
ENSGALG00000054532			yes|up	1
ENSGALG00C	HAPLN3	hyaluronar	yes|down	1
ENSGALG00C	TNFRSF6B	TNF recept	yes|down	1
ENSGALG00C	AGT	angiotensi	yes|down	1
ENSGALG00C	BCL2A1	BCL2 relat	yes|up	1
ENSGALG00C	KCNG1	potassium	yes|down	1
ENSGALG00C	LHX6	LIM homeot	yes|up	1
ENSGALG00C	LRFN2	leucine ri	yes|down	1
ENSGALG00000006588		leucine ri	yes|down	1
ENSGALG00C	NTM	neurotrimi	yes|up	1
ENSGALG00C	C2H8ORF22	chromosome	yes|down	1
ENSGALG00C	CHST9	carbohydra	yes|up	1
ENSGALG00C	LAMB4	laminin su	yes|up	1
ENSGALG00C	LSP1P1	lymphocyte	yes|down	1
ENSGALG00000053627			yes|down	1
ENSGALG00C	RPL15	ribosomal	yes|down	1
ENSGALG00C	NPR3	natriureti	yes|down	1
ENSGALG00C	CDH6	cadherin 6	yes|up	1
ENSGALG00C	SFRP4	secreted f	yes|up	1
ENSGALG00C	CACNA1C	calcium vc	yes|up	1
ENSGALG00000009298			yes|down	1
ENSGALG00C	IL10RA	interleuki	yes|up	1
ENSGALG00C	MYH1D	myosin, he	yes|down	1
ENSGALG00C	SIRPA	signal-reg	yes|up	1
ENSGALG00C	HOXB2	homeobox E	yes|up	1
ENSGALG00C	CH25H	cholesterc	yes|up	1
ENSGALG00C	LDB3	LIM domair	yes|down	1
ENSGALG00C	GPR137C	G protein-	yes|down	1
ENSGALG00C	MAP3K8	mitogen-ac	yes|up	1
ENSGALG00C	CIT	citron rhc	yes|up	1

ENSGALG00CSALL4	spalt likeyes	up	1
ENSGALG00CBACE1	beta-secreyes	down	1
ENSGALG00CENTPD2	ectonucleoyes	down	1
ENSGALG00000041205	uncharacteyes	up	1
ENSGALG00CFBXO32	F-box protyes	down	1
ENSGALG00CCRMP1	collapsin yes	up	1
ENSGALG00CLGI1	leucine riyes	up	1
ENSGALG00CZDHHC2	zinc fingeyes	down	1
ENSGALG00CNRXN3	neurexin 3yes	up	1
ENSGALG00000045940	nuclear GIyes	up	1
ENSGALG00CRGS3	regulator yes	up	1
ENSGALG00CSERPINB5	serpin famyes	down	1
ENSGALG00CSERPINB11	serpin famyes	down	1
ENSGALG00CANGPT2	angiopoietyes	up	1
ENSGALG00CSH2D4B	SH2 domainyes	down	1
ENSGALG00COTUD4	OTU deubiqyes	up	1
ENSGALG00CTASL	chromosomeyes	up	1
ENSGALG00CGRIN2A	glutamate yes	down	1
ENSGALG00CFHIT	fragile hiyes	down	1
ENSGALG00000046098	ubiquitin-yes	up	1
ENSGALG00CDMD	dystrophinyes	down	1
ENSGALG00CRASSF5	Ras associyes	up	1
ENSGALG00000030112	aspartate yes	down	1
ENSGALG00000047057	yes	up	1
ENSGALG00000035579	yes	up	1
ENSGALG00000007070	yes	down	1
ENSGALG00000042810	yes	down	1
ENSGALG00000049721	yes	down	1
ENSGALG00CPAPPA	pappalysinyes	down	1
ENSGALG00000053860	mycocerosiyes	up	1
ENSGALG00CITGA2	integrin syes	up	1
ENSGALG00CDUOX2	dual oxidayes	down	1
ENSGALG00CMC5R	melanocortyes	down	1
ENSGALG00CSESN2	sestrin-2-yes	down	1
ENSGALG00CCAPSL	calcyphosiyes	up	1
ENSGALG00CGPM6B	glycoproteyes	down	1
ENSGALG00CIL7R	interleukiyes	up	1
ENSGALG00CPDGFB	platelet dyes	up	1
ENSGALG00CRSPO2	R-spondin yes	down	1
ENSGALG00CPLCE1	phospholipyes	down	1
ENSGALG00CPGPEP1	pyroglutamyes	down	1
ENSGALG00CRPL3	ribosomal yes	down	1
ENSGALG00CRPL10A	ribosomal yes	down	1
ENSGALG00000021395	ATP-bindinyes	down	1
ENSGALG00CLRRC15	leucine riyes	up	1
ENSGALG00000012177	NCK associyes	down	1
ENSGALG00000007003	CMP-N-acetyes	down	1
ENSGALG00CTLR7	toll like yes	up	1
ENSGALG00CLITAF	lipopolysayes	up	1
ENSGALG00000042928	retinol deyes	down	1
ENSGALG00CPTX3	pentraxin yes	up	1

ENSGALG00C CAMKV	CaM kinase	yes|down	1
ENSGALG00C LPIN1	lipin 1 [S	yes|down	1
ENSGALG00C MHM2	male hyper	yes|up	1
ENSGALG00C TAFA3	TAFA chemo	yes|up	1
ENSGALG00000046481	uncharacte	yes|up	1
ENSGALG00C OSBPL11	oxysterol	yes|down	1
ENSGALG00C RPL14	ribosomal	yes|down	1
ENSGALG00C CSF2RA	colony sti	yes|up	1
ENSGALG00000052069		yes|down	1
ENSGALG00000012055	anti-Mulle	yes|down	1
ENSGALG00000021884	paramyosin	yes|up	1
ENSGALG00C CMKLR1	chemerin c	yes|down	1
ENSGALG00C SLBP	stem-loop	yes|up	1
ENSGALG00000046412	amine oxic	yes|down	1
ENSGALG00C RYR2	ryanodine	yes|down	1
ENSGALG00C SEMA3E	semaphorin	yes|up	1
ENSGALG00000048672	uncharacte	yes|up	1
ENSGALG00C LEFTY1	left-right	yes|down	1
ENSGALG00000048771	sperm assc	yes|up	1
ENSGALG00C JAK3	Janus kina	yes|up	1
ENSGALG00C SLC8A3	solute car	yes|down	1
ENSGALG00C C1QTNF6	C1q and tu	yes|down	1
ENSGALG00C RNF121	ring finge	yes|up	1
ENSGALG00C NREP	neuronal r	yes|down	1
ENSGALG00C PNOC	prepronoci	yes|up	1
ENSGALG00C PITX2	paired lik	yes|up	1
ENSGALG00C MALL	mal, T-cel	yes|up	1
ENSGALG00C PDE1C	phosphodie	yes|down	1
ENSGALG00C SLCO1C1	solute car	yes|down	1
ENSGALG00000046632	ly6/PLAUR	yes|up	1
ENSGALG00C PHF11	PHD finger	yes|up	1
ENSGALG00000048109	uncharacte	yes|down	1
ENSGALG00C CAV3	caveolin 3	yes|down	1
ENSGALG00C PDZD2	PDZ domain	yes|down	1
ENSGALG00C MOXD1	monooxygen	yes|down	1
ENSGALG00000052780		yes|up	1
ENSGALG00C EFEMP1	EGF contai	yes|down	1
ENSGALG00C PRUNE2	prune homo	yes|down	1
ENSGALG00C BMF	Bcl2 modif	yes|down	1
ENSGALG00C TMEM108	transmembr	yes|down	1
ENSGALG00000002988	phosphogly	yes|down	1
ENSGALG00000048073		yes|up	1
ENSGALG00C COL6A3	collagen t	yes|down	1
ENSGALG00C KRT14	keratin 14	yes|up	1
ENSGALG00C CDO1	cysteine c	yes|down	1
ENSGALG00000043355	cadherin E	yes|up	1
ENSGALG00C CDKL1	cyclin dep	yes|down	1
ENSGALG00C SLC2A6	solute car	yes|up	1
ENSGALG00C OXCT1	3-oxoacid	yes|down	1
ENSGALG00C MYOM1	myomesin 1	yes|down	1
ENSGALG00000031045		yes|up	1

ENSGALG00CFADS6	fatty acic	yes|up	1
ENSGALG00CMST1R	macrophage	yes|up	1
ENSGALG00CH2AFJ	H2A histor	yes|down	1
ENSGALG00CMFAP5	microfibri	yes|down	1
ENSGALG00000048343	carboxyles	yes|down	1
ENSGALG00000043914		yes|down	1
ENSGALG00000045548		yes|down	1
ENSGALG00CTIGD5	tigger tra	yes|down	1
ENSGALG00CPCNX2	pecanex hc	yes|down	1
ENSGALG00000050659		yes|down	1
ENSGALG00CLPAR1	lysophosph	yes|down	1
ENSGALG00CNFKBIZ	NFKB inhit	yes|up	1
ENSGALG00CSMCO4	single-pas	yes|up	1
ENSGALG00CTOX3	TOX high m	yes|up	1
ENSGALG00CENPP2	ectonuclec	yes|down	1
ENSGALG00CGADD45B	growth arr	yes|down	1
ENSGALG00CHTR1A	5-hydroxyt	yes|down	1
ENSGALG00CPRR5	proline ri	yes|up	1
ENSGALG00CADCYAP1	adenylate	yes|up	1
ENSGALG00CALX4	ALX homeob	yes|up	1
ENSGALG00CRBP5	retinol bi	yes|down	1
ENSGALG00CSLC16A9	solute car	yes|down	1
ENSGALG00CPTN	pleiotroph	yes|down	1
ENSGALG00CSLC2A14	solute car	yes|up	1
ENSGALG00CMAP2K6	mitogen-ac	yes|down	1
ENSGALG00CNFKBIA	NFKB inhit	yes|up	1
ENSGALG00CCAV1	caveolin 1	yes|up	1
ENSGALG00CSLC16A7	solute car	yes|down	1
ENSGALG00000051176		yes|down	1
ENSGALG00000001935		yes|up	1
ENSGALG00CIL17C	interleuki	yes|up	1
ENSGALG00CPKP2	plakophili	yes|up	1
ENSGALG00CTMOD4	tropomodul	yes|up	1
ENSGALG00CPROX1	prospero h	yes|up	1
ENSGALG00CMEGF10	multiple E	yes|down	1
ENSGALG00000009792	uncharacte	yes|up	1
ENSGALG00000004078	LON peptic	yes|down	1
ENSGALG00CADA	adenosine	yes|down	1
ENSGALG00CFCHSD1	FCH and dc	yes|down	1
ENSGALG00CC1QTNF12	family wit	yes|up	1
ENSGALG00CFAM46A	family wit	yes|down	1
ENSGALG00CMME	membrane m	yes|up	1
ENSGALG00CPPFIBP1	PPFIA binc	yes|down	1
ENSGALG00CHECW1	HECT, C2 a	yes|down	1
ENSGALG00CARSG	arylsulfat	yes|down	1
ENSGALG00CKCNJ4	Potassium	yes|down	1
ENSGALG00CZFPM2	zinc finge	yes|down	1
ENSGALG00CTMEM184A	transmembr	yes|up	1
ENSGALG00CSLC27A6	solute car	yes|down	1
ENSGALG00CGPD1	glycerol-3	yes|down	1
ENSGALG00CMAP3K6	mitogen-ac	yes|up	1

ENSGALG00000048748		yes|up	1
ENSGALG00CCHRM4	Gallus galyes|down		1
ENSGALG00CIGFBP4	insulin liyes|down		1
ENSGALG00CFGF13	fibroblastyes|down		1
ENSGALG00CACTA1	actin alphyes|down		1
ENSGALG00CBDKRB2	bradykininyes|up		1
ENSGALG00CCITED4	Cbp/p300 iyes|up		1
ENSGALG00CCCK	cholecystcyes|up		1
ENSGALG00000031266		yes|down	1
ENSGALG00CANGPTL1	angiopoietyes|up		1
ENSGALG00CEAF2	ELL associyes|up		1
ENSGALG00000050993		yes|up	1
ENSGALG00000047374		yes|down	1
ENSGALG00000049007		yes|up	1
ENSGALG00CABLIM1	actin bincyes|down		1
ENSGALG00000036964		yes|up	1
ENSGALG00CTRAF1	TNF receptyes|up		1
ENSGALG00CGDF11	growth difyes|up		1
ENSGALG00CRARRES1	retinoic ayes|down		1
ENSGALG00CISM1	isthmin 1 yes|down		1
ENSGALG00000045168	KIAA1551 [yes|up		1
ENSGALG00CATG10	autophagy yes|down		1
ENSGALG00000031866	protein NDyes|down		1
ENSGALG00CCYR61	cysteine ryes|up		1
ENSGALG00CZNFX1	zinc fingeyes|up		1
ENSGALG00CWFDC1	WAP four-cyes|down		1
ENSGALG00CCACNA1H	calcium vcyes|up		1
ENSGALG00CTLL1	tolloid liyes|down		1
ENSGALG00000050538		yes|up	1
ENSGALG00000050026	scavenger yes|up		1
ENSGALG00CGALNT15	polypepticyes|up		1
ENSGALG00CGPR27	G protein-yes|up		1
ENSGALG00000054151		yes|up	1
ENSGALG00CDENND2D	DENN domaiyes|up		1
ENSGALG00CCCNA2	cyclin A2 yes|up		1
ENSGALG00000005180		yes|down	1
ENSGALG00CCTH	cystathioryes|down		1
ENSGALG00000044433		yes|down	1
ENSGALG00000037166	uncharacteyes|up		1
ENSGALG00000011889	protein kiyes|up		1
ENSGALG00CCLIC3	chloride iyes|up		1
ENSGALG00000037160	SMAD familyes|up		1
ENSGALG00CAKAP12	A-kinase ayes|down		1
ENSGALG00CASNS	asparagineyes|down		1
ENSGALG00CCTHRC1	collagen tyes|up		1
ENSGALG00000045842	cell divisyes|up		1
ENSGALG00000030111		yes|up	1
ENSGALG00CSLC1A1	solute caryes|down		1
ENSGALG00CTCF7	transcriptyes|down		1
ENSGALG00CGPR65	G protein-yes|up		1
ENSGALG00CRASSF10	Ras associyes|up		1

ENSGALG00CSPIRE2	spire-type	yes|up	1
ENSGALG00CTHBS1	thrombospc	yes|up	1
ENSGALG00000054636	lymphocyte	yes|up	1
ENSGALG00CC1QTNF8	C1q and tu	yes|down	1
ENSGALG00CMYF6	myogenic f	yes|up	1
ENSGALG00000051258		yes|up	1
ENSGALG00CAVD	avidin [Sc	yes|up	1
ENSGALG00CPTPRQ	protein ty	yes|up	1
ENSGALG00000055127	zinc finge	yes|down	1
ENSGALG00CACSL1	acyl-CoA s	yes|up	1
ENSGALG00CGUCY2C	guanylate	yes|up	1
ENSGALG00CIFI27L2	interferon	yes|up	1
ENSGALG00CHAL	histidine	yes|down	1
ENSGALG00CVEGFA	vascular e	yes|up	1
ENSGALG00CHOPX	HOP homeob	yes|up	1
ENSGALG00000044797		yes|up	1
ENSGALG00CMPV17	MPV17, mit	yes|down	1
ENSGALG00CAPOC3	apolipoprc	yes|down	1
ENSGALG00CMIPOL1	mirror-ima	yes|down	1
ENSGALG00CCDKL2	cyclin dep	yes|up	1
ENSGALG00CPTGDS	prostaglan	yes|up	1
ENSGALG00CCOTL1	coactosin	yes|down	1
ENSGALG00CVSIG4	V-set and	yes|up	1
ENSGALG00CCCDC112	coiled-coi	yes|down	1
ENSGALG00CRAMP2	receptor a	yes|down	1
ENSGALG00000004322	aryl hydrc	yes|up	1
ENSGALG00CNFASC	neurofasci	yes|down	1
ENSGALG00CALPL	alkaline p	yes|down	1
ENSGALG00000032170	lipocalin	yes|up	1
ENSGALG00000016964		yes|up	1
ENSGALG00CSLC7A5	solute car	yes|down	1
ENSGALG00CGCHFR	GTP cycloh	yes|up	1
ENSGALG00000050844		yes|up	1
ENSGALG00CCOL4A2	collagen t	yes|down	1
ENSGALG00000042551	pleckstrin	yes|up	1
ENSGALG00CKCTD17	potassium	yes|down	1
ENSGALG00CMADPRT1	mono(ADP-r	yes|up	1
ENSGALG00CEPB41	erythrocyt	yes|down	1
ENSGALG00CLAMA5	laminin su	yes|up	1
ENSGALG00CTRPC7	transient	yes|down	1
ENSGALG00CSEMA3D	semaphorin	yes|down	1
ENSGALG00000054440		yes|up	1
ENSGALG00000035244	histone cl	yes|down	1
ENSGALG00CIRF6	interferon	yes|up	1
ENSGALG00CADAM8	ADAM metal	yes|up	1
ENSGALG00CTM6SF1	transmembr	yes|down	1
ENSGALG00CEXD1	exonucleas	yes|down	1
ENSGALG00CXIRP1	xin actin	yes|down	1
ENSGALG00000033282		yes|down	1
ENSGALG00CFBLN5	fibulin 5	yes|down	1
ENSGALG00CIL13RA1	interleuki	yes|up	1

ENSGALG00CKCNK17	potassium	yes|down	1
ENSGALG00000054209	regulator	yes|up	1
ENSGALG00CBCO2	beta-carot	yes|up	1
ENSGALG00CFHOD1	formin hom	yes|up	1
ENSGALG00CTNNI2	troponin I	yes|down	1
ENSGALG00CPOPDC2	popeye dom	yes|down	1
ENSGALG00CCDH20	cadherin 2	yes|down	1
ENSGALG00CTLR1B	toll-like	yes|up	1
ENSGALG00CSLC13A4	solute car	yes|down	1
ENSGALG00CSORL1	sortilin r	yes|down	1
ENSGALG00CRAB39A	RAB39A, me	yes|down	1
ENSGALG00000053387		yes|up	1
ENSGALG00CKIF1A	kinesin fa	yes|up	1
ENSGALG00000037687		yes|up	1
ENSGALG00CADAM22	ADAM metal	yes|down	1
ENSGALG00CFGF9	fibroblast	yes|down	1
ENSGALG00CLIPG	lipase G,	yes|up	1
ENSGALG00CC5	complement	yes|up	1
ENSGALG00CPTGER3	prostaglar	yes|down	1
ENSGALG00000041693		yes|up	1
ENSGALG00CBIRC3	baculovira	yes|up	1
ENSGALG00CMMP7	matrix met	yes|up	1
ENSGALG00CTMEM123	transmembr	yes|up	1
ENSGALG00CPTPN5	protein ty	yes|down	1
ENSGALG00CDNAAF9	chromosome	yes|down	1
ENSGALG00CILDR2	immunoglob	yes|up	1
ENSGALG00CPTPRO	protein ty	yes|up	1
ENSGALG00000053645		yes|up	1
ENSGALG00CLPAR6	lysophosph	yes|down	1
ENSGALG00000023546	NLR family	yes|down	1
ENSGALG00000026154	caspase re	yes|down	1
ENSGALG00CHTR2A	5-hydroxyt	yes|up	1
ENSGALG00CFOXO6	forkhead b	yes|down	1
ENSGALG00000026152	guanylate-	yes|up	1
ENSGALG00CMTMR7	myotubular	yes|down	1
ENSGALG00000045534	promyelocy	yes|up	1
ENSGALG00CTNFRSF4	TNF recept	yes|up	1
ENSGALG00CF2RL1	F2R like t	yes|up	1
ENSGALG00000050894		yes|up	1
ENSGALG00CCECR2	CECR2, his	yes|down	1
ENSGALG00000031518	DNA damage	yes|down	1
ENSGALG00CTAGAP	T-cell act	yes|up	1
ENSGALG00CPTPRG	protein ty	yes|down	1
ENSGALG00CFAM171B	family wit	yes|down	1
ENSGALG00CFREM1	FRAS1 rela	yes|down	1
ENSGALG00CKRTCAP3	keratinocy	yes|up	1
ENSGALG00CADGRG2	adhesion G	yes|up	1
ENSGALG00CALDH1L2	aldehyde d	yes|down	1
ENSGALG00CAMER3	APC membra	yes|down	1
ENSGALG00CANGPT1L	angiopoiet	yes|down	1
ENSGALG00000007645	prominin 1	yes|up	1

ENSGALG00CMN1	MN1 proto-	yes|down	1
ENSGALG00CDRAM1	DNA damage	yes|up	1
ENSGALG00CPTK2B	protein ty	yes|up	1
ENSGALG00000002638	serine rac	yes|up	1
ENSGALG00000016196	cystathior	yes|down	1
ENSGALG00000054732		yes|up	1
ENSGALG00CGBX2	gastrulati	yes|up	1
ENSGALG00000002108	envoplakir	yes|up	1
ENSGALG00CRSPO1	R-spondin	yes|up	1
ENSGALG00CBEST4	bestrophir	yes|up	1
ENSGALG00000002102	uncharacte	yes|up	1
ENSGALG00CTPPP3	tubulin pc	yes|down	1
ENSGALG00CDUSP4	dual speci	yes|up	1
ENSGALG00CEGFL6	EGF like c	yes|down	1
ENSGALG00CPTRF	polymerase	yes|down	1
ENSGALG00CCST3	cystatin C	yes|down	1
ENSGALG00CSLC4A10	solute car	yes|down	1
ENSGALG00CLAMB3	laminin su	yes|up	1
ENSGALG00CASIC1	acid sensi	yes|down	1
ENSGALG00000052737	GTPase IMA	yes|up	1
ENSGALG00CCA13	carbonic a	yes|up	1
ENSGALG00CMTHFD2	methylenet	yes|down	1
ENSGALG00000015032	CD274 mole	yes|up	1
ENSGALG00000021399	ATP bindir	yes|down	1
ENSGALG00CMUSK	muscle ass	yes|down	1
ENSGALG00CARHGAP25	Rho GTPase	yes|down	1
ENSGALG00CPIK3CD	phosphatic	yes|up	1
ENSGALG00CLTBP2	latent tra	yes|down	1
ENSGALG00CRGS2	regulator	yes|up	1
ENSGALG00CCRCP	CGRP recep	yes|down	1
ENSGALG00000002193	Rho guanir	yes|down	1
ENSGALG00CSVEP1	complement	yes|down	1
ENSGALG00CCOL4A1	collagen t	yes|down	1
ENSGALG00CECRG4	chromosome	yes|down	1
ENSGALG00CNPM1	nucleophos	yes|down	1
ENSGALG00CSTEAP3	STEAP3 met	yes|up	1
ENSGALG00CRAPGEF4	Rap guanir	yes|down	1
ENSGALG00CPLEKHA2	pleckstrir	yes|down	1
ENSGALG00000004955		yes|up	1
ENSGALG00CCD83	CD83 molec	yes|up	1
ENSGALG00CPIGR	polymeric	yes|up	1
ENSGALG00CNRN1	neuritin 1	yes|down	1
ENSGALG00CDRAM2	DNA damage	yes|down	1
ENSGALG00CCYRIB	family wit	yes|up	1
ENSGALG00000027716	HPS5, biog	yes|up	1
ENSGALG00CPLAAT1	HRAS like	yes|down	1
ENSGALG00CISYNA1	inositol-3	yes|down	1
ENSGALG00CCR1L	complement	yes|up	1
ENSGALG00CRNLS	renalase,	yes|down	1
ENSGALG00CTMOD2	tropomodul	yes|down	1
ENSGALG00CEPHB2	EPH recept	yes|down	1

ENSGALG00000026836	collagen t	yes|down	1
ENSGALG00C MCAM	melanoma c	yes|up	1
ENSGALG00000044313	malignant	yes|up	1
ENSGALG00000048448	PDZ domair	yes|down	1
ENSGALG00C CENPL	centromere	yes|up	1
ENSGALG00000038146	tripartite	yes|up	1
ENSGALG00C HSD17B7	hydroxyste	yes|up	1
ENSGALG00C TJP2	tight junc	yes|up	1
ENSGALG00C FAM46C	family wit	yes|down	1
ENSGALG00C FABP4	fatty acic	yes|down	1
ENSGALG00C CHL1	cell adhes	yes|down	1
ENSGALG00000048444		yes|up	1
ENSGALG00C RUNX1	runt-relat	yes|up	1
ENSGALG00C UST	uronyl 2-s	yes|down	1
ENSGALG00C CPE	carboxyper	yes|down	1
ENSGALG00C HSPA9	heat shock	yes|down	1
ENSGALG00C BAAT	bile acid-	yes|up	1
ENSGALG00C COLEC12	collectin	yes|down	1
ENSGALG00C SOCS3	suppressor	yes|up	1
ENSGALG00000015428	pleckstrir	yes|down	1
ENSGALG00C GHR	growth hor	yes|down	1
ENSGALG00000047689	epidermal	yes|down	1
ENSGALG00000014857		yes|down	1
ENSGALG00C ENOX1	ecto-NOX c	yes|up	1
ENSGALG00C NOS2	nitric oxi	yes|up	1
ENSGALG00000048592		yes|up	1
ENSGALG00C RANBP3L	RAN bindir	yes|down	1
ENSGALG00000047682		yes|up	1
ENSGALG00000052663		yes|down	1
ENSGALG00000003464	tetratricc	yes|up	1
ENSGALG00C IFIT5	interferor	yes|up	1
ENSGALG00C INHBB	inhibin be	yes|up	1
