# Supplementary material for: Asparagine synthetase regulates the proliferation and differentiation of chicken skeletal muscle satellite cells
Source: Anim Biosci. 2024 Aug 26;37(11):1848–62. doi: 10.5713/ab.24.0271 (PMC11541025; doi:10.5713/ab.24.0271)
Supplement: Supplementary file 2 [file ab-24-0271-Supplementary-Dataset-2.pdf]

Supplementary Dataset S2. DEGs which mapped onto KEGG pathways.

<table>
<tr><th>Num</th><th>Pathway id</th><th>Description</th><th>Database</th><th>Ratio_in_study</th><th>Ratio_in_pop</th><th>Rich factor</th><th>Pvalue</th><th>Padjust</th><th>First Category</th><th>Second Category</th><th>Gene_ids</th><th>Gene_names</th></tr>
<tr><td>64</td><td>map04060</td><td>Cytokine-cytokine receptor interaction</td><td>KEGG PATHWAY</td><td>64/1477</td><td>238/14104</td><td>0.26890756</td><td>#####</td><td>1.95E-10</td><td>Environmental Information Processing</td><td>Signaling molecules and interaction</td><td>ENSGALG00000046192|ENSGALG00000008153|ENSGALG00000026098|ENSGALG00000006106|ENSGALG00000006346|ENSGALG00000011668|ENSGALG00000009603|ENSGALG00000010915|ENSGALG00000032717|ENSGALG00000035733|ENSGALG00000028465|ENSGALG00000005759|ENSGALG0000000005457</td><td>;;IL8;TNFRSF6B;CXCL14;IL8L1;;IL6;CCL4;CCR7;IL17C;IFNW1;;;CCL19;IL1B;GDF7;;ACVR1C;;CSF3;BMP4;CCL20;IL7R;;TNFRSF11B;TNFRSF4;IL20RA;RELT;IL10RA;;ACVR2B;CSF1R;GDF11;IL21R;TNFRSF21;CX3CL1;;;CRLF2;IL17RA;;;TNFSF10;CSF2RA;IL17D;;IL15;IL10RB;IL22RA1;IL13RA1;CCL4;;IL2RB;GHR;TNFRSF18;IL11;NGF;IL18R1;IL7;INHBB;;IL2RA;TGFB3</td></tr>
</table>

Num	Pathway id	Description	Database	Ratio_in_study	Ratio_in_pop	Rich factor	Pvalue	Padjust	First Category	Second Category	Gene_ids	Gene_names
108	map05200	Pathways in cancer	KEGG PATHWAY	108/1477	528/14104	0.20454545	#####	6.24E-10	Human Diseases	Cancer: overview	ENSGALG000000028015|ENSGALG00000008933|ENSGALG00000003485|ENSGALG000000007562|ENSGALG00000010133|ENSGALG00000014903|ENSGALG000000012735|ENSGALG00000006480|ENSGALG00000033234|ENSGALG000000027476|ENSGALG00000016866|ENSGALG00000001583|ENSGALG0000002314	HES1;CASP7;CCND3;FGF4;PTCH2;ITGA2;EDN1;TCF7;JAK3;PLEKHG5;FGF14;TRAF1;PTGER3;FGF13;GSTA3;GSTA4;;IL6;;BDKRB1;;CCNA2;RASGRP3;IL2RA;BID;ADCY6;FOS;PDGFA;;;LAMB3;MET;DLL1;PTGER2;LAMB4;CDKN2B;CASP18;CTNNA2;MAPK10;PGF;AGT;;COL4A2;LAMC1;SPI1;WNT5A;IL15;MMP9;CASP8;PRKCB;IL2RB;CSF1R;RET;TGFA;TGFB3;EDNRB;VEGFA;FGF9;STAT2;NFKB2;BDKRB2;BIRC3;;LAMA5;LPAR6;;;IL7R;PDGFB;KITLG;PIK3R1;IL8;;GNAI1;R

Num	Pathway id	Description	Database	Ratio_in_study	Ratio_in_pop	Rich factor	Pvalue	Padjust	First Category	Second Category	Gene_ids	Gene_names
44	map04630	JAK-STAT signaling pathway	KEGG PATHWAY	44/1477	154/14104	0.28571429	#####	2.50E-08	Environmental Information Processing	Signal transduction	ENSGALG000000081 53|ENSGALG00000030661|ENSGALG00000035017|ENSGALG00000009603|ENSGALG00000015062|ENSGALG00000003485|ENSGALG00000007158|ENSGALG00000005759|ENSGALG00000020876|ENSGALG00000006190|ENSGALG00000008185|ENSGALG00000004231|ENSGALG0000000765	;STAT2;;;;CCND3;SOCS1;IFNW1;AOX2;FHL1;AOX1;;STAT1;CSF3;IL6;IL7R;PDGFB;;PIK3R1;MCL1;IL20RA;IL10RA;;JAK3;IL15;IL21R;IL10RB;;PIK3CD;;;CSF2RA;IL17D;CISH;CRLF2;IL22RA1;IL13RA1;SOCS3;PDGFA;IL2RB;GHR;IL11;IL7;IL2RA

Num	Pathway id	Description	Database	Ratio_in_study	Ratio_in_pop	Rich factor	Pvalue	Padjust	First Category	Second Category	Gene_ids	Gene_names
33	map04668	TNF signaling pathway	KEGG PATHWAY	33/1477	96/14104	0.34375	#####	2.88E-08	Environmental Information Processing	Signal transduction	ENSGALG000000008393|ENSGALG00000006785|ENSGALG00000008933|ENSGALG00000028037|ENSGALG00000017186|ENSGALG00000046494|ENSGALG00000005759|ENSGALG00000005257|ENSGALG00000029940|ENSGALG00000031430|ENSGALG00000003003|ENSGALG00000008346|ENSGALG00000001386	CREB3L1;IRF1;CASP7;FOS;BIRC3;CASP10;IFNW1;;IL1B;;CCL20;CASP18;TNFAIP3;EDN1;PIK3R1;MAPK10;CX3CL1;TRAF1;PIK3CD;TRAF3;MAP3K8;IL15;MMP9;IL6;MAP2K6;CASP8;SOCS3;;IL18R1;MMP10;TRAF2;PTGS2;NFKBIA

Num	Pathway id	Description	Database	Ratio_in_study	Ratio_in_pop	Rich factor	Pvalue	Padjust	First Category	Second Category	Gene_ids	Gene_names
62	map05171	Coronavirus disease - COVID-19	KEGG PATHWAY	62/1477	296/14104	0.20945946	#####	4.34E-06	Human Diseases	Infectious disease: viral	ENSGALG000000006179|ENSGALG00000051431|ENSGALG00000012802|ENSGALG00000005922|ENSGALG00000009312|ENSGALG00000028037|ENSGALG00000002583|ENSGALG00000035079|ENSGALG00000041192|ENSGALG00000005759|ENSGALG00000002813|ENSGALG00000001565|ENSGALG00000002994	RPL13;RPS15A;F13A1;RPL5;RPL22L1;FOS;PIK3CD;RPSAP58;IFIH1;IFNW1;RPS10;C5;IL1B;;STAT1;IKBKE;RPL9;CSF3;RPS3A;RPL8;RPS16;RPL27A;PIK3R1;IL8;MAPK10;STAT2;CFD;RPL14;RPL3;;RPS3;TLR7;RPS6;RPS13;TMEM173;RPL15;RPL12;RPL31;RPS24;RPS7;RPL26L1;RPS12;RPL35;RPL10A;TRAF3;RPL4;RPL23;IL6;RPS8;NFKBIA;PRKCB;RPLP0;;RPS23;RPL37;MX1;RPS11;MMP10;EIF2AK2;HBEGF;IL8L1;

Num	Pathway id	Description	Database	Ratio_in_study	Ratio_in_pop	Rich factor	Pvalue	Padjust	First Category	Second Category	Gene_ids	Gene_names
22	map04061	Viral protein interaction with cytokine and cytokine receptor	KEGG PATHWAY	22/1477	66/14104	0.33333333	#####	2.53E-05	Environmental Information Processing	Signaling molecules and interaction	ENSGALG000000046192|ENSGALG00000006346|ENSGALG00000037989|ENSGALG000000003003|ENSGALG00000032717|ENSGALG00000035733|ENSGALG000000028256|ENSGALG00000010915|ENSGALG00000026098|ENSGALG000000013869|ENSGALG00000032260|ENSGALG00000005725|ENSGALG0000002666	;CXCL14;IL10RB;CCL20;CCL4;CCR7;CCL19;IL6;IL8;IL20RA;IL10RA;CSF1R;CX3CL1;TNFSF10;;IL22RA1;CCL4;;IL2RB;IL18R1;IL8L1;IL2RA

Num	Pathway id	Description	Database	Ratio_in_study	Ratio_in_pop	Rich factor	Pvalue	Padjust	First Category	Second Category	Gene_ids	Gene_names
33	map03010	Ribosome	KEGG PATHWAY	33/1477	127/14104	0.25984252	#####	2.68E-05	Genetic Information Processing	Translation	ENSGALG000000061 79|ENSGALG00000044433|ENSGALG00000051431|ENSGALG000000005922|ENSGALG00000009312|ENSGALG00000035079|ENSGALG000000002813|ENSGALG00000000150|ENSGALG00000010077|ENSGALG000000016232|ENSGALG00000026490|ENSGALG00000005948|ENSGALG0000002329	RPL13;;RPS15A;RPL5;RPL22L1;RPSAP58;RPS10;RPL9;RPS3A;RPL8;RPS16;RPL27A;RPLP0;RPL14;RPL3;RPL10A;RPS3;RPS6;RPS13;RPL15;RPL12;RPL31;RPS24;RPS7;RPL26L1;RPS12;RPL35;RPL4;RPS8;RPS23;RPL37;RPS11;RPL23

Num	Pathway id	Description	Database	Ratio_in_study	Ratio_in_pop	Rich factor	Pvalue	Padjust	First Category	Second Category	Gene_ids	Gene_names
28	map04620	Toll-like receptor signaling pathway	KEGG PATHWAY	28/1477	100/14104	0.28	#####	3.28E-05	Organismal Systems	Immune system	ENSGALG000000109 15|ENSGALG000000 32717|ENSGALG0000 0011389|ENSGALG00 00000575 9|ENSGALG0000002 9940|ENSGALG0000 0007651|ENSGALG00 00001429 7|ENSGALG0000001 5062|ENSGALG0000 0008346|ENSGALG00 00001335 6|ENSGALG0000001 4786|ENSGALG0000 0026098|ENSGALG00 00001110	IL6;CCL4;TRAF 3;IFNW1;IL1B;S TAT1;IRF7;;CAS P18;IKBKE;PIK3 R1;IL8;MAPK10 ;TLR5;;TLR7;PIK 3CD;LY96;FOS; MAP3K8;SPP1; MAP2K6;CASP 8;CCL4;;IL8L1;T LR1B;NFKBIA

Num	Pathway id	Description	Database	Ratio_in_study	Ratio_in_pop	Rich factor	Pvalue	Padjust	First Category	Second Category	Gene_ids	Gene_names
28	map04512	ECM-receptor interaction	KEGG PATHWAY	28/1477	103/14104	0.27184466	#####	5.59E-05	Environmental Information Processing	Signaling molecules and interaction	ENSGALG0000000964 1|ENSGALG00000000379|ENSGALG00000003589|ENSGALG00000008439|ENSGALG00000053107|ENSGALG00000009626|ENSGALG00000053028|ENSGALG00000042388|ENSGALG00000039080|ENSGALG00000001343|ENSGALG00000003923|ENSGALG00000003283|ENSGALG00000003679	COL1A2;ITGB3;VTN;CD36;;THBS1;;;CD44;LAMB3;COL6A3;;COL4A1;LAMB4;ITGA2;;THBS2;;;FREM1;COL4A2;COL9A3;LAMC1;LAMA5;SPP1;SDC4;FREM2;ITGA9

Num	Pathway id	Description	Database	Ratio_in_study	Ratio_in_pop	Rich factor	Pvalue	Padjust	First Category	Second Category	Gene_ids	Gene_names
27	map04933	AGE-RAGE signaling pathway in diabetic complications	KEGG PATHWAY	27/1477	99/14104	0.27272727	#####	7.19E-05	Human Diseases	Endocrine and metabolic disease	ENSGALG00000002016|ENSGALG00000009641|ENSGALG00000026687|ENSGALG00000007669|ENSGALG00000053028|ENSGALG00000005257|ENSGALG00000029940|ENSGALG00000007651|ENSGALG00000036798|ENSGALG00000010290|ENSGALG00000010915|ENSGALG00000012735|ENSGALG00000001478	PRKCD;COL1A2;PLCD4;EGR1;;;IL1B;STAT1;COL4A1;VEGFA;IL6;EDN1;PIK3R1;IL8;MAPK10;PLCE1;;AGT;;COL4A2;PIK3CD;;PRKCB;;COL3A1;IL8L1;TGFB3

Num	Pathway id	Description	Database	Ratio_in_study	Ratio_in_pop	Rich factor	Pvalue	Padjust	First Category	Second Category	Gene_ids	Gene_names
30	map04974	Protein digestion and absorption	KEGG PATHWAY	30/1477	120/14104	0.25	#####	0.00012	Organismal Systems	Digestive system	ENSGALG000000009641|ENSGALG00000032220|ENSGALG00000010331|ENSGALG00000034119|ENSGALG00000008544|ENSGALG00000015253|ENSGALG00000053028|ENSGALG00000005797|ENSGALG00000009400|ENSGALG00000006834|ENSGALG00000047719|ENSGALG00000003923|ENSGALG0000000254	COL1A2;ELN;MME;;SLC8A1;COL8A1;;COL20A1;SLC8A3;CPB1;;COL6A3;COL5A1;PRCP;KCNQ1;;SLC1A1;;COL4A2;COL9A3;KCNK5;;COL5A2;;;COL3A1;MEP1A;DPP4;;COL4A1

Num	Pathway id	Description	Database	Ratio_in_study	Ratio_in_pop	Rich factor	Pvalue	Padjust	First Category	Second Category	Gene_ids	Gene_names
26	map05222	Small cell lung cancer	KEGG PATHWAY	26/1477	97/14104	0.26804124	#####	0.00012	Human Diseases	Cancer: specific types	ENSGALG000000007172|ENSGALG00000011389|ENSGALG00000017186|ENSGALG00000053107|ENSGALG00000053028|ENSGALG00000042388|ENSGALG000000002583|ENSGALG00000001343|ENSGALG00000036798|ENSGALG000000007917|ENSGALG00000026137|ENSGALG00000014903|ENSGALG000000001478	FHIT;TRAF3;BIRC3;;;;PIK3CD;LAMB3;COL4A1;LAMB4;CDKN2B;ITGA2;PIK3R1;GADD45G;;TRAF1;;COL4A2;LAMC1;GADD45B;LAMA5;NFKBIA;NOS2;TRAF2;;PTGS2

Num	Pathway id	Description	Database	Ratio_in_study	Ratio_in_pop	Rich factor	Pvalue	Padjust	First Category	Second Category	Gene_ids	Gene_names
24	map04657	IL-17 signaling pathway	KEGG PATHWAY	24/1477	85/14104	0.28235294	#####	0.00012	Organismal Systems	Immune system	ENSGALG000000028037|ENSGALG00000042838|ENSGALG00000054572|ENSGALG00000029940|ENSGALG00000030907|ENSGALG00000010915|ENSGALG00000008346|ENSGALG00000013861|ENSGALG00000013356|ENSGALG00000026098|ENSGALG00000011109|ENSGALG00000043641|ENSGALG0000000300	FOS;TRAF2;;IL1B;CSF3;IL6;CASP18;TNFAIP3;IKBKE;IL8;MAPK10;JUND;CCL20;IL17RA;TRAF3;IL17D;NFKBIA;MMP9;CASP8;;IL17C;MMP10;PTGS2;IL8L1

Num	Pathway id	Description	Database	Ratio_in_study	Ratio_in_pop	Rich factor	Pvalue	Padjust	First Category	Second Category	Gene_ids	Gene_names
24	map04625	C-type lectin receptor signaling pathway	KEGG PATHWAY	24/1477	91/14104	0.26373626	#####	0.00035	Organismal Systems	Immune system	ENSGALG000000020 16|ENSGALG00000030661|ENSGALG00000006785|ENSGALG000000056 53|ENSGALG00000000681|ENSGALG00000003149|ENSGALG000000029 940|ENSGALG00000007651|ENSGALG00000010915|ENSGALG000000083 46|ENSGALG00000006583|ENSGALG00000013356|ENSGALG0000001478	PRKCD;STAT2;IRF1;NFKB2;PAK1;;IL1B;STAT1;IL6;CASP18;LSP1P1;IKBKE;PIK3R1;MAPK10;PIK3CD;ITPR2;;IL17D;EGR3;PLK3;CASP8;PTGS2;;NFKBIA

Num	Pathway id	Description	Database	Ratio_in_study	Ratio_in_pop	Rich factor	Pvalue	Padjust	First Category	Second Category	Gene_ids	Gene_names
20	map04622	RIG-I-like receptor signaling pathway	KEGG PATHWAY	20/1477	69/14104	0.28985507	#####	0.00037	Organismal Systems	Immune system	ENSGALG000000054104|ENSGALG00000041129|ENSGALG00000026098|ENSGALG00000014297|ENSGALG00000027864|ENSGALG00000011668|ENSGALG00000041192|ENSGALG00000015062|ENSGALG00000046160|ENSGALG00000023821|ENSGALG00000011389|ENSGALG00000013356|ENSGALG0000004283	;TMEM173;IL8;IRF7;NFKBIA;IL8L1;IFIH1;;;DHX58;TRAF3;IKBKE;TRAF2;CASP18;CASP10;;IFNW1;;CASP8;MAPK10

Num	Pathway id	Description	Database	Ratio_in_study	Ratio_in_pop	Rich factor	Pvalue	Padjust	First Category	Second Category	Gene_ids	Gene_names
26	map04380	Osteoclast differentiation	KEGG PATHWAY	26/1477	104/14104	0.25	#####	0.00038	Organismal Systems	Development and regeneration	ENSGALG000000014124|ENSGALG00000030661|ENSGALG00000005653|ENSGALG00000028037|ENSGALG00000042838|ENSGALG00000005759|ENSGALG00000007158|ENSGALG00000005725|ENSGALG00000029940|ENSGALG00000007651|ENSGALG00000043167|ENSGALG00000014786|ENSGALG00000001110	TEC;STAT2;NFKB2;FOS;TRAF2;IFNW1;SOCS1;CSF1R;IL1B;STAT1;TNFRSF11B;PIK3R1;MAPK10;;SIRPA;IGSF1;PIK3CD;JUND;SPI1;NCF1C;;ITGB3;MAP2K6;NFKBIA;SOCS3;GAB2

Num	Pathway id	Description	Database	Ratio_in_study	Ratio_in_pop	Rich factor	Pvalue	Padjust	First Category	Second Category	Gene_ids	Gene_names
35	map05164	Influenza A	KEGG PATHWAY	35/1477	167/14104	0.20958084	#####	0.00089	Human Diseases	Infectious disease: viral	ENSGALG00000030661|ENSGALG00000010915|ENSGALG00000016400|ENSGALG00000000162|ENSGALG00000003485|ENSGALG00000005759|ENSGALG00000045534|ENSGALG00000002583|ENSGALG00000030940|ENSGALG00000029940|ENSGALG00000007651|ENSGALG00000014297|ENSGALG00000004119	STAT2;IL6;RSAD2;DMB1;CCND3;IFNW1;;PIK3CD;BLB2;IL1B;STAT1;IRF7;IFIH1;;CASP18;IKBKE;PIK3R1;IL8;EIF2AK2;;TLR7;KPNA7;SOCS3;MX1;TNFSF10;;TRAF3;CASP8;PRKCB;;;IL8L1;;NFKBIA;BID

Num	Pathway id	Description	Database	Ratio_in_study	Ratio_in_pop	Rich factor	Pvalue	Padjust	First Category	Second Category	Gene_ids	Gene_names
72	map04151	PI3K-Akt signaling pathway	KEGG PATHWAY	72/1477	434/14104	0.16589862	#####	0.00092	Environmental Information Processing	Signal transduction	ENSGALG00000025748|ENSGALG00000009641|ENSGALG00000010290|ENSGALG00000008393|ENSGALG00000009603|ENSGALG00000035282|ENSGALG00000003589|ENSGALG00000008141|ENSGALG00000039895|ENSGALG00000053107|ENSGALG00000009626|ENSGALG00000003485|ENSGALG0000000575	FGF9;COL1A2;VEGFA;CREB3L1;;IGF2;VTN;;EPHA2;;THBS1;CCND3;IFNW1;;PDGFB;PIK3AP1;PIK3CD;LAMB3;MET;COL6A3;KITLG;IL7;CSF3;LAMB4;;FOXO6;IL7R;ITGA2;;PIK3R1;THBS2;KDR;;G6PC2;JAK3;;FGF14;RPS6;CSF1R;COL4A2;LPAR1;;PIK3R5;COL9A3;PRKAA2;COL4A1;LAMC1;MCL1;;;LAMA5;FGF4;FGF13;ANGPT2;SPP1;ITGB3;IL6;PGF;;PDGFA;IL2RB;GHR;LPAR6;FGF18;NGF;MYB;ITGA9;;TEK;IL2RA;NTF3;TGFA

Num	Pathway id	Description	Database	Ratio_in_study	Ratio_in_pop	Rich factor	Pvalue	Padjust	First Category	Second Category	Gene_ids	Gene_names
33	map05161	Hepatitis B	KEGG PATHWAY	33/1477	158/14104	0.20886076	#####	0.0014	Human Diseases	Infectious disease: viral	ENSGALG000000016564|ENSGALG00000008393|ENSGALG00000030661|ENSGALG00000010915|ENSGALG00000011389|ENSGALG00000046494|ENSGALG00000005759|ENSGALG00000002583|ENSGALG00000007651|ENSGALG00000014297|ENSGALG00000041192|ENSGALG00000015062|ENSGALG0000000834	PTK2B;CREB3L1;STAT2;IL6;TRAF3;CASP10;IFNW1;PIK3CD;STAT1;IRF7;IFIH1;;CASP18;IKBKE;PIK3R1;IL8;MAPK10;BID;JAK3;FOS;;;MMP9;MAP2K6;CASP8;PRKCB;;IL8L1;CCNA2;;EGR3;NFKBIA;TGFB3

Num	Pathway id	Description	Database	Ratio_in_study	Ratio_in_pop	Rich factor	Pvalue	Padjust	First Category	Second Category	Gene_ids	Gene_names
29	map05418	Fluid shear stress and atherosclerosis	KEGG PATHWAY	29/1477	134/14104	0.21641791	0.0001	0.00182	Human Diseases	Cardiovascular disease	ENSGALG00000012429|ENSGALG00000028037|ENSGALG00000008351|ENSGALG00000005257|ENSGALG00000033295|ENSGALG00000029940|ENSGALG00000014645|ENSGALG00000043287|ENSGALG00000010290|ENSGALG00000012178|ENSGALG00000012735|ENSGALG00000014786|ENSGALG0000001110	BMP4;FOS;CAV3;;CAV2;IL1B;MEF2C;CAV1;VEGFA;PDGFB;EDN1;PIK3R1;MAPK10;KDR;ACVR2B;;PRKAA2;DUSP1;PIK3CD;NCF1C;ITGB3;GSTA3;GSTA4;SDC4;MMP9;PECAM1;MAP2K6;;PDGFA

Num	Pathway id	Description	Database	Ratio_in_study	Ratio_in_pop	Rich factor	Pvalue	Padjust	First Category	Second Category	Gene_ids	Gene_names
25	map05145	Toxoplasmosis	KEGG PATHWAY	25/1477	110/14104	0.22727273	0.0001	0.00224	Human Diseases	Infectious disease: parasitic	ENSGALG00000037989|ENSGALG00000017186|ENSGALG00000053107|ENSGALG00000007158|ENSGALG00000042388|ENSGALG00000030940|ENSGALG00000003163|ENSGALG00000001343|ENSGALG00000007651|ENSGALG00000007917|ENSGALG00000008346|ENSGALG00000011109|ENSGALG00000003226	IL10RB;BIRC3;;SOCS1;;BLB2;GNAO1;LAMB3;STAT1;LAMB4;CASP18;MAPK10;IL10RA;GNAI1;;LAMC1;LY96;LAMA5;MAP2K6;CASP8;DMB1;NOS2;PIK3R5;NFKBIA;TGFB3

Num	Pathway id	Description	Database	Ratio_in_study	Ratio_in_pop	Rich factor	Pvalue	Padjust	First Category	Second Category	Gene_ids	Gene_names
24	map05410	Hypertrophic cardiomyopathy	KEGG PATHWAY	24/1477	105/14104	0.22857143	0.0002	0.0024	Human Diseases	Cardiovascular disease	ENSGALG000000015358|ENSGALG00000016281|ENSGALG00000008544|ENSGALG00000053107|ENSGALG00000051776|ENSGALG00000052907|ENSGALG00000009400|ENSGALG00000042388|ENSGALG00000010812|ENSGALG00000010915|ENSGALG00000048310|ENSGALG00000014903|ENSGALG00000001273	MYH15;DMD;SLC8A1;;;;SLC8A3;;RYR2;IL6;;ITGA2;EDN1;CACNA1C;;TNNC1;AGT;;SGCD;SGCG;ITGB3;PRKAA2;ITGA9;TGFB3

Num	Pathway id	Description	Database	Ratio_in_study	Ratio_in_pop	Rich factor	Pvalue	Padjust	First Category	Second Category	Gene_ids	Gene_names
29	map04210	Apoptosis	KEGG PATHWAY	29/1477	137/14104	0.21167883	0.0002	0.00249	Cellular Processes	Cell growth and death	ENSGALG000000008933|ENSGALG00000028037|ENSGALG00000042838|ENSGALG00000046494|ENSGALG00000006511|ENSGALG00000008444|ENSGALG00000035325|ENSGALG00000003149|ENSGALG00000002583|ENSGALG00000008346|ENSGALG00000014786|ENSGALG00000011324|ENSGALG00000001110	CASP7;FOS;TRAF2;CASP10;BCL2A1;TUBAL3;PMAIP1;;PIK3CD;CASP18;PIK3R1;;MAPK10;MCL1;GADD45G;TRAF1;;;CTSS;ITPR2;TUBA3E;TNFSF10;;GADD45B;CASP8;NGF;BIRC3;NFKBIA;BID

Num	Pathway id	Description	Database	Ratio_in_study	Ratio_in_pop	Rich factor	Pvalue	Padjust	First Category	Second Category	Gene_ids	Gene_names
42	map04510	Focal adhesion	KEGG PATHWAY	42/1477	227/14104	0.18502203	0.0002	0.0025	Cellular Processes	Cellular community - eukaryotes	ENSGALG00000007917|ENSGALG00000012178|ENSGALG00000000681|ENSGALG00000003589|ENSGALG00000009641|ENSGALG00000017186|ENSGALG00000008351|ENSGALG00000009626|ENSGALG00000003485|ENSGALG00000053028|ENSGALG00000042388|ENSGALG00000033295|ENSGALG0000000258	LAMB4;PDGFB;PAK1;VTN;COL1A2;BIRC3;CAV3;THBS1;CCND3;;;CAV2;PIK3CD;LAMB3;MET;COL6A3;;COL4A1;VEGFA;ITGA2;;PIK3R1;CAV1;MAPK10;THBS2;PGF;;;COL4A2;COL9A3;LAMC1;KDR;LAMA5;;SPP1;ITGB3;PPP1R12B;PRKCB;PDGFA;ITGA9;;

Num	Pathway id	Description	Database	Ratio_in_study	Ratio_in_pop	Rich factor	Pvalue	Padjust	First Category	Second Category	Gene_ids	Gene_names
61	map04080	Neuroactive ligand-receptor interaction	KEGG PATHWAY	61/1477	369/14104	0.16531165	0.0002	0.00268	Environmental Information Processing	Signaling molecules and interaction	ENSGALG000000016600|ENSGALG00000005223|ENSGALG00000016912|ENSGALG00000026902|ENSGALG00000038728|ENSGALG00000027415|ENSGALG00000023581|ENSGALG00000009603|ENSGALG00000011080|ENSGALG00000008365|ENSGALG00000014202|ENSGALG00000016744|ENSGALG0000000046	POMC;ADCYAP1R1;EDNRB;P2RX6;;GRIN2C;UTS2R;;BDKRB2;CHRM4;GABRA4;GABRA5;ADRA1A;C5;ADRB2;GRM3;AVPR1B;HTR7;MC5R;LPAR6;P2RY4;GRIA4;HTR2A;VIP;CCK;EDN1;GRIN2A;GCG;GHR;F2RL1;P2RY1;GRIK1;AVP;AGT;PTGFR;GABRG3;AVPR2;AGTR2;SSTR2;EDN2;HTR1A;GLP2R;LPAR1;ADCYAP1;GRIA1;PTGER3;P2RX7;ADORA2B;ADORA1;;BDKRB1;HTR2B;ADRA2A;CYSLTR2;CNR1;NPY2R;TRH;;PTAFR;;PTGER2

Num	Pathway id	Description	Database	Ratio_in_study	Ratio_in_pop	Rich factor	Pvalue	Padjust	First Category	Second Category	Gene_ids	Gene_names
16	map05144	Malaria	KEGG PATHWAY	16/1477	58/14104	0.27586207	0.0002	0.00275	Human Diseases	Infectious disease: parasitic	ENSGALG000000029940|ENSGALG00000036883|ENSGALG00000023950|ENSGALG00000003283|ENSGALG00000030907|ENSGALG00000046125|ENSGALG00000010915|ENSGALG00000046160|ENSGALG00000008439|ENSGALG00000026098|ENSGALG00000009626|ENSGALG00000011200|ENSGALG00000003143	IL1B;MET;CR1L;;CSF3;PECAM1;IL6;;CD36;IL8;THBS1;THBS2;;IL8L1;;TGFB3

Num	Pathway id	Description	Database	Ratio_in_study	Ratio_in_pop	Rich factor	Pvalue	Padjust	First Category	Second Category	Gene_ids	Gene_names
27	map05162	Measles	KEGG PATHWAY	27/1477	128/14104	0.2109375	0.0003	0.00344	Human Diseases	Infectious disease: viral	ENSGALG000000306 61|ENSGALG00000010915|ENSGALG00000011389|ENSGALG000000348 5|ENSGALG00000005759|ENSGALG00000029940|ENSGALG000000765 1|ENSGALG00000014297|ENSGALG00000041192|ENSGALG000001506 2|ENSGALG00000008346|ENSGALG00000013861|ENSGALG0000001335	STAT2;IL6;TRAF3;CCND3;IFNW1;IL1B;STAT1;IRF7;IFIH1;;CASP18;TNFAIP3;IKBKE;PIK3R1;MAPK10;;JAK3;EIF2AK2;TLR7;PIK3CD;FOS;NFKBIA;CASP8;IL2RB;MX1;IL2RA;BID

Num	Pathway id	Description	Database	Ratio_in_study	Ratio_in_pop	Rich factor	Pvalue	Padjust	First Category	Second Category	Gene_ids	Gene_names
41	map04015	Rap1 signaling pathway	KEGG PATHWAY	41/1477	225/14104	0.18222222	0.0003	0.00354	Environmental Information Processing	Signal transduction	ENSGALG000000031244|ENSGALG00000009480|ENSGALG00000025748|ENSGALG000000007278|ENSGALG00000009626|ENSGALG00000039895|ENSGALG000000015729|ENSGALG00000003163|ENSGALG00000002583|ENSGALG000000036883|ENSGALG00000010290|ENSGALG00000043694|ENSGALG0000001217	ADCY6;DOCK4;FGF9;GRIN2A;THBS1;EPHA2;LPAR1;GNAO1;PIK3CD;MET;VEGFA;RAPGEF4;PDGFB;KITLG;PIK3R1;SIPA1L2;PLCE1;PGF;P2RY1;FGF14;CSF1R;ID1;GNAI1;PARD6B;KDR;FGF4;FGF13;ADORA2B;ITGB3;RAPGEF3;MAP2K6;PRKCB;PDGFA;CNR1;FGF18;NGF;RASGRP3;;RASSF5;TEK;ANGPT2

Num	Pathway id	Description	Database	Ratio_in_study	Ratio_in_pop	Rich factor	Pvalue	Padjust	First Category	Second Category	Gene_ids	Gene_names
50	map04010	MAPK signaling pathway	KEGG PATHWAY	50/1477	293/14104	0.17064846	0.0003	0.00394	Environmental Information Processing	Signal transduction	ENSGALG00000025748|ENSGALG00000011419|ENSGALG00000030706|ENSGALG00000005653|ENSGALG00000028037|ENSGALG00000000681|ENSGALG00000042838|ENSGALG00000006368|ENSGALG00000039895|ENSGALG00000032618|ENSGALG00000005884|ENSGALG00000011207|ENSGALG00000002994	FGF9;DUSP4;MAP3K6;NFKB2;FOS;PAK1;TRAF2;PTPN5;EPHA2;DUSP5;;DUSP6;IL1B;MEF2C;VEGFA;PDGFB;KITLG;MAPK10;CACNA1C;KDR;RASGRF2;NGF;GADD45G;MAP3K8;CSF1R;;JUND;FGF4;;GADD45B;DUSP1;PGF;FGF14;IGF2;FGF13;;MET;CACNA1H;;MAP2K6;PRKCB;CACNA1E;PDGFA;FGF18;ANGPT2;RASGRP3;TGFB3;TEK;NTF3;TGFA

Num	Pathway id	Description	Database	Ratio_in_study	Ratio_in_pop	Rich factor	Pvalue	Padjust	First Category	Second Category	Gene_ids	Gene_names
31	map04621	NOD-like receptor signaling pathway	KEGG PATHWAY	31/1477	162/14104	0.19135802	0.0007	0.0072	Organismal Systems	Immune system	ENSGALG00000002016|ENSGALG00000030661|ENSGALG00000011389|ENSGALG00000017186|ENSGALG00000005759|ENSGALG00000026152|ENSGALG00000000720|ENSGALG00000003149|ENSGALG00000029940|ENSGALG00000015062|ENSGALG00000007651|ENSGALG00000014297|ENSGALG0000004353	PRKCD;STAT2;TRAF3;BIRC3;IFNW1;;;;IL1B;;STAT1;IRF7;RIPK2;IL6;CASP18;;TNFAIP3;IKBKE;IL8;MAPK10;;TMEM173;PANX1;ITPR2;;P2RX7;CASP8;;IL8L1;TRAF2;NFKBIA
5	map00430	Taurine and hypotaurine metabolism	KEGG PATHWAY	5/1477	9/14104	0.55555556	0.0011	0.01176	Metabolism	Metabolism of other amino acids	ENSGALG00000001062|ENSGALG00000002358|ENSGALG00000040504|ENSGALG00000038593|ENSGALG00000040619	GGT7;CDO1;GGT5;;BAAT

Num	Pathway id	Description	Database	Ratio_in_study	Ratio_in_pop	Rich factor	Pvalue	Padjust	First Category	Second Category	Gene_ids	Gene_names
16	map04924	Renin secretion	KEGG PATHWAY	16/1477	69/14104	0.23188406	0.0017	0.01771	Organismal Systems	Endocrine system	ENSGALG000000031244|ENSGALG00000003149|ENSGALG00000011117|ENSGALG00000008382|ENSGALG00000014858|ENSGALG00000037869|ENSGALG00000012396|ENSGALG00000005223|ENSGALG00000039935|ENSGALG00000004574|ENSGALG00000012735|ENSGALG00000014071|ENSGALG00000004003	ADCY6;;AGT;GNAI1;ADCYAP1;;PTGER2;ADCYAP1R1;PDE1C;NPPA;EDN1;ITPR2;ADRB2;EDN2;ADORA1;CACNA1C

Num	Pathway id	Description	Database	Ratio_in_study	Ratio_in_pop	Rich factor	Pvalue	Padjust	First Category	Second Category	Gene_ids	Gene_names
18	map05412	Arrhythmogenic right ventricular cardiomyopathy	KEGG PATHWAY	18/1477	82/14104	0.2195122	0.0018	0.01775	Human Diseases	Cardiovascular disease	ENSGALG00000000379|ENSGALG00000036964|ENSGALG00000015142|ENSGALG00000010812|ENSGALG00000003886|ENSGALG00000017122|ENSGALG00000015132|ENSGALG00000014903|ENSGALG00000012913|ENSGALG00000008544|ENSGALG00000027853|ENSGALG00000053107|ENSGALG0000001302	ITGB3;;DSG2;RYR2;SGCD;SGCG;CDH2;ITGA2;PKP2;SLC8A1;CTNNA2;;CACNA1C;ITGA9;;DMD;SLC8A3;TCF7

Num	Pathway id	Description	Database	Ratio_in_study	Ratio_in_pop	Rich factor	Pvalue	Padjust	First Category	Second Category	Gene_ids	Gene_names
55	map05165	Human papillomavirus infection	KEGG PATHWAY	55/1477	355/14104	0.15492958	0.0019	0.01873	Human Diseases	Infectious disease: viral	ENSGALG000000028015|ENSGALG00000010290|ENSGALG00000008393|ENSGALG00000030661|ENSGALG00000006785|ENSGALG00000011389|ENSGALG000000003589|ENSGALG00000009641|ENSGALG00000053107|ENSGALG000000009626|ENSGALG00000003485|ENSGALG00000005759|ENSGALG0000004238	HES1;VEGFA;CREB3L1;STAT2;IRF1;TRAF3;VTN;COL1A2;;THBS1;CCND3;IFNW1;;PIK3CD;LAMB3;STAT1;;ATP6V0E2;COL4A1;LAMB4;;CASP18;ITGA2;IKBKE;PIK3R1;TCF7;THBS2;CCNA2;;HES4;;;;WNT16;COL4A2;COL9A3;LAMC1;PARD6B;MX1;;LAMA5;HEY1;OASL;WNT5A;;SPP1;ITGB3;COL6A3;CASP8;SLC9A3R1;ITGA9;ATP6V0D2;WNT11B;EIF2AK2;PTGS2

Num	Pathway id	Description	Database	Ratio_in_study	Ratio_in_pop	Rich factor	Pvalue	Padjust	First Category	Second Category	Gene_ids	Gene_names
7	map00532	Glycosaminoglycan biosynthesis - chondroitin sulfate / dermatan sulfate	KEGG PATHWAY	7/1477	19/14104	0.36842105	0.0022	0.01925	Metabolism	Glycan biosynthesis and metabolism	ENSGALG00000012345|ENSGALG00000010125|ENSGALG00000002543|ENSGALG00000009704|ENSGALG00000029632|ENSGALG00000030607|ENSGALG00000006757	UST;CSGALNACT1;CSGALNACT2;CHST15;CHST13;CHST11;XYLT1

Num	Pathway id	Description	Database	Ratio_in_study	Ratio_in_pop	Rich factor	Pvalue	Padjust	First Category	Second Category	Gene_ids	Gene_names
21	map04928	Parathyroid hormone synthesis, secretion and action	KEGG PATHWAY	21/1477	103/14104	0.2038835	0.002	0.01944	Organismal Systems	Endocrine system	ENSGALG000000031244|ENSGALG00000003149|ENSGALG00000037943|ENSGALG00000014645|ENSGALG00000008382|ENSGALG00000007650|ENSGALG00000008393|ENSGALG00000003670|ENSGALG00000033090|ENSGALG00000007669|ENSGALG00000027565|ENSGALG00000037029|ENSGALG0000002803	ADCY6;;PRKCB;MEF2C;GNAI1;SLC9A3R1;CREB3L1;MAFB;VDR;EGR1;;;FOS;MMP16;PDE4B;ITPR2;JUND;NR4A2;HBEGF;GATA3;CYP24A1

Num	Pathway id	Description	Database	Ratio_in_study	Ratio_in_pop	Rich factor	Pvalue	Padjust	First Category	Second Category	Gene_ids	Gene_names
38	map05163	Human cytomegalovirus infection	KEGG PATHWAY	38/1477	226/14104	0.16814159	0.0022	0.01974	Human Diseases	Infectious disease: viral	ENSGALG000000031244|ENSGALG00000037943|ENSGALG00000008393|ENSGALG00000037989|ENSGALG00000010915|ENSGALG00000032717|ENSGALG00000042838|ENSGALG00000005759|ENSGALG00000003163|ENSGALG00000029940|ENSGALG00000008382|ENSGALG00000012396|ENSGALG0000001029	ADCY6;PRKCB;CREB3L1;IL10RB;IL6;CCL4;TRAF2;IFNW1;GNAO1;IL1B;GNAI1;PTGER2;VEGFA;;;CASP18;PIK3R1;IL8;CX3CL1;IL10RA;;TMEM173;TAP1;;PIK3CD;ITPR2;PTGER3;ITGB3;MAP2K6;CASP8;PTK2B;CCL4;;IL8L1;;PTGS2;NFKBIA;BID

Num	Pathway id	Description	Database	Ratio_in_study	Ratio_in_pop	Rich factor	Pvalue	Padjust	First Category	Second Category	Gene_ids	Gene_names
14	map05134	Legionellosis	KEGG PATHWAY	14/1477	58/14104	0.24137931	0.0022	0.02005	Human Diseases	Infectious disease: bacterial	ENSGALG000000029940|ENSGALG00000023950|ENSGALG00000027864|ENSGALG000000005843|ENSGALG00000008933|ENSGALG00000010915|ENSGALG000000008346|ENSGALG00000013037|ENSGALG00000046160|ENSGALG000000026098|ENSGALG00000005653|ENSGALG00000009392|ENSGALG000000000835	IL1B;CR1L;NFKBIA;EEF1A2;CASP7;IL6;CASP18;BCL2L13;;IL8;NFKB2;TLR5;CASP8;IL8L1

Num	Pathway id	Description	Database	Ratio_in_study	Ratio_in_pop	Rich factor	Pvalue	Padjust	First Category	Second Category	Gene_ids	Gene_names
23	map04926	Relaxin signaling pathway	KEGG PATHWAY	23/1477	119/14104	0.19327731	0.0027	0.02282	Organismal Systems	Endocrine system	ENSGALG000000031244|ENSGALG00000009641|ENSGALG00000016912|ENSGALG00000008393|ENSGALG00000008177|ENSGALG00000028037|ENSGALG00000053028|ENSGALG00000003163|ENSGALG00000002583|ENSGALG00000036798|ENSGALG00000010290|ENSGALG00000012735|ENSGALG0000001478	ADCY6;COL1A2;EDNRB;CREB3L1;NOS1;FOS;;GNAO1;PIK3CD;COL4A1;VEGFA;EDN1;PIK3R1;MAPK10;;GNAI1;COL4A2;;;MMP9;NFKBIA;NOS2;COL3A1

Num	Pathway id	Description	Database	Ratio_in_study	Ratio_in_pop	Rich factor	Pvalue	Padjust	First Category	Second Category	Gene_ids	Gene_names
38	map04014	Ras signaling pathway	KEGG PATHWAY	38/1477	234/14104	0.16239316	0.0041	0.03375	Environmental Information Processing	Signal transduction	ENSGALG000000025748|ENSGALG00000007278|ENSGALG00000035017|ENSGALG00000035282|ENSGALG00000000681|ENSGALG00000002556|ENSGALG00000054619|ENSGALG00000039895|ENSGALG00000040549|ENSGALG00000036883|ENSGALG00000010290|ENSGALG00000012178|ENSGALG0000001120	FGF9;GRIN2A;;IGF2;PAK1;NGF;GAB2;EPHA2;HTR7;MET;VEGFA;PDGFB;KITLG;PIK3R1;RASAL2;PLCE1;PGF;RASGRF2;MAPK10;FGF14;CSF1R;;PIK3CD;KDR;;FGF4;FGF13;RASSF1;PRKCB;PDGFA;REL;FGF18;ANGPT2;RASGRP3;RASSF5;TEK;NTF3;TGFA

Num	Pathway id	Description	Database	Ratio_in_study	Ratio_in_pop	Rich factor	Pvalue	Padjust	First Category	Second Category	Gene_ids	Gene_names
31	map04360	Axon guidance	KEGG PATHWAY	31/1477	183/14104	0.16939891	0.0048	0.03859	Organismal Systems	Development and regeneration	ENSGALG00000055081|ENSGALG00000008297|ENSGALG00000038225|ENSGALG00000000681|ENSGALG00000006473|ENSGALG00000039895|ENSGALG00000002583|ENSGALG00000047554|ENSGALG00000036883|ENSGALG00000011406|ENSGALG00000014687|ENSGALG00000003677|ENSGALG0000001559	SEMA4C;SEMA4B;SEMA3E;PAK1;PLXNA4;EPHA2;PIK3CD;GDF7;MET;NTN4;EPHA1;NTNG2;EPHA7;PIK3R1;SEMA5A;UNC5D;ABLIM1;GNAI1;EPHB2;PARD6B;;SEMA3D;SEMA3A;WNT5A;UNC5A;EPHB1;EFNB2;;SLIT3;RGS3;TRPC4

Num	Pathway id	Description	Database	Ratio_in_study	Ratio_in_pop	Rich factor	Pvalue	Padjust	First Category	Second Category	Gene_ids	Gene_names
32	map05167	Kaposi sarcoma-associated herpesvirus infection	KEGG PATHWAY	32/1477	192/14104	0.16666667	0.0054	0.04245	Human Diseases	Infectious disease: viral	ENSGALG000000030661|ENSGALG00000011389|ENSGALG00000042838|ENSGALG000000005759|ENSGALG00000003149|ENSGALG00000002583|ENSGALG000000007651|ENSGALG00000014297|ENSGALG00000010290|ENSGALG000000015062|ENSGALG00000008346|ENSGALG00000012178|ENSGALG0000000001335	STAT2;TRAF3;TRAF2;IFNW1;;PIK3CD;STAT1;IRF7;VEGFA;;CASP18;PDGFB;IKBKE;PIK3R1;IL8;MAPK10;;TCF7;ITPR2;FOS;IL6;MAP2K6;CASP8;;IL8L1;PIK3R5;EIF2AK2;;PTGS2;NFKBIA;ANGPT2;BID

Num	Pathway id	Description	Database	Ratio_in_study	Ratio_in_pop	Rich factor	Pvalue	Padjust	First Category	Second Category	Gene_ids	Gene_names
32	map05207	Chemical carcinogenesis - receptor activation	KEGG PATHWAY	32/1477	192/14104	0.16666667	0.0054	0.04245	Human Diseases	Cancer: overview	ENSGALG00000031244|ENSGALG00000025748|ENSGALG00000008393|ENSGALG00000003485|ENSGALG00000028037|ENSGALG00000040035|ENSGALG00000011805|ENSGALG00000002583|ENSGALG00000008382|ENSGALG00000011182|ENSGALG00000004436|ENSGALG00000010290|ENSGALG00000003713	ADCY6;FGF9;CREB3L1;CCND3;FOS;ADRB2;;PIK3CD;GNAI1;DLL1;CYP3A4;VEGFA;;IKBKE;PIK3R1;;CACNA1C;FGF14;KPNA7;VDR;PAQR8;EPHX4;CYP1B1;FGF4;FGF13;GSTA3;GSTA4;AR;;PRKCB;CDC6;FGF18

Num	Pathway id	Description	Database	Ratio_in_study	Ratio_in_pop	Rich factor	Pvalue	Padjust	First Category	Second Category	Gene_ids	Gene_names
18	map04713	Circadian entrainment	KEGG PATHWAY	18/1477	92/14104	0.19565217	0.0065	0.04894	Organismal Systems	Environmental adaptation	ENSGALG000000005215|ENSGALG00000031244|ENSGALG00000003163|ENSGALG00000038995|ENSGALG00000037943|ENSGALG00000008382|ENSGALG00000010812|ENSGALG00000007278|ENSGALG00000027415|ENSGALG00000005223|ENSGALG00000008177|ENSGALG00000004860|ENSGALG0000002803	CACNA1H;ADCY6;GNAO1;GRIA4;PRKCB;GNAI1;RYR2;GRIN2A;GRIN2C;ADCYAP1R1;NOS1;RASD1;FOS;KCNJ5;;CACNA1C;GRIA1;ADCYAP1

Num	Pathway id	Description	Database	Ratio_in_study	Ratio_in_pop	Rich factor	Pvalue	Padjust	First Category	Second Category	Gene_ids	Gene_names
15	map04115	p53 signaling pathway	KEGG PATHWAY	15/1477	72/14104	0.20833333	0.0069	0.05067	Cellular Processes	Cell growth and death	ENSGALG00000028005|ENSGALG00000011619|ENSGALG00000016442|ENSGALG00000037603|ENSGALG00000008355|ENSGALG00000012117|ENSGALG00000008346|ENSGALG00000029968|ENSGALG00000009626|ENSGALG00000003485|ENSGALG00000027207|ENSGALG00000006407|ENSGALG0000001303	GADD45G;SIVA1;RRM2;SESN2;CASP8;STEAP3;CASP18;GADD45B;THBS1;CCND3;PERP2;;BID;SERPINB5;PMAIP1

Num	Pathway id	Description	Database	Ratio_in_study	Ratio_in_pop	Rich factor	Pvalue	Padjust	First Category	Second Category	Gene_ids	Gene_names
13	map04923	Regulation of lipolysis in adipocytes	KEGG PATHWAY	13/1477	59/14104	0.22033898	0.0071	0.051	Organismal Systems	Endocrine system	ENSGALG000000031244|ENSGALG00000033635|ENSGALG00000002583|ENSGALG000000000168|ENSGALG00000008382|ENSGALG00000037869|ENSGALG000000004574|ENSGALG00000001314|ENSGALG00000030025|ENSGALG000000014786|ENSGALG00000040035|ENSGALG00000023142|ENSGALG000000003273	ADCY6;PTGS2;PIK3CD;ADORA1;GNAI1;;NPPA;PTGS1;FABP4;PIK3R1;ADRB2;PTGER3;MGLL

Num	Pathway id	Description	Database	Ratio_in_study	Ratio_in_pop	Rich factor	Pvalue	Padjust	First Category	Second Category	Gene_ids	Gene_names
36	map04024	cAMP signaling pathway	KEGG PATHWAY	36/1477	227/14104	0.15859031	0.0074	0.05239	Environmental Information Processing	Signal transduction	ENSGALG000000031244|ENSGALG00000016600|ENSGALG00000038995|ENSGALG00000008393|ENSGALG00000007278|ENSGALG00000027415|ENSGALG00000027565|ENSGALG00000023142|ENSGALG00000000681|ENSGALG00000040035|ENSGALG00000043694|ENSGALG00000002583|ENSGALG00000001081	ADCY6;POMC;GRIA4;CREB3L1;GRIN2A;GRIN2C;;PTGER3;PAK1;ADRB2;RAPGEF4;PIK3CD;RYR2;PTGER2;ADCYAP1R1;VIP;NPPA;EDN1;MAPK10;CACNA1C;SSTR2;RAPGEF3;GNAI1;PIK3R1;;PDE4B;GCG;HTR1A;GRIA1;FOS;ADORA1;NFKBIA;ADCYAP1;EDN2;;PLCE1

Num	Pathway id	Description	Database	Ratio_in_study	Ratio_in_pop	Rich factor	Pvalue	Padjust	First Category	Second Category	Gene_ids	Gene_names
20	map04726	Serotonergic synapse	KEGG PATHWAY	20/1477	108/14104	0.18518519	0.008	0.05535	Organismal Systems	Nervous system	ENSGALG000000040360|ENSGALG00000001181|ENSGALG00000003163|ENSGALG00000004246|ENSGALG00000006236|ENSGALG00000008382|ENSGALG00000031244|ENSGALG00000014742|ENSGALG00000037943|ENSGALG00000016992|ENSGALG00000007681|ENSGALG00000001314|ENSGALG0000000003713	DUSP1;KCNJ5;GNAO1;SLC6A4;TPH1;GNAI1;ADCY6;HTR1A;PRKCB;HTR2A;HTR2B;PTGS1;;CYP2D6;;ITPR2;CACNA1C;RAPGEF3;PTGS2;HTR7

Num	Pathway id	Description	Database	Ratio_in_study	Ratio_in_pop	Rich factor	Pvalue	Padjust	First Category	Second Category	Gene_ids	Gene_names
14	map05133	Pertussis	KEGG PATHWAY	14/1477	67/14104	0.20895522	0.0086	0.05834	Human Diseases	Infectious disease: bacterial	ENSGALG000000029940|ENSGALG00000008382|ENSGALG00000038096|ENSGALG000000008933|ENSGALG00000010915|ENSGALG00000001565|ENSGALG000000028037|ENSGALG00000026098|ENSGALG00000046160|ENSGALG000000032701|ENSGALG00000002503|ENSGALG00000006785|ENSGALG0000001166	IL1B;GNAI1;NOS2;CASP7;IL6;C5;FOS;IL8;;LY96;SFTPA2;IRF1;IL8L1;MAPK10

Num	Pathway id	Description	Database	Ratio_in_study	Ratio_in_pop	Rich factor	Pvalue	Padjust	First Category	Second Category	Gene_ids	Gene_names
28	map04062	Chemokine signaling pathway	KEGG PATHWAY	28/1477	168/14104	0.16666667	0.0088	0.05845	Organismal Systems	Immune system	ENSGALG00000002016|ENSGALG00000031244|ENSGALG00000037943|ENSGALG00000046192|ENSGALG00000006346|ENSGALG00000030661|ENSGALG00000034478|ENSGALG00000000681|ENSGALG00000032717|ENSGALG00000035733|ENSGALG00000028256|ENSGALG00000002583|ENSGALG00000000765	PRKCD;ADCY6;PRKCB;;CXCL14;STAT2;CCL4;PAK1;CCL4;CCR7;CCL19;PIK3CD;STAT1;CCL20;FOXO6;PIK3R1;IL8;CX3CL1;JAK3;GNAI1;NCF1C;;NFKBIA;PTK2B;DOCK2;;PIK3R5;IL8L1

Num	Pathway id	Description	Database	Ratio_in_study	Ratio_in_pop	Rich factor	Pvalue	Padjust	First Category	Second Category	Gene_ids	Gene_names
15	map05223	Non-small cell lung cancer	KEGG PATHWAY	15/1477	75/14104	0.2	0.0101	0.06429	Human Diseases	Cancer: specific types	ENSGALG00000028005|ENSGALG00000002583|ENSGALG00000036883|ENSGALG00000007172|ENSGALG00000037943|ENSGALG00000012462|ENSGALG00000026153|ENSGALG00000029968|ENSGALG00000014786|ENSGALG00000002555|ENSGALG00000038943|ENSGALG00000038951|ENSGALG00000005137	GADD45G;PIK3CD;MET;FHIT;PRKCB;KIF5C;FOXO6;GADD45B;PIK3R1;RET;RASSF5;RASSF1;;JAK3;TGFA

Num	Pathway id	Description	Database	Ratio_in_study	Ratio_in_pop	Rich factor	Pvalue	Padjust	First Category	Second Category	Gene_ids	Gene_names
41	map05132	Salmonella infection	KEGG PATHWAY	41/1477	271/14104	0.15129151	0.0101	0.06543	Human Diseases	Infectious disease: bacterial	ENSGALG000000089 33|ENSGALG00000006480|ENSGALG00000016485|ENSGALG000000068 1|ENSGALG00000007740|ENSGALG00000017186|ENSGALG000000258 3|ENSGALG00000008444|ENSGALG00000011687|ENSGALG000000031527|ENSGALG00000028037|ENSGALG00000029940|ENSGALG0000000005	CASP7;TCF7;RHOB;PAK1;;BIRC3;PIK3CD;TUBAL3;;ARL8BL;FOS;IL1B;TUBB3;RIPK2;KIF5C;IL6;CASP18;;MAPK10;TLR5;IL8;RPS3;;FHOD1;LY96;TUBA3E;TNFSF10;;DYNC1I1;PODXL;WASF3;CASP8;RAB7B;CYFIP2;;IL8L1;CYTH4;RHOG;MAP2K6;TRAF2;NFKBIA

Num	Pathway id	Description	Database	Ratio_in_study	Ratio_in_pop	Rich factor	Pvalue	Padjust	First Category	Second Category	Gene_ids	Gene_names
10	map00260	Glycine, serine and threonine metabolism	KEGG PATHWAY	10/1477	43/14104	0.23255814	0.0117	0.07351	Metabolism	Amino acid metabolism	ENSGALG00000002397|ENSGALG00000002988|ENSGALG00000012312|ENSGALG00000008229|ENSGALG00000015180|ENSGALG00000011331|ENSGALG00000002638|ENSGALG00000016196|ENSGALG00000046412|ENSGALG00000052983	PSPH;;GCAT;ALDH7A1;PSAT1;CTH;;;;

Num	Pathway id	Description	Database	Ratio_in_study	Ratio_in_pop	Rich factor	Pvalue	Padjust	First Category	Second Category	Gene_ids	Gene_names
24	map04270	Vascular smooth muscle contraction	KEGG PATHWAY	24/1477	142/14104	0.16901408	0.0123	0.07576	Organismal Systems	Circulatory system	ENSGALG000000020 16|ENSGALG00000031244|ENSGALG00000037246|ENSGALG000000004 69|ENSGALG00000031929|ENSGALG00000003149|ENSGALG000000004574|ENSGALG00000012735|ENSGALG00000013022|ENSGALG000000027323|ENSGALG00000024449|ENSGALG00000014117|ENSGALG0000000001111	PRKCD;ADCY6;RAMP3;ADRA1A;;;NPPA;EDN1;CACNA1C;MYH1D;RAMP2;AVP;AGT;;CALD1;MYH10;ITPR2;ADORA2B;PPP1R12B;;PRKCB;AVPR1B;EDN2;

Num	Pathway id	Description	Database	Ratio_in_study	Ratio_in_pop	Rich factor	Pvalue	Padjust	First Category	Second Category	Gene_ids	Gene_names
20	map04659	Th17 cell differentiation	KEGG PATHWAY	20/1477	113/14104	0.17699115	0.0131	0.07896	Organismal Systems	Immune system	ENSGALG00000030940|ENSGALG00000029940|ENSGALG00000029270|ENSGALG00000007651|ENSGALG00000006318|ENSGALG00000027864|ENSGALG00000012055|ENSGALG00000010915|ENSGALG00000000162|ENSGALG00000028037|ENSGALG00000016022|ENSGALG00000004322|ENSGALG00000001110	BLB2;IL1B;GATA3;STAT1;IL21R;NFKBIA;;IL6;DMB1;FOS;RUNX1;;MAPK10;NFKBIE;;IRF9;IL2RB;IL2RA;JAK3;IRF4
6	map00670	One carbon pool by folate	KEGG PATHWAY	6/1477	20/14104	0.3	0.0139	0.08102	Metabolism	Metabolism of cofactors and vitamins	ENSGALG00000026757|ENSGALG00000012412|ENSGALG00000052983|ENSGALG00000014464|ENSGALG00000034344|ENSGALG00000012691	DHFR;MTHFD1L;;MTR;MTHFD2;ALDH1L2

Num	Pathway id	Description	Database	Ratio_in_study	Ratio_in_pop	Rich factor	Pvalue	Padjust	First Category	Second Category	Gene_ids	Gene_names
10	map04930	Type II diabetes mellitus	KEGG PATHWAY	10/1477	44/14104	0.22727273	0.0138	0.08181	Human Diseases	Endocrine and metabolic disease	ENSGALG00000002016|ENSGALG00000002583|ENSGALG00000021039|ENSGALG00000003833|ENSGALG00000027786|ENSGALG00000037131|ENSGALG00000014786|ENSGALG00000011109|ENSGALG00000013022|ENSGALG00000007158	PRKCD;PIK3CD;HKDC1;CACNA1E;SOCS3;;PIK3R1;MAPK10;CACNA1C;SOCS1

Num	Pathway id	Description	Database	Ratio_in_study	Ratio_in_pop	Rich factor	Pvalue	Padjust	First Category	Second Category	Gene_ids	Gene_names
12	map04929	GnRH secretion	KEGG PATHWAY	12/1477	58/14104	0.20689655	0.0156	0.08904	Organismal Systems	Endocrine system	ENSGALG00000005215|ENSGALG00000003149|ENSGALG00000010926|ENSGALG00000031411|ENSGALG00000037943|ENSGALG00000001181|ENSGALG00000014786|ENSGALG00000014071|ENSGALG00000013022|ENSGALG00000017044|ENSGALG00000002583|ENSGALG00000033653	CACNA1H;;SPP1;KCNN1;PRKCB;KCNJ5;PIK3R1;ITPR2;CACNA1C;TRPC4;PIK3CD;

Num	Pathway id	Description	Database	Ratio_in_study	Ratio_in_pop	Rich factor	Pvalue	Padjust	First Category	Second Category	Gene_ids	Gene_names
11	map04623	Cytosolic DNA-sensing pathway	KEGG PATHWAY	11/1477	52/14104	0.21153846	0.0171	0.09637	Organismal Systems	Immune system	ENSGALG000000029940|ENSGALG00000041129|ENSGALG00000013356|ENSGALG00000014297|ENSGALG00000027864|ENSGALG00000015062|ENSGALG00000010915|ENSGALG00000032717|ENSGALG00000034478|ENSGALG00000005759|ENSGALG00000054104	IL1B;TMEM173;IKBKE;IRF7;NFKBIA;;IL6;CCL4;CCL4;IFNW1;

Num	Pathway id	Description	Database	Ratio_in_study	Ratio_in_pop	Rich factor	Pvalue	Padjust	First Category	Second Category	Gene_ids	Gene_names
33	map05417	Lipid and atherosclerosis	KEGG PATHWAY	33/1477	217/14104	0.15207373	0.0181	0.10007	Human Diseases	Cardiovascular disease	ENSGALG00000007114|ENSGALG00000008933|ENSGALG00000010915|ENSGALG00000011389|ENSGALG00000005759|ENSGALG00000005257|ENSGALG00000029940|ENSGALG00000014297|ENSGALG00000015062|ENSGALG00000008346|ENSGALG00000013356|ENSGALG00000014786|ENSGALG00000002609	APOA1;CASP7;IL6;TRAF3;IFNW1;;IL1B;IRF7;;CASP18;IKBKE;PIK3R1;IL8;MAPK10;;CD36;;;PIK3CD;LY96;NCF1C;TNFSF10;;FOS;MMP9;MAP2K6;CASP8;;IL8L1;MMP10;TRAF2;NFKBIA;BID

Num	Pathway id	Description	Database	Ratio_in_study	Ratio_in_pop	Rich factor	Pvalue	Padjust	First Category	Second Category	Gene_ids	Gene_names
34	map05205	Proteoglycans in cancer	KEGG PATHWAY	34/1477	227/14104	0.14977974	0.0205	0.10787	Human Diseases	Cancer: overview	ENSGALG000000009641|ENSGALG00000006087|ENSGALG00000035282|ENSGALG000000000681|ENSGALG00000003589|ENSGALG00000009626|ENSGALG000000039080|ENSGALG00000033295|ENSGALG00000003149|ENSGALG000000002583|ENSGALG00000036883|ENSGALG00000043287|ENSGALG0000001029	COL1A2;GPC3;IGF2;PAK1;VTN;THBS1;CD44;CAV2;;PIK3CD;MET;CAV1;VEGFA;ITGA2;PIK3R1;HPSE;TWIST1;PLCE1;KDR;RPS6;WNT16;ITPR2;;ITGB3;WNT5A;ANK2;LUM;SDC4;MMP9;PPP1R12B;PRKCB;CAV3;WNT11B;HBEGF

Num	Pathway id	Description	Database	Ratio_in_study	Ratio_in_pop	Rich factor	Pvalue	Padjust	First Category	Second Category	Gene_ids	Gene_names
15	map01521	EGFR tyrosine kinase inhibitor resistance	KEGG PATHWAY	15/1477	81/14104	0.18518519	0.0199	0.10836	Human Diseases	Drug resistance: antineoplastic	ENSGALG00000015082|ENSGALG00000036883|ENSGALG00000037943|ENSGALG00000010290|ENSGALG00000010915|ENSGALG00000003642|ENSGALG00000012178|ENSGALG00000016820|ENSGALG00000014786|ENSGALG00000026153|ENSGALG00000005011|ENSGALG00000013907|ENSGALG00000003364	RPS6;MET;PRKCB;VEGFA;IL6;PDGFA;PDGFB;GAS6;PIK3R1;FOXO6;;KDR;NRG2;PIK3CD;TGFA

Num	Pathway id	Description	Database	Ratio_in_study	Ratio_in_pop	Rich factor	Pvalue	Padjust	First Category	Second Category	Gene_ids	Gene_names
9	map05219	Bladder cancer	KEGG PATHWAY	9/1477	40/14104	0.225	0.0203	0.10852	Human Diseases	Cancer: specific types	ENSGALG00000000949|ENSGALG00000006992|ENSGALG00000010290|ENSGALG00000046160|ENSGALG00000026098|ENSGALG00000009626|ENSGALG00000051375|ENSGALG00000038951|ENSGALG00000011668	HBEGF;MMP9;VEGFA;;IL8;THBS1;;RASSF1;IL8L1

Num	Pathway id	Description	Database	Ratio_in_study	Ratio_in_pop	Rich factor	Pvalue	Padjust	First Category	Second Category	Gene_ids	Gene_names
19	map04750	Inflammatory mediator regulation of TRP channels	KEGG PATHWAY	19/1477	111/14104	0.17117117	0.0213	0.1105	Organismal Systems	Sensory system	ENSGALG000000020016|ENSGALG00000031244|ENSGALG00000003149|ENSGALG000000029940|ENSGALG00000020386|ENSGALG00000034025|ENSGALG000000012396|ENSGALG00000037943|ENSGALG00000016992|ENSGALG000000011080|ENSGALG00000007681|ENSGALG00000014786|ENSGALG0000000001110	PRKCD;ADCY6;;IL1B;BDKRB1;ASIC1;PTGER2;PRKCB;HTR2A;BDKRB2;HTR2B;PIK3R1;MAPK10;ITPR2;MAP2K6;F2RL1;;PIK3CD;NGF

Num	Pathway id	Description	Database	Ratio_in_study	Ratio_in_pop	Rich factor	Pvalue	Padjust	First Category	Second Category	Gene_ids	Gene_names
13	map05218	Melanoma	KEGG PATHWAY	13/1477	68/14104	0.19117647	0.0227	0.1161	Human Diseases	Cancer: specific types	ENSGALG000000028005|ENSGALG00000016866|ENSGALG00000002583|ENSGALG00000036883|ENSGALG00000025748|ENSGALG00000003642|ENSGALG00000012178|ENSGALG00000029968|ENSGALG00000014786|ENSGALG00000002203|ENSGALG00000051375|ENSGALG00000007562|ENSGALG0000000650	GADD45G;FGF14;PIK3CD;MET;FGF9;PDGFA;PDGFB;GADD45B;PIK3R1;FGF18;;FGF4;FGF13

Num	Pathway id	Description	Database	Ratio_in_study	Ratio_in_pop	Rich factor	Pvalue	Padjust	First Category	Second Category	Gene_ids	Gene_names
28	map05146	Amoebiasis	KEGG PATHWAY	28/1477	183/14104	0.15300546	0.0258	0.12989	Human Diseases	Infectious disease: parasitic	ENSGALG000000009641|ENSGALG00000010243|ENSGALG00000046160|ENSGALG00000053107|ENSGALG00000053028|ENSGALG00000042388|ENSGALG00000000769|ENSGALG00000029940|ENSGALG00000001343|ENSGALG00000036798|ENSGALG00000007917|ENSGALG00000010915|ENSGALG00000001478	COL1A2;PRDX1;;;;;RAB7B;IL1B;LAMB3;COL4A1;LAMB4;IL6;PIK3R1;IL8;;;COL4A2;LAMC1;PIK3CD;LAMA5;SERPINB10B;PRKCB;SERPINB1;NOS2;COL3A1;;IL8L1;TGFB3
3	map00750	Vitamin B6 metabolism	KEGG PATHWAY	3/1477	7/14104	0.42857143	0.029	0.13967	Metabolism	Metabolism of cofactors and vitamins	ENSGALG00000015180|ENSGALG00000020876|ENSGALG00000008185	PSAT1;AOX2;AOX1

Num	Pathway id	Description	Database	Ratio_in_study	Ratio_in_pop	Rich factor	Pvalue	Padjust	First Category	Second Category	Gene_ids	Gene_names
12	map00983	Drug metabolism - other enzymes	KEGG PATHWAY	12/1477	63/14104	0.19047619	0.0287	0.14011	Metabolism	Xenobiotics biodegradation and metabolism	ENSGALG000000016325|ENSGALG00000016324|ENSGALG00000007382|ENSGALG00000016442|ENSGALG00000016322|ENSGALG00000013073|ENSGALG00000050831|ENSGALG00000038923|ENSGALG00000002932|ENSGALG00000011805|ENSGALG00000007191|ENSGALG00000048343	GSTA3;GSTA4;CDA;RRM2;;UPP1;;;NME2;;TK1;

Num	Pathway id	Description	Database	Ratio_in_study	Ratio_in_pop	Rich factor	Pvalue	Padjust	First Category	Second Category	Gene_ids	Gene_names
10	map00480	Glutathione metabolism	KEGG PATHWAY	10/1477	49/14104	0.20408163	0.0284	0.14058	Metabolism	Metabolism of other amino acids	ENSGALG00000016325|ENSGALG00000016324|ENSGALG00000002595|ENSGALG00000016442|ENSGALG00000016322|ENSGALG00000027874|ENSGALG00000001062|ENSGALG00000004521|ENSGALG00000040504|ENSGALG00000010633	GSTA3;GSTA4;GPX4;RRM2;;CHAC1;GGT7;GPX3;GGT5;GPX7

Num	Pathway id	Description	Database	Ratio_in_study	Ratio_in_pop	Rich factor	Pvalue	Padjust	First Category	Second Category	Gene_ids	Gene_names
19	map04935	Growth hormone synthesis, secretion and action	KEGG PATHWAY	19/1477	115/14104	0.16521739	0.0298	0.14123	Organismal Systems	Endocrine system	ENSGALG000000031244|ENSGALG00000003149|ENSGALG00000002583|ENSGALG000000007651|ENSGALG00000008382|ENSGALG00000008393|ENSGALG000000004370|ENSGALG00000037943|ENSGALG00000027786|ENSGALG000000028037|ENSGALG00000014855|ENSGALG00000007158|ENSGALG0000001478	ADCY6;;PIK3CD;STAT1;GNAI1;CREB3L1;MAP2K6;PRKCB;SOCS3;FOS;GHR;SOCS1;PIK3R1;MAPK10;ITPR2;CACNA1C;SSTR2;;IGFALS
2	map00290	Valine, leucine and isoleucine biosynthesis	KEGG PATHWAY	2/1477	3/14104	0.66666667	0.0306	0.14303	Metabolism	Amino acid metabolism	ENSGALG000000002638|ENSGALG00000013177	;BCAT1

Num	Pathway id	Description	Database	Ratio_in_study	Ratio_in_pop	Rich factor	Pvalue	Padjust	First Category	Second Category	Gene_ids	Gene_names
38	map05202	Transcriptional misregulation in cancer	KEGG PATHWAY	38/1477	267/14104	0.1423221	0.031	0.14304	Human Diseases	Cancer: overview	ENSGALG00000016462|ENSGALG00000017186|ENSGALG00000003642|ENSGALG00000000504|ENSGALG00000030008|ENSGALG00000006511|ENSGALG00000045534|ENSGALG00000035244|ENSGALG00000014645|ENSGALG00000013568|ENSGALG00000051325|ENSGALG00000010915|ENSGALG00000002609	MYCN;BIRC3;PDGFA;ETV7;HHEX;BCL2A1;;;MEF2C;NR4A3;;IL6;IL8;;GADD45G;CSF1R;TRAF1;NFKBIZ;WNT16;PAX5;;RUNX1;GADD45B;SPI1;;;PAX3;MET;HMGA2;MMP9;;IL2RB;REL;CCNA2;MMP10;ETV6;;IL8L1

Num	Pathway id	Description	Database	Ratio_in_study	Ratio_in_pop	Rich factor	Pvalue	Padjust	First Category	Second Category	Gene_ids	Gene_names
9	map05033	Nicotine addiction	KEGG PATHWAY	9/1477	43/14104	0.20930233	0.0315	0.14329	Human Diseases	Substance dependence	ENSGALG00000007278|ENSGALG00000027415|ENSGALG00000038995|ENSGALG00000037131|ENSGALG00000014202|ENSGALG00000016744|ENSGALG00000004083|ENSGALG00000050064|ENSGALG00000041042	GRIN2A;GRIN2C;GRIA4;;GABRA4;GABRA5;GRIA1;;GABRG3

Num	Pathway id	Description	Database	Ratio_in_study	Ratio_in_pop	Rich factor	Pvalue	Padjust	First Category	Second Category	Gene_ids	Gene_names
56	map04020	Calcium signaling pathway	KEGG PATHWAY	56/1477	419/14104	0.13365155	0.0331	0.14446	Environmental Information Processing	Signal transduction	ENSGALG00000025748|ENSGALG00000016564|ENSGALG00000016912|ENSGALG00000026902|ENSGALG00000007278|ENSGALG00000027415|ENSGALG00000008177|ENSGALG00000039935|ENSGALG00000023142|ENSGALG00000008709|ENSGALG00000008544|ENSGALG00000000469|ENSGALG00000004003	FGF9;PTK2B;EDNRB;P2RX6;GRIN2A;GRIN2C;NOS1;PDE1C;PTGER3;MCOLN3;SLC8A1;ADRA1A;ADRB2;SPHK1;SLC8A3;CAMK1G;TNNC2;;HTR7;MET;RYR2;VEGFA;HTR2A;BDKRB2;PDGFB;MST1R;CACNA1C;PLCE1;NTRK3;TNNC1;NOS2;FGF14;PTGFR;PLCD4;;ITPR2;KDR;FGF4;FGF13;P2RX7;CACNA1H;ADORA2B;PTAFR;BDKRB1;PRKCB;CACNA1E;HTR2B;PDGFA;AVPR1B;FGF18;NGF;RET;;CASQ2;;CYSLTR2

Num	Pathway id	Description	Database	Ratio_in_study	Ratio_in_pop	Rich factor	Pvalue	Padjust	First Category	Second Category	Gene_ids	Gene_names
19	map05142	Chagas disease	KEGG PATHWAY	19/1477	116/14104	0.1637931	0.0322	0.14467	Human Diseases	Infectious disease: parasitic	ENSGALG000000027864|ENSGALG00000003163|ENSGALG00000029940|ENSGALG00000010915|ENSGALG00000008382|ENSGALG00000008355|ENSGALG00000038096|ENSGALG00000011668|ENSGALG00000011080|ENSGALG00000046160|ENSGALG00000028037|ENSGALG00000014786|ENSGALG00000002609	NFKBIA;GNAO1;IL1B;IL6;GNAI1;CASP8;NOS2;IL8L1;BDKRB2;;FOS;PIK3R1;IL8;CASP18;IFNW1;TGFB3;PIK3CD;;MAPK10

Num	Pathway id	Description	Database	Ratio_in_study	Ratio_in_pop	Rich factor	Pvalue	Padjust	First Category	Second Category	Gene_ids	Gene_names
19	map04915	Estrogen signaling pathway	KEGG PATHWAY	19/1477	116/14104	0.1637931	0.0322	0.14467	Organismal Systems	Endocrine system	ENSGALG00000002016|ENSGALG00000031244|ENSGALG00000003163|ENSGALG00000002583|ENSGALG00000006992|ENSGALG00000008382|ENSGALG00000003690|ENSGALG00000008393|ENSGALG00000016600|ENSGALG00000003149|ENSGALG00000034204|ENSGALG00000028037|ENSGALG0000000118	PRKCD;ADCY6;GNAO1;PIK3CD;MMP9;GNAI1;KRT14;CREB3L1;POMC;;;FOS;KCNJ5;PIK3R1;KRT18;ITPR2;;HBEGF;TGFA

Num	Pathway id	Description	Database	Ratio_in_study	Ratio_in_pop	Rich factor	Pvalue	Padjust	First Category	Second Category	Gene_ids	Gene_names
8	map04216	Ferroptosis	KEGG PATHWAY	8/1477	37/14104	0.21621622	0.0347	0.1498	Cellular Processes	Cell growth and death	ENSGALG000000012298|ENSGALG00000002595|ENSGALG00000016348|ENSGALG00000009758|ENSGALG00000012117|ENSGALG00000007220|ENSGALG00000012847|ENSGALG00000010628	SLC39A8;GPX4;SAT1;SLC7A11;STEAP3;FTH1;;ACSL1
8	map04973	Carbohydrate digestion and absorption	KEGG PATHWAY	8/1477	38/14104	0.21052632	0.0401	0.17075	Organismal Systems	Digestive system	ENSGALG00000002583|ENSGALG00000010899|ENSGALG00000021039|ENSGALG00000037943|ENSGALG00000008427|ENSGALG00000002466|ENSGALG00000014786|ENSGALG00000038740	PIK3CD;G6PC2;HKDC1;PRKCB;GNAT3;SLC2A5;PIK3R1;AMY2A

Num	Pathway id	Description	Database	Ratio_in_study	Ratio_in_pop	Rich factor	Pvalue	Padjust	First Category	Second Category	Gene_ids	Gene_names
16	map04350	TGF-beta signaling pathway	KEGG PATHWAY	16/1477	97/14104	0.16494845	0.0441	0.1831	Environmental Information Processing	Signal transduction	ENSGALG00000006158|ENSGALG00000047554|ENSGALG00000034661|ENSGALG00000039474|ENSGALG00000041257|ENSGALG00000012429|ENSGALG00000026137|ENSGALG00000012055|ENSGALG00000009256|ENSGALG00000053285|ENSGALG00000042492|ENSGALG00000009626|ENSGALG00000002954	ACVR2B;GDF7;ID1;ID4;ACVR1C;BMP4;CDKN2B;;LEFTY1;;PITX2;THBS1;SMAD7B;INHBB;;TGFB3

Num	Pathway id	Description	Database	Ratio_in_study	Ratio_in_pop	Rich factor	Pvalue	Padjust	First Category	Second Category	Gene_ids	Gene_names
12	map04742	Taste transduction	KEGG PATHWAY	12/1477	67/14104	0.17910448	0.0439	0.18451	Organismal Systems	Sensory system	ENSGALG000000003149|ENSGALG00000039696|ENSGALG00000004346|ENSGALG00000034077|ENSGALG00000037131|ENSGALG00000014202|ENSGALG00000016744|ENSGALG00000039935|ENSGALG00000008427|ENSGALG00000013022|ENSGALG00000014742|ENSGALG00000010357	;ENTPD2;P2RY4;KCNK5;;GABRA4;GABRA5;PDE1C;GNAT3;CACNA1C;HTR1A;P2RY1

Num	Pathway id	Description	Database	Ratio_in_study	Ratio_in_pop	Rich factor	Pvalue	Padjust	First Category	Second Category	Gene_ids	Gene_names
17	map04724	Glutamatergic synapse	KEGG PATHWAY	17/1477	105/14104	0.16190476	0.0453	0.18567	Organismal Systems	Nervous system	ENSGALG00000031244|ENSGALG00000003163|ENSGALG00000037943|ENSGALG00000008382|ENSGALG00000008518|ENSGALG00000010187|ENSGALG00000007278|ENSGALG00000014813|ENSGALG00000038995|ENSGALG00000003149|ENSGALG00000037131|ENSGALG00000014071|ENSGALG00000001302	ADCY6;GNAO1;PRKCB;GNAI1;;SLC1A1;GRIN2A;HOMER1;GRIA4;;;ITPR2;CACNA1C;GRIA1;GRM3;GRIN2C;GRIK1

Num	Pathway id	Description	Database	Ratio_in_study	Ratio_in_pop	Rich factor	Pvalue	Padjust	First Category	Second Category	Gene_ids	Gene_names
8	map04624	Toll and Imd signaling pathway	KEGG PATHWAY	8/1477	39/14104	0.20512821	0.046	0.18629	Organismal Systems	Immune system	ENSGALG00000041510|ENSGALG00000008355|ENSGALG00000008346|ENSGALG00000012044|ENSGALG00000007845|ENSGALG00000017186|ENSGALG00000011109|ENSGALG00000027864	DUOX2;CASP8;CASP18;ANK2;REL;BIRC3;MAPK10;NFKBIA

Num	Pathway id	Description	Database	Ratio_in_study	Ratio_in_pop	Rich factor	Pvalue	Padjust	First Category	Second Category	Gene_ids	Gene_names
13	map04911	Insulin secretion	KEGG PATHWAY	13/1477	75/14104	0.17333333	0.0466	0.18634	Organismal Systems	Endocrine system	ENSGALG000000031244|ENSGALG00000003149|ENSGALG00000037943|ENSGALG00000031929|ENSGALG00000010812|ENSGALG00000008393|ENSGALG00000031411|ENSGALG00000005223|ENSGALG00000043694|ENSGALG00000029788|ENSGALG00000013022|ENSGALG00000011104|ENSGALG00000001485	ADCY6;;PRKCB;;RYR2;CREB3L1;KCNN1;ADCYAP1R1;RAPGEF4;CCK;CACNA1C;GCG;ADCYAP1

Num	Pathway id	Description	Database	Ratio_in_study	Ratio_in_pop	Rich factor	Pvalue	Padjust	First Category	Second Category	Gene_ids	Gene_names
17	map04725	Cholinergic synapse	KEGG PATHWAY	17/1477	106/14104	0.16037736	0.0489	0.1933	Organismal Systems	Nervous system	ENSGALG00000031244|ENSGALG00000003163|ENSGALG00000008365|ENSGALG00000016804|ENSGALG00000008382|ENSGALG00000008393|ENSGALG00000037943|ENSGALG00000003149|ENSGALG00000028037|ENSGALG00000037131|ENSGALG00000037138|ENSGALG00000014786|ENSGALG0000001407	ADCY6;GNAO1;CHRM4;SLC5A7;GNAI1;CREB3L1;PRKCB;;FOS;;KCNQ1;PIK3R1;ITPR2;CACNA1C;PIK3R5;KCNJ4;PIK3CD

Num	Pathway id	Description	Database	Ratio_in_study	Ratio_in_pop	Rich factor	Pvalue	Padjust	First Category	Second Category	Gene_ids	Gene_names
15	map05032	Morphine addiction	KEGG PATHWAY	15/1477	91/14104	0.16483516	0.0504	0.19691	Human Diseases	Substance dependence	ENSGALG000000031244|ENSGALG00000003163|ENSGALG00000037131|ENSGALG000000000168|ENSGALG00000008382|ENSGALG00000041042|ENSGALG000000037943|ENSGALG00000027565|ENSGALG00000039935|ENSGALG000000001181|ENSGALG00000014202|ENSGALG00000016744|ENSGALG0000000003702	ADCY6;GNAO1;;ADORA1;GNAI1;GABRG3;PRKCB;;PDE1C;KCNJ5;GABRA4;GABRA5;;;PDE4B
6	map01523	Antifolate resistance	KEGG PATHWAY	6/1477	27/14104	0.22222222	0.0566	0.21866	Human Diseases	Drug resistance: antineoplastic	ENSGALG000000026757|ENSGALG00000029940|ENSGALG00000010293|ENSGALG000000010915|ENSGALG00000030677|ENSGALG00000052983	DHFR;IL1B;RBP;IL6;ABCG2;

Num	Pathway id	Description	Database	Ratio_in_study	Ratio_in_pop	Rich factor	Pvalue	Padjust	First Category	Second Category	Gene_ids	Gene_names
8	map00760	Nicotinate and nicotinamide metabolism	KEGG PATHWAY	8/1477	41/14104	0.19512195	0.0594	0.22172	Metabolism	Metabolism of cofactors and vitamins	ENSGALG00000008179|ENSGALG00000008185|ENSGALG00000014509|ENSGALG00000003651|ENSGALG00000034397|ENSGALG00000004842|ENSGALG00000020876|ENSGALG00000030120	NT5C2;AOX1;BST1;NT5C3B;;NT5M;AOX2;ITGB1BP3
7	map04215	Apoptosis - multiple species	KEGG PATHWAY	7/1477	34/14104	0.20588235	0.059	0.22259	Cellular Processes	Cell growth and death	ENSGALG00000008355|ENSGALG00000008933|ENSGALG00000008346|ENSGALG00000017186|ENSGALG00000011109|ENSGALG00000013039|ENSGALG00000035325	CASP8;CASP7;CASP18;BIRC3;MAPK10;BID;PMAIP1

Num	Pathway id	Description	Database	Ratio_in_study	Ratio_in_pop	Rich factor	Pvalue	Padjust	First Category	Second Category	Gene_ids	Gene_names
9	map00270	Cysteine and methionine metabolism	KEGG PATHWAY	9/1477	48/14104	0.1875	0.0587	0.22404	Metabolism	Amino acid metabolism	ENSGALG000000002988|ENSGALG00000015180|ENSGALG00000002358|ENSGALG00000016196|ENSGALG00000013177|ENSGALG00000011331|ENSGALG00000014464|ENSGALG00000002479|ENSGALG00000000893	;PSAT1;CDO1;;BCAT1;CTH;MTR;MAT1A;TAT

Num	Pathway id	Description	Database	Ratio_in_study	Ratio_in_pop	Rich factor	Pvalue	Padjust	First Category	Second Category	Gene_ids	Gene_names
14	map04925	Aldosterone synthesis and secretion	KEGG PATHWAY	14/1477	86/14104	0.1627907	0.0626	0.23077	Organismal Systems	Endocrine system	ENSGALG000000005215|ENSGALG00000031244|ENSGALG00000003149|ENSGALG000000011117|ENSGALG00000008393|ENSGALG00000037869|ENSGALG000000037943|ENSGALG00000016600|ENSGALG00000004574|ENSGALG000000001181|ENSGALG00000014071|ENSGALG00000013022|ENSGALG0000001253	CACNA1H;ADCY6;;AGT;CREB3L1;;PRKCB;POMC;NPPA;KCNJ5;ITPR2;CACNA1C;NR4A2;CAMK1G

Num	Pathway id	Description	Database	Ratio_in_study	Ratio_in_pop	Rich factor	Pvalue	Padjust	First Category	Second Category	Gene_ids	Gene_names
28	map05203	Viral carcinogenesis	KEGG PATHWAY	28/1477	199/14104	0.14070352	0.0646	0.23296	Human Diseases	Cancer: overview	ENSGALG00000049751|ENSGALG00000008393|ENSGALG00000037322|ENSGALG00000005653|ENSGALG00000011389|ENSGALG00000042838|ENSGALG00000003485|ENSGALG00000035325|ENSGALG00000002583|ENSGALG00000014297|ENSGALG00000026137|ENSGALG00000008346|ENSGALG00000004940	;CREB3L1;HIST1H46;NFKB2;TRAF3;TRAF2;CCND3;PMAIP1;PIK3CD;IRF7;CDKN2B;CASP18;HIST1H2B8;PIK3R1;EIF2AK2;JAK3;;TRAF1;CASP8;HIST1H2B7;;REL;CCNA2;ATP6V0D2;GSN;;EGR3;NFKBIA

Num	Pathway id	Description	Database	Ratio_in_study	Ratio_in_pop	Rich factor	Pvalue	Padjust	First Category	Second Category	Gene_ids	Gene_names
12	map04917	Prolactin signaling pathway	KEGG PATHWAY	12/1477	71/14104	0.16901408	0.0639	0.23329	Organismal Systems	Endocrine system	ENSGALG00000002260|ENSGALG00000002583|ENSGALG00000007651|ENSGALG00000006785|ENSGALG00000009603|ENSGALG00000026153|ENSGALG00000028037|ENSGALG00000014786|ENSGALG00000011109|ENSGALG00000007158|ENSGALG00000005011|ENSGALG00000027786	CISH;PIK3CD;STAT1;IRF1;;FOXO6;FOS;PIK3R1;MAPK10;SOCS1;;SOCS3

Num	Pathway id	Description	Database	Ratio_in_study	Ratio_in_pop	Rich factor	Pvalue	Padjust	First Category	Second Category	Gene_ids	Gene_names
21	map05224	Breast cancer	KEGG PATHWAY	21/1477	142/14104	0.14788732	0.0655	0.23387	Human Diseases	Cancer: specific types	ENSGALG000000028005|ENSGALG00000025748|ENSGALG00000028015|ENSGALG00000035031|ENSGALG00000011182|ENSGALG00000028069|ENSGALG00000005653|ENSGALG00000028037|ENSGALG00000029968|ENSGALG00000002055|ENSGALG00000014786|ENSGALG00000002203|ENSGALG0000000648	GADD45G;FGF9;HES1;HEY1;DLL1;WNT16;NFKB2;FOS;GADD45B;HES4;PIK3R1;FGF18;TCF7;WNT11B;FGF14;;;FGF4;FGF13;PIK3CD;WNT5A

Num	Pathway id	Description	Database	Ratio_in_study	Ratio_in_pop	Rich factor	Pvalue	Padjust	First Category	Second Category	Gene_ids	Gene_names
12	map05120	Epithelial cell signaling in Helicobacter pylori infection	KEGG PATHWAY	12/1477	72/14104	0.16666667	0.0698	0.23876	Human Diseases	Infectious disease: bacterial	ENSGALG00000036883|ENSGALG00000020754|ENSGALG00000027864|ENSGALG00000046160|ENSGALG00000001472|ENSGALG00000000681|ENSGALG00000026098|ENSGALG00000011109|ENSGALG00000035017|ENSGALG00000000949|ENSGALG00000011668|ENSGALG00000034294	MET;ATP6V0E2;NFKBIA;;JAM3;PAK1;IL8;MAPK10;;HBEGF;IL8L1;ATP6V0D2

Num	Pathway id	Description	Database	Ratio_in_study	Ratio_in_pop	Rich factor	Pvalue	Padjust	First Category	Second Category	Gene_ids	Gene_names
21	map04723	Retrograde endocannabinoid signaling	KEGG PATHWAY	21/1477	143/14104	0.14685315	0.0696	0.24307	Organismal Systems	Nervous system	ENSGALG000000031244|ENSGALG00000003163|ENSGALG00000037943|ENSGALG000000049848|ENSGALG00000008382|ENSGALG00000008285|ENSGALG000000041042|ENSGALG00000038995|ENSGALG00000003149|ENSGALG000000001181|ENSGALG00000014202|ENSGALG00000029500|ENSGALG0000000001674	ADCY6;GNAO1;PRKCB;CNR1;GNAI1;NAPEPLD;GABRG3;GRIA4;;KCNJ5;GABRA4;ND5;GABRA5;MAPK10;ITPR2;CACNA1C;GRIA1;;PTGS2;;MGLL

Num	Pathway id	Description	Database	Ratio_in_study	Ratio_in_pop	Rich factor	Pvalue	Padjust	First Category	Second Category	Gene_ids	Gene_names
21	map05160	Hepatitis C	KEGG PATHWAY	21/1477	143/14104	0.14685315	0.0696	0.24307	Human Diseases	Infectious disease: viral	ENSGALG00000002583|ENSGALG00000007651|ENSGALG00000014297|ENSGALG00000030661|ENSGALG00000008355|ENSGALG00000027786|ENSGALG00000015062|ENSGALG00000008346|ENSGALG00000011389|ENSGALG00000016400|ENSGALG00000013356|ENSGALG00000016142|ENSGALG00000004283	PIK3CD;STAT1;IRF7;STAT2;CASP8;SOCS3;;CASP18;TRAF3;RSAD2;IKBKE;MX1;TRAF2;EIF2AK2;CLDN1;PIK3R1;IFNW1;;;NFKBIA;BID

Num	Pathway id	Description	Database	Ratio_in_study	Ratio_in_pop	Rich factor	Pvalue	Padjust	First Category	Second Category	Gene_ids	Gene_names
15	map04540	Gap junction	KEGG PATHWAY	15/1477	95/14104	0.15789474	0.0688	0.24308	Cellular Processes	Cellular community - eukaryotes	ENSGALG000000031244|ENSGALG00000003149|ENSGALG00000000059|ENSGALG000000008382|ENSGALG00000015729|ENSGALG00000037943|ENSGALG000000016992|ENSGALG00000007681|ENSGALG00000003642|ENSGALG000000012178|ENSGALG00000000433|ENSGALG00000011324|ENSGALG0000000001407	ADCY6;;TUBB3;GNAI1;LPAR1;PRKCB;HTR2A;HTR2B;PDGFA;PDGFB;;;ITPR2;TUBA3E;TUBAL3

Num	Pathway id	Description	Database	Ratio_in_study	Ratio_in_pop	Rich factor	Pvalue	Padjust	First Category	Second Category	Gene_ids	Gene_names
11	map00240	Pyrimidine metabolism	KEGG PATHWAY	11/1477	65/14104	0.16923077	0.0735	0.24634	Metabolism	Nucleotide metabolism	ENSGALG00000008179|ENSGALG00000007382|ENSGALG00000034397|ENSGALG00000016442|ENSGALG00000008936|ENSGALG00000028982|ENSGALG00000003651|ENSGALG00000002932|ENSGALG00000004842|ENSGALG00000013073|ENSGALG00000007191	NT5C2;CDA;;RRM2;;CMPK2;NT5C3B;NME2;NT5M;UPP1;TK1

Num	Pathway id	Description	Database	Ratio_in_study	Ratio_in_pop	Rich factor	Pvalue	Padjust	First Category	Second Category	Gene_ids	Gene_names
9	map00330	Arginine and proline metabolism	KEGG PATHWAY	9/1477	50/14104	0.18	0.0728	0.24677	Metabolism	Amino acid metabolism	ENSGALG000000034655|ENSGALG00000031158|ENSGALG00000008229|ENSGALG00000016348|ENSGALG00000008177|ENSGALG00000019514|ENSGALG00000038096|ENSGALG00000007300|ENSGALG00000054033	ALDH18A1;OAT;ALDH7A1;SAT1;NOS1;CNDP2;NOS2;PYCR1;
7	map00982	Drug metabolism - cytochrome P450	KEGG PATHWAY	7/1477	36/14104	0.19444444	0.0764	0.25373	Metabolism	Xenobiotics biodegradation and metabolism	ENSGALG000000016325|ENSGALG00000016324|ENSGALG00000008185|ENSGALG00000016322|ENSGALG00000040573|ENSGALG00000011805|ENSGALG00000020876	GSTA3;GSTA4;AOX1;;FMO3;;AOX2

Num	Pathway id	Description	Database	Ratio_in_study	Ratio_in_pop	Rich factor	Pvalue	Padjust	First Category	Second Category	Gene_ids	Gene_names
12	map05220	Chronic myeloid leukemia	KEGG PATHWAY	12/1477	74/14104	0.16216216	0.0824	0.27083	Human Diseases	Cancer: specific types	ENSGALG00000028005|ENSGALG00000002583|ENSGALG00000027864|ENSGALG00000054619|ENSGALG00000016022|ENSGALG00000029968|ENSGALG00000014786|ENSGALG00000035017|ENSGALG00000005011|ENSGALG00000051375|ENSGALG00000012055|ENSGALG00000010346	GADD45G;PIK3CD;NFKBIA;GAB2;RUNX1;GADD45B;PIK3R1;;;;;TGFB3

Num	Pathway id	Description	Database	Ratio_in_study	Ratio_in_pop	Rich factor	Pvalue	Padjust	First Category	Second Category	Gene_ids	Gene_names
8	map00830	Retinol metabolism	KEGG PATHWAY	8/1477	44/14104	0.18181818	0.0838	0.27264	Metabolism	Metabolism of cofactors and vitamins	ENSGALG00000010873|ENSGALG00000045037|ENSGALG00000008185|ENSGALG00000004436|ENSGALG00000011532|ENSGALG00000011805|ENSGALG00000020876|ENSGALG00000043213	DHRS9;CYP2W1;AOX1;CYP3A4;CYP27C1;;AOX2;CYP26C1
7	map05204	Chemical carcinogenesis - DNA adducts	KEGG PATHWAY	7/1477	37/14104	0.18918919	0.0861	0.27769	Human Diseases	Cancer: overview	ENSGALG00000016325|ENSGALG00000016324|ENSGALG00000016322|ENSGALG00000004436|ENSGALG00000025822|ENSGALG00000011805|ENSGALG00000033635	GSTA3;GSTA4;;CYP3A4;CYP1B1;;PTGS2

Num	Pathway id	Description	Database	Ratio_in_study	Ratio_in_pop	Rich factor	Pvalue	Padjust	First Category	Second Category	Gene_ids	Gene_names
21	map05226	Gastric cancer	KEGG PATHWAY	21/1477	147/14104	0.14285714	0.0874	0.27904	Human Diseases	Cancer: specific types	ENSGALG00000028005|ENSGALG00000025748|ENSGALG00000007562|ENSGALG00000036883|ENSGALG00000026137|ENSGALG00000028069|ENSGALG00000035094|ENSGALG00000051375|ENSGALG00000029968|ENSGALG00000014786|ENSGALG00000027853|ENSGALG00000006480|ENSGALG00000000440	GADD45G;FGF9;FGF4;MET;CDKN2B;WNT16;CDH17;;GADD45B;PIK3R1;CTNNA2;TCF7;WNT11B;FGF14;FGF18;;TGFB3;FGF13;PIK3CD;;WNT5A

Num	Pathway id	Description	Database	Ratio_in_study	Ratio_in_pop	Rich factor	Pvalue	Padjust	First Category	Second Category	Gene_ids	Gene_names
26	map04064	NF-kappa B signaling pathway	KEGG PATHWAY	26/1477	189/14104	0.13756614	0.0896	0.28332	Environmental Information Processing	Signal transduction	ENSGALG000000046192|ENSGALG00000005653|ENSGALG00000011389|ENSGALG00000032717|ENSGALG00000042838|ENSGALG00000006511|ENSGALG00000005257|ENSGALG00000028256|ENSGALG00000029940|ENSGALG00000013861|ENSGALG00000026098|ENSGALG00000048671|ENSGALG0000002800	;NFKB2;TRAF3;CCL4;TRAF2;BCL2A1;;CCL19;IL1B;TNFAIP3;IL8;;GADD45G;TRAF1;GADD45B;LY96;;NFKBIA;PRKCB;CCL4;;CARD11;BIRC3;PTGS2;;IL8L1

Num	Pathway id	Description	Database	Ratio_in_study	Ratio_in_pop	Rich factor	Pvalue	Padjust	First Category	Second Category	Gene_ids	Gene_names
11	map05211	Renal cell carcinoma	KEGG PATHWAY	11/1477	68/14104	0.16176471	0.0949	0.29432	Human Diseases	Cancer: specific types	ENSGALG000000012178|ENSGALG00000002583|ENSGALG00000010005|ENSGALG000000036883|ENSGALG00000010290|ENSGALG00000010001|ENSGALG000000000681|ENSGALG00000014786|ENSGALG00000035017|ENSGALG000000024024|ENSGALG00000010346	PDGFB;PIK3CD;EPAS1;MET;VEGFA;EGLN3;PAK1;PIK3R1;;TGFA;TGFB3

Num	Pathway id	Description	Database	Ratio_in_study	Ratio_in_pop	Rich factor	Pvalue	Padjust	First Category	Second Category	Gene_ids	Gene_names
19	map04068	FoxO signaling pathway	KEGG PATHWAY	19/1477	132/14104	0.14393939	0.0946	0.2964	Environmental Information Processing	Signal transduction	ENSGALG000000028005|ENSGALG00000014725|ENSGALG00000026153|ENSGALG00000010129|ENSGALG00000041202|ENSGALG00000014813|ENSGALG00000026137|ENSGALG00000010915|ENSGALG00000010826|ENSGALG00000013372|ENSGALG00000009800|ENSGALG00000029968|ENSGALG00000001478	GADD45G;PLK2;FOXO6;PLK3;FBXO32;HOMER1;CDKN2B;IL6;PRKAA2;IL7R;SETD7;GADD45B;PIK3R1;MAPK10;TNFSF10;G6PC2;PIK3CD;;TGFB3

Num	Pathway id	Description	Database	Ratio_in_study	Ratio_in_pop	Rich factor	Pvalue	Padjust	First Category	Second Category	Gene_ids	Gene_names
24	map04022	cGMP-PKG signaling pathway	KEGG PATHWAY	24/1477	174/14104	0.13793103	0.0975	0.29967	Environmental Information Processing	Signal transduction	ENSGALG000000031244|ENSGALG00000016912|ENSGALG00000015358|ENSGALG00000008393|ENSGALG00000008544|ENSGALG00000000469|ENSGALG00000004003 5|ENSGALG00000009400|ENSGALG00000003149|ENSGALG00000014645|ENSGALG00000048401|ENSGALG00000011080|ENSGALG0000000457	ADCY6;EDNRB;MYH15;CREB3L1;SLC8A1;ADRA1A;ADRB2;SLC8A3;;MEF2C;;BDKRB2;NPPA;CACNA1C;;;GNAI1;;ITPR2;ADORA1;RGS2;;ADRA2A;PIK3R5

Num	Pathway id	Description	Database	Ratio_in_study	Ratio_in_pop	Rich factor	Pvalue	Padjust	First Category	Second Category	Gene_ids	Gene_names
11	map04920	Adipocytokine signaling pathway	KEGG PATHWAY	11/1477	69/14104	0.15942029	0.1027	0.31293	Organismal Systems	Endocrine system	ENSGALG00000016600|ENSGALG00000008439|ENSGALG00000010899|ENSGALG00000027864|ENSGALG00000027786|ENSGALG00000010826|ENSGALG00000042838|ENSGALG00000011109|ENSGALG00000035017|ENSGALG00000044525|ENSGALG00000010628	POMC;CD36;G6PC2;NFKBIA;SOCS3;PRKAA2;TRAF2;MAPK10;;NFKBIE;ACSL1
4	map04614	Renin-angiotensin system	KEGG PATHWAY	4/1477	18/14104	0.22222222	0.1117	0.3342	Organismal Systems	Endocrine system	ENSGALG00000010331|ENSGALG00000011117|ENSGALG00000005936|ENSGALG00000037521	MME;AGT;AGTR2;PRCP

Num	Pathway id	Description	Database	Ratio_in_study	Ratio_in_pop	Rich factor	Pvalue	Padjust	First Category	Second Category	Gene_ids	Gene_names
4	map00220	Arginine biosynthesis	KEGG PATHWAY	4/1477	18/14104	0.22222222	0.1117	0.3342	Metabolism	Amino acid metabolism	ENSGALG000000008518|ENSGALG00000033338|ENSGALG00000008177|ENSGALG000000038096	;GPT2;NOS1;NOS2
11	map05321	Inflammatory bowel disease	KEGG PATHWAY	11/1477	70/14104	0.15714286	0.111	0.335	Human Diseases	Immune disease	ENSGALG000000030940|ENSGALG00000029940|ENSGALG00000029270|ENSGALG000000007651|ENSGALG00000006318|ENSGALG00000010915|ENSGALG000000000162|ENSGALG00000040621|ENSGALG00000009392|ENSGALG000000035447|ENSGALG00000010346	BLB2;IL1B;GATA3;STAT1;IL21R;IL6;DMB1;IL18R1;TLR5;;TGFB3

Num	Pathway id	Description	Database	Ratio_in_study	Ratio_in_pop	Rich factor	Pvalue	Padjust	First Category	Second Category	Gene_ids	Gene_names
11	map05212	Pancreatic cancer	KEGG PATHWAY	11/1477	71/14104	0.15492958	0.1196	0.35145	Human Diseases	Cancer: specific types	ENSGALG00000028005|ENSGALG00000002583|ENSGALG00000007651|ENSGALG000000010290|ENSGALG00000029968|ENSGALG00000014786|ENSGALG000000011109|ENSGALG00000051375|ENSGALG00000010346|ENSGALG000000012055|ENSGALG00000024024	GADD45G;PIK3CD;STAT1;VEGFA;GADD45B;PIK3R1;MAPK10;;TGFB3;;TGFA
6	map00512	Mucin type O-glycan biosynthesis	KEGG PATHWAY	6/1477	33/14104	0.18181818	0.125	0.36076	Metabolism	Glycan biosynthesis and metabolism	ENSGALG000000006233|ENSGALG00000011235|ENSGALG00000001130|ENSGALG000000009027|ENSGALG00000054784|ENSGALG00000014944	GALNT11;GALNT15;GALNT17;ST6GALNAC3;;GCNT4

Num	Pathway id	Description	Database	Ratio_in_study	Ratio_in_pop	Rich factor	Pvalue	Padjust	First Category	Second Category	Gene_ids	Gene_names
8	map02010	ABC transporters	KEGG PATHWAY	8/1477	48/14104	0.16666667	0.124	0.36121	Environmental Information Processing	Membrane transport	ENSGALG00000008912|ENSGALG00000030677|ENSGALG00000010891|ENSGALG00000035075|ENSGALG00000029381|ENSGALG00000011096|ENSGALG00000021395|ENSGALG00000021399	ABCB1;ABCG2;ABCB11;TAP1;;ABCB10;;

Num	Pathway id	Description	Database	Ratio_in_study	Ratio_in_pop	Rich factor	Pvalue	Padjust	First Category	Second Category	Gene_ids	Gene_names
13	map04610	Complement and coagulation cascades	KEGG PATHWAY	13/1477	88/14104	0.14772727	0.1276	0.36207	Organismal Systems	Immune system	ENSGALG000000023950|ENSGALG00000031149|ENSGALG00000020386|ENSGALG00000019552|ENSGALG00000012802|ENSGALG00000011080|ENSGALG00000001565|ENSGALG00000003589|ENSGALG00000026677|ENSGALG00000040832|ENSGALG00000005077|ENSGALG00000031659|ENSGALG00000004479	CR1L;;BDKRB1;SERPINB10;F13A1;BDKRB2;C5;VTN;F10;CFD;F8;;VSIG4

Num	Pathway id	Description	Database	Ratio_in_study	Ratio_in_pop	Rich factor	Pvalue	Padjust	First Category	Second Category	Gene_ids	Gene_names
9	map00590	Arachidonic acid metabolism	KEGG PATHWAY	9/1477	56/14104	0.16071429	0.1268	0.36286	Metabolism	Lipid metabolism	ENSGALG000000012791|ENSGALG00000019233|ENSGALG00000001314|ENSGALG000000004521|ENSGALG00000040504|ENSGALG00000010633|ENSGALG000000046708|ENSGALG00000033635|ENSGALG00000030886	TBXAS1;;PTGS1;GPX3;GGT5;GPX7;PTGES;PTGS2;PTGDS
5	map00534	Glycosaminoglycan biosynthesis - heparan sulfate / heparin	KEGG PATHWAY	5/1477	26/14104	0.19230769	0.1293	0.36379	Metabolism	Glycan biosynthesis and metabolism	ENSGALG000000026594|ENSGALG00000012015|ENSGALG00000043829|ENSGALG000000006757|ENSGALG00000048802	HS3ST5;NDST4;;XYLT1;

Num	Pathway id	Description	Database	Ratio_in_study	Ratio_in_pop	Rich factor	Pvalue	Padjust	First Category	Second Category	Gene_ids	Gene_names
16	map04931	Insulin resistance	KEGG PATHWAY	16/1477	113/14104	0.14159292	0.1306	0.36432	Human Diseases	Endocrine and metabolic disease	ENSGALG00000002016|ENSGALG00000008439|ENSGALG00000011117|ENSGALG00000008393|ENSGALG00000027864|ENSGALG00000037943|ENSGALG00000010915|ENSGALG00000010826|ENSGALG00000027786|ENSGALG00000014786|ENSGALG00000011109|ENSGALG00000035017|ENSGALG00000001089	PRKCD;CD36;AGT;CREB3L1;NFKBIA;PRKCB;IL6;PRKAA2;SOCS3;PIK3R1;MAPK10;;G6PC2;SLC27A6;PIK3CD;SREBF1

Num	Pathway id	Description	Database	Ratio_in_study	Ratio_in_pop	Rich factor	Pvalue	Padjust	First Category	Second Category	Gene_ids	Gene_names
17	map04670	Leukocyte transendothelial migration	KEGG PATHWAY	17/1477	122/14104	0.13934426	0.1355	0.37501	Organismal Systems	Immune system	ENSGALG000000016564|ENSGALG00000006992|ENSGALG00000008382|ENSGALG000000046125|ENSGALG00000037943|ENSGALG00000048592|ENSGALG000000001472|ENSGALG00000026862|ENSGALG00000014786|ENSGALG000000027853|ENSGALG00000035017|ENSGALG00000001189|ENSGALG0000000004377	PTK2B;MMP9;GNAI1;PECAM1;PRKCB;;JAM3;CLDN1;PIK3R1;CTNNA2;;NCF1C;RAPGEF3;RASSF5;RAPGEF4;PIK3CD;

Num	Pathway id	Description	Database	Ratio_in_study	Ratio_in_pop	Rich factor	Pvalue	Padjust	First Category	Second Category	Gene_ids	Gene_names
11	map05214	Glioma	KEGG PATHWAY	11/1477	73/14104	0.15068493	0.138	0.37855	Human Diseases	Cancer: specific types	ENSGALG000000028005|ENSGALG00000051375|ENSGALG00000002583|ENSGALG00000037943|ENSGALG00000003642|ENSGALG00000012178|ENSGALG00000029968|ENSGALG00000014786|ENSGALG00000005011|ENSGALG00000001319|ENSGALG00000024024	GADD45G;;PIK3CD;PRKCB;PDGFA;PDGFB;GADD45B;PIK3R1;;CAMK1G;TGFA

Num	Pathway id	Description	Database	Ratio_in_study	Ratio_in_pop	Rich factor	Pvalue	Padjust	First Category	Second Category	Gene_ids	Gene_names
37	map05169	Epstein-Barr virus infection	KEGG PATHWAY	37/1477	295/14104	0.12542373	0.1412	0.38435	Human Diseases	Infectious disease: viral	ENSGALG000000028015|ENSGALG00000008936|ENSGALG00000005653|ENSGALG00000015062|ENSGALG00000042838|ENSGALG00000003485|ENSGALG00000005759|ENSGALG00000039080|ENSGALG00000030940|ENSGALG00000002583|ENSGALG00000007651|ENSGALG00000014297|ENSGALG00000002938	HES1;;NFKB2;;TRAF2;CCND3;IFNW1;CD44;BLB2;PIK3CD;STAT1;IRF7;;IL6;CASP18;TNFAIP3;IKBKE;PIK3R1;MAPK10;STAT2;;JAK3;HES4;GADD45G;TAP1;GADD45B;NFKBIE;TRAF3;MAP2K6;CASP8;DMB1;CCNA2;EIF2AK2;;;NFKBIA;BID

Num	Pathway id	Description	Database	Ratio_in_study	Ratio_in_pop	Rich factor	Pvalue	Padjust	First Category	Second Category	Gene_ids	Gene_names
14	map04658	Th1 and Th2 cell differentiation	KEGG PATHWAY	14/1477	98/14104	0.14285714	0.1426	0.3848	Organismal Systems	Immune system	ENSGALG000000030940|ENSGALG00000029270|ENSGALG00000011182|ENSGALG00000007651|ENSGALG00000027864|ENSGALG00000000162|ENSGALG00000012472|ENSGALG00000011109|ENSGALG00000047000|ENSGALG00000035447|ENSGALG00000044525|ENSGALG00000028037|ENSGALG00000004247	BLB2;GATA3;DLL1;STAT1;NFKBIA;DMB1;IL2RB;MAPK10;;;NFKBIE;FOS;IL2RA;JAK3

Num	Pathway id	Description	Database	Ratio_in_study	Ratio_in_pop	Rich factor	Pvalue	Padjust	First Category	Second Category	Gene_ids	Gene_names
7	map00140	Steroid hormone biosynthesis	KEGG PATHWAY	7/1477	42/14104	0.16666667	0.1445	0.38702	Metabolism	Lipid metabolism	ENSGALG00000012834|ENSGALG00000004436|ENSGALG00000037852|ENSGALG00000011894|ENSGALG00000010625|ENSGALG00000025822|ENSGALG00000011805	AKR1D1;CYP3A4;HSD17B7;CYP2D6;SRD5A2;CYP1B1;

Num	Pathway id	Description	Database	Ratio_in_study	Ratio_in_pop	Rich factor	Pvalue	Padjust	First Category	Second Category	Gene_ids	Gene_names
23	map04640	Hematopoietic cell lineage	KEGG PATHWAY	23/1477	175/14104	0.13142857	0.1501	0.39855	Organismal Systems	Immune system	ENSGALG00000010331|ENSGALG00000039080|ENSGALG00000030940|ENSGALG00000029940|ENSGALG00000031149|ENSGALG00000030907|ENSGALG00000010915|ENSGALG00000013372|ENSGALG00000014903|ENSGALG00000046156|ENSGALG00000008439|ENSGALG00000005725|ENSGALG0000002395	MME;CD44;BLB2;IL1B;;CSF3;IL6;IL7R;ITGA2;;CD36;CSF1R;CR1L;;CSF2RA;ITGB3;KITLG;DMB1;IL11;IL7;;IL2RA;CD34

Num	Pathway id	Description	Database	Ratio_in_study	Ratio_in_pop	Rich factor	Pvalue	Padjust	First Category	Second Category	Gene_ids	Gene_names
22	map04514	Cell adhesion molecules	KEGG PATHWAY	22/1477	168/14104	0.13095238	0.1605	0.41307	Environmental Information Processing	Signaling molecules and interaction	ENSGALG000000091 07|ENSGALG00000031637|ENSGALG00000026862|ENSGALG00000015132|ENSGALG00000031794|ENSGALG00000015032|ENSGALG00000030940|ENSGALG00000009514|ENSGALG00000003677|ENSGALG00000001472|ENSGALG00000005257|ENSGALG00000029378|ENSGALG0000003177	NRXN1;ICOSLG;CLDN1;CDH2;;;BLB2;NRCAM;NTNG2;JAM3;;ITGA9;NFASC;;CADM3;NEGR1;;SDC4;PECAM1;DMB1;NRXN3;CD34

Num	Pathway id	Description	Database	Ratio_in_study	Ratio_in_pop	Rich factor	Pvalue	Padjust	First Category	Second Category	Gene_ids	Gene_names
19	map04261	Adrenergic signaling in cardiomyocytes	KEGG PATHWAY	19/1477	142/14104	0.13380282	0.1583	0.41372	Organismal Systems	Circulatory system	ENSGALG000000031244|ENSGALG00000026167|ENSGALG00000011117|ENSGALG00000015358|ENSGALG00000008382|ENSGALG00000010812|ENSGALG00000008393|ENSGALG00000035960|ENSGALG00000040035|ENSGALG00000005936|ENSGALG00000043694|ENSGALG00000037138|ENSGALG0000000854	ADCY6;PIK3R5;AGT;MYH15;GNAI1;RYR2;CREB3L1;;ADRB2;AGTR2;RAPGEF4;KCNQ1;SLC8A1;ADRA1A;CACNA1C;RAPGEF3;SLC8A3;TNNC1;

Num	Pathway id	Description	Database	Ratio_in_study	Ratio_in_pop	Rich factor	Pvalue	Padjust	First Category	Second Category	Gene_ids	Gene_names
9	map05221	Acute myeloid leukemia	KEGG PATHWAY	9/1477	59/14104	0.15254237	0.16	0.41496	Human Diseases	Cancer: specific types	ENSGALG00000002583|ENSGALG00000005725|ENSGALG00000016022|ENSGALG00000014786|ENSGALG00000011881|ENSGALG00000006480|ENSGALG00000008127|ENSGALG00000006511|ENSGALG00000045534	PIK3CD;CSF1R;RUNX1;PIK3R1;CCNA2;TCF7;SPI1;BCL2A1;

Num	Pathway id	Description	Database	Ratio_in_study	Ratio_in_pop	Rich factor	Pvalue	Padjust	First Category	Second Category	Gene_ids	Gene_names
13	map04916	Melanogenesis	KEGG PATHWAY	13/1477	92/14104	0.14130435	0.1627	0.41555	Organismal Systems	Endocrine system	ENSGALG00000031244|ENSGALG00000003163|ENSGALG00000016912|ENSGALG00000008382|ENSGALG00000008393|ENSGALG00000028069|ENSGALG00000037943|ENSGALG00000016600|ENSGALG00000011206|ENSGALG00000012735|ENSGALG00000006480|ENSGALG00000004401|ENSGALG00000003416	ADCY6;GNAO1;EDNRB;GNAI1;CREB3L1;WNT16;PRKCB;POMC;KITLG;EDN1;TCF7;WNT11B;WNT5A

Num	Pathway id	Description	Database	Ratio_in_study	Ratio_in_pop	Rich factor	Pvalue	Padjust	First Category	Second Category	Gene_ids	Gene_names
13	map05215	Prostate cancer	KEGG PATHWAY	13/1477	92/14104	0.14130435	0.1627	0.41555	Human Diseases	Cancer: specific types	ENSGALG000000025 83|ENSGALG000000 30879|ENSGALG0000 0006992|ENSGALG00 00000839 3|ENSGALG0000001 4786|ENSGALG0000 0027864|ENSGALG00 00000364 2|ENSGALG0000001 2178|ENSGALG0000 0010625|ENSGALG00 00000648 0|ENSGALG0000001 9063|ENSGALG0000 0051375|ENSGALG00 00002402	PIK3CD;AR;MMP9;CREB3L1;PIK3R1;NFKBIA;PDGFA;PDGFB;SRD5A2;TCF7;MMP10;;TGFA

Num	Pathway id	Description	Database	Ratio_in_study	Ratio_in_pop	Rich factor	Pvalue	Padjust	First Category	Second Category	Gene_ids	Gene_names
7	map05030	Cocaine addiction	KEGG PATHWAY	7/1477	43/14104	0.1627907	0.1581	0.41646	Human Diseases	Substance dependence	ENSGALG00000031244|ENSGALG00000008382|ENSGALG00000001797|ENSGALG00000008393|ENSGALG00000007278|ENSGALG00000027415|ENSGALG00000006576	ADCY6;GNAI1;GPSM1;CREB3L1;GRIN2A;GRIN2C;GRM3

Num	Pathway id	Description	Database	Ratio_in_study	Ratio_in_pop	Rich factor	Pvalue	Padjust	First Category	Second Category	Gene_ids	Gene_names
28	map05166	Human T-cell leukemia virus infection 1	KEGG PATHWAY	28/1477	222/14104	0.12612613	0.1727	0.42159	Human Diseases	Infectious disease: viral	ENSGALG000000031244|ENSGALG00000008153|ENSGALG00000008393|ENSGALG00000003485|ENSGALG00000005653|ENSGALG00000028037|ENSGALG00000017184|ENSGALG00000007669|ENSGALG00000015013|ENSGALG00000005884|ENSGALG00000030940|ENSGALG00000002583|ENSGALG00000002613	ADCY6;;CREB3L1;CCND3;NFKB2;FOS;MMP7;EGR1;MSX1;;BLB2;PIK3CD;CDKN2B;IL6;PIK3R1;MAPK10;JAK3;MSX2;SPI1;;IL15;NFKBIA;DMB1;IL2RB;CCNA2;;IL2RA;TGFB3

Num	Pathway id	Description	Database	Ratio_in_study	Ratio_in_pop	Rich factor	Pvalue	Padjust	First Category	Second Category	Gene_ids	Gene_names
21	map04921	Oxytocin signaling pathway	KEGG PATHWAY	21/1477	161/14104	0.13043478	0.1718	0.42242	Organismal Systems	Endocrine system	ENSGALG000000031244|ENSGALG00000003163|ENSGALG00000037943|ENSGALG00000014645|ENSGALG00000008382|ENSGALG00000010812|ENSGALG00000037869|ENSGALG00000000336|ENSGALG00000003149|ENSGALG00000010826|ENSGALG00000028037|ENSGALG00000001181|ENSGALG0000004274	ADCY6;GNAO1;PRKCB;MEF2C;GNAI1;RYR2;;PPP1R12B;;PRKAA2;FOS;KCNJ5;RGS2;NPPA;ITPR2;CACNA1C;PIK3R5;KCNJ4;PTGS2;CAMK1G;
4	map00770	Pantothenate and CoA biosynthesis	KEGG PATHWAY	4/1477	21/14104	0.19047619	0.1712	0.42408	Metabolism	Metabolism of cofactors and vitamins	ENSGALG000000034397|ENSGALG00000013177|ENSGALG00000048781|ENSGALG000000013993	;BCAT1;;VNN1

Num	Pathway id	Description	Database	Ratio_in_study	Ratio_in_pop	Rich factor	Pvalue	Padjust	First Category	Second Category	Gene_ids	Gene_names
31	map05168	Herpes simplex virus 1 infection	KEGG PATHWAY	31/1477	248/14104	0.125	0.1708	0.42628	Human Diseases	Infectious disease: viral	ENSGALG00000030661|ENSGALG00000015062|ENSGALG00000017186|ENSGALG00000001565|ENSGALG00000005759|ENSGALG00000045534|ENSGALG00000030940|ENSGALG00000029940|ENSGALG00000055127|ENSGALG00000007651|ENSGALG00000014297|ENSGALG00000029381|ENSGALG00000001091	STAT2;;BIRC3;C5;IFNW1;;BLB2;IL1B;;STAT1;IRF7;;IL6;CASP18;IKBKE;PIK3R1;EIF2AK2;IFIH1;;TMEM173;TAP1;PIK3CD;;TRAF3;ITGB3;CASP8;SOCS3;DMB1;TRAF2;NFKBIA;BID

Num	Pathway id	Description	Database	Ratio_in_study	Ratio_in_pop	Rich factor	Pvalue	Padjust	First Category	Second Category	Gene_ids	Gene_names
10	map01524	Platinum drug resistance	KEGG PATHWAY	10/1477	68/14104	0.14705882	0.1703	0.42828	Human Diseases	Drug resistance: antineoplastic	ENSGALG000000016325|ENSGALG00000016324|ENSGALG00000002583|ENSGALG00000016322|ENSGALG00000008355|ENSGALG00000008346|ENSGALG00000014786|ENSGALG00000017186|ENSGALG00000013039|ENSGALG00000035325	GSTA3;GSTA4;PIK3CD;;CASP8;CASP18;PIK3R1;BIRC3;BID;PMAIP1
8	map04979	Cholesterol metabolism	KEGG PATHWAY	8/1477	54/14104	0.14814815	0.1998	0.45115	Organismal Systems	Digestive system	ENSGALG00000007114|ENSGALG00000008439|ENSGALG00000023824|ENSGALG00000010891|ENSGALG00000036686|ENSGALG00000000619|ENSGALG00000030920|ENSGALG00000028928	APOA1;CD36;;ABCB11;PLTP;ANGPTL4;APOC3;LCAT

Num	Pathway id	Description	Database	Ratio_in_study	Ratio_in_pop	Rich factor	Pvalue	Padjust	First Category	Second Category	Gene_ids	Gene_names
7	map00514	Other types of O-glycan biosynthesis	KEGG PATHWAY	7/1477	45/14104	0.15555556	0.1867	0.4524	Metabolism	Glycan biosynthesis and metabolism	ENSGALG00000006233|ENSGALG00000038320|ENSGALG00000011235|ENSGALG000000001130|ENSGALG00000007804|ENSGALG00000054784|ENSGALG000000006141	GALNT11;COLGALT2;GALNT15;GALNT17;GXYLT2;;POFUT2

Num	Pathway id	Description	Database	Ratio_in_study	Ratio_in_pop	Rich factor	Pvalue	Padjust	First Category	Second Category	Gene_ids	Gene_names
20	map04217	Necroptosis	KEGG PATHWAY	20/1477	156/14104	0.12820513	0.1996	0.45399	Cellular Processes	Cell growth and death	ENSGALG00000008518|ENSGALG00000029940|ENSGALG00000007651|ENSGALG00000010560|ENSGALG00000007220|ENSGALG00000030661|ENSGALG00000008355|ENSGALG00000015062|ENSGALG00000008346|ENSGALG00000013861|ENSGALG00000017186|ENSGALG00000011109|ENSGALG00000004985	;IL1B;STAT1;EIF2AK2;FTH1;STAT2;CASP8;;CASP18;TNFAIP3;BIRC3;MAPK10;H2AFJ;TNFSF10;IFNW1;;TRAF2;;JAK3;BID

Num	Pathway id	Description	Database	Ratio_in_study	Ratio_in_pop	Rich factor	Pvalue	Padjust	First Category	Second Category	Gene_ids	Gene_names
17	map04371	Apelin signaling pathway	KEGG PATHWAY	17/1477	130/14104	0.13076923	0.1996	0.45711	Environmental Information Processing	Signal transduction	ENSGALG000000031244|ENSGALG00000003149|ENSGALG00000010926|ENSGALG00000014645|ENSGALG00000008382|ENSGALG00000010812|ENSGALG00000026167|ENSGALG00000008177|ENSGALG00000010826|ENSGALG00000008544|ENSGALG00000015082|ENSGALG00000038096|ENSGALG00000001407	ADCY6;;SPP1;MEF2C;GNAI1;RYR2;PIK3R5;NOS1;PRKAA2;SLC8A1;RPS6;NOS2;ITPR2;EGR1;SPHK1;NOV;SLC8A3
1	map00660	C5-Branched dibasic acid metabolism	KEGG PATHWAY	1/1477	2/14104	0.5	0.1985	0.45762	Metabolism	Carbohydrate metabolism	ENSGALG00000016919	ACOD1

Num	Pathway id	Description	Database	Ratio_in_study	Ratio_in_pop	Rich factor	Pvalue	Padjust	First Category	Second Category	Gene_ids	Gene_names
36	map05130	Pathogenic Escherichia coli infection	KEGG PATHWAY	36/1477	297/14104	0.12121212	0.1978	0.45913	Human Diseases	Infectious disease: bacterial	ENSGALG0000008933|ENSGALG00000028037|ENSGALG00000000681|ENSGALG00000007740|ENSGALG00000042838|ENSGALG00000026862|ENSGALG00000008444|ENSGALG00000015729|ENSGALG00000029940|ENSGALG00000000059|ENSGALG00000007650|ENSGALG00000010915|ENSGALG0000000834	CASP7;FOS;PAK1;;TRAF2;CLDN1;TUBAL3;LPAR1;IL1B;TUBB3;SLC9A3R1;IL6;CASP18;;RPS3;TLR5;IL8;MYH1D;MAPK10;WIPF3;;;MYH10;;TUBA3E;TNFSF10;;WASF3;CASP8;CYFIP2;;IL8L1;CYTH4;BAIAP2L1;;NFKBIA

Num	Pathway id	Description	Database	Ratio_in_study	Ratio_in_pop	Rich factor	Pvalue	Padjust	First Category	Second Category	Gene_ids	Gene_names
16	map04728	Dopaminergic synapse	KEGG PATHWAY	16/1477	122/14104	0.13114754	0.2053	0.46046	Organismal Systems	Nervous system	ENSGALG000000031244|ENSGALG00000003163|ENSGALG00000037943|ENSGALG000000008382|ENSGALG00000008393|ENSGALG00000007278|ENSGALG000000038995|ENSGALG00000012462|ENSGALG00000003149|ENSGALG000000028037|ENSGALG00000001181|ENSGALG00000011109|ENSGALG0000001407	ADCY6;GNAO1;PRKCB;GNAI1;CREB3L1;GRIN2A;GRIA4;KIF5C;;FOS;KCNJ5;MAPK10;ITPR2;CACNA1C;GRIA1;

Num	Pathway id	Description	Database	Ratio_in_study	Ratio_in_pop	Rich factor	Pvalue	Padjust	First Category	Second Category	Gene_ids	Gene_names
19	map04932	Non-alcoholic fatty liver disease	KEGG PATHWAY	19/1477	147/14104	0.1292517	0.1971	0.46084	Human Diseases	Endocrine and metabolic disease	ENSGALG000000028037|ENSGALG00000011668|ENSGALG00000029940|ENSGALG000000032079|ENSGALG00000031518|ENSGALG00000008355|ENSGALG000000027786|ENSGALG00000008933|ENSGALG00000010826|ENSGALG000000046160|ENSGALG00000010915|ENSGALG00000011109|ENSGALG0000001478	FOS;IL8L1;IL1B;CYTB;;CASP8;SOCS3;CASP7;PRKAA2;;IL6;MAPK10;PIK3R1;IL8;SREBF1;TRAF2;CASP18;PIK3CD;BID

Num	Pathway id	Description	Database	Ratio_in_study	Ratio_in_pop	Rich factor	Pvalue	Padjust	First Category	Second Category	Gene_ids	Gene_names
15	map04361	Axon regeneration	KEGG PATHWAY	15/1477	112/14104	0.13392857	0.1921	0.46208	Organismal Systems	Development and regeneration	ENSGALG000000042551|ENSGALG00000002016|ENSGALG00000003163|ENSGALG00000026153|ENSGALG00000006236|ENSGALG00000036883|ENSGALG00000006609|ENSGALG00000011419|ENSGALG00000010826|ENSGALG00000015428|ENSGALG00000011207|ENSGALG00000002583|ENSGALG0000001110	;PRKCD;GNAO1;FOXO6;TPH1;MET;EPHB1;DUSP4;PRKAA2;;DUSP6;PIK3CD;MAPK10;DUSP5;HTR7

Num	Pathway id	Description	Database	Ratio_in_study	Ratio_in_pop	Rich factor	Pvalue	Padjust	First Category	Second Category	Gene_ids	Gene_names
14	map04066	HIF-1 signaling pathway	KEGG PATHWAY	14/1477	104/14104	0.13461538	0.1971	0.46406	Environmental Information Processing	Signal transduction	ENSGALG000000037943|ENSGALG00000021039|ENSGALG00000014786|ENSGALG00000010290|ENSGALG00000010915|ENSGALG00000040369|ENSGALG00000010001|ENSGALG00000012735|ENSGALG00000038096|ENSGALG00000015082|ENSGALG00000004574|ENSGALG00000001840|ENSGALG0000000258	PRKCB;HKDC1;PIK3R1;VEGFA;IL6;PFKFB3;EGLN3;EDN1;NOS2;RPS6;NPPA;TEK;PIK3CD;ANGPT2

Num	Pathway id	Description	Database	Ratio_in_study	Ratio_in_pop	Rich factor	Pvalue	Padjust	First Category	Second Category	Gene_ids	Gene_names
9	map04927	Cortisol synthesis and secretion	KEGG PATHWAY	9/1477	62/14104	0.14516129	0.1969	0.4703	Organismal Systems	Endocrine system	ENSGALG00000005215|ENSGALG00000031244|ENSGALG00000016600|ENSGALG00000011117|ENSGALG00000047769|ENSGALG00000008393|ENSGALG00000003149|ENSGALG00000014071|ENSGALG00000013022	CACNA1H;ADCY6;POMC;AGT;;CREB3L1;;ITPR2;CACNA1C
9	map05031	Amphetamine addiction	KEGG PATHWAY	9/1477	62/14104	0.14516129	0.1969	0.4703	Human Diseases	Substance dependence	ENSGALG00000031244|ENSGALG00000037943|ENSGALG00000008393|ENSGALG00000007278|ENSGALG00000027415|ENSGALG00000038995|ENSGALG00000028037|ENSGALG00000013022|ENSGALG00000004083	ADCY6;PRKCB;CREB3L1;GRIN2A;GRIN2C;GRIA4;FOS;CACNA1C;GRIA1

Num	Pathway id	Description	Database	Ratio_in_study	Ratio_in_pop	Rich factor	Pvalue	Padjust	First Category	Second Category	Gene_ids	Gene_names
11	map04727	GABAergic synapse	KEGG PATHWAY	11/1477	80/14104	0.1375	0.2127	0.47401	Organismal Systems	Nervous system	ENSGALG000000031244|ENSGALG00000003163|ENSGALG00000008382|ENSGALG00000008518|ENSGALG00000041042|ENSGALG00000037943|ENSGALG00000037131|ENSGALG00000014202|ENSGALG00000016744|ENSGALG00000013022|ENSGALG00000050064	ADCY6;GNAO1;GNAI1;;GABRG3;PRKCB;;GABRA4;GABRA5;CACNA1C;
3	map00120	Primary bile acid biosynthesis	KEGG PATHWAY	3/1477	16/14104	0.1875	0.2311	0.50811	Metabolism	Lipid metabolism	ENSGALG00000012834|ENSGALG00000006352|ENSGALG00000040619	AKR1D1;CH25H;BAAT

Num	Pathway id	Description	Database	Ratio_in_study	Ratio_in_pop	Rich factor	Pvalue	Padjust	First Category	Second Category	Gene_ids	Gene_names
12	map04912	GnRH signaling pathway	KEGG PATHWAY	12/1477	90/14104	0.13333333	0.23	0.50899	Organismal Systems	Endocrine system	ENSGALG00000002016|ENSGALG00000031244|ENSGALG00000003149|ENSGALG000000037943|ENSGALG00000004370|ENSGALG00000005884|ENSGALG000000016564|ENSGALG00000011109|ENSGALG00000014071|ENSGALG000000007669|ENSGALG00000000949|ENSGALG00000013022	PRKCD;ADCY6;;PRKCB;MAP2K6;;PTK2B;MAPK10;ITPR2;EGR1;HBEGF;CACNA1C
4	map00340	Histidine metabolism	KEGG PATHWAY	4/1477	24/14104	0.16666667	0.239	0.52197	Metabolism	Amino acid metabolism	ENSGALG000000012377|ENSGALG00000011421|ENSGALG00000019514|ENSGALG000000008229	HNMT;HAL;CNDP2;ALDH7A1
2	map00740	Riboflavin metabolism	KEGG PATHWAY	2/1477	9/14104	0.22222222	0.2415	0.524	Metabolism	Metabolism of cofactors and vitamins	ENSGALG000000034397|ENSGALG00000054345	;RFKL

Num	Pathway id	Description	Database	Ratio_in_study	Ratio_in_pop	Rich factor	Pvalue	Padjust	First Category	Second Category	Gene_ids	Gene_names
10	map03320	PPAR signaling pathway	KEGG PATHWAY	10/1477	74/14104	0.13513514	0.2438	0.52549	Organismal Systems	Endocrine system	ENSGALG00000007114|ENSGALG00000008439|ENSGALG00000017246|ENSGALG00000036686|ENSGALG00000030025|ENSGALG00000037050|ENSGALG00000000619|ENSGALG00000030920|ENSGALG00000000184|ENSGALG00000010628	APOA1;CD36;ME3;PLTP;FABP4;FABP3;ANGPTL4;APOC3;SLC27A6;ACSL1
7	map00280	Valine, leucine and isoleucine degradation	KEGG PATHWAY	7/1477	49/14104	0.14285714	0.2494	0.52749	Metabolism	Amino acid metabolism	ENSGALG00000010211|ENSGALG00000008185|ENSGALG00000040702|ENSGALG00000008229|ENSGALG00000014846|ENSGALG00000013177|ENSGALG00000020876	ALDH6A1;AOX1;BCKDHB;ALDH7A1;OXCT1;BCAT1;AOX2

Num	Pathway id	Description	Database	Ratio_in_study	Ratio_in_pop	Rich factor	Pvalue	Padjust	First Category	Second Category	Gene_ids	Gene_names
12	map04013	MAPK signaling pathway - fly	KEGG PATHWAY	12/1477	92/14104	0.13043478	0.2534	0.52913	Environmental Information Processing	Signal transduction	ENSGALG00000041510|ENSGALG00000034297|ENSGALG00000014124|ENSGALG00000009791|ENSGALG00000012429|ENSGALG00000014913|ENSGALG00000011109|ENSGALG00000000504|ENSGALG00000035017|ENSGALG00000009298|ENSGALG00000012921|ENSGALG00000053321	DUOX2;TBX2;TEC;PROX1;BMP4;ROS1;MAPK10;ETV7;;;ETV6;DOK2

Num	Pathway id	Description	Database	Ratio_in_study	Ratio_in_pop	Rich factor	Pvalue	Padjust	First Category	Second Category	Gene_ids	Gene_names
23	map05414	Dilated cardiomyopathy	KEGG PATHWAY	23/1477	189/14104	0.12169312	0.2529	0.53142	Human Diseases	Cardiovascular disease	ENSGALG000000031244|ENSGALG00000015358|ENSGALG00000016281|ENSGALG000000008544|ENSGALG00000053107|ENSGALG00000052907|ENSGALG000000009400|ENSGALG00000042388|ENSGALG00000010812|ENSGALG000000051776|ENSGALG00000048310|ENSGALG00000014903|ENSGALG0000001302	ADCY6;MYH15;DMD;SLC8A1;;;SLC8A3;;RYR2;;;ITGA2;CACNA1C;;TNNC1;AGT;;SGCD;ITGB3;SGCG;ITGA9;;TGFB3

Num	Pathway id	Description	Database	Ratio_in_study	Ratio_in_pop	Rich factor	Pvalue	Padjust	First Category	Second Category	Gene_ids	Gene_names
19	map00230	Purine metabolism	KEGG PATHWAY	19/1477	153/14104	0.12418301	0.2491	0.53364	Metabolism	Nucleotide metabolism	ENSGALG000000031244|ENSGALG00000008179|ENSGALG00000002932|ENSGALG000000053647|ENSGALG00000016442|ENSGALG00000037029|ENSGALG000000011838|ENSGALG00000008936|ENSGALG00000037869|ENSGALG000000007172|ENSGALG00000027565|ENSGALG00000039935|ENSGALG0000003165	ADCY6;NT5C2;NME2;;RRM2;;GUCY2C;;;FHIT;;PDE1C;AK5;ENTPD2;NT5C3B;;NT5M;PDE4B;ADA

Num	Pathway id	Description	Database	Ratio_in_study	Ratio_in_pop	Rich factor	Pvalue	Padjust	First Category	Second Category	Gene_ids	Gene_names
19	map05034	Alcoholism	KEGG PATHWAY	19/1477	153/14104	0.12418301	0.2491	0.53364	Human Diseases	Substance dependence	ENSGALG000000014182|ENSGALG00000003163|ENSGALG00000008382|ENSGALG000000031244|ENSGALG00000049408|ENSGALG00000051325|ENSGALG000000037322|ENSGALG00000050350|ENSGALG00000027571|ENSGALG000000053281|ENSGALG00000008393|ENSGALG00000049751|ENSGALG0000004712	ADORA2B;GNAO1;GNAI1;ADCY6;HIST1H2B8;;HIST1H46;HIST1H2B7;;PKIA;CREB3L1;;;H2AFJ;GRIN2A;;;;GRIN2C
3	map00053	Ascorbate and aldarate metabolism	KEGG PATHWAY	3/1477	17/14104	0.17647059	0.2604	0.53697	Metabolism	Carbohydrate metabolism	ENSGALG000000011805|ENSGALG00000016724|ENSGALG00000008229	;RGN;ALDH7A1

Num	Pathway id	Description	Database	Ratio_in_study	Ratio_in_pop	Rich factor	Pvalue	Padjust	First Category	Second Category	Gene_ids	Gene_names
20	map05225	Hepatocellular carcinoma	KEGG PATHWAY	20/1477	163/14104	0.12269939	0.2593	0.53808	Human Diseases	Cancer: specific types	ENSGALG00000028005|ENSGALG00000016325|ENSGALG00000037943|ENSGALG00000036883|ENSGALG00000016324|ENSGALG00000028069|ENSGALG00000016322|ENSGALG00000051630|ENSGALG00000035282|ENSGALG00000029968|ENSGALG00000014786|ENSGALG00000006480|ENSGALG00000000440	GADD45G;GSTA3;PRKCB;MET;GSTA4;WNT16;;;IGF2;GADD45B;PIK3R1;TCF7;WNT11B;;;WNT5A;TGFB3;PIK3CD;;TGFA

Num	Pathway id	Description	Database	Ratio_in_study	Ratio_in_pop	Rich factor	Pvalue	Padjust	First Category	Second Category	Gene_ids	Gene_names
11	map01522	Endocrine resistance	KEGG PATHWAY	11/1477	86/14104	0.12790698	0.2875	0.58199	Human Diseases	Drug resistance: antineoplastic	ENSGALG000000031244|ENSGALG00000002583|ENSGALG00000006992|ENSGALG00000011182|ENSGALG00000010891|ENSGALG00000028037|ENSGALG00000014786|ENSGALG00000011109|ENSGALG00000005011|ENSGALG00000000949|ENSGALG00000051375	ADCY6;PIK3CD;MMP9;DLL1;ABCB11;FOS;PIK3R1;MAPK10;;HBEGF;

Num	Pathway id	Description	Database	Ratio_in_study	Ratio_in_pop	Rich factor	Pvalue	Padjust	First Category	Second Category	Gene_ids	Gene_names
11	map05210	Colorectal cancer	KEGG PATHWAY	11/1477	86/14104	0.12790698	0.2875	0.58199	Human Diseases	Cancer: specific types	ENSGALG000000280 05|ENSGALG00000002583|ENSGALG00000006480|ENSGALG000000280 37|ENSGALG00000029968|ENSGALG00000014786|ENSGALG000000035325|ENSGALG00000011109|ENSGALG00000024024|ENSGALG000000012055|ENSGALG00000010346	GADD45G;PIK3CD;TCF7;FOS;GADD45B;PIK3R1;PMAIP1;MAPK10;TGFA;;TGFB3
4	map04950	Maturity onset diabetes of the young	KEGG PATHWAY	4/1477	26/14104	0.15384615	0.2871	0.58481	Human Diseases	Endocrine and metabolic disease	ENSGALG000000280 15|ENSGALG00000030008|ENSGALG00000041154|ENSGALG000000002055	HES1;HHEX;MNX1;HES4

Num	Pathway id	Description	Database	Ratio_in_study	Ratio_in_pop	Rich factor	Pvalue	Padjust	First Category	Second Category	Gene_ids	Gene_names
6	map00380	Tryptophan metabolism	KEGG PATHWAY	6/1477	43/14104	0.13953488	0.2924	0.58481	Metabolism	Amino acid metabolism	ENSGALG000000012418|ENSGALG00000006236|ENSGALG00000008229|ENSGALG00000008185|ENSGALG00000025822|ENSGALG00000020876	KYNU;TPH1;ALDH7A1;AOX1;CYP1B1;AOX2
17	map04550	Signaling pathways regulating pluripotency of stem cells	KEGG PATHWAY	17/1477	139/14104	0.12230216	0.2856	0.58525	Cellular Processes	Cellular community - eukaryotes	ENSGALG00000031482|ENSGALG00000012429|ENSGALG00000041257|ENSGALG00000039474|ENSGALG00000028770|ENSGALG00000028069|ENSGALG00000028095|ENSGALG00000006158|ENSGALG00000028091|ENSGALG00000014786|ENSGALG00000006480|ENSGALG00000004401|ENSGALG00000003416	Pou5f3;BMP4;ACVR1C;ID4;INHBB;WNT16;HOXA1;ACVR2B;MYF5;PIK3R1;TCF7;WNT11B;WNT5A;LEFTY1;PIK3CD;JAK3;ID1

Num	Pathway id	Description	Database	Ratio_in_study	Ratio_in_pop	Rich factor	Pvalue	Padjust	First Category	Second Category	Gene_ids	Gene_names
9	map04730	Long-term depression	KEGG PATHWAY	9/1477	69/14104	0.13043478	0.2944	0.58535	Organismal Systems	Nervous system	ENSGALG00000003149|ENSGALG00000008382|ENSGALG00000008177|ENSGALG00000037943|ENSGALG00000006719|ENSGALG00000003163|ENSGALG00000037131|ENSGALG00000014071|ENSGALG00000004083	;GNAI1;NOS1;PRKCB;GNAZ;GNAO1;;ITPR2;GRIA1

Num	Pathway id	Description	Database	Ratio_in_study	Ratio_in_pop	Rich factor	Pvalue	Padjust	First Category	Second Category	Gene_ids	Gene_names
20	map04390	Hippo signaling pathway	KEGG PATHWAY	20/1477	167/14104	0.11976048	0.2962	0.58536	Environmental Information Processing	Signal transduction	ENSGALG000000038951|ENSGALG00000047554|ENSGALG00000034661|ENSGALG000000002287|ENSGALG00000028069|ENSGALG00000012429|ENSGALG000000012055|ENSGALG00000010412|ENSGALG00000007994|ENSGALG000000027853|ENSGALG00000006480|ENSGALG00000004401|ENSGALG0000000348	RASSF1;GDF7;ID1;CRB1;WNT16;BMP4;;WWTR1;PARD6B;CTNNA2;TCF7;WNT11B;CCND3;SNAI1;BIRC3;SMAD7B;NOV;TGFB3;;WNT5A

Num	Pathway id	Description	Database	Ratio_in_study	Ratio_in_pop	Rich factor	Pvalue	Padjust	First Category	Second Category	Gene_ids	Gene_names
29	map05020	Prion disease	KEGG PATHWAY	29/1477	250/14104	0.116	0.3073	0.58632	Human Diseases	Neurodegenerative disease	ENSGALG000000020 16|ENSGALG00000008393|ENSGALG00000007278|ENSGALG000000027415|ENSGALG00000040586|ENSGALG00000001565|ENSGALG000000007669|ENSGALG00000008444|ENSGALG00000033295|ENSGALG000000003149|ENSGALG00000029940|ENSGALG00000000059|ENSGALG000000001081	PRKCD;CREB3L1;GRIN2A;GRIN2C;TUBA3E;C5;EGR1;TUBAL3;CAV2;;IL1B;TUBB3;RYR2;KIF5C;IL6;PIK3R1;;MAPK10;CACNA1C;CYTB;;LAMC1;;PIK3CD;ITPR2;NCF1C;CAV1;CAV3;ND5

Num	Pathway id	Description	Database	Ratio_in_study	Ratio_in_pop	Rich factor	Pvalue	Padjust	First Category	Second Category	Gene_ids	Gene_names
18	map04934	Cushing syndrome	KEGG PATHWAY	18/1477	150/14104	0.12	0.3062	0.58757	Human Diseases	Endocrine and metabolic disease	ENSGALG000000052 15|ENSGALG00000031244|ENSGALG00000003149|ENSGALG0000001111 7|ENSGALG00000047769|ENSGALG00000008382|ENSGALG0000000839 3|ENSGALG00000004401|ENSGALG00000016600|ENSGALG0000002613 7|ENSGALG00000004860|ENSGALG00000004322|ENSGALG0000000648	CACNA1H;ADCY6;;AGT;;GNAI1;CREB3L1;WNT11B;POMC;CDKN2B;RASD1;;TCF7;ITPR2;CACNA1C;;WNT16;WNT5A
5	map00250	Alanine, aspartate and glutamate metabolism	KEGG PATHWAY	5/1477	35/14104	0.14285714	0.3021	0.58998	Metabolism	Amino acid metabolism	ENSGALG000000333 38|ENSGALG00000008518|ENSGALG00000054772|ENSGALG0000000974 8|ENSGALG00000015057	GPT2;;NAT8L;ASNS;DDO

Num	Pathway id	Description	Database	Ratio_in_study	Ratio_in_pop	Rich factor	Pvalue	Padjust	First Category	Second Category	Gene_ids	Gene_names
20	map04218	Cellular senescence	KEGG PATHWAY	20/1477	168/14104	0.11904762	0.3057	0.59005	Cellular Processes	Cell growth and death	ENSGALG000000028005|ENSGALG00000051375|ENSGALG00000003149|ENSGALG000000002583|ENSGALG00000004370|ENSGALG00000026137|ENSGALG000000010915|ENSGALG00000046160|ENSGALG00000011668|ENSGALG000000029968|ENSGALG00000014786|ENSGALG00000011881|ENSGALG0000002615	GADD45G;;;PIK3CD;MAP2K6;CDKN2B;IL6;;IL8L1;GADD45B;PIK3R1;CCNA2;FOXO6;ITPR2;CCND3;IL8;RASSF5;ZFP36L1;;TGFB3

Num	Pathway id	Description	Database	Ratio_in_study	Ratio_in_pop	Rich factor	Pvalue	Padjust	First Category	Second Category	Gene_ids	Gene_names
28	map04072	Phospholipase D signaling pathway	KEGG PATHWAY	28/1477	240/14104	0.11666667	0.3004	0.5901	Environmental Information Processing	Signal transduction	ENSGALG000000031244|ENSGALG00000003642|ENSGALG00000046160|ENSGALG00000023541|ENSGALG00000006576|ENSGALG00000014711|ENSGALG00000015729|ENSGALG00000002583|ENSGALG00000016998|ENSGALG00000043694|ENSGALG00000012178|ENSGALG00000011206|ENSGALG0000001478	ADCY6;PDGFA;;SPHK1;GRM3;PLPP1;LPAR1;PIK3CD;LPAR6;RAPGEF4;PDGFB;KITLG;PIK3R1;IL8;RAPGEF3;AVP;AGT;PTGFR;AVPR2;;;PTK2B;GAB2;AVPR1B;CYTH4;PIK3R5;;IL8L1
4	map04392	Hippo signaling pathway - multiple species	KEGG PATHWAY	4/1477	27/14104	0.14814815	0.3117	0.59133	Environmental Information Processing	Signal transduction	ENSGALG000000000681|ENSGALG00000038951|ENSGALG00000011823|ENSGALG000000010412	PAK1;RASSF1;FAT4;WWTR1

Num	Pathway id	Description	Database	Ratio_in_study	Ratio_in_pop	Rich factor	Pvalue	Padjust	First Category	Second Category	Gene_ids	Gene_names
8	map04720	Long-term potentiation	KEGG PATHWAY	8/1477	61/14104	0.13114754	0.3055	0.59316	Organismal Systems	Nervous system	ENSGALG000000003149|ENSGALG00000007278|ENSGALG00000027415|ENSGALG000000037943|ENSGALG00000014071|ENSGALG00000013022|ENSGALG000000004083|ENSGALG00000043775	;GRIN2A;GRIN2C;PRKCB;ITPR2;CACNA1C;GRIA1;RAPGEF3
5	map00350	Tyrosine metabolism	KEGG PATHWAY	5/1477	36/14104	0.13888889	0.3235	0.61023	Metabolism	Amino acid metabolism	ENSGALG000000046412|ENSGALG00000020876|ENSGALG00000000893|ENSGALG000000008185|ENSGALG00000006482	;AOX2;TAT;AOX1;FAH

Num	Pathway id	Description	Database	Ratio_in_study	Ratio_in_pop	Rich factor	Pvalue	Padjust	First Category	Second Category	Gene_ids	Gene_names
27	map05131	Shigellosis	KEGG PATHWAY	27/1477	235/14104	0.11489362	0.334	0.62655	Human Diseases	Infectious disease: bacterial	ENSGALG000000020 16|ENSGALG00000026687|ENSGALG00000042838|ENSGALG000000575 9|ENSGALG00000039080|ENSGALG00000035244|ENSGALG000000314 9|ENSGALG00000029940|ENSGALG00000041129|ENSGALG000000513 25|ENSGALG00000043535|ENSGALG00000026153|ENSGALG0000001478	PRKCD;PLCD4;TRAF2;IFNW1;CD44;;;IL1B;TMEM173;;RIPK2;FOXO6;PIK3R1;IL8;MAPK10;TLR5;PLCE1;HKDC1;PIK3CD;ITPR2;TIFA;NFKBIA;;;CYTH4;;IL8L1

Num	Pathway id	Description	Database	Ratio_in_study	Ratio_in_pop	Rich factor	Pvalue	Padjust	First Category	Second Category	Gene_ids	Gene_names
8	map05217	Basal cell carcinoma	KEGG PATHWAY	8/1477	63/14104	0.12698413	0.3378	0.63013	Human Diseases	Cancer: specific types	ENSGALG000000028005|ENSGALG00000028069|ENSGALG00000012429|ENSGALG00000010133|ENSGALG00000029968|ENSGALG00000006480|ENSGALG00000004401|ENSGALG00000034168	GADD45G;WNT16;BMP4;PTCH2;GADD45B;TCF7;WNT11B;WNT5A

Num	Pathway id	Description	Database	Ratio_in_study	Ratio_in_pop	Rich factor	Pvalue	Padjust	First Category	Second Category	Gene_ids	Gene_names
12	map04972	Pancreatic secretion	KEGG PATHWAY	12/1477	99/14104	0.12121212	0.341	0.63253	Organismal Systems	Digestive system	ENSGALG000000068 34|ENSGALG00000031244|ENSGALG00000003149|ENSGALG000000019233|ENSGALG00000014509|ENSGALG00000010812|ENSGALG000000037943|ENSGALG00000029788|ENSGALG00000007959|ENSGALG000000014071|ENSGALG00000038740|ENSGALG00000037138	CPB1;ADCY6;;;BST1;RYR2;PRKCB;CCK;SLC26A3;ITPR2;AMY2A;KCNQ1
3	map00100	Steroid biosynthesis	KEGG PATHWAY	3/1477	20/14104	0.15	0.3501	0.63856	Metabolism	Lipid metabolism	ENSGALG000000037852|ENSGALG00000023824|ENSGALG00000042279	HSD17B7;;CYP24A1

Num	Pathway id	Description	Database	Ratio_in_study	Ratio_in_pop	Rich factor	Pvalue	Padjust	First Category	Second Category	Gene_ids	Gene_names
8	map04976	Bile secretion	KEGG PATHWAY	8/1477	64/14104	0.125	0.3542	0.6391	Organismal Systems	Digestive system	ENSGALG00000031244|ENSGALG00000016326|ENSGALG00000008912|ENSGALG000000030677|ENSGALG00000040619|ENSGALG00000010891|ENSGALG000000004261|ENSGALG00000011805	ADCY6;;ABCB1;ABCG2;BAAT;ABCB11;AQP9;
7	map05213	Endometrial cancer	KEGG PATHWAY	7/1477	55/14104	0.12727273	0.3527	0.63986	Human Diseases	Cancer: specific types	ENSGALG000000028005|ENSGALG00000002583|ENSGALG00000026153|ENSGALG000000029968|ENSGALG00000014786|ENSGALG00000027853|ENSGALG000000006480	GADD45G;PIK3CD;FOXO6;GADD45B;PIK3R1;CTNNA2;TCF7

Num	Pathway id	Description	Database	Ratio_in_study	Ratio_in_pop	Rich factor	Pvalue	Padjust	First Category	Second Category	Gene_ids	Gene_names
29	map05152	Tuberculosis	KEGG PATHWAY	29/1477	255/14104	0.11372549	0.3471	0.64024	Human Diseases	Infectious disease: bacterial	ENSGALG00000037989|ENSGALG00000010915|ENSGALG00000046494|ENSGALG00000023541|ENSGALG00000005759|ENSGALG00000030940|ENSGALG00000029940|ENSGALG00000007651|ENSGALG00000043535|ENSGALG00000015062|ENSGALG00000008346|ENSGALG00000006583|ENSGALG00000001110	IL10RB;IL6;CASP10;SPHK1;IFNW1;BLB2;IL1B;STAT1;RIPK2;;CASP18;LSP1P1;MAPK10;IL10RA;;NOS2;CR1L;VDR;CTSS;;PLK3;HSPA9;CASP8;DMB1;ATP6V0D2;TLR1B;BID;;TGFB3
6	map04978	Mineral absorption	KEGG PATHWAY	6/1477	46/14104	0.13043478	0.3499	0.64183	Organismal Systems	Digestive system	ENSGALG00000033090|ENSGALG00000007220|ENSGALG00000015154|ENSGALG00000007959|ENSGALG00000008544|ENSGALG00000009400	VDR;FTH1;TRPM6;SLC26A3;SLC8A1;SLC8A3

Num	Pathway id	Description	Database	Ratio_in_study	Ratio_in_pop	Rich factor	Pvalue	Padjust	First Category	Second Category	Gene_ids	Gene_names
8	map04918	Thyroid hormone synthesis	KEGG PATHWAY	8/1477	65/14104	0.12307692	0.3706	0.66159	Organismal Systems	Endocrine system	ENSGALG00000031244|ENSGALG00000041510|ENSGALG00000010633|ENSGALG00000008393|ENSGALG00000037943|ENSGALG00000003149|ENSGALG00000004521|ENSGALG00000014071	ADCY6;DUOX2;GPX7;CREB3L1;PRKCB;;GPX3;ITPR2
10	map04970	Salivary secretion	KEGG PATHWAY	10/1477	83/14104	0.12048193	0.3695	0.66305	Organismal Systems	Digestive system	ENSGALG00000031244|ENSGALG00000003149|ENSGALG00000037943|ENSGALG00000014071|ENSGALG00000014509|ENSGALG00000038740|ENSGALG00000008177|ENSGALG00000000469|ENSGALG00000028520|ENSGALG00000040035	ADCY6;;PRKCB;ITPR2;BST1;AMY2A;NOS1;ADRA1A;CST3;ADRB2

Num	Pathway id	Description	Database	Ratio_in_study	Ratio_in_pop	Rich factor	Pvalue	Padjust	First Category	Second Category	Gene_ids	Gene_names
24	map05170	Human immunodeficiency virus 1 infection	KEGG PATHWAY	24/1477	213/14104	0.11267606	0.3837	0.68116	Human Diseases	Infectious disease: viral	ENSGALG000000037943|ENSGALG00000028037|ENSGALG00000000681|ENSGALG00000042838|ENSGALG00000005759|ENSGALG00000003149|ENSGALG00000002583|ENSGALG00000041129|ENSGALG00000029381|ENSGALG00000015062|ENSGALG00000008346|ENSGALG00000014786|ENSGALG00000001110	PRKCB;FOS;PAK1;TRAF2;IFNW1;;PIK3CD;TMEM173;;;CASP18;PIK3R1;MAPK10;;GNAI1;TAP1;GNAO1;AP1S3;ITPR2;MAP2K6;CASP8;PTK2B;NFKBIA;BID
5	map00980	Metabolism of xenobiotics by cytochrome P450	KEGG PATHWAY	5/1477	39/14104	0.12820513	0.3883	0.68578	Metabolism	Xenobiotics biodegradation and metabolism	ENSGALG00000011805|ENSGALG00000016325|ENSGALG00000016322|ENSGALG00000025822|ENSGALG00000016324	;GSTA3;;CYP1B1;GSTA4

Num	Pathway id	Description	Database	Ratio_in_study	Ratio_in_pop	Rich factor	Pvalue	Padjust	First Category	Second Category	Gene_ids	Gene_names
13	map05231	Choline metabolism in cancer	KEGG PATHWAY	13/1477	112/14104	0.11607143	0.391	0.68684	Human Diseases	Cancer: overview	ENSGALG000000037943|ENSGALG00000017103|ENSGALG00000035026|ENSGALG000000003642|ENSGALG00000028037|ENSGALG00000012178|ENSGALG000000007740|ENSGALG00000014786|ENSGALG00000011109|ENSGALG000000015439|ENSGALG00000016804|ENSGALG00000002583|ENSGALG0000000001471	PRKCB;WASF3;SLC22A4;PDGFA;FOS;PDGFB;;PIK3R1;MAPK10;SLC44A1;SLC5A7;PIK3CD;PLPP1
2	map00730	Thiamine metabolism	KEGG PATHWAY	2/1477	13/14104	0.15384615	0.4017	0.70189	Metabolism	Metabolism of cofactors and vitamins	ENSGALG000000031652|ENSGALG00000023542	AK5;ALPL

Num	Pathway id	Description	Database	Ratio_in_study	Ratio_in_pop	Rich factor	Pvalue	Padjust	First Category	Second Category	Gene_ids	Gene_names
10	map05100	Bacterial invasion of epithelial cells	KEGG PATHWAY	10/1477	86/14104	0.11627907	0.4133	0.70724	Human Diseases	Infectious disease: bacterial	ENSGALG00000033295|ENSGALG00000002583|ENSGALG00000036883|ENSGALG00000043287|ENSGALG00000008351|ENSGALG00000014786|ENSGALG00000027853|ENSGALG00000010031|ENSGALG00000026995|ENSGALG00000005011	CAV2;PIK3CD;MET;CAV1;CAV3;PIK3R1;CTNNA2;ARHGAP10;RHOG;

Num	Pathway id	Description	Database	Ratio_in_study	Ratio_in_pop	Rich factor	Pvalue	Padjust	First Category	Second Category	Gene_ids	Gene_names
16	map01240	Biosynthesis of cofactors	KEGG PATHWAY	16/1477	142/14104	0.11267606	0.4177	0.7111	Metabolism	Global and overview maps	ENSGALG00000026757|ENSGALG00000012418|ENSGALG00000054345|ENSGALG00000016724|ENSGALG00000015180|ENSGALG00000012412|ENSGALG00000034344|ENSGALG00000028982|ENSGALG00000031652|ENSGALG00000012200|ENSGALG00000002932|ENSGALG00000013177|ENSGALG00000005298	DHFR;KYNU;RFKL;RGN;PSAT1;MTHFD1L;MTHFD2;CMPK2;AK5;GCH1;NME2;BCAT1;;;MAT1A;ALPL

Num	Pathway id	Description	Database	Ratio_in_study	Ratio_in_pop	Rich factor	Pvalue	Padjust	First Category	Second Category	Gene_ids	Gene_names
14	map04722	Neurotrophin signaling pathway	KEGG PATHWAY	14/1477	124/14104	0.11290323	0.4251	0.7128	Organismal Systems	Nervous system	ENSGALG00000002016|ENSGALG00000005884|ENSGALG00000002583|ENSGALG00000027864|ENSGALG00000043535|ENSGALG00000026153|ENSGALG00000014786|ENSGALG00000040241|ENSGALG00000002556|ENSGALG00000035017|ENSGALG00000044525|ENSGALG00000005011|ENSGALG00000005352	PRKCD;;PIK3CD;NFKBIA;RIPK2;FOXO6;PIK3R1;NTRK3;NGF;;NFKBIE;;NTF3;MAPK10
4	map00790	Folate biosynthesis	KEGG PATHWAY	4/1477	31/14104	0.12903226	0.4107	0.71393	Metabolism	Metabolism of cofactors and vitamins	ENSGALG000000026757|ENSGALG00000012200|ENSGALG00000023542|ENSGALG000000006236	DHFR;GCH1;ALPL;TPH1

Num	Pathway id	Description	Database	Ratio_in_study	Ratio_in_pop	Rich factor	Pvalue	Padjust	First Category	Second Category	Gene_ids	Gene_names
4	map00410	beta-Alanine metabolism	KEGG PATHWAY	4/1477	31/14104	0.12903226	0.4107	0.71393	Metabolism	Metabolism of other amino acids	ENSGALG000000046412|ENSGALG00000010211|ENSGALG00000019514|ENSGALG000000008229	;ALDH6A1;CNDP2;ALDH7A1
4	map04960	Aldosterone-regulated sodium reabsorption	KEGG PATHWAY	4/1477	31/14104	0.12903226	0.4107	0.71393	Organismal Systems	Excretory system	ENSGALG000000014786|ENSGALG00000005608|ENSGALG00000002583|ENSGALG000000037943	PIK3R1;SLC9A3R2;PIK3CD;PRKCB
1	map00524	Neomycin, kanamycin and gentamicin biosynthesis	KEGG PATHWAY	1/1477	5/14104	0.2	0.4249	0.71605	Metabolism	Biosynthesis of other secondary metabolites	ENSGALG000000021039	HKDC1
7	map05230	Central carbon metabolism in cancer	KEGG PATHWAY	7/1477	59/14104	0.11864407	0.4239	0.71796	Human Diseases	Cancer: overview	ENSGALG000000002583|ENSGALG00000036883|ENSGALG00000021039|ENSGALG000000005845|ENSGALG00000014786|ENSGALG00000040241|ENSGALG000000002555	PIK3CD;MET;HKDC1;SLC7A5;PIK3R1;NTRK3;RET

Num	Pathway id	Description	Database	Ratio_in_study	Ratio_in_pop	Rich factor	Pvalue	Padjust	First Category	Second Category	Gene_ids	Gene_names
15	map04611	Platelet activation	KEGG PATHWAY	15/1477	135/14104	0.11111111	0.4449	0.74219	Organismal Systems	Immune system	ENSGALG000000031244|ENSGALG00000003149|ENSGALG00000009641|ENSGALG000000000379|ENSGALG00000035584|ENSGALG00000008382|ENSGALG000000012791|ENSGALG00000048592|ENSGALG00000001314|ENSGALG000000014903|ENSGALG00000014786|ENSGALG00000002583|ENSGALG000000001407	ADCY6;;COL1A2;ITGB3;COL3A1;GNAI1;TBXAS1;;PTGS1;ITGA2;PIK3R1;PIK3CD;ITPR2;PIK3R5;P2RY1
3	map04320	Dorso-ventral axis formation	KEGG PATHWAY	3/1477	24/14104	0.125	0.4669	0.77509	Organismal Systems	Development and regeneration	ENSGALG000000012921|ENSGALG00000000521|ENSGALG00000000504	ETV6;SPIRE2;ETV7
1	map00400	Phenylalanine, tyrosine and tryptophan biosynthesis	KEGG PATHWAY	1/1477	6/14104	0.16666667	0.4851	0.79735	Metabolism	Amino acid metabolism	ENSGALG00000000893	TAT

Num	Pathway id	Description	Database	Ratio_in_study	Ratio_in_pop	Rich factor	Pvalue	Padjust	First Category	Second Category	Gene_ids	Gene_names
6	map03460	Fanconi anemia pathway	KEGG PATHWAY	6/1477	53/14104	0.11320755	0.485	0.80109	Genetic Information Processing	Replication and repair	ENSGALG00000028015|ENSGALG00000054746|ENSGALG00000007155|ENSGALG00000000516|ENSGALG00000024481|ENSGALG00000002055	HES1;;RMI2;FANCA;CENPS;HES4
17	map04310	Wnt signaling pathway	KEGG PATHWAY	17/1477	158/14104	0.10759494	0.4913	0.80345	Environmental Information Processing	Signal transduction	ENSGALG00000023640|ENSGALG00000009241|ENSGALG00000031997|ENSGALG00000003015|ENSGALG00000030030|ENSGALG00000028069|ENSGALG00000037943|ENSGALG00000004401|ENSGALG00000006480|ENSGALG00000017184|ENSGALG00000002900|ENSGALG00000011109|ENSGALG0000000102	RSPO4;SFRP2;SFRP4;SERPINF1;RSPO2;WNT16;PRKCB;WNT11B;TCF7;MMP7;NOTUM;MAPK10;;CCND3;RSPO1;SFRP1;WNT5A

Num	Pathway id	Description	Database	Ratio_in_study	Ratio_in_pop	Rich factor	Pvalue	Padjust	First Category	Second Category	Gene_ids	Gene_names
25	map04810	Regulation of actin cytoskeleton	KEGG PATHWAY	25/1477	235/14104	0.10638298	0.4984	0.81116	Cellular Processes	Cell motility	ENSGALG000000025748|ENSGALG00000000681|ENSGALG00000014903|ENSGALG000000002583|ENSGALG00000002193|ENSGALG00000011080|ENSGALG000000012178|ENSGALG00000014786|ENSGALG00000002203|ENSGALG000000027323|ENSGALG00000016866|ENSGALG00000015729|ENSGALG0000000118	FGF9;PAK1;ITGA2;PIK3CD;;BDKRB2;PDGFB;PIK3R1;FGF18;MYH1D;FGF14;LPAR1;MYH10;CHRM4;FGF4;FGF13;ITGB3;IQGAP3;PPP1R12B;BDKRB1;CYFIP2;PDGFA;ITGA9;GSN;IQGAP2

Num	Pathway id	Description	Database	Ratio_in_study	Ratio_in_pop	Rich factor	Pvalue	Padjust	First Category	Second Category	Gene_ids	Gene_names
7	map04370	VEGF signaling pathway	KEGG PATHWAY	7/1477	64/14104	0.109375	0.5113	0.82003	Environmental Information Processing	Signal transduction	ENSGALG00000002583|ENSGALG00000010290|ENSGALG00000037943|ENSGALG00000014786|ENSGALG00000023541|ENSGALG00000013907|ENSGALG00000033635	PIK3CD;VEGFA;PRKCB;PIK3R1;SPHK1;KDR;PTGS2
4	map05216	Thyroid cancer	KEGG PATHWAY	4/1477	35/14104	0.11428571	0.5065	0.82022	Human Diseases	Cancer: specific types	ENSGALG00000028005|ENSGALG00000029968|ENSGALG00000006480|ENSGALG00000002555	GADD45G;GADD45B;TCF7;RET
2	map00450	Selenocompound metabolism	KEGG PATHWAY	2/1477	16/14104	0.125	0.511	0.82349	Metabolism	Metabolism of other amino acids	ENSGALG00000014464|ENSGALG00000011331	MTR;CTH
3	map00601	Glycosphingolipid biosynthesis - lacto and neolacto series	KEGG PATHWAY	3/1477	26/14104	0.11538462	0.5218	0.825	Metabolism	Glycan biosynthesis and metabolism	ENSGALG00000007003|ENSGALG00000028803|ENSGALG00000048035	;B3GNT4;GCNT2

Num	Pathway id	Description	Database	Ratio_in_study	Ratio_in_pop	Rich factor	Pvalue	Padjust	First Category	Second Category	Gene_ids	Gene_names
11	map05235	PD-L1 expression and PD-1 checkpoint pathway in cancer	KEGG PATHWAY	11/1477	103/14104	0.10679612	0.52	0.83	Human Diseases	Cancer: overview	ENSGALG000000005884|ENSGALG00000002583|ENSGALG00000007651|ENSGALG000000052425|ENSGALG00000004370|ENSGALG00000027864|ENSGALG000000028037|ENSGALG00000014786|ENSGALG00000035017|ENSGALG000000044525|ENSGALG00000015032	;PIK3CD;STAT1;BATF3;MAP2K6;NFKBIA;FOS;PIK3R1;;NFKBIE;

Num	Pathway id	Description	Database	Ratio_in_study	Ratio_in_pop	Rich factor	Pvalue	Padjust	First Category	Second Category	Gene_ids	Gene_names
11	map00564	Glycerophospholipid metabolism	KEGG PATHWAY	11/1477	103/14104	0.10679612	0.52	0.83	Metabolism	Lipid metabolism	ENSGALG00000019233|ENSGALG00000007251|ENSGALG00000044574|ENSGALG00000016456|ENSGALG00000003574|ENSGALG00000012061|ENSGALG00000004300|ENSGALG00000049891|ENSGALG00000041787|ENSGALG00000028928|ENSGALG00000014711	;PCYT2;PLPP5;LPIN1;LPCAT2;;PTDSS2;GPD1;PLA2G15;LCAT;PLPP1
1	map00440	Phosphonate and phosphinate metabolism	KEGG PATHWAY	1/1477	7/14104	0.14285714	0.5391	0.83632	Metabolism	Metabolism of other amino acids	ENSGALG00000007251	PCYT2

Num	Pathway id	Description	Database	Ratio_in_study	Ratio_in_pop	Rich factor	Pvalue	Padjust	First Category	Second Category	Gene_ids	Gene_names
9	map04211	Longevity regulating pathway	KEGG PATHWAY	9/1477	85/14104	0.10588235	0.5383	0.83904	Organismal Systems	Aging	ENSGALG000000031244|ENSGALG00000002583|ENSGALG00000008393|ENSGALG000000026153|ENSGALG00000008347|ENSGALG00000037603|ENSGALG000000014786|ENSGALG00000010826|ENSGALG00000015259	ADCY6;PIK3CD;CREB3L1;FOXO6;ATG13;SESN2;PIK3R1;PRKAA2;RB1CC1

Num	Pathway id	Description	Database	Ratio_in_study	Ratio_in_pop	Rich factor	Pvalue	Padjust	First Category	Second Category	Gene_ids	Gene_names
36	map05010	Alzheimer disease	KEGG PATHWAY	36/1477	345/14104	0.10434783	0.5358	0.83905	Human Diseases	Neurodegenerative disease	ENSGALG000000010331|ENSGALG00000007278|ENSGALG00000027415|ENSGALG000000008177|ENSGALG00000042838|ENSGALG00000015259|ENSGALG000000008444|ENSGALG00000003149|ENSGALG00000029940|ENSGALG000000000059|ENSGALG00000012462|ENSGALG00000010915|ENSGALG0000000834	MME;GRIN2A;GRIN2C;NOS1;TRAF2;RB1CC1;TUBAL3;;IL1B;TUBB3;KIF5C;IL6;CASP18;ATG13;PIK3R1;;MAPK10;CACNA1C;BACE2;EIF2AK2;CYTB;CASP7;WNT16;;;PIK3CD;ITPR2;TUBA3E;WNT5A;BACE1;CASP8;ND5;NOS2;WNT11B;PTGS2;BID

Num	Pathway id	Description	Database	Ratio_in_study	Ratio_in_pop	Rich factor	Pvalue	Padjust	First Category	Second Category	Gene_ids	Gene_names
15	map05017	Spinocerebellar ataxia	KEGG PATHWAY	15/1477	144/14104	0.10416667	0.5492	0.84025	Human Diseases	Neurodegenerative disease	ENSGALG00000003149|ENSGALG00000002583|ENSGALG00000007278|ENSGALG00000027415|ENSGALG00000037943|ENSGALG00000008347|ENSGALG00000037131|ENSGALG00000004083|ENSGALG00000014786|ENSGALG00000042838|ENSGALG00000011109|ENSGALG00000014071|ENSGALG00000004621	;PIK3CD;GRIN2A;GRIN2C;PRKCB;ATG13;;GRIA1;PIK3R1;TRAF2;MAPK10;ITPR2;;RB1CC1;BEAN1
2	map00360	Phenylalanine metabolism	KEGG PATHWAY	2/1477	17/14104	0.11764706	0.5444	0.8406	Metabolism	Amino acid metabolism	ENSGALG00000046412|ENSGALG00000000893	;TAT
2	map00910	Nitrogen metabolism	KEGG PATHWAY	2/1477	17/14104	0.11764706	0.5444	0.8406	Metabolism	Energy metabolism	ENSGALG00000008518|ENSGALG00000033428	;CA13

Num	Pathway id	Description	Database	Ratio_in_study	Ratio_in_pop	Rich factor	Pvalue	Padjust	First Category	Second Category	Gene_ids	Gene_names
5	map04961	Endocrine and other factor-regulated calcium reabsorption	KEGG PATHWAY	5/1477	46/14104	0.10869565	0.5353	0.84232	Organismal Systems	Excretory system	ENSGALG000000033090|ENSGALG00000008544|ENSGALG00000037943|ENSGALG00000009400|ENSGALG00000011080	VDR;SLC8A1;PRKCB;SLC8A3;BDKRB2
8	map04520	Adherens junction	KEGG PATHWAY	8/1477	77/14104	0.1038961	0.5645	0.85966	Cellular Processes	Cellular community - eukaryotes	ENSGALG000000036883|ENSGALG00000017103|ENSGALG00000010049|ENSGALG00000007740|ENSGALG00000027853|ENSGALG00000006480|ENSGALG00000008018|ENSGALG00000012055	MET;WASF3;PTPRB;;CTNNA2;TCF7;SNAI1;

Num	Pathway id	Description	Database	Ratio_in_study	Ratio_in_pop	Rich factor	Pvalue	Padjust	First Category	Second Category	Gene_ids	Gene_names
25	map04145	Phagosome	KEGG PATHWAY	25/1477	243/14104	0.10288066	0.5691	0.86274	Cellular Processes	Transport and catabolism	ENSGALG000000008177|ENSGALG00000009722|ENSGALG00000040586|ENSGALG000000009626|ENSGALG00000008444|ENSGALG00000030940|ENSGALG000000000769|ENSGALG00000000059|ENSGALG00000003283|ENSGALG000000020754|ENSGALG00000029381|ENSGALG00000014903|ENSGALG00000001132	NOS1;DYNC1I1;TUBA3E;THBS1;TUBAL3;BLB2;RAB7B;TUBB3;;ATP6V0E2;;ITGA2;;THBS2;SFTPA2;CD36;TAP1;;CTSS;NCF1C;ITGB3;COLEC12;DMB1;ATP6V0D2;
4	map00500	Starch and sucrose metabolism	KEGG PATHWAY	4/1477	38/14104	0.10526316	0.5734	0.86537	Metabolism	Carbohydrate metabolism	ENSGALG000000034397|ENSGALG00000010899|ENSGALG00000021039|ENSGALG000000038740	;G6PC2;HKDC1;AMY2A

Num	Pathway id	Description	Database	Ratio_in_study	Ratio_in_pop	Rich factor	Pvalue	Padjust	First Category	Second Category	Gene_ids	Gene_names
9	map04012	ErbB signaling pathway	KEGG PATHWAY	9/1477	88/14104	0.10227273	0.5816	0.86973	Environmental Information Processing	Signal transduction	ENSGALG00000002583|ENSGALG00000037943|ENSGALG00000000681|ENSGALG00000014786|ENSGALG00000011109|ENSGALG00000005011|ENSGALG00000000949|ENSGALG00000033641|ENSGALG00000024024	PIK3CD;PRKCB;PAK1;PIK3R1;MAPK10;;HBEGF;NRG2;TGFA

Num	Pathway id	Description	Database	Ratio_in_study	Ratio_in_pop	Rich factor	Pvalue	Padjust	First Category	Second Category	Gene_ids	Gene_names
18	map05323	Rheumatoid arthritis	KEGG PATHWAY	18/1477	176/14104	0.10227273	0.5792	0.87018	Human Diseases	Immune disease	ENSGALG00000009870|ENSGALG00000029940|ENSGALG00000010915|ENSGALG00000030940|ENSGALG00000000162|ENSGALG00000010290|ENSGALG00000003003|ENSGALG00000046160|ENSGALG00000028037|ENSGALG00000054976|ENSGALG00000034294|ENSGALG00000020754|ENSGALG00000001906	IL15;IL1B;IL6;BLB2;DMB1;VEGFA;CCL20;;FOS;IL11;ATP6V0D2;ATP6V0E2;MMP10;IL8;;TEK;IL8L1;TGFB3

Num	Pathway id	Description	Database	Ratio_in_study	Ratio_in_pop	Rich factor	Pvalue	Padjust	First Category	Second Category	Gene_ids	Gene_names
12	map04071	Sphingolipid signaling pathway	KEGG PATHWAY	12/1477	118/14104	0.10169492	0.5876	0.87479	Environmental Information Processing	Signal transduction	ENSGALG000000002583|ENSGALG00000000168|ENSGALG00000008382|ENSGALG000000037943|ENSGALG00000011080|ENSGALG00000054619|ENSGALG000000010902|ENSGALG00000014786|ENSGALG00000042838|ENSGALG000000011109|ENSGALG00000023541|ENSGALG00000013039	PIK3CD;ADORA1;GNAI1;PRKCB;BDKRB2;GAB2;CERS6;PIK3R1;TRAF2;MAPK10;SPHK1;BID

Num	Pathway id	Description	Database	Ratio_in_study	Ratio_in_pop	Rich factor	Pvalue	Padjust	First Category	Second Category	Gene_ids	Gene_names
8	map04212	Longevity regulating pathway - worm	KEGG PATHWAY	8/1477	79/14104	0.10126582	0.5945	0.87717	Organismal Systems	Aging	ENSGALG000000016325|ENSGALG00000016324|ENSGALG00000002583|ENSGALG000000016322|ENSGALG00000004370|ENSGALG00000002363|ENSGALG000000026153|ENSGALG00000011109	GSTA3;GSTA4;PIK3CD;;MAP2K6;HSPA9;FOXO6;MAPK10
6	map04330	Notch signaling pathway	KEGG PATHWAY	6/1477	59/14104	0.10169492	0.5931	0.87913	Environmental Information Processing	Signal transduction	ENSGALG000000028015|ENSGALG00000011182|ENSGALG00000035031|ENSGALG000000012075|ENSGALG00000047000|ENSGALG00000002055	HES1;DLL1;HEY1;DTX3L;;HES4

Num	Pathway id	Description	Database	Ratio_in_study	Ratio_in_pop	Rich factor	Pvalue	Padjust	First Category	Second Category	Gene_ids	Gene_names
7	map04971	Gastric acid secretion	KEGG PATHWAY	7/1477	70/14104	0.1	0.6092	0.89497	Organismal Systems	Digestive system	ENSGALG000000031244|ENSGALG00000003149|ENSGALG00000008382|ENSGALG000000037943|ENSGALG00000014071|ENSGALG00000004418|ENSGALG000000037138	ADCY6;;GNAI1;PRKCB;ITPR2;SSTR2;KCNQ1

Num	Pathway id	Description	Database	Ratio_in_study	Ratio_in_pop	Rich factor	Pvalue	Padjust	First Category	Second Category	Gene_ids	Gene_names
15	map05140	Leishmaniasis	KEGG PATHWAY	15/1477	150/14104	0.1	0.6149	0.89938	Human Diseases	Infectious disease: parasitic	ENSGALG000000030940|ENSGALG00000052177|ENSGALG00000037943|ENSGALG000000007651|ENSGALG00000023950|ENSGALG00000027864|ENSGALG000000005843|ENSGALG00000000162|ENSGALG00000028037|ENSGALG000000032335|ENSGALG00000029940|ENSGALG00000038096|ENSGALG0000000118	BLB2;;PRKCB;STAT1;CR1L;NFKBIA;EEF1A2;DMB1;FOS;MARCKSL1;IL1B;NOS2;NCF1C;PTGS2;TGFB3

Num	Pathway id	Description	Database	Ratio_in_study	Ratio_in_pop	Rich factor	Pvalue	Padjust	First Category	Second Category	Gene_ids	Gene_names
12	map04152	AMPK signaling pathway	KEGG PATHWAY	12/1477	122/14104	0.09836066	0.6349	0.91652	Environmental Information Processing	Signal transduction	ENSGALG000000008439|ENSGALG00000010899|ENSGALG00000008393|ENSGALG00000010826|ENSGALG00000026153|ENSGALG00000014786|ENSGALG00000011881|ENSGALG00000027188|ENSGALG00000012613|ENSGALG000000000469|ENSGALG00000040369|ENSGALG00000002583	CD36;G6PC2;CREB3L1;PRKAA2;FOXO6;PIK3R1;CCNA2;SREBF1;FBP1;ADRA1A;PFKFB3;PIK3CD
5	map00565	Ether lipid metabolism	KEGG PATHWAY	5/1477	51/14104	0.09803922	0.6296	0.91686	Metabolism	Lipid metabolism	ENSGALG000000010645|ENSGALG00000003574|ENSGALG00000032746|ENSGALG00000019233|ENSGALG00000014711	ENPP6;LPCAT2;ENPP2;;PLPP1

Num	Pathway id	Description	Database	Ratio_in_study	Ratio_in_pop	Rich factor	Pvalue	Padjust	First Category	Second Category	Gene_ids	Gene_names
2	map00531	Glycosaminoglycan degradation	KEGG PATHWAY	2/1477	20/14104	0.1	0.6347	0.92023	Metabolism	Glycan biosynthesis and metabolism	ENSGALG000000112 03|ENSGALG00000029000	HPSE;HYAL3
8	map04721	Synaptic vesicle cycle	KEGG PATHWAY	8/1477	83/14104	0.09638554	0.6512	0.93593	Organismal Systems	Nervous system	ENSGALG00000010187|ENSGALG00000015332|ENSGALG00000004246|ENSGALG00000010098|ENSGALG00000037131|ENSGALG00000034294|ENSGALG00000020754|ENSGALG00000005672	SLC1A1;CPLX1;SLC6A4;SLC6A9;;ATP6V0D2;ATP6V0E2;SLC6A7

Num	Pathway id	Description	Database	Ratio_in_study	Ratio_in_pop	Rich factor	Pvalue	Padjust	First Category	Second Category	Gene_ids	Gene_names
25	map05206	MicroRNAs in cancer	KEGG PATHWAY	25/1477	254/14104	0.0984252	0.66	0.94039	Human Diseases	Cancer: overview	ENSGALG000000018245|ENSGALG00000009626|ENSGALG00000040079|ENSGALG000000007158|ENSGALG00000039080|ENSGALG00000014537|ENSGALG000000002583|ENSGALG00000036883|ENSGALG00000010290|ENSGALG000000012178|ENSGALG00000014786|ENSGALG00000044383|ENSGALG000000003895	gga-mir-155;THBS1;ZFPM2;SOCS1;CD44;BMF;PIK3CD;MET;VEGFA;PDGFB;PIK3R1;MCL1;RASSF1;CYP24A1;MMP16;CYP1B1;ITGB3;;;HMGA2;MMP9;PRKCB;PDGFA;SERPINB5;PTGS2

Num	Pathway id	Description	Database	Ratio_in_study	Ratio_in_pop	Rich factor	Pvalue	Padjust	First Category	Second Category	Gene_ids	Gene_names
12	map04919	Thyroid hormone signaling pathway	KEGG PATHWAY	12/1477	124/14104	0.09677419	0.6575	0.94095	Organismal Systems	Endocrine system	ENSGALG000000025 83|ENSGALG00000000379|ENSGALG00000015358|ENSGALG0000000765 1|ENSGALG00000037943|ENSGALG00000013154|ENSGALG0000002600 5|ENSGALG00000026687|ENSGALG00000014786|ENSGALG0000003952 8|ENSGALG00000035960|ENSGALG00000012429	PIK3CD;ITGB3;MYH15;STAT1;PRKCB;SLCO1C1;DIO2;PLCD4;PIK3R1;PLCE1;;BMP4
3	map04710	Circadian rhythm	KEGG PATHWAY	3/1477	32/14104	0.09375	0.6657	0.94455	Organismal Systems	Environmental adaptation	ENSGALG000000001 04|ENSGALG00000008308|ENSGALG00000010826	;BHLHE40;PRKAA2
1	map00130	Ubiquinone and other terpenoid-quinone biosynthesis	KEGG PATHWAY	1/1477	10/14104	0.1	0.6693	0.94558	Metabolism	Metabolism of cofactors and vitamins	ENSGALG000000008 93	TAT

Num	Pathway id	Description	Database	Ratio_in_study	Ratio_in_pop	Rich factor	Pvalue	Padjust	First Category	Second Category	Gene_ids	Gene_names
14	map04150	mTOR signaling pathway	KEGG PATHWAY	14/1477	147/14104	0.0952381	0.6866	0.96595	Environmental Information Processing	Signal transduction	ENSGALG00000028069|ENSGALG00000015082|ENSGALG00000037603|ENSGALG00000043787|ENSGALG00000037943|ENSGALG00000016456|ENSGALG00000010826|ENSGALG00000005845|ENSGALG00000007964|ENSGALG00000014786|ENSGALG00000014213|ENSGALG00000004401|ENSGALG00000000258	WNT16;RPS6;SESN2;DEPDC6;PRKCB;LPIN1;PRKAA2;SLC7A5;CASTOR1;PIK3R1;PRR5;WNT11B;PIK3CD;WNT5A
5	map04213	Longevity regulating pathway - multiple species	KEGG PATHWAY	5/1477	56/14104	0.08928571	0.7113	0.99229	Organismal Systems	Aging	ENSGALG00000031244|ENSGALG00000014786|ENSGALG00000002583|ENSGALG00000026153|ENSGALG00000010826	ADCY6;PIK3R1;PIK3CD;FOXO6;PRKAA2

Num	Pathway id	Description	Database	Ratio_in_study	Ratio_in_pop	Rich factor	Pvalue	Padjust	First Category	Second Category	Gene_ids	Gene_names
6	map00310	Lysine degradation	KEGG PATHWAY	6/1477	67/14104	0.08955224	0.7162	0.99487	Metabolism	Amino acid metabolism	ENSGALG00000009800|ENSGALG00000038320|ENSGALG00000008229|ENSGALG00000015935|ENSGALG00000019094|ENSGALG00000010653	SETD7;COLGALT2;ALDH7A1;SMYD1;PHF11;SMYD3
9	map04070	Phosphatidylinositol signaling system	KEGG PATHWAY	9/1477	98/14104	0.09183673	0.7102	0.9949	Environmental Information Processing	Signal transduction	ENSGALG00000003149|ENSGALG00000002583|ENSGALG00000026536|ENSGALG00000037943|ENSGALG00000026687|ENSGALG00000014786|ENSGALG00000014071|ENSGALG00000036021|ENSGALG00000039528	;PIK3CD;IP6K3;PRKCB;PLCD4;PIK3R1;ITPR2;MTMR7;PLCE1
2	map03008	Ribosome biogenesis in eukaryotes	KEGG PATHWAY	2/1477	200/14104	0.01	1	1	Genetic Information Processing	Translation	ENSGALG00000040238|ENSGALG00000040680	IMP3;
1	map00920	Sulfur metabolism	KEGG PATHWAY	1/1477	12/14104	0.08333333	0.735	1	Metabolism	Energy metabolism	ENSGALG00000033780	

Num	Pathway id	Description	Database	Ratio_in_study	Ratio_in_pop	Rich factor	Pvalue	Padjust	First Category	Second Category	Gene_ids	Gene_names
19	map05208	Chemical carcinogenesis - reactive oxygen species	KEGG PATHWAY	19/1477	204/14104	0.09313725	0.7397	1	Human Diseases	Cancer: overview	ENSGALG00000016325|ENSGALG00000016324|ENSGALG00000002583|ENSGALG00000036883|ENSGALG00000032079|ENSGALG00000016322|ENSGALG00000014786|ENSGALG00000027864|ENSGALG00000010290|ENSGALG00000026153|ENSGALG00000028037|ENSGALG00000029500|ENSGALG0000000432	GSTA3;GSTA4;PIK3CD;MET;CYTB;;PIK3R1;NFKBIA;VEGFA;FOXO6;FOS;ND5;;MAPK10;CYP1B1;;NCF1C;PRKCD;EPHX4
6	map04137	Mitophagy - animal	KEGG PATHWAY	6/1477	69/14104	0.08695652	0.7425	1	Cellular Processes	Transport and catabolism	ENSGALG00000013818|ENSGALG00000026153|ENSGALG00000013037|ENSGALG00000001525|ENSGALG00000011109|ENSGALG00000000769	CITED2;FOXO6;BCL2L13;;MAPK10;RAB7B

Num	Pathway id	Description	Database	Ratio_in_study	Ratio_in_pop	Rich factor	Pvalue	Padjust	First Category	Second Category	Gene_ids	Gene_names
2	map04966	Collecting duct acid secretion	KEGG PATHWAY	2/1477	25/14104	0.08	0.7533	1	Organismal Systems	Excretory system	ENSGALG000000020754|ENSGALG00000034294	ATP6V0E2;ATP6V0D2
12	map04910	Insulin signaling pathway	KEGG PATHWAY	12/1477	134/14104	0.08955224	0.758	1	Organismal Systems	Endocrine system	ENSGALG00000015082|ENSGALG00000010899|ENSGALG00000021039|ENSGALG00000027786|ENSGALG00000010826|ENSGALG00000014786|ENSGALG00000027188|ENSGALG00000012613|ENSGALG00000007158|ENSGALG00000005011|ENSGALG00000002583|ENSGALG00000011109	RPS6;G6PC2;HKDC1;SOCS3;PRKAA2;PIK3R1;SREBF1;FBP1;SOCS1;;PIK3CD;MAPK10
1	map03450	Non-homologous end-joining	KEGG PATHWAY	1/1477	13/14104	0.07692308	0.7628	1	Genetic Information Processing	Replication and repair	ENSGALG00000049101	

Num	Pathway id	Description	Database	Ratio_in_study	Ratio_in_pop	Rich factor	Pvalue	Padjust	First Category	Second Category	Gene_ids	Gene_names
7	map04146	Peroxisome	KEGG PATHWAY	7/1477	82/14104	0.08536585	0.7686	1	Cellular Processes	Transport and catabolism	ENSGALG000000015057|ENSGALG00000040619|ENSGALG00000010243|ENSGALG00000026239|ENSGALG00000030164|ENSGALG00000010628|ENSGALG00000038096	DDO;BAAT;PRDX1;MPV17;ECH1;ACSL1;NOS2
4	map04975	Fat digestion and absorption	KEGG PATHWAY	4/1477	49/14104	0.08163265	0.769	1	Organismal Systems	Digestive system	ENSGALG00000007114|ENSGALG00000008439|ENSGALG00000019233|ENSGALG00000014711	APOA1;CD36;;PLPP1

Num	Pathway id	Description	Database	Ratio_in_study	Ratio_in_pop	Rich factor	Pvalue	Padjust	First Category	Second Category	Gene_ids	Gene_names
15	map05416	Viral myocarditis	KEGG PATHWAY	15/1477	168/14104	0.08928571	0.7798	1	Human Diseases	Cardiovascular disease	ENSGALG000000030940|ENSGALG00000015358|ENSGALG00000031149|ENSGALG000000003886|ENSGALG00000008355|ENSGALG00000043287|ENSGALG000000008346|ENSGALG00000016281|ENSGALG00000053107|ENSGALG000000000162|ENSGALG00000017122|ENSGALG00000042388|ENSGALG000000005217	BLB2;MYH15;;SGCD;CASP8;CAV1;CASP18;DMD;;DMB1;SGCG;;;;BID

Num	Pathway id	Description	Database	Ratio_in_study	Ratio_in_pop	Rich factor	Pvalue	Padjust	First Category	Second Category	Gene_ids	Gene_names
16	map04666	Fc gamma R-mediated phagocytosis	KEGG PATHWAY	16/1477	179/14104	0.08938547	0.784	1	Organismal Systems	Immune system	ENSGALG000000020 16|ENSGALG00000011172|ENSGALG00000002583|ENSGALG000000032335|ENSGALG00000017103|ENSGALG00000037943|ENSGALG000000023541|ENSGALG00000000681|ENSGALG00000007740|ENSGALG000000014786|ENSGALG00000054619|ENSGALG00000001446|ENSGALG000000004859	PRKCD;;PIK3CD;MARCKSL1;WASF3;PRKCB;SPHK1;PAK1;;PIK3R1;GAB2;GSN;;NCF1C;;PLPP1
6	map00561	Glycerolipid metabolism	KEGG PATHWAY	6/1477	73/14104	0.08219178	0.7899	1	Metabolism	Lipid metabolism	ENSGALG000000008229|ENSGALG00000044574|ENSGALG00000043734|ENSGALG000000016456|ENSGALG00000032736|ENSGALG00000014711	ALDH7A1;PLPP5;LIPG;LPIN1;MGLL;PLPP1

Num	Pathway id	Description	Database	Ratio_in_study	Ratio_in_pop	Rich factor	Pvalue	Padjust	First Category	Second Category	Gene_ids	Gene_names
4	map05332	Graft-versus-host disease	KEGG PATHWAY	4/1477	51/14104	0.07843137	0.7957	1	Human Diseases	Immune disease	ENSGALG00000030940|ENSGALG00000029940|ENSGALG00000010915|ENSGALG00000000162	BLB2;IL1B;IL6;DMB1
4	map04962	Vasopressin-regulated water reabsorption	KEGG PATHWAY	4/1477	51/14104	0.07843137	0.7957	1	Organismal Systems	Excretory system	ENSGALG00000008393|ENSGALG00000014117|ENSGALG00000009497|ENSGALG00000009722	CREB3L1;AVP;AVPR2;DYNC1I1
4	map04214	Apoptosis - fly	KEGG PATHWAY	4/1477	51/14104	0.07843137	0.7957	1	Cellular Processes	Cell growth and death	ENSGALG00000008355|ENSGALG00000008933|ENSGALG00000011109|ENSGALG00000008346	CASP8;CASP7;MAPK10;CASP18
3	map00051	Fructose and mannose metabolism	KEGG PATHWAY	3/1477	40/14104	0.075	0.8046	1	Metabolism	Carbohydrate metabolism	ENSGALG00000040369|ENSGALG00000021039|ENSGALG00000012613	PFKFB3;HKDC1;FBP1
2	map00650	Butanoate metabolism	KEGG PATHWAY	2/1477	28/14104	0.07142857	0.8072	1	Metabolism	Carbohydrate metabolism	ENSGALG00000014846|ENSGALG00000012338	OXCT1;BDH2

Num	Pathway id	Description	Database	Ratio_in_study	Ratio_in_pop	Rich factor	Pvalue	Padjust	First Category	Second Category	Gene_ids	Gene_names
2	map04977	Vitamin digestion and absorption	KEGG PATHWAY	2/1477	28/14104	0.07142857	0.8072	1	Organismal Systems	Digestive system	ENSGALG000000071 14|ENSGALG00000006194	APOA1;SLC52A3
2	map00030	Pentose phosphate pathway	KEGG PATHWAY	2/1477	28/14104	0.07142857	0.8072	1	Metabolism	Carbohydrate metabolism	ENSGALG00000016724|ENSGALG00000012613	RGN;FBP1
11	map04142	Lysosome	KEGG PATHWAY	11/1477	130/14104	0.08461538	0.8132	1	Cellular Processes	Transport and catabolism	ENSGALG00000010199|ENSGALG00000003217|ENSGALG00000023824|ENSGALG00000045196|ENSGALG00000029000|ENSGALG00000004193|ENSGALG00000000775|ENSGALG00000005171|ENSGALG00000008759|ENSGALG00000041787|ENSGALG00000034294	MFSD8;LITAF;;LAPTM4B;HYAL3;ARSG;CTSS;AP1S3;LAMP3;PLA2G15;ATP6V0D2

Num	Pathway id	Description	Database	Ratio_in_study	Ratio_in_pop	Rich factor	Pvalue	Padjust	First Category	Second Category	Gene_ids	Gene_names
6	map00562	Inositol phosphate metabolism	KEGG PATHWAY	6/1477	76/14104	0.07894737	0.8208	1	Metabolism	Carbohydrate metabolism	ENSGALG00000010211|ENSGALG00000002583|ENSGALG00000036021|ENSGALG00000026687|ENSGALG00000039528|ENSGALG00000030344	ALDH6A1;PIK3CD;MTMR7;PLCD4;PLCE1;ISYNA1
11	map04660	T cell receptor signaling pathway	KEGG PATHWAY	11/1477	131/14104	0.08396947	0.8208	1	Organismal Systems	Immune system	ENSGALG000000007356|ENSGALG00000002583|ENSGALG00000014124|ENSGALG00000027864|ENSGALG00000028037|ENSGALG00000000681|ENSGALG00000014786|ENSGALG00000011109|ENSGALG00000044525|ENSGALG00000004398|ENSGALG00000039164	MAP3K8;PIK3CD;TEC;NFKBIA;FOS;PAK1;PIK3R1;MAPK10;NFKBIE;CARD11;

Num	Pathway id	Description	Database	Ratio_in_study	Ratio_in_pop	Rich factor	Pvalue	Padjust	First Category	Second Category	Gene_ids	Gene_names
2	map04136	Autophagy - other	KEGG PATHWAY	2/1477	29/14104	0.06896552	0.8227	1	Cellular Processes	Transport and catabolism	ENSGALG000000277 14|ENSGALG00000008347	ATG10;ATG13
17	map05415	Diabetic cardiomyopathy	KEGG PATHWAY	17/1477	196/14104	0.08673469	0.8273	1	Human Diseases	Cardiovascular disease	ENSGALG00000002016|ENSGALG00000029500|ENSGALG00000009641|ENSGALG00000011117|ENSGALG00000006992|ENSGALG00000032079|ENSGALG00000010812|ENSGALG00000037943|ENSGALG00000014786|ENSGALG00000011109|ENSGALG00000035584|ENSGALG00000009700|ENSGALG0000000118	PRKCD;ND5;COL1A2;AGT;MMP9;CYTB;RYR2;PRKCB;PIK3R1;MAPK10;COL3A1;PDK4;NCF1C;CD36;PIK3CD;;TGFB3
1	map00604	Glycosphingolipid biosynthesis - ganglio series	KEGG PATHWAY	1/1477	16/14104	0.0625	0.8298	1	Metabolism	Glycan biosynthesis and metabolism	ENSGALG00000009027	ST6GALNAC3

Num	Pathway id	Description	Database	Ratio_in_study	Ratio_in_pop	Rich factor	Pvalue	Padjust	First Category	Second Category	Gene_ids	Gene_names
1	map00533	Glycosaminoglycan biosynthesis - keratan sulfate	KEGG PATHWAY	1/1477	16/14104	0.0625	0.8298	1	Metabolism	Glycan biosynthesis and metabolism	ENSGALG00000007701	B3GNT7
4	map05110	Vibrio cholerae infection	KEGG PATHWAY	4/1477	54/14104	0.07407407	0.831	1	Human Diseases	Infectious disease: bacterial	ENSGALG00000037138|ENSGALG00000020754|ENSGALG00000034294|ENSGALG00000015109	KCNQ1;ATP6V0E2;ATP6V0D2;TJP2
6	map04914	Progesterone-mediated oocyte maturation	KEGG PATHWAY	6/1477	78/14104	0.07692308	0.8393	1	Organismal Systems	Endocrine system	ENSGALG00000031244|ENSGALG00000002583|ENSGALG00000008382|ENSGALG00000014786|ENSGALG00000011881|ENSGALG00000011109	ADCY6;PIK3CD;GNAI1;PIK3R1;CCNA2;MAPK10
4	map04913	Ovarian steroidogenesis	KEGG PATHWAY	4/1477	57/14104	0.07017544	0.8611	1	Organismal Systems	Endocrine system	ENSGALG00000037852|ENSGALG00000031244|ENSGALG00000033635|ENSGALG00000025822	HSD17B7;ADCY6;PTGS2;CYP1B1
1	map00061	Fatty acid biosynthesis	KEGG PATHWAY	1/1477	18/14104	0.05555556	0.8636	1	Metabolism	Lipid metabolism	ENSGALG00000010628	ACSL1

Num	Pathway id	Description	Database	Ratio_in_study	Ratio_in_pop	Rich factor	Pvalue	Padjust	First Category	Second Category	Gene_ids	Gene_names
9	map05143	African trypanosomiasis	KEGG PATHWAY	9/1477	116/14104	0.07758621	0.8695	1	Human Diseases	Infectious disease: parasitic	ENSGALG00000007114|ENSGALG00000029940|ENSGALG00000031430|ENSGALG00000037943|ENSGALG00000010915|ENSGALG00000004574|ENSGALG00000052177|ENSGALG00000005257|ENSGALG00000014984	APOA1;IL1B;;PRKCB;IL6;NPPA;;;F2RL1

Num	Pathway id	Description	Database	Ratio_in_study	Ratio_in_pop	Rich factor	Pvalue	Padjust	First Category	Second Category	Gene_ids	Gene_names
15	map04530	Tight junction	KEGG PATHWAY	15/1477	183/14104	0.08196721	0.8739	1	Cellular Processes	Cellular community - eukaryotes	ENSGALG00000016022|ENSGALG00000007650|ENSGALG00000048592|ENSGALG00000010826|ENSGALG00000001472|ENSGALG00000007994|ENSGALG00000000433|ENSGALG00000001183|ENSGALG00000011324|ENSGALG00000011109|ENSGALG00000026862|ENSGALG00000040586|ENSGALG00000001510	RUNX1;SLC9A3R1;;PRKAA2;JAM3;PARD6B;;MYH10;;MAPK10;CLDN1;TUBA3E;TJP2;TUBAL3;MYH1D
2	map00630	Glyoxylate and dicarboxylate metabolism	KEGG PATHWAY	2/1477	33/14104	0.06060606	0.874	1	Metabolism	Carbohydrate metabolism	ENSGALG00000008518|ENSGALG00000052983	;
2	map01040	Biosynthesis of unsaturated fatty acids	KEGG PATHWAY	2/1477	33/14104	0.06060606	0.874	1	Metabolism	Lipid metabolism	ENSGALG00000028142|ENSGALG00000040619	HACD4;BAAT

Num	Pathway id	Description	Database	Ratio_in_study	Ratio_in_pop	Rich factor	Pvalue	Padjust	First Category	Second Category	Gene_ids	Gene_names
7	map04922	Glucagon signaling pathway	KEGG PATHWAY	7/1477	95/14104	0.07368421	0.8811	1	Organismal Systems	Endocrine system	ENSGALG00000003149|ENSGALG00000010899|ENSGALG00000012613|ENSGALG00000008393|ENSGALG00000010826|ENSGALG00000014071|ENSGALG00000011104	;G6PC2;FBP1;CREB3L1;PRKAA2;ITPR2;GCG
2	map03410	Base excision repair	KEGG PATHWAY	2/1477	34/14104	0.05882353	0.8845	1	Genetic Information Processing	Replication and repair	ENSGALG00000050752|ENSGALG00000049101	;
5	map04391	Hippo signaling pathway - fly	KEGG PATHWAY	5/1477	72/14104	0.06944444	0.8851	1	Environmental Information Processing	Signal transduction	ENSGALG00000002287|ENSGALG00000007994|ENSGALG00000034910|ENSGALG00000011823|ENSGALG00000011109	CRB1;PARD6B;ZDHHC18;FAT4;MAPK10
4	map04940	Type I diabetes mellitus	KEGG PATHWAY	4/1477	60/14104	0.06666667	0.8865	1	Human Diseases	Endocrine and metabolic disease	ENSGALG00000030940|ENSGALG00000029940|ENSGALG00000000162|ENSGALG00000027788	BLB2;IL1B;DMB1;CPE

Num	Pathway id	Description	Database	Ratio_in_study	Ratio_in_pop	Rich factor	Pvalue	Padjust	First Category	Second Category	Gene_ids	Gene_names
4	map00010	Glycolysis / Gluconeogenesis	KEGG PATHWAY	4/1477	60/14104	0.06666667	0.8865	1	Metabolism	Carbohydrate metabolism	ENSGALG00000010899|ENSGALG00000021039|ENSGALG00000012613|ENSGALG00000008229	G6PC2;HKDC1;FBP1;ALDH7A1
14	map04650	Natural killer cell mediated cytotoxicity	KEGG PATHWAY	14/1477	175/14104	0.08	0.8881	1	Organismal Systems	Immune system	ENSGALG00000016564|ENSGALG00000037943|ENSGALG00000015062|ENSGALG00000006407|ENSGALG00000000681|ENSGALG00000009179|ENSGALG00000014786|ENSGALG00000035017|ENSGALG00000052177|ENSGALG00000005759|ENSGALG00000005011|ENSGALG00000054104|ENSGALG00000000258	PTK2B;PRKCB;;;PAK1;TNFSF10;PIK3R1;;;IFNW1;;;PIK3CD;BID

Num	Pathway id	Description	Database	Ratio_in_study	Ratio_in_pop	Rich factor	Pvalue	Padjust	First Category	Second Category	Gene_ids	Gene_names
2	map00591	Linoleic acid metabolism	KEGG PATHWAY	2/1477	35/14104	0.05714286	0.8942	1	Metabolism	Lipid metabolism	ENSGALG000000044 36|ENSGALG00000019233	CYP3A4;
2	map00640	Propanoate metabolism	KEGG PATHWAY	2/1477	35/14104	0.05714286	0.8942	1	Metabolism	Carbohydrate metabolism	ENSGALG000000102 11|ENSGALG00000040702	ALDH6A1;BCKDHB
2	map03030	DNA replication	KEGG PATHWAY	2/1477	35/14104	0.05714286	0.8942	1	Genetic Information Processing	Replication and repair	ENSGALG000000507 52|ENSGALG00000049101	;

Num	Pathway id	Description	Database	Ratio_in_study	Ratio_in_pop	Rich factor	Pvalue	Padjust	First Category	Second Category	Gene_ids	Gene_names
20	map04613	Neutrophil extracellular trap formation	KEGG PATHWAY	20/1477	243/14104	0.08230453	0.899	1	Organismal Systems	Immune system	ENSGALG000000027571|ENSGALG00000016590|ENSGALG00000002583|ENSGALG000000000379|ENSGALG00000004261|ENSGALG00000023950|ENSGALG000000049751|ENSGALG00000049408|ENSGALG00000051325|ENSGALG000000037322|ENSGALG00000037943|ENSGALG00000050350|ENSGALG0000000004712	;TLR7;PIK3CD;ITGB3;AQP9;CR1L;;HIST1H2B8;;HIST1H46;PRKCB;HIST1H2B7;;PIK3R1;C5;H2AFJ;NCF1C;;;
3	map00520	Amino sugar and nucleotide sugar metabolism	KEGG PATHWAY	3/1477	49/14104	0.06122449	0.8994	1	Metabolism	Carbohydrate metabolism	ENSGALG000000004614|ENSGALG00000034948|ENSGALG00000021039	NPL;UAP1L1;HKDC1
1	map03430	Mismatch repair	KEGG PATHWAY	1/1477	21/14104	0.04761905	0.9022	1	Genetic Information Processing	Replication and repair	ENSGALG000000050752	

Num	Pathway id	Description	Database	Ratio_in_study	Ratio_in_pop	Rich factor	Pvalue	Padjust	First Category	Second Category	Gene_ids	Gene_names
15	map05322	Systemic lupus erythematosus	KEGG PATHWAY	15/1477	191/14104	0.07853403	0.9092	1	Human Diseases	Immune disease	ENSGALG00000030940|ENSGALG00000001565|ENSGALG00000027571|ENSGALG00000049408|ENSGALG00000051325|ENSGALG00000037322|ENSGALG00000050350|ENSGALG00000049751|ENSGALG00000047126|ENSGALG00000000162|ENSGALG00000049859|ENSGALG00000007278|ENSGALG00000005125	BLB2;C5;;HIST1H2B8;;HIST1H46;HIST1H2B7;;;DMB1;H2AFJ;GRIN2A;;;

Num	Pathway id	Description	Database	Ratio_in_study	Ratio_in_pop	Rich factor	Pvalue	Padjust	First Category	Second Category	Gene_ids	Gene_names
10	map04672	Intestinal immune network for IgA production	KEGG PATHWAY	10/1477	136/14104	0.07352941	0.9148	1	Organismal Systems	Immune system	ENSGALG00000009870|ENSGALG00000008153|ENSGALG00000028341|ENSGALG00000030940|ENSGALG00000031637|ENSGALG00000010915|ENSGALG00000000162|ENSGALG00000000919|ENSGALG00000049589|ENSGALG00000052177	IL15;;;BLB2;ICOSLG;IL6;DMB1;PIGR;;
2	map00071	Fatty acid degradation	KEGG PATHWAY	2/1477	38/14104	0.05263158	0.9189	1	Metabolism	Lipid metabolism	ENSGALG00000010628|ENSGALG00000008229	ACSL1;ALDH7A1
2	map00052	Galactose metabolism	KEGG PATHWAY	2/1477	38/14104	0.05263158	0.9189	1	Metabolism	Carbohydrate metabolism	ENSGALG00000010899|ENSGALG00000021039	G6PC2;HKDC1

Num	Pathway id	Description	Database	Ratio_in_study	Ratio_in_pop	Rich factor	Pvalue	Padjust	First Category	Second Category	Gene_ids	Gene_names
10	map04140	Autophagy - animal	KEGG PATHWAY	10/1477	137/14104	0.0729927	0.919	1	Cellular Processes	Transport and catabolism	ENSGALG00000002016|ENSGALG00000002583|ENSGALG00000043787|ENSGALG00000010826|ENSGALG00000008347|ENSGALG00000014786|ENSGALG00000011109|ENSGALG00000027714|ENSGALG00000015259|ENSGALG00000000769	PRKCD;PIK3CD;DEPDC6;PRKAA2;ATG13;PIK3R1;MAPK10;ATG10;RB1CC1;RAB7B

Num	Pathway id	Description	Database	Ratio_in_study	Ratio_in_pop	Rich factor	Pvalue	Padjust	First Category	Second Category	Gene_ids	Gene_names
12	map04260	Cardiac muscle contraction	KEGG PATHWAY	12/1477	162/14104	0.07407407	0.9267	1	Organismal Systems	Circulatory system	ENSGALG000000036964|ENSGALG00000032079|ENSGALG00000010812|ENSGALG000000035960|ENSGALG00000008544|ENSGALG00000015018|ENSGALG000000030112|ENSGALG00000013022|ENSGALG00000016734|ENSGALG000000009400|ENSGALG00000001459|ENSGALG00000015358	;CYTB;RYR2;;SLC8A1;CASQ2;;CACNA1C;SLC9A7;SLC8A3;TNNC1;MYH15

Num	Pathway id	Description	Database	Ratio_in_study	Ratio_in_pop	Rich factor	Pvalue	Padjust	First Category	Second Category	Gene_ids	Gene_names
11	map04662	B cell receptor signaling pathway	KEGG PATHWAY	11/1477	151/14104	0.07284768	0.9284	1	Organismal Systems	Immune system	ENSGALG00000005547|ENSGALG00000002583|ENSGALG00000027864|ENSGALG00000037943|ENSGALG00000004398|ENSGALG00000014786|ENSGALG00000010435|ENSGALG00000044525|ENSGALG00000028037|ENSGALG00000039164|ENSGALG00000052177	PIK3AP1;PIK3CD;NFKBIA;PRKCB;CARD11;PIK3R1;RASGRP3;NFKBIE;FOS;;

Num	Pathway id	Description	Database	Ratio_in_study	Ratio_in_pop	Rich factor	Pvalue	Padjust	First Category	Second Category	Gene_ids	Gene_names
40	map05022	Pathways of neurodegeneration - multiple diseases	KEGG PATHWAY	40/1477	467/14104	0.0856531	0.9291	1	Human Diseases	Neurodegenerative disease	ENSGALG00000007278|ENSGALG00000027415|ENSGALG00000008933|ENSGALG00000046098|ENSGALG00000040586|ENSGALG00000042838|ENSGALG00000015259|ENSGALG00000008444|ENSGALG00000003149|ENSGALG00000029940|ENSGALG00000000059|ENSGALG00000010812|ENSGALG00000003899	GRIN2A;GRIN2C;CASP7;;TUBA3E;TRAF2;RB1CC1;TUBAL3;;IL1B;TUBB3;RYR2;GRIA4;KIF5C;IL6;CASP18;ATG13;;MAPK10;CACNA1C;GPX7;CYTB;NOS1;WNT16;;;ITPR2;GRIA1;WNT5A;SNCAIP;MAP2K6;CASP8;PRKCB;GPX3;ND5;NOS2;WNT11B;;PTGS2;BID
1	map00040	Pentose and glucuronate interconversions	KEGG PATHWAY	1/1477	24/14104	0.04166667	0.9299	1	Metabolism	Carbohydrate metabolism	ENSGALG00000011805	
3	map00600	Sphingolipid metabolism	KEGG PATHWAY	3/1477	55/14104	0.05454545	0.9371	1	Metabolism	Lipid metabolism	ENSGALG00000023541|ENSGALG00000010902|ENSGALG00000014711	SPHK1;CERS6;PLPP1

Num	Pathway id	Description	Database	Ratio_in_study	Ratio_in_pop	Rich factor	Pvalue	Padjust	First Category	Second Category	Gene_ids	Gene_names
3	map04340	Hedgehog signaling pathway	KEGG PATHWAY	3/1477	57/14104	0.05263158	0.9464	1	Environmental Information Processing	Signal transduction	ENSGALG000000032161|ENSGALG00000054164|ENSGALG00000010133	SCUBE2;;PTCH2
8	map04110	Cell cycle	KEGG PATHWAY	8/1477	122/14104	0.06557377	0.9495	1	Cellular Processes	Cell growth and death	ENSGALG00000028005|ENSGALG00000026137|ENSGALG00000051645|ENSGALG000000029968|ENSGALG00000011881|ENSGALG00000003485|ENSGALG000000051375|ENSGALG00000010346	GADD45G;CDKN2B;CDC6;GADD45B;CCNA2;CCND3;;TGFB3
1	map00860	Porphyrin and chlorophyll metabolism	KEGG PATHWAY	1/1477	27/14104	0.03703704	0.9497	1	Metabolism	Metabolism of cofactors and vitamins	ENSGALG00000011805	
1	map04341	Hedgehog signaling pathway - fly	KEGG PATHWAY	1/1477	28/14104	0.03571429	0.955	1	Environmental Information Processing	Signal transduction	ENSGALG00000012429	BMP4
2	map00620	Pyruvate metabolism	KEGG PATHWAY	2/1477	45/14104	0.04444444	0.9571	1	Metabolism	Carbohydrate metabolism	ENSGALG00000017246|ENSGALG00000008229	ME3;ALDH7A1
1	map00592	alpha-Linolenic acid metabolism	KEGG PATHWAY	1/1477	29/14104	0.03448276	0.9597	1	Metabolism	Lipid metabolism	ENSGALG00000019233	

Num	Pathway id	Description	Database	Ratio_in_study	Ratio_in_pop	Rich factor	Pvalue	Padjust	First Category	Second Category	Gene_ids	Gene_names
1	map04130	SNARE interactions in vesicular transport	KEGG PATHWAY	1/1477	30/14104	0.03333333	0.9639	1	Genetic Information Processing	Folding, sorting and degradation	ENSGALG000000011673	STX11
1	map04745	Phototransduction - fly	KEGG PATHWAY	1/1477	30/14104	0.03333333	0.9639	1	Organismal Systems	Sensory system	ENSGALG000000017044	TRPC4
1	map00062	Fatty acid elongation	KEGG PATHWAY	1/1477	30/14104	0.03333333	0.9639	1	Metabolism	Lipid metabolism	ENSGALG000000028142	HACD4
6	map04114	Oocyte meiosis	KEGG PATHWAY	6/1477	104/14104	0.05769231	0.9677	1	Cellular Processes	Cell growth and death	ENSGALG000000031244|ENSGALG00000003149|ENSGALG00000030879|ENSGALG00000011281|ENSGALG00000013607|ENSGALG00000014071	ADCY6;;AR;SGO1;FBXO5;ITPR2
5	map04612	Antigen processing and presentation	KEGG PATHWAY	5/1477	91/14104	0.05494505	0.968	1	Organismal Systems	Immune system	ENSGALG000000030940|ENSGALG00000000775|ENSGALG00000029381|ENSGALG000000000162|ENSGALG00000035075	BLB2;CTSS;;DMB1;TAP1
1	map03020	RNA polymerase	KEGG PATHWAY	1/1477	32/14104	0.03125	0.9711	1	Genetic Information Processing	Transcription	ENSGALG000000050269	

Num	Pathway id	Description	Database	Ratio_in_study	Ratio_in_pop	Rich factor	Pvalue	Padjust	First Category	Second Category	Gene_ids	Gene_names
17	map05012	Parkinson disease	KEGG PATHWAY	17/1477	242/14104	0.07024793	0.9747	1	Human Diseases	Neurodegenerative disease	ENSGALG000000031244|ENSGALG00000003149|ENSGALG00000000059|ENSGALG000000005330|ENSGALG00000012462|ENSGALG00000031518|ENSGALG000000046098|ENSGALG00000032079|ENSGALG00000000433|ENSGALG000000040360|ENSGALG00000029500|ENSGALG00000011324|ENSGALG0000000001110	ADCY6;;TUBB3;SNCAIP;KIF5C;;;CYTB;;DUSP1;ND5;;MAPK10;ITPR2;GNAI1;TUBA3E;TUBAL3

Num	Pathway id	Description	Database	Ratio_in_study	Ratio_in_pop	Rich factor	Pvalue	Padjust	First Category	Second Category	Gene_ids	Gene_names
17	map05135	Yersinia infection	KEGG PATHWAY	17/1477	246/14104	0.06910569	0.9792	1	Human Diseases	Infectious disease: bacterial	ENSGALG000000027864|ENSGALG00000029940|ENSGALG00000011170|ENSGALG000000004370|ENSGALG00000042838|ENSGALG00000016564|ENSGALG000000011668|ENSGALG00000010915|ENSGALG00000046160|ENSGALG000000028037|ENSGALG00000026995|ENSGALG00000014786|ENSGALG0000002609	NFKBIA;IL1B;WIPF3;MAP2K6;TRAF2;PTK2B;IL8L1;IL6;;FOS;RHOG;PIK3R1;IL8;MAPK10;IFNW1;;PIK3CD
1	map03440	Homologous recombination	KEGG PATHWAY	1/1477	39/14104	0.02564103	0.9867	1	Genetic Information Processing	Replication and repair	ENSGALG000000050752	

Num	Pathway id	Description	Database	Ratio_in_study	Ratio_in_pop	Rich factor	Pvalue	Padjust	First Category	Second Category	Gene_ids	Gene_names
9	map05150	Staphylococcus aureus infection	KEGG PATHWAY	9/1477	158/14104	0.05696203	0.988	1	Human Diseases	Infectious disease: bacterial	ENSGALG000000030940|ENSGALG00000052177|ENSGALG00000003690|ENSGALG000000001565|ENSGALG00000000162|ENSGALG00000034204|ENSGALG000000040832|ENSGALG00000030002|ENSGALG00000000783	BLB2;;KRT14;C5;DMB1;;CFD;KRT18;PTAFR
1	map03420	Nucleotide excision repair	KEGG PATHWAY	1/1477	41/14104	0.02439024	0.9894	1	Genetic Information Processing	Replication and repair	ENSGALG000000050752	

Num	Pathway id	Description	Database	Ratio_in_study	Ratio_in_pop	Rich factor	Pvalue	Padjust	First Category	Second Category	Gene_ids	Gene_names
17	map04144	Endocytosis	KEGG PATHWAY	17/1477	261/14104	0.0651341	0.9903	1	Cellular Processes	Transport and catabolism	ENSGALG000000006045|ENSGALG00000033295|ENSGALG00000015096|ENSGALG00000011170|ENSGALG00000043287|ENSGALG00000010293|ENSGALG00000012462|ENSGALG00000042551|ENSGALG00000003227|ENSGALG00000007994|ENSGALG00000012454|ENSGALG00000008351|ENSGALG00000001542	SH3GL3;CAV2;SH3GL2;WIPF3;CAV1;RBP;KIF5C;;RAB11FIP4;PARD6B;CYTH4;CAV3;;IL2RB;;IL2RA;
3	map03018	RNA degradation	KEGG PATHWAY	3/1477	81/14104	0.03703704	0.9931	1	Genetic Information Processing	Folding, sorting and degradation	ENSGALG00000002167|ENSGALG00000030280|ENSGALG00000002363	PATL2;PABPC1;HSPA9
1	map00970	Aminoacyl-tRNA biosynthesis	KEGG PATHWAY	1/1477	46/14104	0.02173913	0.9939	1	Genetic Information Processing	Translation	ENSGALG00000004813	IARS1
1	map00513	Various types of N-glycan biosynthesis	KEGG PATHWAY	1/1477	48/14104	0.02083333	0.9951	1	Metabolism	Glycan biosynthesis and metabolism	ENSGALG00000019489	CHST9

<table>
<tr><th>Num</th><th>Pathway id</th><th>Description</th><th>Database</th><th>Ratio_in_study</th><th>Ratio_in_pop</th><th>Rich factor</th><th>Pvalue</th><th>Padjust</th><th>First Category</th><th>Second Category</th><th>Gene_ids</th><th>Gene_names</th></tr>
<tr><td>13</td><td>map04714</td><td>Thermogenesis</td><td>KEGG PATHWAY</td><td>13/1477</td><td>227/14104</td><td>0.05726872</td><td>0.996</td><td>1</td><td>Organismal Systems</td><td>Environmental adaptation</td><td>ENSGALG00000031244|ENSGALG00000015082|ENSGALG00000032079|ENSGALG00000008393|ENSGALG00000037869|ENSGALG00000010826|ENSGALG00000004574|ENSGALG00000049848|ENSGALG00000032736|ENSGALG00000029500|ENSGALG00000051630|ENSGALG00000011104|ENSGALG0000001062</td><td>ADCY6;RPS6;CYTB;CREB3L1;;PRKAA2;NPPA;CNR1;MGLL;ND5;;GCG;ACSL1</td></tr>
</table>

Num	Pathway id	Description	Database	Ratio_in_study	Ratio_in_pop	Rich factor	Pvalue	Padjust	First Category	Second Category	Gene_ids	Gene_names
19	map05016	Huntington disease	KEGG PATHWAY	19/1477	303/14104	0.06270627	0.9962	1	Human Diseases	Neurodegenerative disease	ENSGALG00000004083|ENSGALG00000042838|ENSGALG00000000059|ENSGALG00000032079|ENSGALG00000000433|ENSGALG00000008393|ENSGALG00000008355|ENSGALG00000038995|ENSGALG00000012462|ENSGALG00000008346|ENSGALG00000008347|ENSGALG00000004521|ENSGALG00000002950	GRIA1;TRAF2;TUBB3;CYTB;;CREB3L1;CASP8;GRIA4;KIF5C;CASP18;ATG13;GPX3;ND5;;MAPK10;GPX7;TUBA3E;RB1CC1;TUBAL3

Num	Pathway id	Description	Database	Ratio_in_study	Ratio_in_pop	Rich factor	Pvalue	Padjust	First Category	Second Category	Gene_ids	Gene_names
7	map04664	Fc epsilon RI signaling pathway	KEGG PATHWAY	7/1477	152/14104	0.04605263	0.9972	1	Organismal Systems	Immune system	ENSGALG000000025 83|ENSGALG000000 04370|ENSGALG0000 0026781|ENSGALG00 00005461 9|ENSGALG0000001 4786|ENSGALG0000 0011109|ENSGALG00 00005217 7	PIK3CD;MAP2K6;ALOX5AP;GAB2;PIK3R1;MAPK10;
1	map03022	Basal transcription factors	KEGG PATHWAY	1/1477	58/14104	0.01724138	0.9984	1	Genetic Information Processing	Transcription	ENSGALG000000484 01	
6	map05340	Primary immunodeficiency	KEGG PATHWAY	6/1477	146/14104	0.04109589	0.9985	1	Human Diseases	Immune disease	ENSGALG000000350 75|ENSGALG000000 29381|ENSGALG0000 0013372|ENSGALG00 00005217 7|ENSGALG0000003 3234|ENSGALG0000 0004170	TAP1;;IL7R;;JAK3;ADA

Num	Pathway id	Description	Database	Ratio_in_study	Ratio_in_pop	Rich factor	Pvalue	Padjust	First Category	Second Category	Gene_ids	Gene_names
7	map04141	Protein processing in endoplasmic reticulum	KEGG PATHWAY	7/1477	161/14104	0.04347826	0.9986	1	Genetic Information Processing	Folding, sorting and degradation	ENSGALG000000016893|ENSGALG00000005994|ENSGALG00000031518|ENSGALG000000042838|ENSGALG00000010560|ENSGALG00000004657|ENSGALG000000011109	UGGT2;DERL3;;TRAF2;EIF2AK2;FBXO2;MAPK10
7	map04120	Ubiquitin mediated proteolysis	KEGG PATHWAY	7/1477	163/14104	0.04294479	0.9988	1	Genetic Information Processing	Folding, sorting and degradation	ENSGALG000000042001|ENSGALG00000027786|ENSGALG00000046098|ENSGALG000000017186|ENSGALG00000007158|ENSGALG00000045534|ENSGALG000000004657	;SOCS3;;BIRC3;SOCS1;;FBXO2
3	map05310	Asthma	KEGG PATHWAY	3/1477	109/14104	0.02752294	0.9995	1	Human Diseases	Immune disease	ENSGALG000000030940|ENSGALG00000052177|ENSGALG00000000162	BLB2;;DMB1

Num	Pathway id	Description	Database	Ratio_in_study	Ratio_in_pop	Rich factor	Pvalue	Padjust	First Category	Second Category	Gene_ids	Gene_names
4	map00190	Oxidative phosphorylation	KEGG PATHWAY	4/1477	127/14104	0.03149606	0.9995	1	Metabolism	Energy metabolism	ENSGALG00000020754|ENSGALG00000029500|ENSGALG00000034294|ENSGALG00000032079	ATP6V0E2;ND5;ATP6V0D2;CYTB
23	map05014	Amyotrophic lateral sclerosis	KEGG PATHWAY	23/1477	392/14104	0.05867347	0.9996	1	Human Diseases	Neurodegenerative disease	ENSGALG00000007278|ENSGALG00000027415|ENSGALG00000008177|ENSGALG00000004083|ENSGALG00000042838|ENSGALG00000015259|ENSGALG00000008444|ENSGALG00000003149|ENSGALG00000000059|ENSGALG00000012462|ENSGALG00000008347|ENSGALG00000011324|ENSGALG00000003364	GRIN2A;GRIN2C;NOS1;GRIA1;TRAF2;RB1CC1;TUBAL3;;TUBB3;KIF5C;ATG13;;NRG2;CYTB;;;TUBA3E;MAP2K6;GPX3;ND5;NOS2;GPX7;BID

Num	Pathway id	Description	Database	Ratio_in_study	Ratio_in_pop	Rich factor	Pvalue	Padjust	First Category	Second Category	Gene_ids	Gene_names
4	map03013	Nucleocytoplasmic transport	KEGG PATHWAY	4/1477	134/14104	0.02985075	0.9997	1	Genetic Information Processing	Translation	ENSGALG00000011966|ENSGALG00000005843|ENSGALG00000049412|ENSGALG000000004665	RANGAP1;EEF1A2;;KPNA7
2	map03015	mRNA surveillance pathway	KEGG PATHWAY	2/1477	99/14104	0.02020202	0.9998	1	Genetic Information Processing	Translation	ENSGALG00000049412|ENSGALG00000030280	;PABPC1
5	map05320	Autoimmune thyroid disease	KEGG PATHWAY	5/1477	155/14104	0.03225806	0.9998	1	Human Diseases	Immune disease	ENSGALG00000030940|ENSGALG00000054104|ENSGALG00000015062|ENSGALG000000000162|ENSGALG00000052177	BLB2;;;DMB1;
3	map05330	Allograft rejection	KEGG PATHWAY	3/1477	138/14104	0.02173913	1	1	Human Diseases	Immune disease	ENSGALG00000030940|ENSGALG00000052177|ENSGALG00000000162	BLB2;;DMB1
2	map03040	Spliceosome	KEGG PATHWAY	2/1477	125/14104	0.016	1	1	Genetic Information Processing	Transcription	ENSGALG00000035453|ENSGALG00000049412	;

Num	Pathway id	Description	Database	Ratio_in_study	Ratio_in_pop	Rich factor	Pvalue	Padjust	First Category	Second Category	Gene_ids	Gene_names
4	map04740	Olfactory transduction	KEGG PATHWAY	4/1477	191/14104	0.02094241	1	1	Organismal Systems	Sensory system	ENSGALG000000008544|ENSGALG00000009400|ENSGALG00000042744|ENSGALG000000039935	SLC8A1;SLC8A3;RGS2;PDE1C
